# ICA1 affects APP processing through the PICK1‐PKCα signaling pathway

**DOI:** 10.1111/cns.14754

**Published:** 2024-06-17

**Authors:** Liangye Ji, ZiJun Meng, Xiangjun Dong, Qunxian Wang, Yanshuang Jiang, Jie Zhang, Dongjie Hu, Shipeng Guo, Weihui Zhou, Weihong Song

**Affiliations:** ^1^ Department of Pediatric Research Institute Children's Hospital of Chongqing Medical University National Clinical Research Center for Child Health and Disorders, Ministry of Education Key Laboratory of Child Development and Disorders, China International Science and Technology Cooperation base of Child development and Critical Disorders, Chongqing Key Laboratory of Child Neurodevelopment and Cognitive Disorders, Children's Hospital of Chongqing Medical University Chongqing China; ^2^ The Second Affiliated Hospital and Yuying Children's Hospital, Institute of Aging, Key Laboratory of Alzheimer's Disease of Zhejiang Province Wenzhou Medical University Wenzhou China; ^3^ Oujiang Laboratory (Zhejiang Lab for Regenerative Medicine, Vision and Brain Health) Wenzhou China

**Keywords:** Alzheimer's disease, APP, ICA1, PICK1, PKCα

## Abstract

**Aims:**

Islet cell autoantigen 1 (ICA1) is involved in autoimmune diseases and may affect synaptic plasticity as a neurotransmitter. Databases related to Alzheimer's disease (AD) have shown decreased ICA1 expression in patients with AD. However, the role of ICA1 in AD remains unclear. Here, we report that ICA1 expression is decreased in the brains of patients with AD and an AD mouse model.

**Results:**

The ICA1 increased the expression of amyloid precursor protein (APP), disintegrin and metalloprotease 10 (ADAM10), and disintegrin and metalloprotease 17 (ADAM17), but did not affect protein half‐life or mRNA levels. Transcriptome sequencing analysis showed that ICA1 regulates the G protein‐coupled receptor signaling pathway. The overexpression of ICA1 increased PKCα protein levels and phosphorylation.

**Conclusion:**

Our results demonstrated that ICA1 shifts APP processing to non‐amyloid pathways by regulating the PICK1‐PKCα signaling pathway. Thus, this study suggests that ICA1 is a novel target for the treatment of AD.

## Graphical abstract

In this study, we demonstrated that ICA1 alters APP processing and shifts it to non‐amyloid pathways by regulating the PICK1‐PKCα signaling pathway, and we suggested ICA1 as a potential therapeutic target for AD therapy by modulating APP processing.

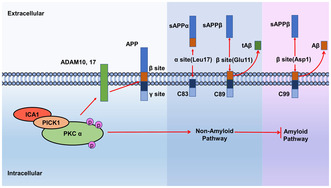

## 1 INTRODUCTION

Alzheimer's disease (AD) is the most common neurodegenerative disorder that causes dementia.^1^ The clinical manifestations of AD include the progressive loss of short‐term memory and later long‐term memory, impairments in language and behavior, and disorientation in time and space.^2^ The pathological features of AD are characterized by extracellular senile plaques, intracellular neurofibrillary tangles (NTFs), and neuron death.^1^ Amyloid β protein (Aβ), a 4‐kDa peptide identified as the key component of senile amyloid, is derived from the sequential cleavage of β‐ and γ‐secretase of amyloid precursor protein (APP) in the amylogenic pathway.^3^, ^4^, ^5^ When there are no interfering factors, the majority of APPs undergo processing by α‐secretase at the Leu17 position within the Aβ domain. This process results in the formation of a large soluble fragment known as sAPPα and a membrane‐bound C‐terminal fragment composed of 83 amino acids, referred to as CTFα or C83. Subsequently, γ‐secretase cleaves C83, leading to the production of P3 fragments and CTFγ. This process is characterized as a non‐amyloidogenic pathway. In AD, the amyloidogenic process begins with the cleavage of APP by β‐site APP cleaving enzyme 1 (BACE1). This cleavage at the Asp1 location results in the formation of a C99 fragment and sAPPβ, following which γ‐secretase cleaves C99, leading to the formation of Aβ.^5^, ^6^, ^7^ APP can also be cleaved by BACE1 at the Glu11 position, resulting in the formation of a C89 fragment and sAPPβ. Alternatively, it can be cleaved by BACE2 at the Phe20 position, leading to the production of a C80 fragment and sAPPθ. Subsequently, γ‐secretase further cleaves C89 and C80, generating truncated Aβ and P3θ, respectively.^8^, ^9^ The imbalance between production and clearance causes Aβ to deposit and form plaques that promote intracellular neurofibrillary tangles, oxidative stress, neuroinflammation, neuronal death, and synaptic loss.^10^, ^11^ Although many studies have explored APP processing and the pathogenesis of AD, the mechanism underlying APP processing is still being investigated, and there is currently no effective treatment for AD.

To find a new target, we searched AD‐related databases. Transcriptome sequencing analysis of patients with AD has shown that Islet cell autoantigen 1 (ICA1) is significantly reduced in the brains of patients with AD,^12^ suggesting that ICA1 may be involved in the development and progression of AD, but the exact role is unclear. Islet cell autoantigen 1 (ICA1), also known as ICA69 (Islet cell antigen p69), is located on chromosome 7p21.3 and consists of the Bin/amphiphysin/Rvs (BAR) and ICAC domains. The N‐terminal 1–256 amino acids of ICA1 form the BAR domain, which has lipid‐binding ability and can bind to other proteins with the BAR domain.^13^, ^14^, ^15^, ^16^ The C‐terminal 257–480 amino acids form the ICAC domain, and the amino acid sequences are highly evolutionarily conserved.^17^

The ICA1 protein was first identified as a cross‐reacting protein in cloned rat β‐islet tumor cell extracts or isolated from BB rat islets using rat anti‐bovine serum albumin antiserum.^18^ Thus, ICA1 is thought to be an autoantigen that causes Type I diabetes (IDDM).^19^ It functions as an autoimmune target antigen in primary Sjogren's syndrome, rheumatoid arthritis, and other autoimmune diseases.^20^, ^21^ ICA1 is widely expressed throughout the body, primarily in the pancreas, muscles, digestive tract, and brain. Immunoelectron microscopy has shown ICA1 subcellular localization in the endoplasmic reticulum, Golgi complex, and vesicles, suggesting the role of this neuroendocrine molecule in cellular protein transport and processing.^22^, ^23^

The Rab GTPases are a large family of GTPases that control membrane trafficking by recruiting effector proteins, such as sorting adaptors, tethering factors, kinases, phosphatases, and motors, mediate the various downstream functions of Rab GTPases, including membrane identity, vesicle budding, uncoating, motility, and fusion.^24^ It has been shown that Rab2 binds to ICA1 in a GTP‐dependent fashion, recruits it to membranes in insulinoma INS‐1 cells, and regulates the transport of coat protein complex I(COPI) vesicles between the endoplasmic reticulum and the Golgi complex.^25^

In the brain, more than three‐fourths of ICA1 and proteins interacting with C kinase 1(PICK1) bind to each other through the BAR domain to form heterogenic complexes.^15^ PICK1 is an adaptor protein that attaches to and arranges the subcellular positioning of a variety of membrane proteins and has an interactive relationship with protein kinase Cα (PKCα).^26^ PICK1 has a PDZ (PSD‐95/Dlg/ZO1) domain that engages with the C terminal of AMPA receptors. Additionally, the BAR domain of PICK1 attaches to the membrane, facilitating the transport of AMPA receptors.^27^ However, ICA1 binds to PICK1, affects AMPA receptor recruitment, and influences synaptic plasticity.^15^, ^28^

Here, our observations indicated that in the APP23/PS45 mouse model, the levels of ICA1 were lower than those in the wild‐type mice of the same age. Furthermore, ICA1 affects APP processing through the PICK1‐PKCα signaling pathway. Finally, we treated cells overexpressing ICA1 with the PKCα inhibitor Go 6983, which rescued the increased protein expression. Our findings show that ICA1 is reduced in AD and affects APP processing through the PICK1‐PKCα signaling pathway.

## 2 MATERIALS AND METHODS

### 2.1 Cell culture and treatment

All cell lines were cultured in 90% Dulbecco's modified Eagle's medium (DMEM) (Gibco) and 10% fetal bovine serum (FBS) and maintained in an incubator at 37°C under 5% CO_2_. The 2 EB2 cell line is HEK 293 cells stably transfected human APP695 with Swedish mutation and BACE1 and cultured in 90% DMEM and 10% FBS containing 100 μg/mL zeocin (Invitrogen) and 50 μg/mL Geneticin (Gibco). The 20E2 cell line is HEK 293 cells stably transfected human APP695 with a Swedish mutation and cultured in 90% DMEM and 10% FBS containing 50 μg/mL Geneticin. The SAS cell line is SH‐SY5Y cells stably transfected human APP695 with Swedish mutation and cultured with 90% DMEM and 10% FBS containing 100 μg/mL zeocin. For PKC inhibition, 2 μM Go 6983 (HY‐13689, MedChemExpress) or 100 nM aprinocarsen sodium (HY‐148413, MedChemExpress) were used to treat the 20E2 cells overexpressing ICA1 for 24 h.

### 2.2 Cycloheximide treatment

The 20E2 cells were seeded in a 6 cm dish to proliferate to 80%–90% density and then transfected with the ICA1 plasmid. The 20E2 cells were seeded in a 6‐well plate the next day to proliferate to 95%–100% of density and then treated with 20 μg/mL cycloheximide (HY‐12320, MedChemExpress) for 0 h, 2 h, 4 h, 8 h, 12 h, 24 h, or 0 h, 15 min, 30 min, 1 h, 2 h, 4 h.

### 2.3 Plasmids/si‐RNA and transfection

For ICA1 plasmids construction, cDNA was extracted from HEK 293 cells as a template and cloned into the pcDNA4‐myc‐His vector. Primer sequences 5'‐TACCGAGCTCGGATCCGCCACCATGTCAGGACACAAATGCAG‐3′ (sense strand of ICA1) and 5'‐TCGAAGGGCCCTCTAGACTCGAGTGCATTGAGCAATTCGTGTT‐3′ (antisense strand of ICA1) were used to construct the ICA1 plasmid. Primer sequences 5'‐CGAAUUGCUCAAUGCAUGAAUTT‐3′ (sense strand of si‐ICA1) and 5'‐AUUCAUGCAUUGAGCAAUUCGTT‐3′ (antisense strand of si‐ICA1) were constructed to knock down ICA1 expression. 2 EB2, 20E2, or SAS cells were seeded into a six‐well plate grown to 80%–90% cell density, and then transfected with 2 μg ICA1 plasmid or si‐ICA1 at a MOI of 20 with lipo2000 (Invitrogen) according to the reagent protocol. Protein was collected after 36 h.

### 2.4 Western blot analysis

Cells and brain tissues were lysed in RIPA lysis buffer supplemented with protease inhibitor (Roche) and phosphatase inhibitor (Roche). The solutions were ultrasonicated and centrifuged at 12000 rpm × 15 min at 4°C. The supernatants were removed to new tubes, diluted with 5 × Protein Sample Loading Buffer (LT‐101, Shanghai Epizyme Biomedical Technology), boiled, and then resolved on 10%, 12.5% tri‐glycine, and 16% tri‐tricine SDS‐PAGE and transferred on to a polyvinylidene fluoride membrane with a 0.22 μm aperture (Millipore). The targeted proteins (30 μg) were immunoblotted with the primary antibody overnight at 4°C. After incubation with goat anti‐rabbit IgG (Beyotime, 1:5, 000) or goat anti‐mouse IgG (Beyotime, 1:5, 000) at room temperature for 1.5 h, the protein was detected with the Bio‐Rad Imager using ECL Western blotting substrate (Bio‐Rad). Antibodies against ICA1 (A17500, ABclonal, 1:1, 000) and Presenilin 1 (ab76083, abcam, 1:1, 000) were used to detect the ICA1 and PS1 bands, respectively. BACE1 (#5606, CST, 1:1, 000) and ADAM10 (ab124695, abcam, 1:1, 000) antibodies were used to detect BACE1 and ADAM10, respectively. The APP and CTF bands were assayed using a C20 polyclonal antibody produced in our laboratory. The Gapdh (60004–1‐1 g, 1:200, 000) and PICK1 (A1519, 1:1, 000) antibodies were purchased from Proteintech and ABclonal, respectively. The antibodies of PKCα (#2056, CST, 1:1, 000) and p‐PKCα (#9375, CST, 1:1, 000) were purchased for testing PKCα and p‐PKCα. All original images can be found in File S2.

### 2.5 Transcriptome sequencing

Total RNA was extracted using Trizol reagent (Thermo Fisher, 15,596,018) following the manufacturer's procedure. The total RNA quantity and purity were analyzed with the Bioanalyzer 2100 and RNA 6000 Nano LabChip Kit (Agilent, 5067–1511). High‐quality RNA samples with an RIN number >7.0 were used to construct a sequencing library. After total RNA was extracted, mRNA was purified from the total RNA (5 ug) using Dynabeads Oligo (dT) (Thermo Fisher) with two rounds of purification. Following purification, the mRNA was fragmented into short fragments using divalent cations under elevated temperature (Magnesium RNA Fragmentation Module (NEB, cat.e6150) at 94°C 5–7 min). Then the cleaved RNA fragments were reverse‐transcribed to create the cDNA by SuperScript™ II Reverse Transcriptase (Invitrogen, cat. 1,896,649), which were next used to synthesize U‐labeled second‐stranded DNAs with E. coli DNA polymerase I (NEB, cat.m0209), RNase H (NEB, cat.m0297) and dUTP Solution (Thermo Fisher, cat.R0133). An A‐base was then added to the blunt ends of each strand, preparing them for ligation to the indexed adapters. Each adapter contained a T‐base overhang for ligating the adapter to the A‐tailed fragmented DNA. Dual‐index adapters were ligated to the fragments, and size selection was performed with AMPureXP beads. After the heat‐labile UDG enzyme (NEB, cat.m0280) treatment of the U‐labeled second‐stranded DNAs, the ligated products were amplified with PCR by the following conditions: initial denaturation at 95°C for 3 min; 8 cycles of denaturation at 98°C for 15 s; annealing at 60°C for 15 s; and extension at 72°C for 30 s; and then final extension at 72°C for 5 min. The average insert size for the final cDNA libraries was 300 ± 50 bp. At last, we performed the 2 × 150 bp paired‐end sequencing (PE150) on an Illumina Novaseq™ 6000 (LC‐Bio Technology Co., Ltd.) following the vendor's recommended protocol. All data were analyzed by R (version: 3.6). We used the human ensembl database version 107 genome for mapping.^29^ We aligned the reads of all samples to the reference genome using HISAT2 (https://daehwankimlab.github.io/hisat2/, version:hisat2‐2.2.1) package, which initially remove a portion of the reads based on quality information accompanying each read and then maps the reads to the reference genome. The mapped reads of each sample were assembled using StringTie (http://ccb.jhu.edu/software/stringtie/, version:stringtie‐2.1.6) with default parameters. Gene differential expression analysis was performed by DESeq2 software (version: 1.22.2) between two different groups. The genes with the parameter of false discovery rate (FDR) below 0.05 and an absolute fold change of ≥2 were considered differentially expressed genes. Differentially expressed genes were then subjected to enrichment analysis of GO functions and KEGG pathways in OmicStudio.^30^ GO terms meeting this condition with *p* < 0.05 were defined as significantly enriched GO terms in DEGs. We performed gene set enrichment analysis using software GSEA (v4.1.0) and MSigDB to identify whether a set of genes in specific GO terms, KEGG pathways, DO terms (for Homo sapiens), and Reactome (for a few model animals) shows significant differences in two groups. Only |NES|>1, NOM *p*‐val <0.05, FDR *q*‐val <0.25 were considered to be different in two groups.^31^, ^32^ NES is the normalized enrichment score after correction. NOM *p*‐val is *p*‐value, a statistical analysis of enrichment scores used to indicate the credibility of enrichment results.

### 2.6 Statistical analysis

All data were shown as mean ± SEM, and all results were analyzed using Shapiro‐Wilk test to assess data distribution. Two‐tailed Student's *t*‐test, two‐tailed Welch's *t*‐test, one‐way ANOVA test, and two‐way ANOVA test were used to analyze parametric data appropriately. Non‐parametric data were assessed by Mann‐Whitney test.

## 3 RESULTS

### 3.1 ICA1 was reduced in the brains of AD

**FIGURE 1:**
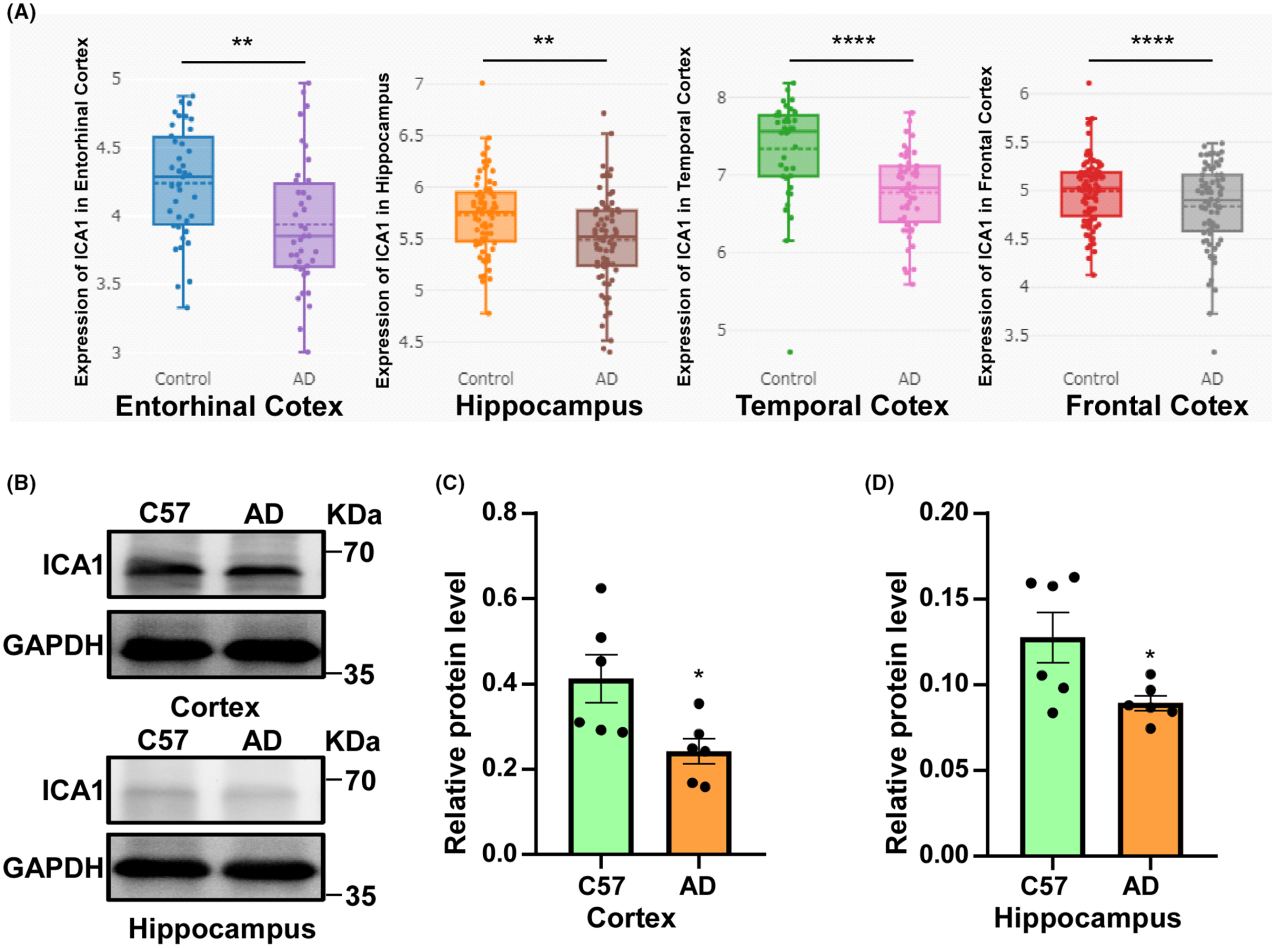
ICA1 was reduced in AD. (A) ICA1 expression was significantly reduced in the entorhinal cortex, hippocampus, temporal cortex, and frontal cortex in patients with AD than in controls (**p = 0.003, **p = 0.005, ****p < 0.0001, ****p < 0.0001, respectively, log_2_ FoldChange is −0.32, −0.22, −0.6, −0.28, respectively). (B) The expression of ICA1 in the cortex and hippocampus of APP23/PS45 mice was reduced compared with that of C57 mice. (C) Quantification of the relative protein level of ICA1 in the APP23/PS45 mice cortex compared to that in C57 mice (*p < 0.05, APP23/PS45 *n* = 6, C57 *n* = 6). (D) Quantification of the relative protein level of ICA1 in APP23/PS45 mice hippocampus compared to that in C57 mice (*p < 0.05, APP23/PS45 *n* = 6, C57 *n* = 6). The data for each group conformed to a normal distribution by Shapiro‐Wilk test. *p* Value was determined by a two‐tailed Welch's *t*‐test or a two‐tailed Student's *t*‐test.

To verify the decreased expression of ICA1 in AD, we searched for ICA1 in the Alzdata database and performed the differential expression analysis.^33^ We found that the mRNA levels of ICA1 in various parts of the brain in patients with AD were reduced compared to those in normal individuals (Figure 1A), suggesting that ICA1 may play a role in the pathological process of AD. To further investigate its role in AD, we extracted cortical and hippocampal tissue proteins from 3‐month‐old APP23/PS45 and C57 mice (APP23/PS45 *n* = 6, C57 *n* = 6) and detected the expression of ICA1 by western blotting (WB). The expression of ICA1 was also reduced in the cortex and hippocampus of APP23/PS45 mice (*p* < 0.05, Figure 1B–D). These data suggest that ICA1 expression is reduced in the brain in AD. Since Aβ, which is generated through APP processing, is the most critical factor in the development of AD. We then first investigated whether ICA1 affects APP processing.

### 3.2 ICA1 overexpression changed APP processing

**FIGURE 2:**
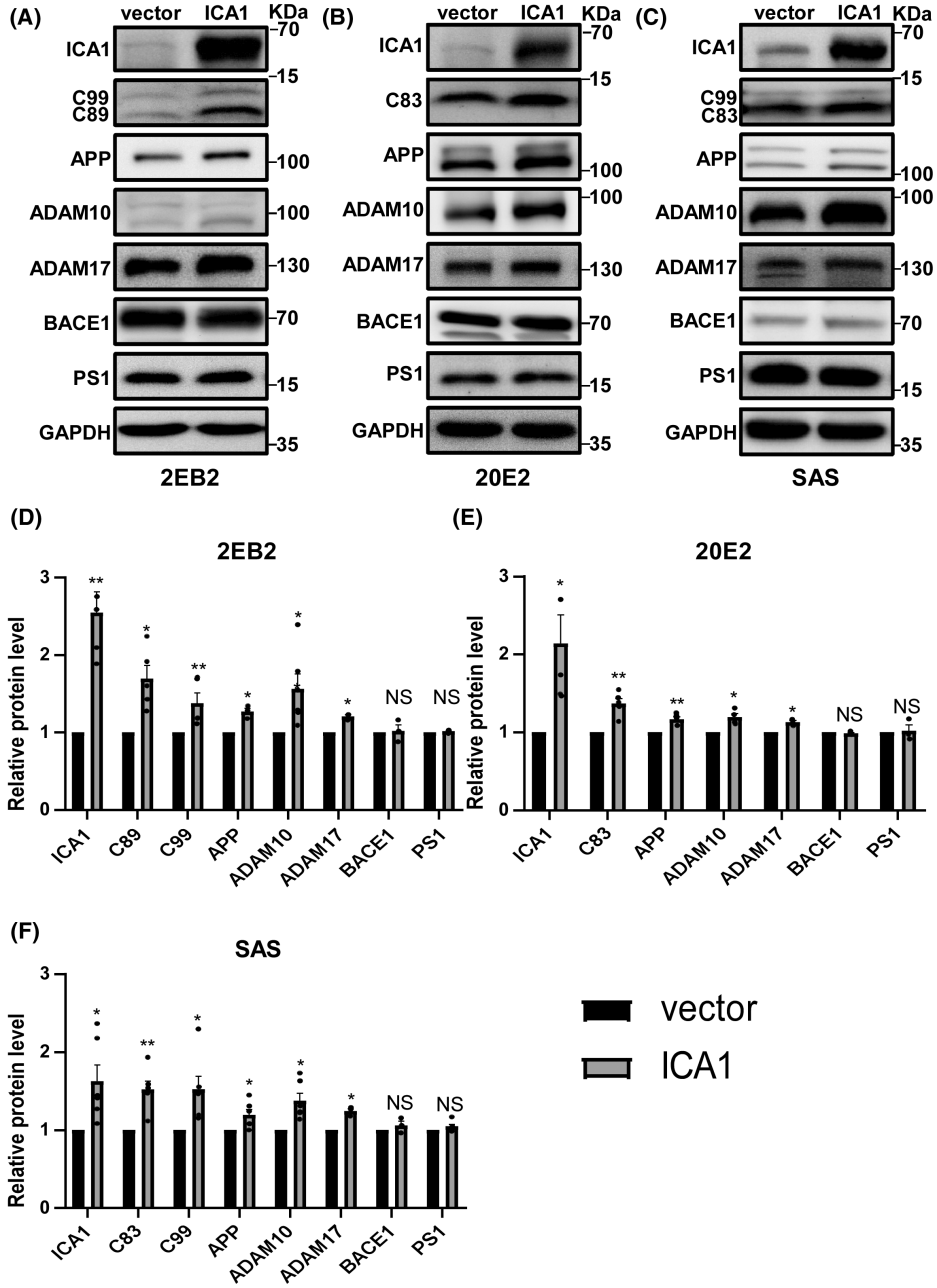
ICA1 overexpression changed APP processing. (A) The effect of ICA1 overexpression on APP processing in 2 EB2. (B) The effect of ICA1 overexpression on APP processing in 20E2. (C) The effect of ICA1 overexpression on APP processing in SAS. (D) Quantification of the relative protein level of APP(*n* = 3), ADAM10(*n* = 6), ADAM17(*n* = 3), BACE1(*n* = 3), PS1(*n* = 3), C89(*n* = 5), C99(*n* = 5) and ICA1 (*n* = 4) in the ICA1 overexpression group compared to the vector group in 2 EB2, *p < 0.05, **p < 0.01. (E) Quantification of the relative protein level of APP(*n* = 5), ADAM10(*n* = 4), ADAM17(*n* = 3), BACE1(*n* = 3), PS1(*n* = 3), C83(*n* = 5) and ICA1(*n* = 5) in the ICA1 overexpression group compared to the vector group in 20E2, *p < 0.05, **p < 0.01. (F) Quantification of the relative protein levels of APP (*n* = 3), ADAM10 (*n* = 3), ADAM17 (*n* = 3), BACE1 (*n* = 3), PS1 (*n* = 3), C83 (*n* = 3), C99 (*n* = 3), and ICA1 (*n* = 3) in the ICA1 overexpression group compared to the vector group in SAS, *p < 0.05, **p < 0.01. The data for each group conformed to a normal distribution by Shapiro‐Wilk test, except for C99 in 2 EB2. *p* Value was determined by a two‐tailed Welch's *t*‐test or Mann‐Whitney test. The 2 EB2 cell line is HEK 293 cells stably transfected human APP695 with a Swedish mutation and BACE1. The 20E2 cell line is HEK 293 cells stably transfected human APP695 with a Swedish mutation. The SAS cell line is SH‐SY5Y cells stably transfected human APP695 with a Swedish mutation.

To assess the effect of ICA1 on AD, the plasmid expressing ICA1 was transfected into 2 EB2, 20E2, and SAS cells, and APP processing was detected in vitro (File S1, Figure S2A). The protein levels of APP, APP‐CTFs, ADAM10, ADAM17, BACE1, and PS1 in cell lysates were detected by WB. Quantification revealed that the relative protein levels of C89 and C99 (*n* = 5, *p* < 0.05, *p* < 0.01 respectively, Figure 2A,D) in 2 EB2, C83 (*n* = 5, *p* < 0.01, Figure 2B,E) in 20E2, and C83 and C99 (*n* = 3, *p* < 0.01, *p* < 0.05 respectively, Figure 2C,F) in SAS cells were significantly higher in the overexpression group than in the vector group. Therefore, we tested the expression of APP, ADAM10, ADAM17, BACE1, and PS1. In all cell lines, the relative protein levels of APP (*n* = 3–5, *p* < 0.05, *p* < 0.01, *p* < 0.05, respectively, Figure 2A–F), ADAM10 (*n* = 3–6, *p* < 0.05, *p* < 0.05, *p* < 0.05, respectively, Figure 2A–F), and ADAM17 (*n* = 3, *p* < 0.05 for all, Figure 2A–F) were sharply increased in the overexpression group relative to those in the controls; however, ICA1 had no effect on the protein levels of BACE1 and PS1 (*n* = 3, *p* > 0.05 for all, Figure 2F). These data indicate that ICA1 overexpression affects APP processing and increases α‐secretase cleavage of APP.

### 3.3 ICA1 knockdown decreases APP, ADAM10, and ADAM17 expression

**FIGURE 3:**
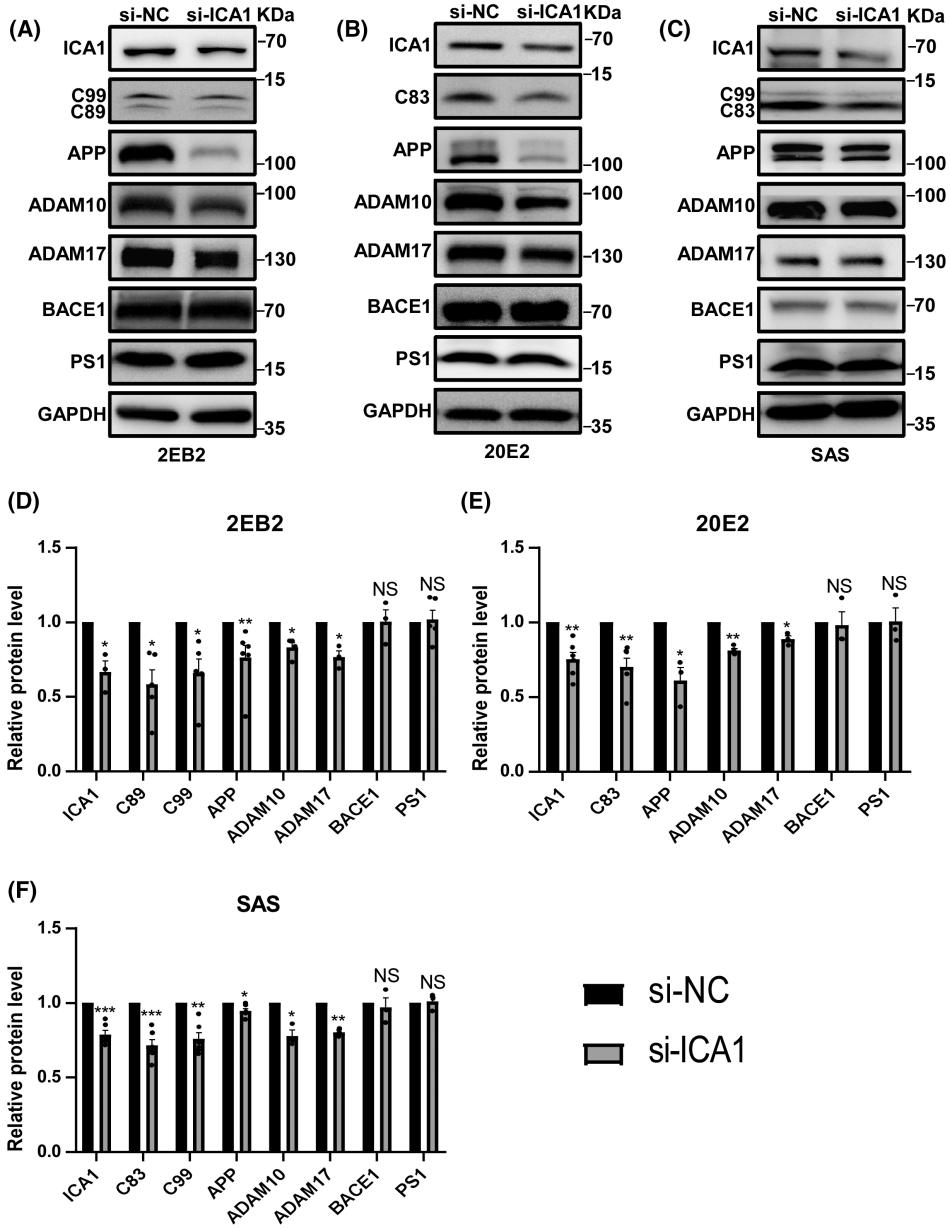
ICA1 knockdown decreases APP, ADAM10, and ADAM17 expression. (A) The effect of knockdown ICA1 on APP processing in 2 EB2. (B) The effect of knockdown ICA1 on APP processing in 20E2. (C) The effect of knockdown ICA1 on APP processing in SAS. (D) Quantification of the relative protein level of APP (*n* = 6), ADAM10 (*n* = 4), ADAM17 (*n* = 3), BACE1 (*n* = 3), PS1 (*n* = 5), C89 (*n* = 5), C99 (*n* = 5) and ICA1 (*n* = 3) in the knockdown group compared to the vector group in 2 EB2, *p < 0.05, **p < 0.01. (E) Quantification of the relative protein level of APP (*n* = 3), ADAM10 (*n* = 4), ADAM17 (*n* = 3), BACE1 (*n* = 3), PS1 (*n* = 3), C83 (*n* = 6) and ICA1 (*n* = 6) in the knockdown group compared to the vector group in 20E2, *p < 0.05, **p < 0.01. (F) Quantification of the relative protein level of APP (*n* = 3), ADAM10 (*n* = 3), ADAM17 (*n* = 3), BACE1 (*n* = 3), PS1 (*n* = 3), C83 (*n* = 3), C99 (*n* = 3) and ICA1 (*n* = 3) in the knockdown group compared to the vector group in SAS, *p < 0.05, **p < 0.01, ***p < 0.001. The data for each group conformed to a normal distribution by Shapiro‐Wilk test, except for APP in 2 EB2 and BACE1 in 20E2. *p* Value was determined by a two‐tailed Student's *t*‐test or Mann‐Whitney test. The 2 EB2 cell line is HEK 293 cells stably transfected human APP695 with a Swedish mutation and BACE1. The 20E2 cell line is HEK 293 cells stably transfected human APP695 with a Swedish mutation. The SAS cell line is SH‐SY5Y cells stably transfected human APP695 with a Swedish mutation.

To further determine the effect of ICA1 on APP processing, an experiment was set up to knock down ICA1 in 2 EB2, 20E2, and SAS cells (File S1, Figure S2B). We conducted the ELISA experiment using cell supernatant and found that Aβ40 and Aβ42 increased, also the ratio of Aβ42 to Aβ40 (File S1, Figure S2C–E). Quantification showed that the relative protein levels of C89 and C99 (*n* = 5, *p* < 0.05 for both, Figure 3A,D) in 2 EB2, C83 (*n* = 6, *p* < 0.01, Figure 3B,E) in 20E2, and C83 and C99 (*n* = 3, *p* < 0.001, *p* < 0.01, respectively, Figure 3C,F) in SAS were significantly lower in the knockdown group than in the negative control group. Then the expression of APP, ADAM10, ADAM17, BACE1, and PS1 was tested. The results showed that the relative protein levels of APP (*n* = 3–6, *p* < 0.01, *p* < 0.05, *p* < 0.05, respectively, Figure 3A–F), ADAM10 (*n* = 3–4, *p* < 0.05, *p* < 0.01, *p* < 0.05, respectively, Figure 3A–F), and ADAM17 (*n* = 3, *p* < 0.05, *p* < 0.05, *p* < 0.01, respectively, Figure 3A–F) were significantly decreased in all cell lines. Knockdown of ICA1 also had no effect on BACE1 or PS1 expression (*n* = 3–5, *p* > 0.05, for all, Figure 3A–F). These data further suggest that ICA1 alters APP processing.

### 3.4 ICA1 did not affect the degradation or mRNA level of APP, ADAM10, and ADAM17

**FIGURE 4:**
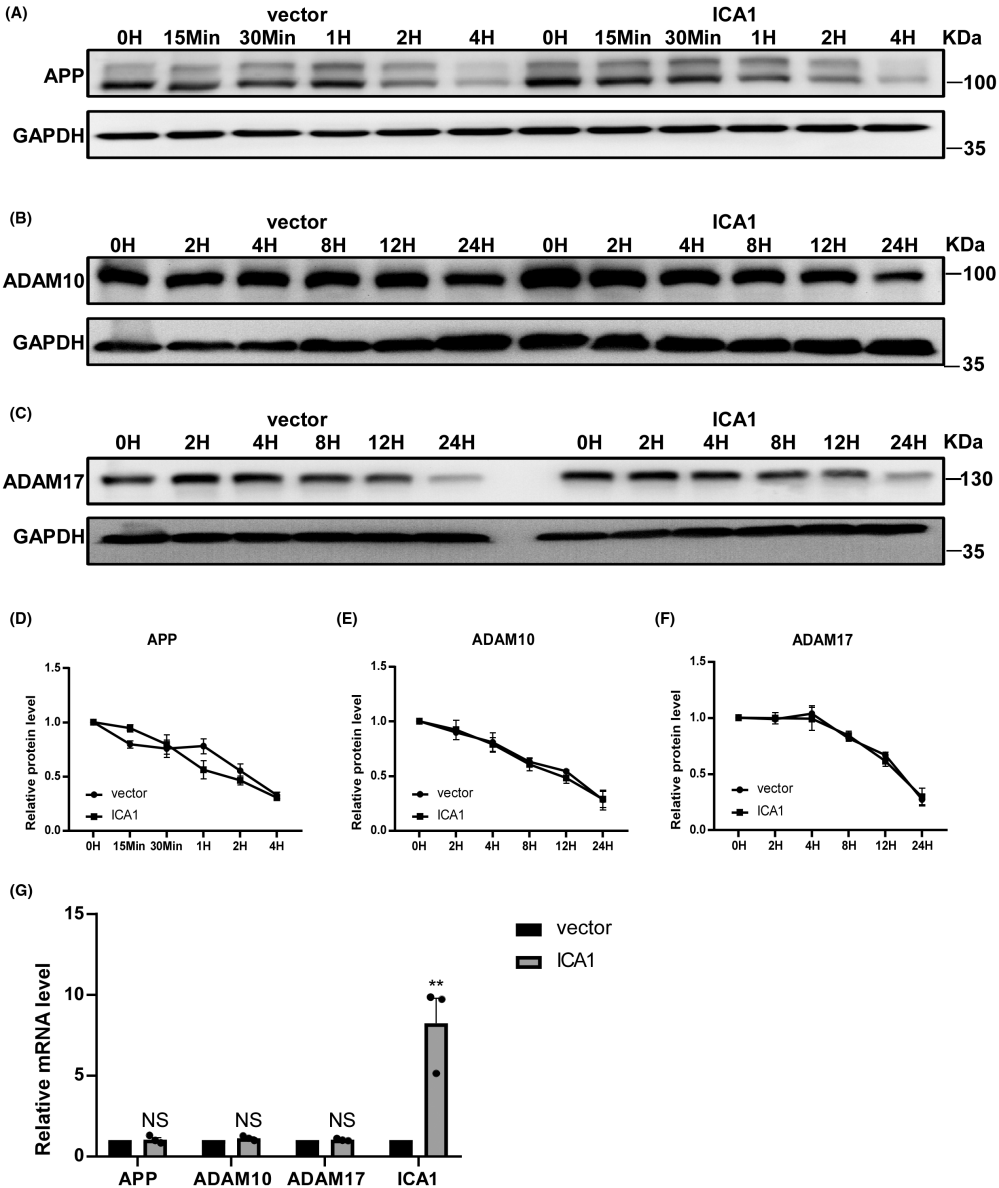
ICA1 did not affect the degradation or mRNA level of APP, ADAM10, and ADAM17. (A) ICA1 did not affect the degradation of APP. (B) ICA1 did not affect the degradation of ADAM10. (C) ICA1 did not affect the degradation of ADAM17. (D) Quantification of the expression of APP (*n* = 4, *p* > 0.05). (E) Quantification of the expression of ADAM10 (*n* = 3, *p* > 0.05). (F) Quantification of the expression of ADAM17 (*n* = 3, *p* > 0.05). (G) ICA1 did not affect the transcription of APP, ADAM10, and ADAM17, **p < 0.01. The data for each group conformed to a normal distribution by Shapiro‐Wilk test. *p* Value was determined by a two‐way ANOVA test (D–F) and a two‐tailed Student's *t*‐test (G).

To explore whether the effect of ICA1 on APP processing was caused by impaired degradation, 20E2 cells overexpressing ICA1 were treated with cycloheximide (CHX), and the protein levels of APP, ADAM10, and ADAM17 were analyzed. Quantification revealed that ICA1 had no effect on the catabolism of APP, ADAM10, and ADAM17 (*n* = 4 for APP, *n* = 3 for ADAM10, *n* = 3 for ADAM17, *p* > 0.05 for all, Figure 4A–F). These data indicated that ICA1 does not affect APP, ADAM10, or ADAM17 degradation. We then extracted RNA from HEK 293 cells overexpressing ICA1 to determine whether ICA1 enhanced synthesis using qPCR to detect the mRNA levels of APP, ADAM10, and ADAM17. The results showed that ICA1 overexpression did not affect the transcription of APP, ADAM10, or ADAM17 (*n* = 3, *p* > 0.05 for all except *p* < 0.01 for ICA1, Figure 4G).

### 3.5 Transcriptome sequencing analysis of ICA1 knockdown in 20E2

**FIGURE 5:**
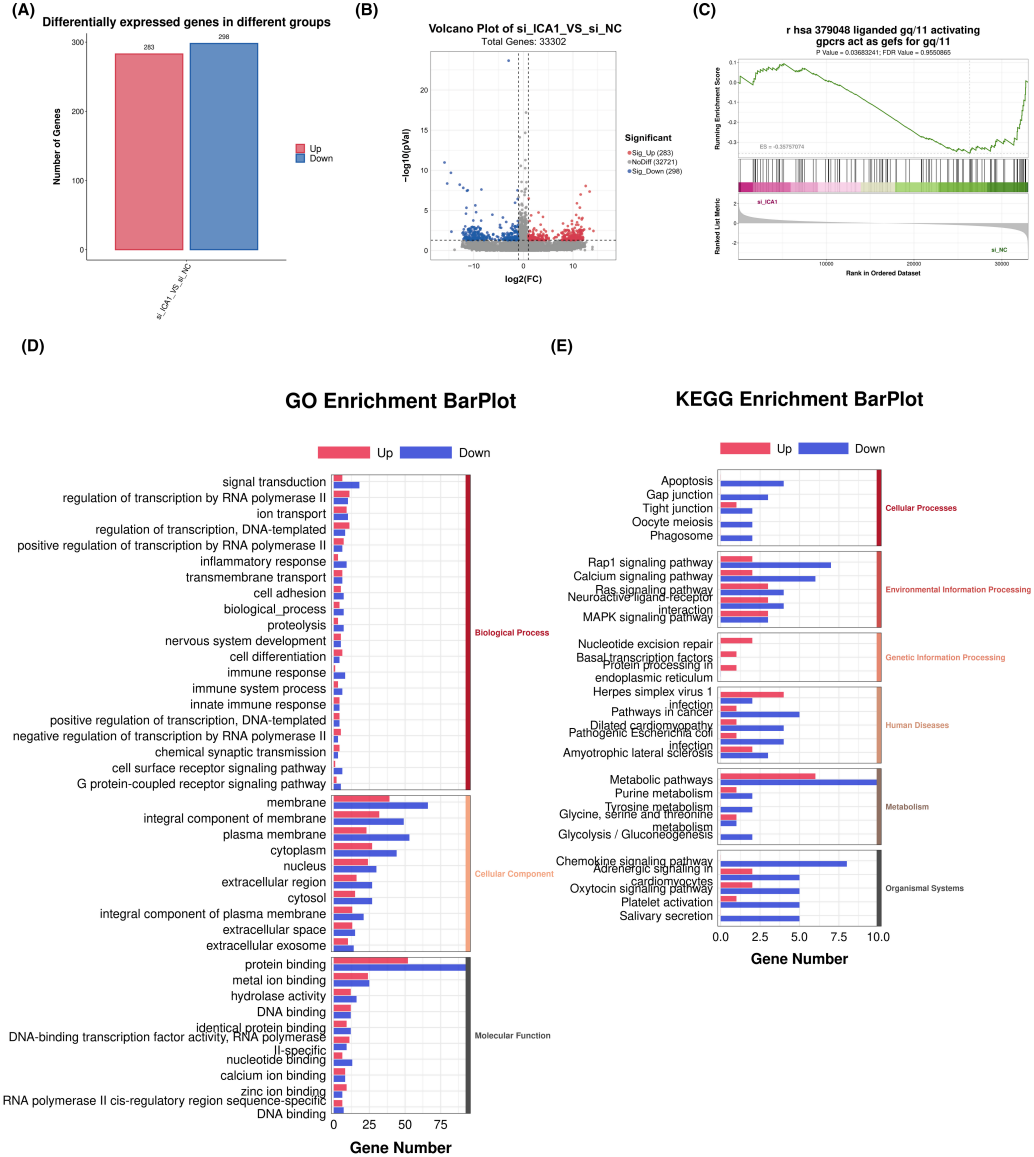
Transcriptome sequencing analysis of ICA1 knockdown in 20E2. (A) Transcriptome analysis was performed on ICA1 knockdown 20E2 cells, and 581 differentially expressed genes were found. (B) Volcano plot of differential genes. (C) GSEA analysis of differential genes in 20E2 after knockdown of ICA1. (D) GO enrichment analysis of differential genes in 20E2 after knockdown of ICA1. (E) KEGG enrichment analysis of differential genes in 20E2 after knockdown of ICA1.

To explore the specific mechanism of ICA1 affecting APP processing, we performed transcriptome sequencing analysis between the knockdown and negative control groups (*n* = 3). Differential expression analysis identified 581 genes, of which 283 genes were significantly up‐regulated and 298 genes were significantly down‐regulated (Figure 5A,B, Table S1). Gene Ontology (GO) and Kyoto Encyclopedia of Genes and Genomes (KEGG) terms (Figure 5D,E, Table S1) were analyzed. In GO terms, “signal transdution,” “regulation of transcription by RNA polymerase II,” “regulation of transcription, DNA‐templated,” “ion transport,” “membrane,” “integral component of membrane,” “cytoplasm,” “nucleus,” “plasma membrane,” “protein binding,” and “G protein‐coupled receptor signaling pathway” were enriched. KEGG pathway enrichment analysis revealed that these DEGs were mainly enriched in the apoptosis, rap1 signaling pathway, calcium signaling pathway, ras signaling pathway, neuroactive ligand‐receptor interaction, MAPK signaling pathway, metabolism pathway, and chemokine signaling pathway. Consistently, gene set enrichment analysis (GSEA) showed significant enrichment in “liganded gq/11 activating gpcrs act as gefs for gq/11” (Figure 5C). The gq/11 is upstream of PKC. It activates PKC by increasing PLC activity to produce the intracellular messengers inositol triphosphate (IP3) and diacylglycerol (DAG).

### 3.6 ICA1 affects APP processing through PICK1‐PKCα signaling pathway

**FIGURE 6:**
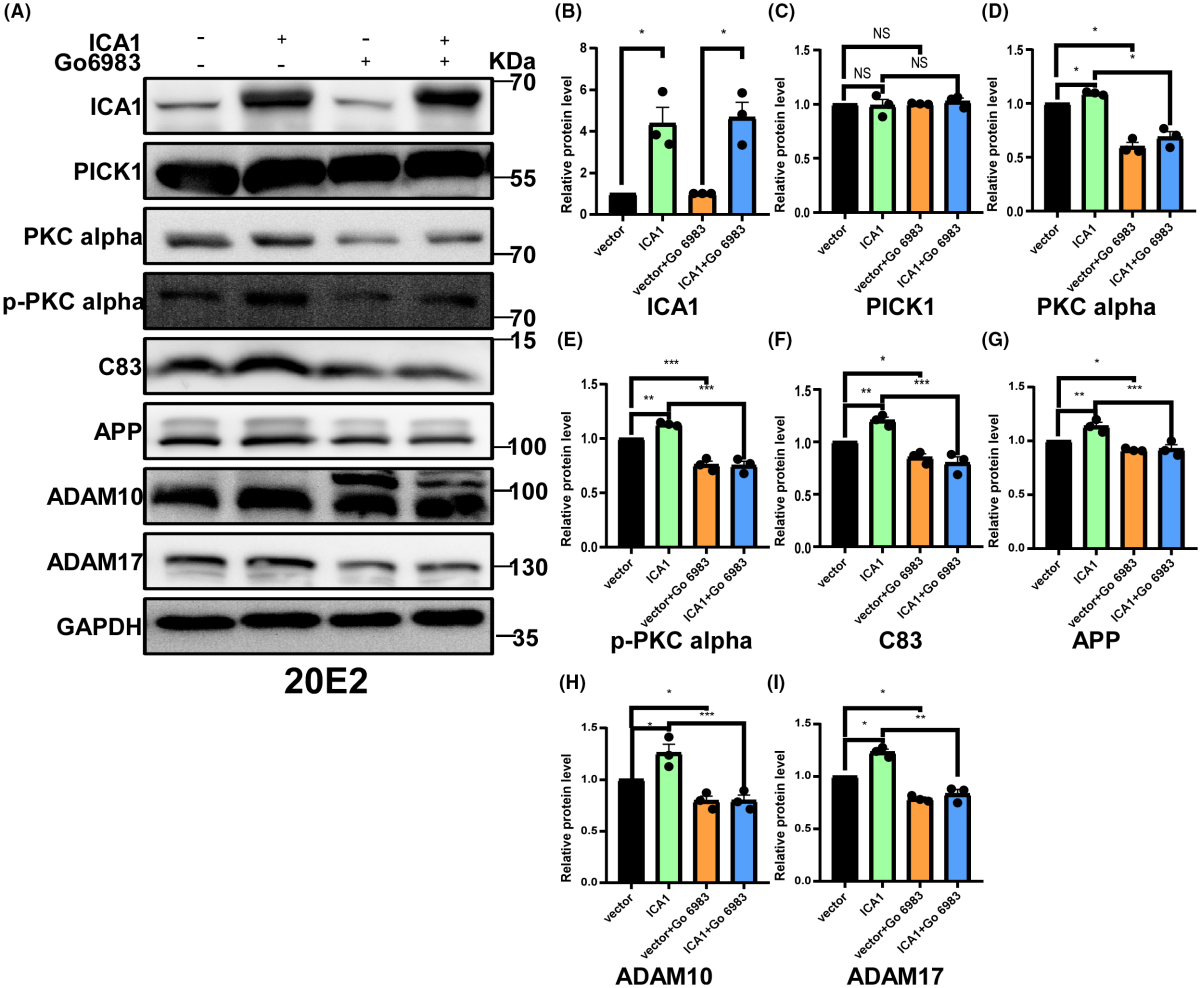
ICA1 affects APP processing through PICK1‐PKCα signaling pathway. (A) Western blotting showed that ICA1 affects APP processing via the PICK1‐PKCα signaling pathway. (B) Quantification of the relative protein level of ICA1 (*n* = 3) in the ICA1 overexpression group compared to the vector group in 20E2, *p < 0.05 for both. The data conformed to a normal distribution by Shapiro‐Wilk test. *p* Value was determined by a two‐tailed Student's *t*‐test. (C) Quantification of the relative protein level of PICK1 (*n* = 3) in the ICA1 overexpression group compared with that in the vector group in 20E2 cells, *p* > 0.05. The data conformed to a normal distribution by Shapiro‐Wilk test. *p* Value was determined by a one‐way ANOVA test. (D) Quantification of the relative protein level of PKCα (*n* = 3) after ICA1 overexpression and PKCα inhibition, *p < 0.05. (E) Quantification of the relative protein level of p‐PKCα (*n* = 3) after ICA1 overexpression and PKCα inhibition, **p < 0.01, ***p < 0.001. (F) Quantification of the relative protein level of C83 (*n* = 3) after ICA1 overexpression and PKCα inhibition, *p < 0.05, **p < 0.01, ***p < 0.001. (G) Quantification of the relative protein level of APP (*n* = 3) after ICA1 overexpression and PKCα inhibition, *p < 0.05, **p < 0.01, ***p < 0.001. (H) Quantification of the relative protein level of ADAM10 (*n* = 3) after ICA1 overexpression and PKCα inhibition, *p < 0.05, ***p < 0.001. (I) Quantification of the relative protein level of ADAM17 (*n* = 3) after ICA1 overexpression and PKCα inhibition, *p < 0.05, **p < 0.01.The data for each group conformed to a normal distribution by Shapiro‐Wilk test. *p* Value was determined by a one‐way ANOVA test.

In previous transcriptome sequencing results, we found that ICA1 knockdown affected the PKC signaling pathway. It has been shown that ICA1 indirectly binds to PKCα after binding to PICK1. We successfully overexpressed ICA1 (*n* = 3, *p* < 0.05, Figure 6B) and measured the expression of PICK1, PKCα, and phosphorylated PKCα (p‐PKCα) in 20E2. Overexpression of ICA1 did not affect PICK1 protein level (*n* = 3, *p* > 0.05, Figure 6A,C), but significantly increased PKCα protein level (*n* = 3, *p* < 0.05, Figure 6A,D) and promoted PKCα phosphorylation (*n* = 3, *p* < 0.01, Figure 6A,E). Next, we inhibited PKC using Go 6983 in 20E2 cells that overexpressed ICA1. We found that Go 6983 decreased the protein levels of PKCα (*n* = 3, *p* < 0.05, Figure 6A,D), p‐PKCα (*n* = 3, *p* < 0.001, Figure 6A,E), APP (*n* = 3, *p* < 0.05, Figure 6A,G), ADAM10 (*n* = 3, *p* < 0.05, Figure 6A,H), ADAM17 (*n* = 3, *p* < 0.05, Figure 6A,1), and C83 (*n* = 3, *p* < 0.05, Figure 6A,F), and the increased levels of PKCα (*n* = 3, *p* < 0.05, Figure 6A,D), p‐PKCα (*n* = 3, *p* < 0.001, Figure 6A,E), APP (*n* = 3, *p* < 0.001, Figure 6A,G), ADAM10 (*n* = 3, *p* < 0.001, Figure 6A,H), ADAM17 (*n* = 3, *p* < 0.01, Figure 6A,I) and C83 (*n* = 3, *p* < 0.001, Figure 6A,F) induced by overexpression of ICA1 were rescued. To further determine whether ICA1 affects the PICK1‐PKCα signaling pathway, we treated 2 EB2 cells (File S1, Figure S1) with GO 6983 and 20E2 cells with the PKCα‐specific inhibitor Aprinocarsen (File S1, Figure S3), and we obtained similar results. These data indicated that ICA1 affects APP processing through the PICK1‐PKCα signaling pathway.

## 4 DISCUSSION

ICA1 is recognized as an autoimmune antigen with high expression in the brain, suggesting it has an important function in AD. Our research found that ICA1 expression was notably diminished in the brains of patients with AD and in an AD mouse model. We also found that overexpression of ICA1 increased the expression of APP, ADAM10, ADAM17, and CTFs, while knockdown of ICA1 decreased their expression. In order to examine the influence of ICA1 on the expression of proteins related to APP processing, we assessed the protein half‐life and mRNA levels of APP, ADAM10, and ADAM17 in ICA1‐overexpressing 20E2 cells. Our study demonstrated that ICA1 overexpression did not affect the protein half‐life of APP, ADAM10, or ADAM17, or their mRNA levels. Transcriptome sequencing was performed to explore the specific mechanisms by which ICA1 affects APP processing. Our transcriptome sequencing results further suggested that ICA1 affects the PKC signaling pathway.

PKCα, a subtype of G protein‐coupled receptor, is a family of phospholipid‐dependent serine/threonine kinases with either a canonical or dual binding mode with PICK1.^34^ PKC activation in neurons increases the Ser880 phosphorylation of the GluR2 subunit and recruits PICK1 to excitatory synapses. PKC stimulation in neurons results in the rapid internalization of surface GluR2 subunits. Therefore, PKC modulates the surface expression of AMPA receptors during synaptic plasticity.^35^ It has been shown that ICA1 regulates the trafficking of the PKCα‐PICK1 complex to the plasma membrane.^15^, ^28^ Many studies have shown that PKCα co‐translocates with ADAM10 to the cell membrane and modulates α‐secretase.^36^, ^37^ Moreover, PKC facilitates the clearance of Aβ. PKC binds to the activated TFEB transcription factor by inactivation of the ZKSCAN3 transcription repressor through two parallel signaling cascades. Activated PKC inactivates GSK3β, resulting in reduced phosphorylation, nuclear translocation, and TFEB activation, while PKC activates JNK and p38 MAPK and phosphorylates ZKSCAN3, resulting in its inactivation via extranuclear translocation, consequently alleviating transcriptional repression, regulating lysosome biogenesis, and ameliorating Aβ plaque formation in the brain of APP/PS1 mice.^38^

The PKC signaling pathway is involved in α‐secretase and Aβ clearance. Our findings revealed that ICA1 regulated the PICK1‐PKCα signaling pathway, thus increasing α‐secretase and affecting APP processing. Our transcriptome sequencing data revealed that in ICA1‐knockdown 20E2 cells, peroxisome proliferator‐activated receptor gamma (PPARγ) expression was notably decreased. PPARγ is a ligand‐activated nuclear receptor that acts in a coupled metabolic cycle with liver X receptors (LXRs).^39^ PPARγ upregulates the expression of the scavenger receptor CD36 and stimulates microglia to increase Aβ phagocytosis.^40^ Therefore, ICA1 may promote the clearance of Aβ. Moreover, the PKC signaling pathway activates ERK/MAPK^38^ and increases APP expression.^41^ The increased expression of APP after overexpression of ICA1 may be the important reason for the increased levels of C89 and C99 in 2 EB2.

In previous studies, ICA1 indirectly binds to PKCα and affects its transport by binding to PICK1.^28^ Our research conclusively showed the impact of ICA1 on the development of AD and its promising role in the treatment of AD. Our study reports that ICA1 regulates the expression and phosphorylation of PKCα, but the specific mechanism remains unclear. Next, we will explore the effect of ICA1 on the expression and phosphorylation of PKCα and verify it in mice.

In conclusion, we found that ICA1 modifies APP processing and redirects it towards non‐amyloid pathways by regulating the PICK1‐PKCα signaling pathway. These discoveries offer fresh perspectives on the function of ICA1 in the development of AD and its promise as a novel therapeutic target for AD.

## AUTHOR CONTRIBUTIONS

LJ, ZM, WZ, and WS conceived and designed this research. LJ, ZM, XD, QW, YJ, JZ, DH, and SG conducted the experiments. WZ and WS contributed reagents, materials, and analytical tools. WS and WZ supervised the project. LJ, WZ, and WS wrote the manuscript and revised the manuscript. All authors reviewed and approved the manuscript.

## FUNDING INFORMATION

No funding supported.

## CONFLICT OF INTEREST STATEMENT

The authors declare no competing interests.

## Supporting information


Figures S1–S3.

File S1.

File S2.

Table S1.

## Data Availability

Described in Results and Methods section and available upon request. The RNA‐seq datasets analyzed in this study are available in the Genome sequence archive database (Accession: HRA006173).
