# Supplementary material for: ICA1 affects APP processing through the PICK1‐PKCα signaling pathway
Source: CNS Neurosci Ther. 2024 Jun 17;30(6):e14754. doi: 10.1111/cns.14754 (PMC11181291; doi:10.1111/cns.14754)
Supplement: Supplementary file 1 — Figures S1–S3. [file CNS-30-e14754-s001.docx]

Supplemental information for

**ICA1 affects APP processing through the PICK1-PKCα signaling pathway**

**Liangye Ji^1^, ZiJun Meng^1,*^, Xiangjun Dong^1^, Qunxian Wang^1^, Yanshuang Jiang^1^, Jie Zhang^1^, Dongjie Hu^1^, Shipeng Guo^1^, Weihui Zhou^1,*^ and Weihong Song^1,2,3*^**

**Methods**

**ELISA**

Human Aβ1-40 (Amyloid Beta 1-40) ELISA Kit (E-EL-H0542, Elabscience) and Human Aβ1-42 (Amyloid Beta 1-42) ELISA Kit (E-EL-H0543, Elabscience) were respectively used to detect Aβ40 and Aβ42, according to the protocol.

**Figures(Fig. S1 to S3)**

**
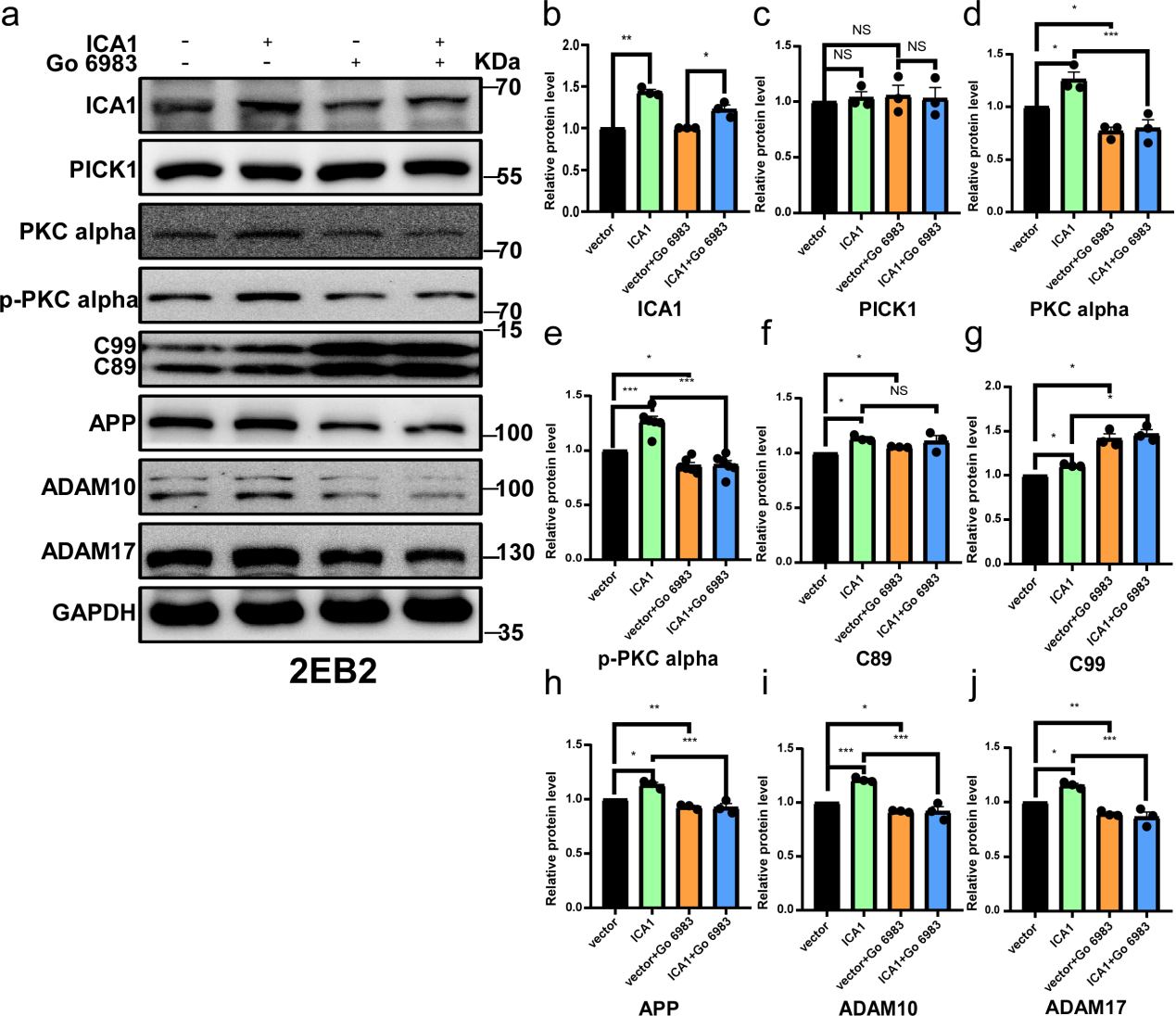
Supplemental Figure1 ICA1 affects APP processing through PICK1-PKCα signaling pathway in 2EB2.**

(a) Western blotting showed that ICA1 affects APP processing via the PICK1-PKCα signaling pathway. (b) Quantification of the relative protein level of ICA1(n=3) in the ICA1 overexpression group compared to the vector group in 20E2, *p<0.05, **p<0.01. The data conformed to a normal distribution. p Value was determined by two-tailed Student's t-test. (c) Quantification of the relative protein level of PICK1(n=3) in the ICA1 overexpression group compared with that in the vector group in 20E2 cells, p>0.05. The data conformed to a normal distribution. p Value was determined by two-tailed Student's t-test. (d) Quantification of the relative protein level of PKCα(n=3) after ICA1 overexpression and PKCα inhibition, *p<0.05, ***p<0.001. (e) Quantification of the relative protein level of p-PKCα(n=6) after ICA1 overexpression and PKCα inhibition, *p<0.05, ***p<0.001. (f) Quantification of the relative protein level of C83(n=3) after ICA1 overexpression and PKCα inhibition, *p<0.05. (g) Quantification of the relative protein level of C99(n=3) after ICA1 overexpression and PKCα inhibition, *p<0.05. (h) Quantification of the relative protein level of APP(n=3) after ICA1 overexpression and PKCα inhibition, *p<0.05, **p<0.01, ***p<0.001. (i) Quantification of the relative protein level of ADAM10(n=3) after ICA1 overexpression and PKCα inhibition, *p<0.05, ***p<0.001. (j) Quantification of the relative protein level of ADAM17(n=3) after ICA1 overexpression and PKCα inhibition, *p<0.05, **p<0.01, ***p<0.001.The data for each group conformed to a normal distribution by Shapiro-Wilk test. p Value was determined by one-way ANOVA test. The 2EB2 cell line is HEK 293 cells stably transfected human APP695 with Swedish mutation and BACE1.


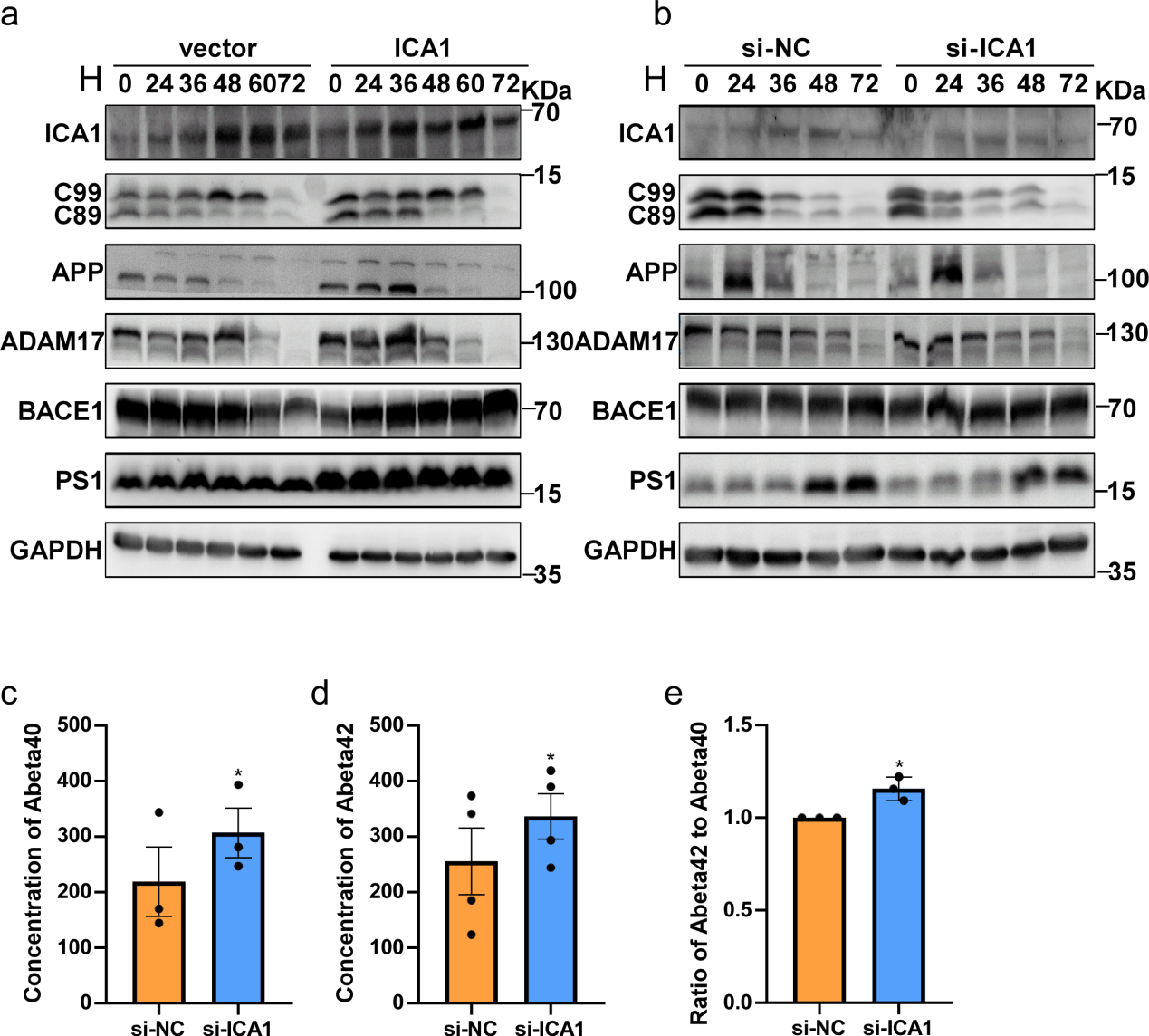


**Supplemental Figure2 Overexpression or knockdown ICA1 in 2EB2.**

(a) Different time points after transfection of ICA1 plasmid. (b) Different time points after transfection of si-RNA. (c) Concentration of Abeta40 in 2EB2 after knockdown ICA1. n=3, *p<0.05. (d) Concentration of Abeta42 in 2EB2 after knockdown ICA1. n=4, *p<0.05. (e) Relative ratio of Abeta42 to Abeta40. n=3, *p<0.05. The data for each group conformed to a normal distribution by Shapiro-Wilk test. p Value was determined by two-railed paired Student's-t test.


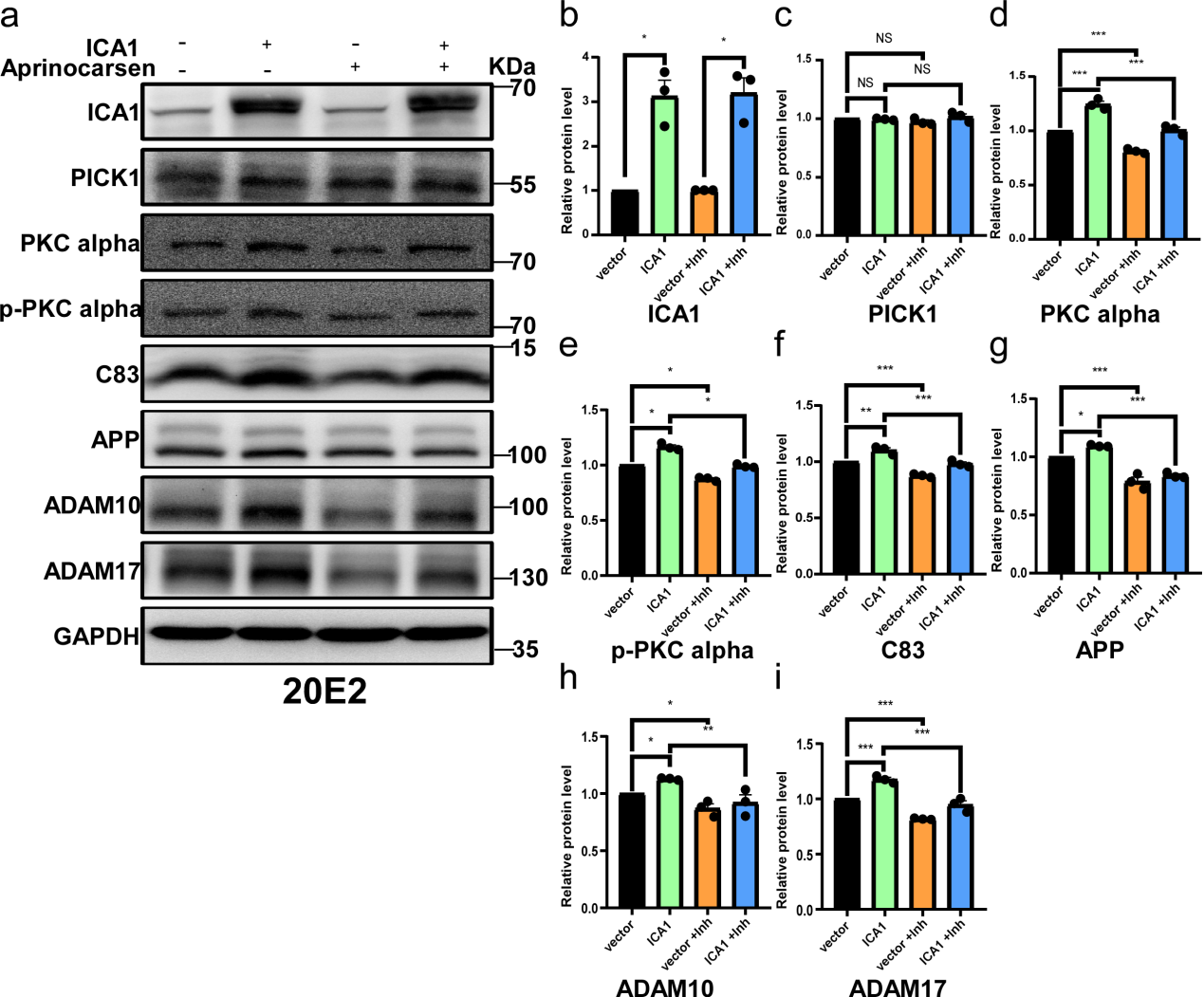


**Supplemental Figure3 ICA1 affects APP processing through PICK1-PKCα signaling pathway.**

(a) Western blotting showed that ICA1 affects APP processing via the PICK1-PKCα signaling pathway. (b) Quantification of the relative protein level of ICA1(n=3) in the ICA1 overexpression group compared to the vector group in 20E2, *p<0.05 for both. The data conformed to a normal distribution by Shapiro-Wilk test. p Value was determined by two-tailed Student's t-test. (c) Quantification of the relative protein level of PICK1(n=3) in the ICA1 overexpression group compared with that in the vector group in 20E2 cells, p>0.05. The data conformed to a normal distribution by Shapiro-Wilk test. p Value was determined by one-way ANOVA test. (d) Quantification of the relative protein level of PKCα(n=3) after ICA1 overexpression and PKCα inhibition, ***p<0.001. (e) Quantification of the relative protein level of p-PKCα(n=3) after ICA1 overexpression and PKCα inhibition, *p<0.05. (f) Quantification of the relative protein level of C83(n=3) after ICA1 overexpression and PKCα inhibition, **p<0.01, ***p<0.001. (g) Quantification of the relative protein level of APP(n=3) after ICA1 overexpression and PKCα inhibition, *p<0.05, ***p<0.001. (h) Quantification of the relative protein level of ADAM10(n=3) after ICA1 overexpression and PKCα inhibition, *p<0.05, **p<0.01. (i) Quantification of the relative protein level of ADAM17(n=3) after ICA1 overexpression and PKCα inhibition, ***p<0.001. The data for each group conformed to a normal distribution by Shapiro-Wilk test. p Value was determined by one-way ANOVA test. The 20E2 cell line is HEK 293 cells stably transfected human APP695 with Swedish mutation.
