# Supplementary material for: ICA1 affects APP processing through the PICK1‐PKCα signaling pathway
Source: CNS Neurosci Ther. 2024 Jun 17;30(6):e14754. doi: 10.1111/cns.14754 (PMC11181291; doi:10.1111/cns.14754)

Full unedited blot for Figure 1b Cortex ICA1

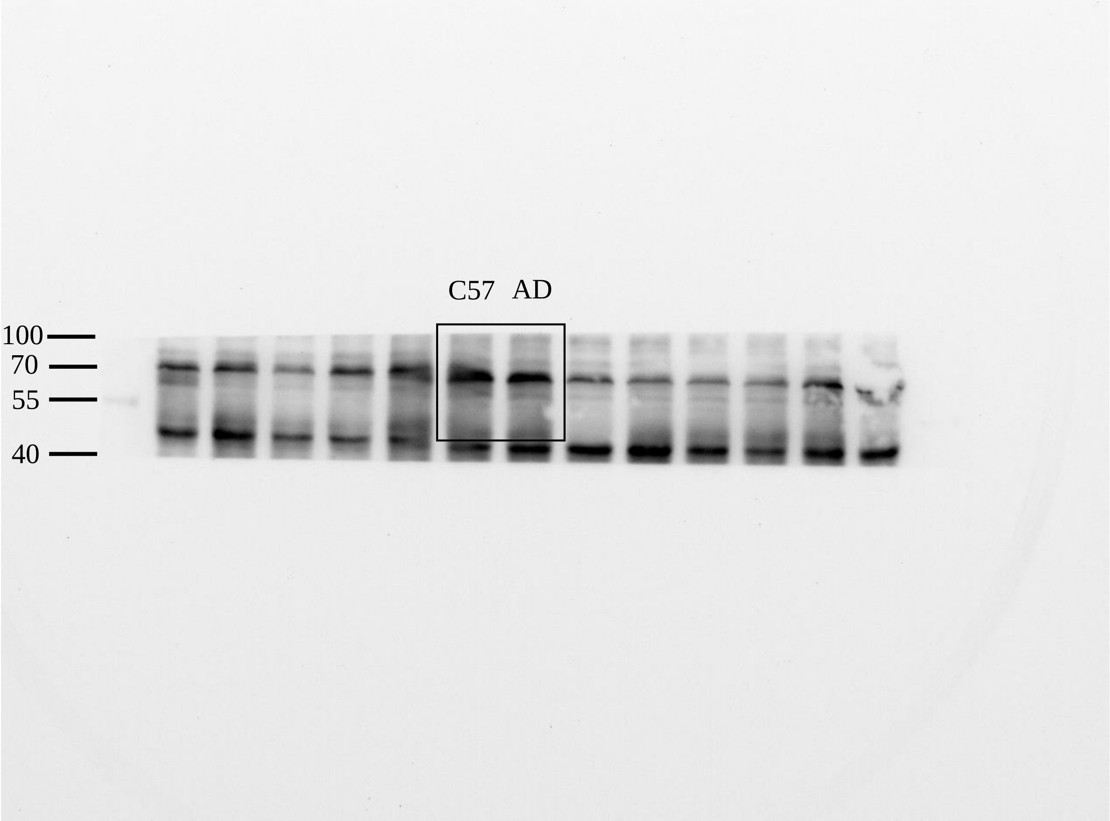

Full unedited blot for Figure 1b Cortex GAPDH

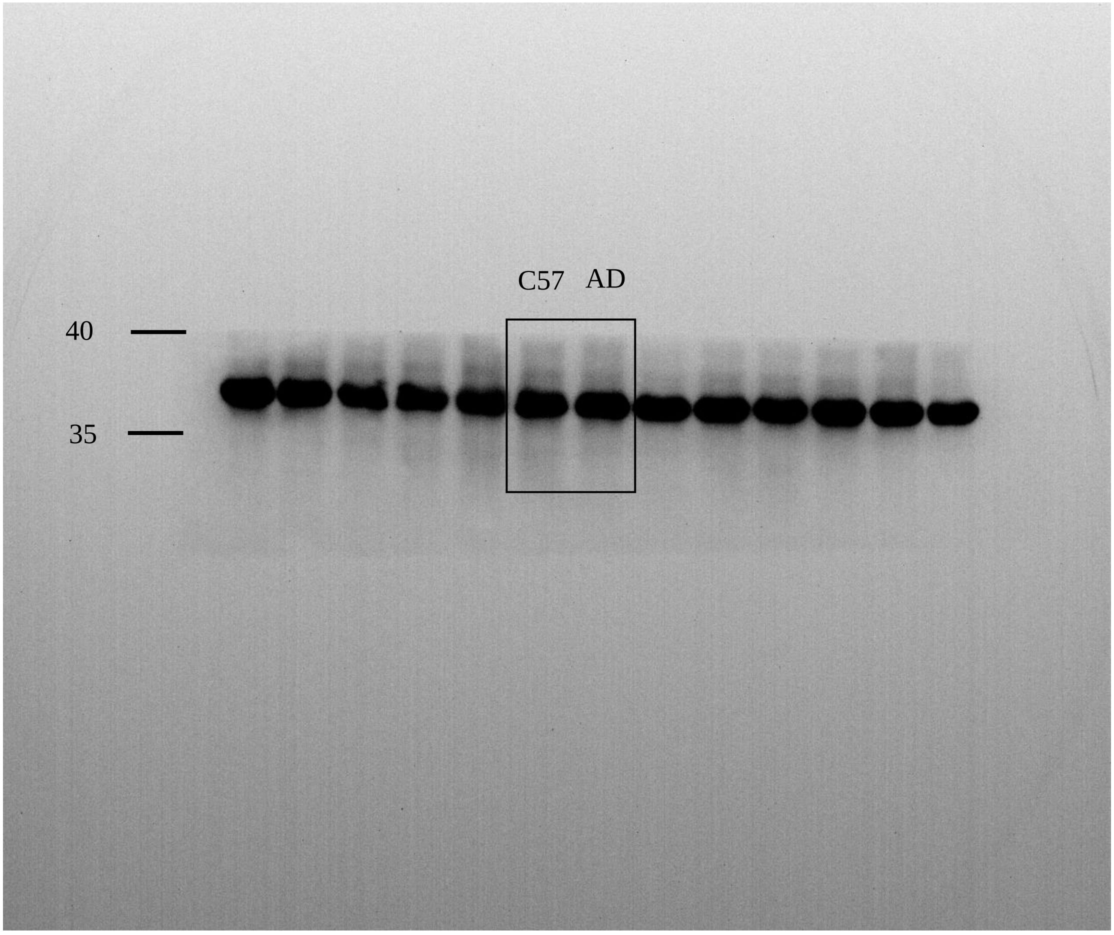

Full unedited blot for Figure 1b Hippocampus ICA1

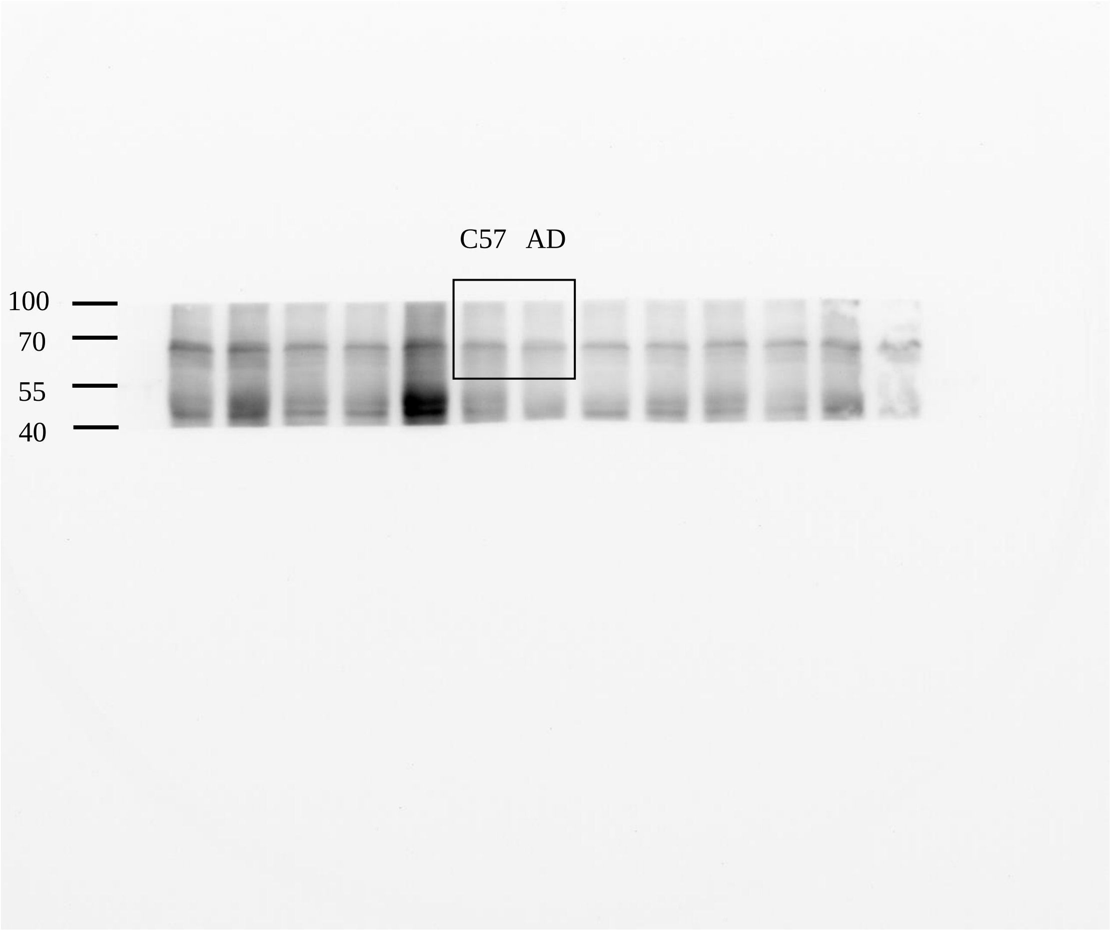

Full unedited blot for Figure 1b Hippocampus GAPDH

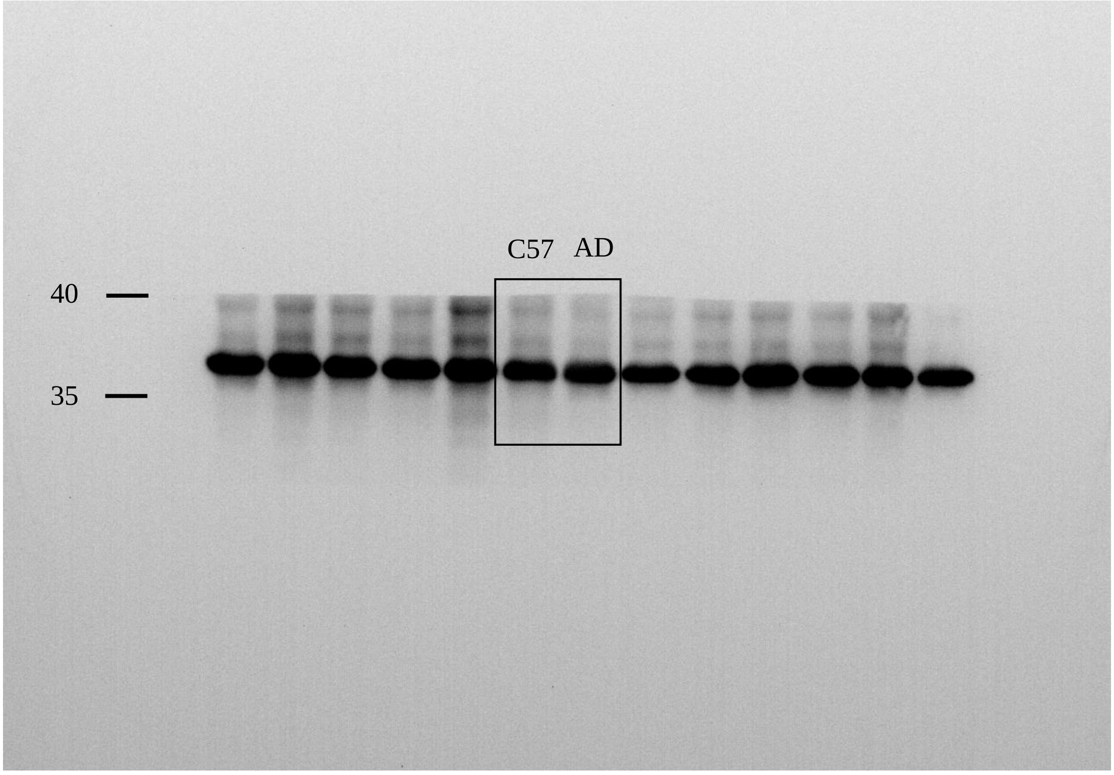

Full unedited blot for Figure 2a ICA1

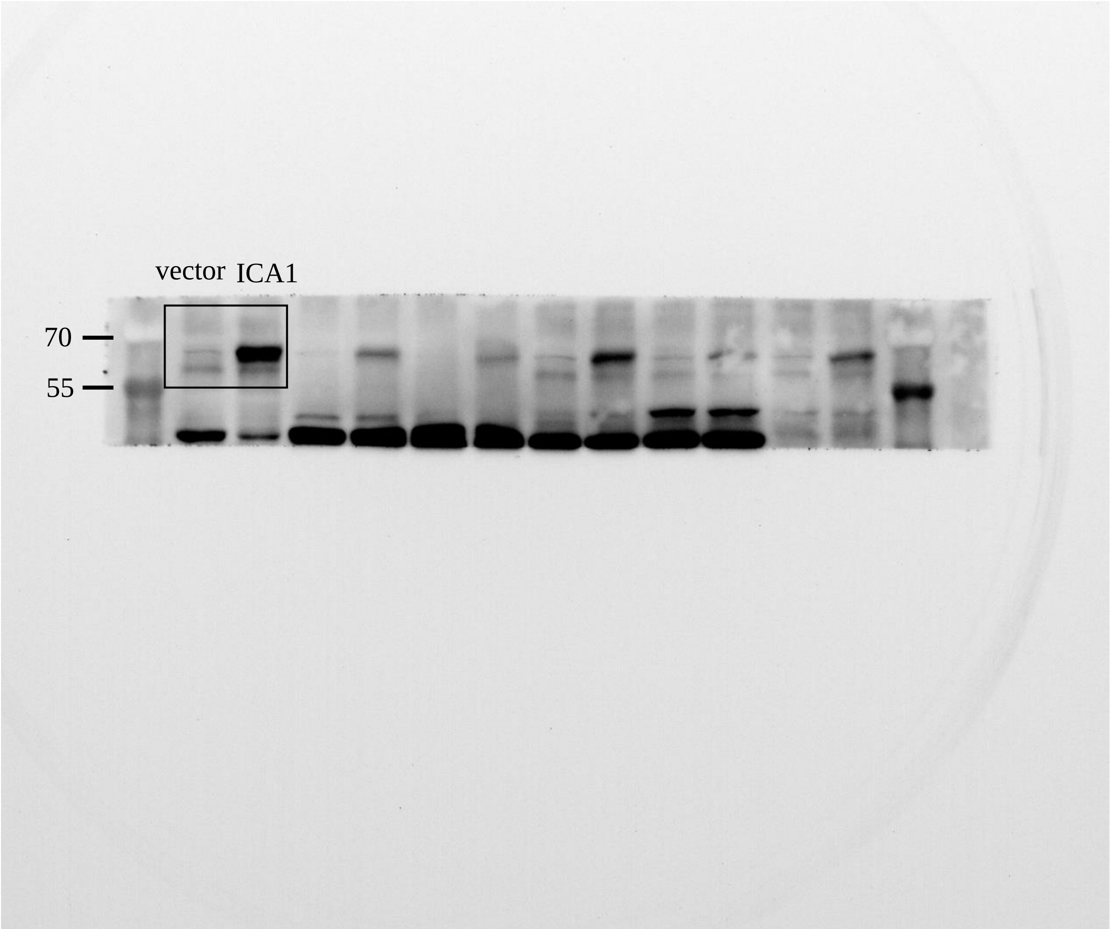

Full unedited blot for Figure 2a C89 and C99

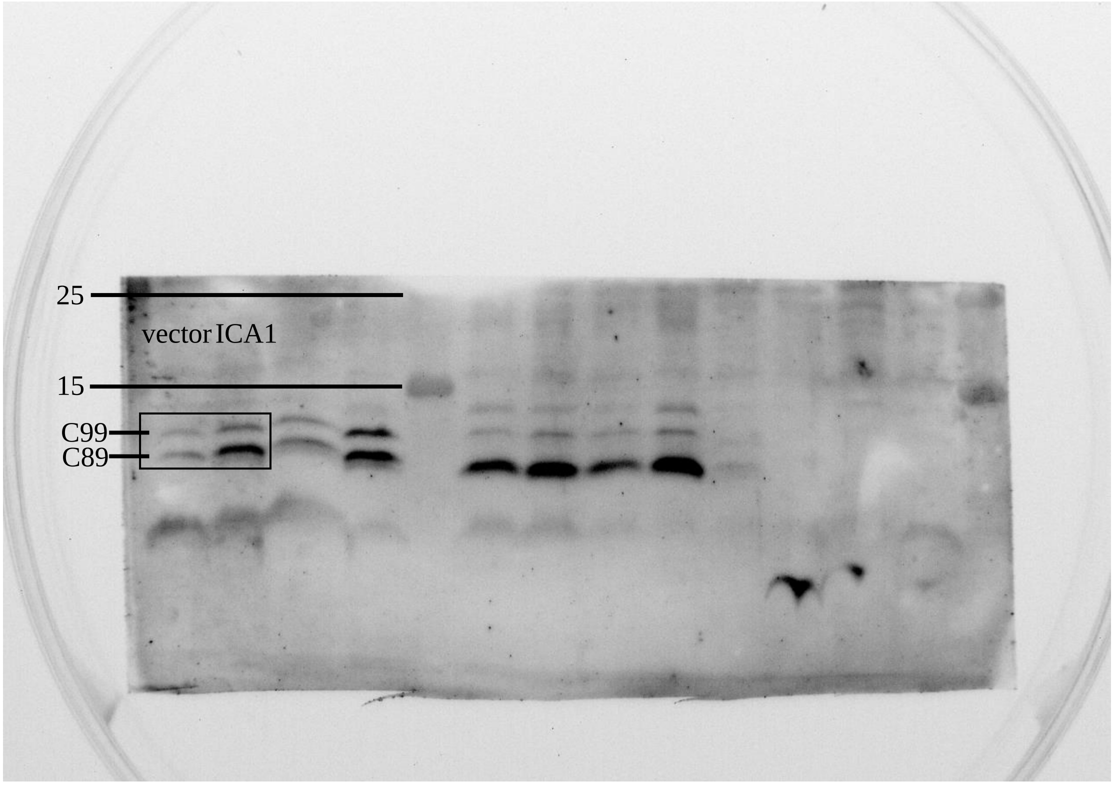

Full unedited blot for Figure 2a APP

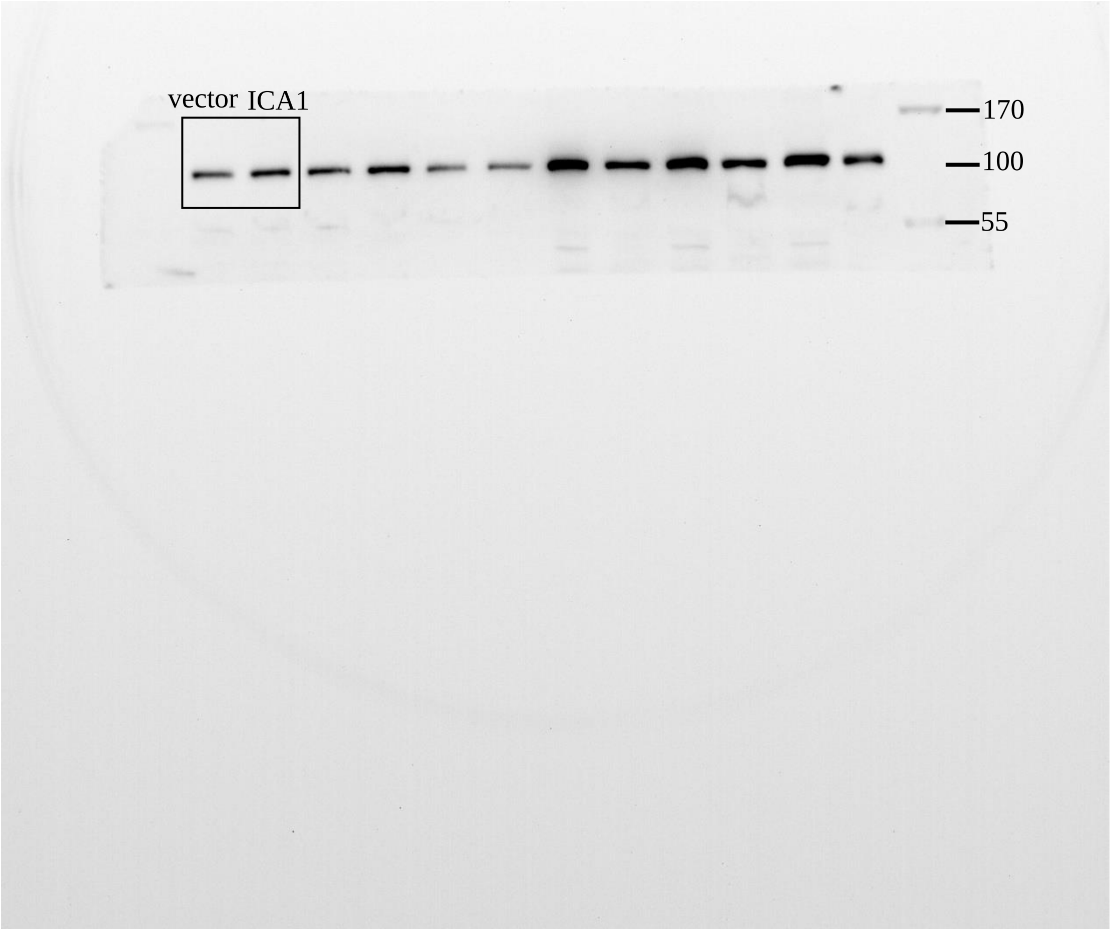

Full unedited blot for Figure 2a ADAM10

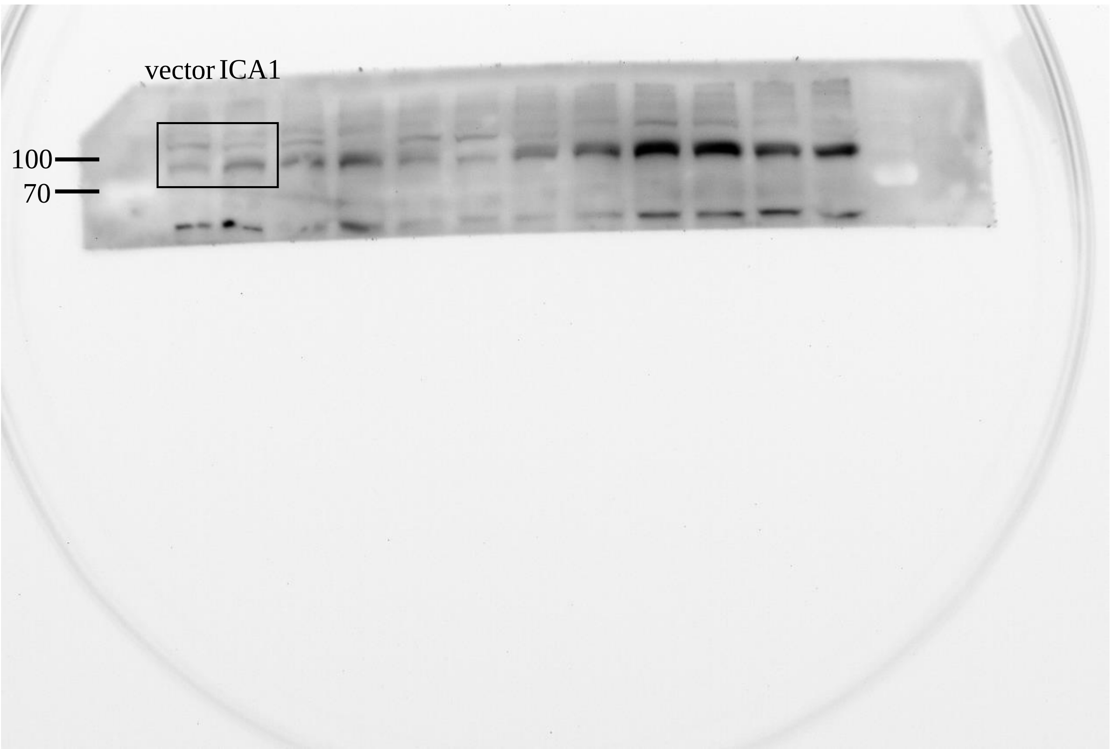

Full unedited blot for Figure 2a ADAM17

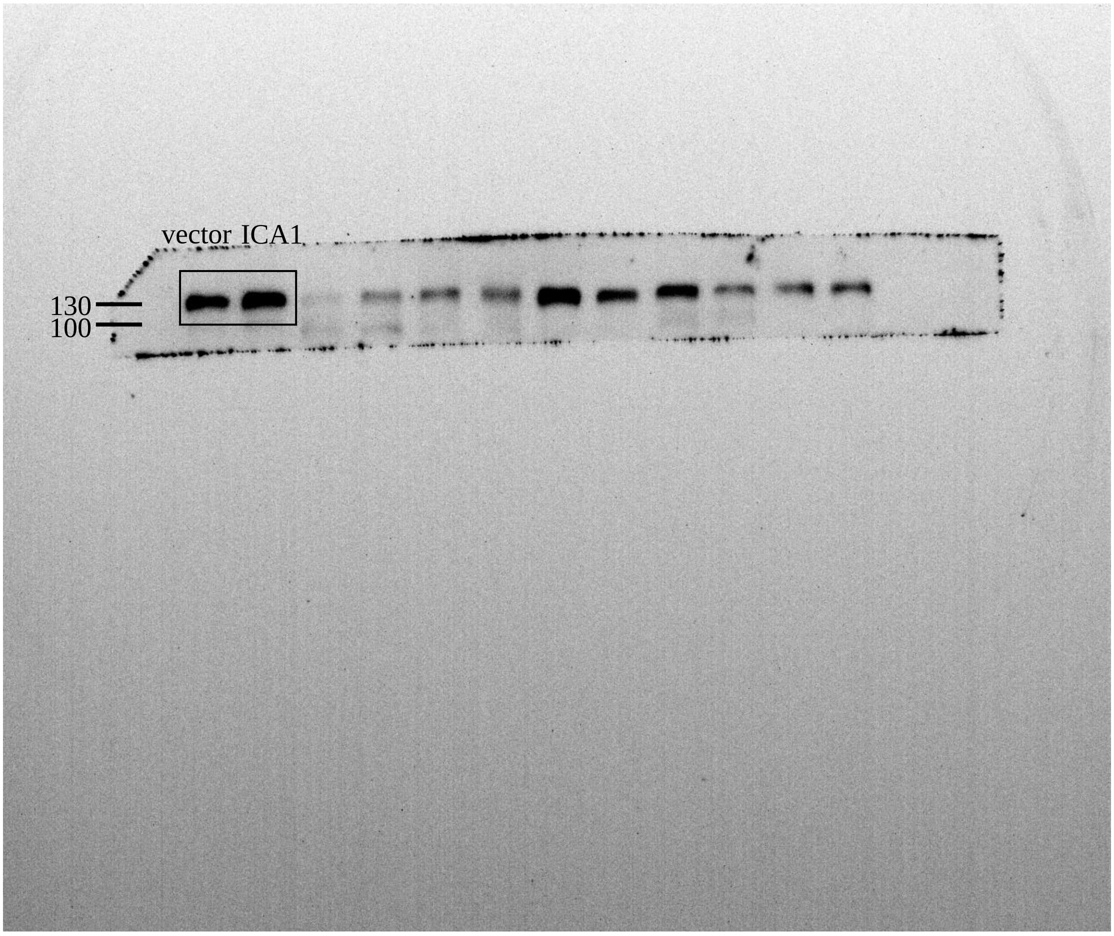

Full unedited blot for Figure 2a BACE1

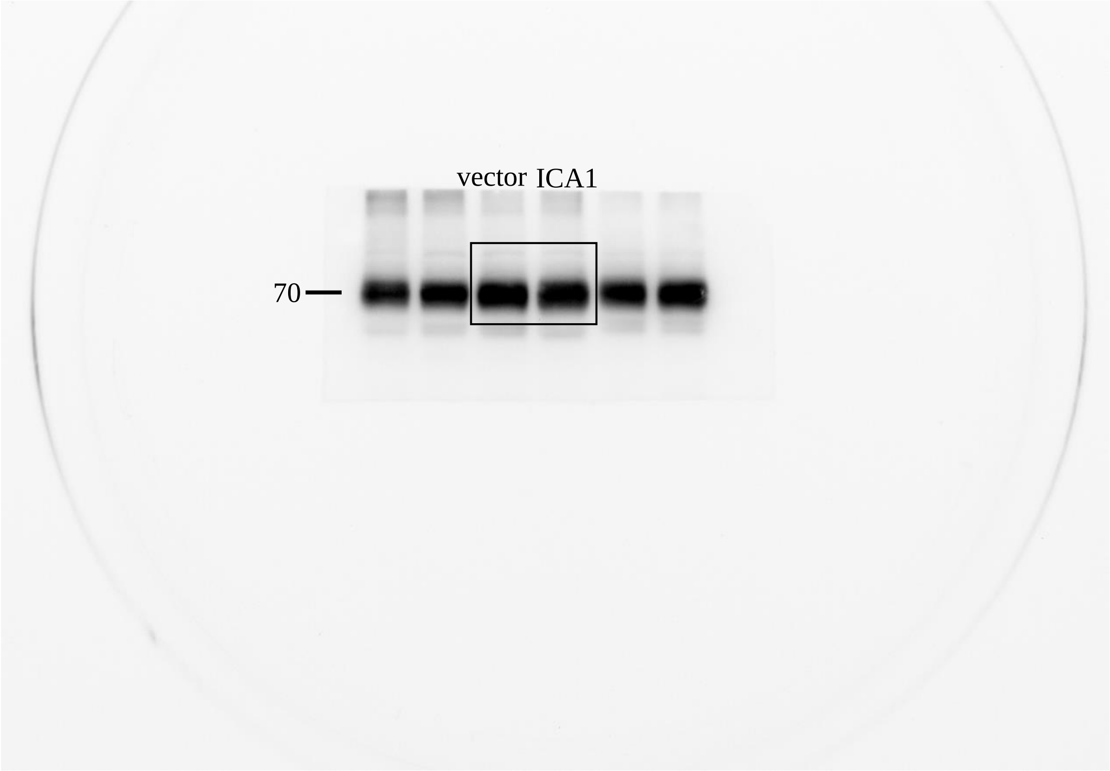

Full unedited blot for Figure 2a PS1

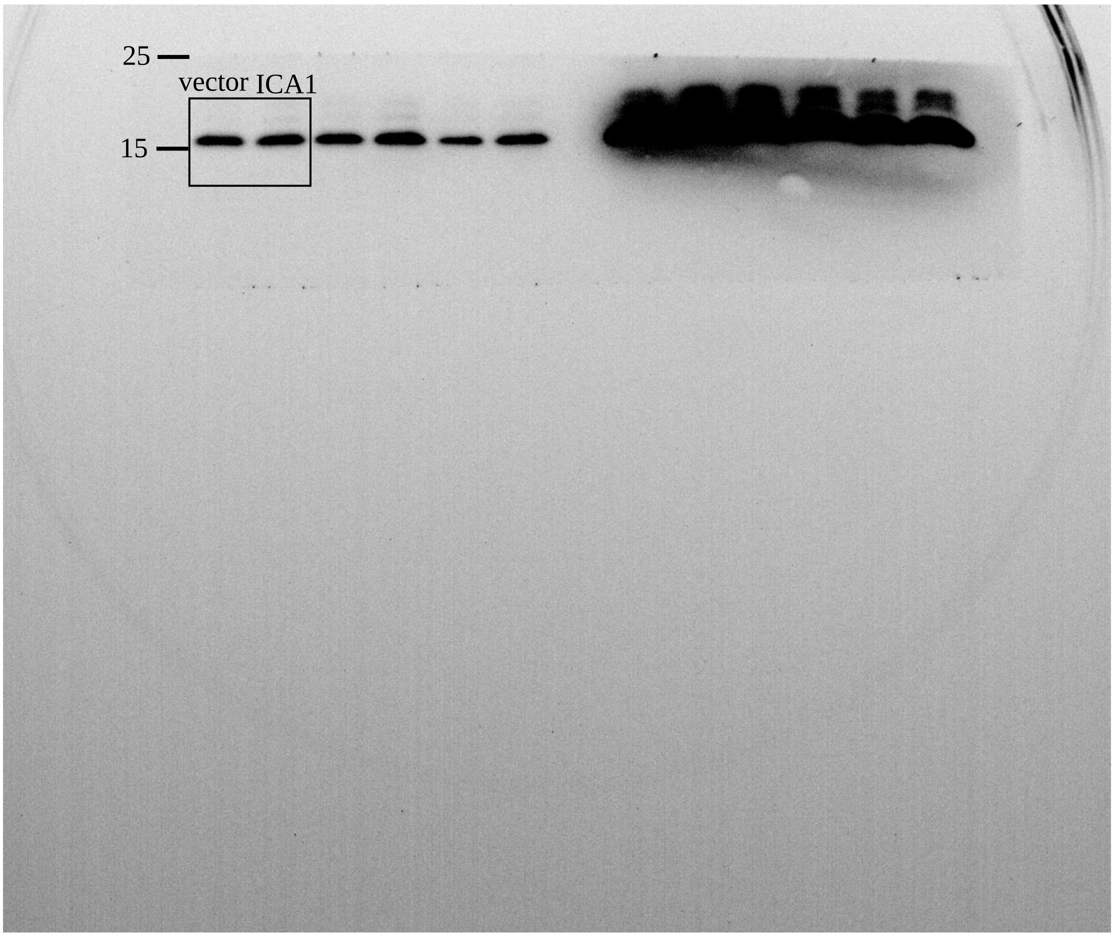

Full unedited blot for Figure 2a GAPDH

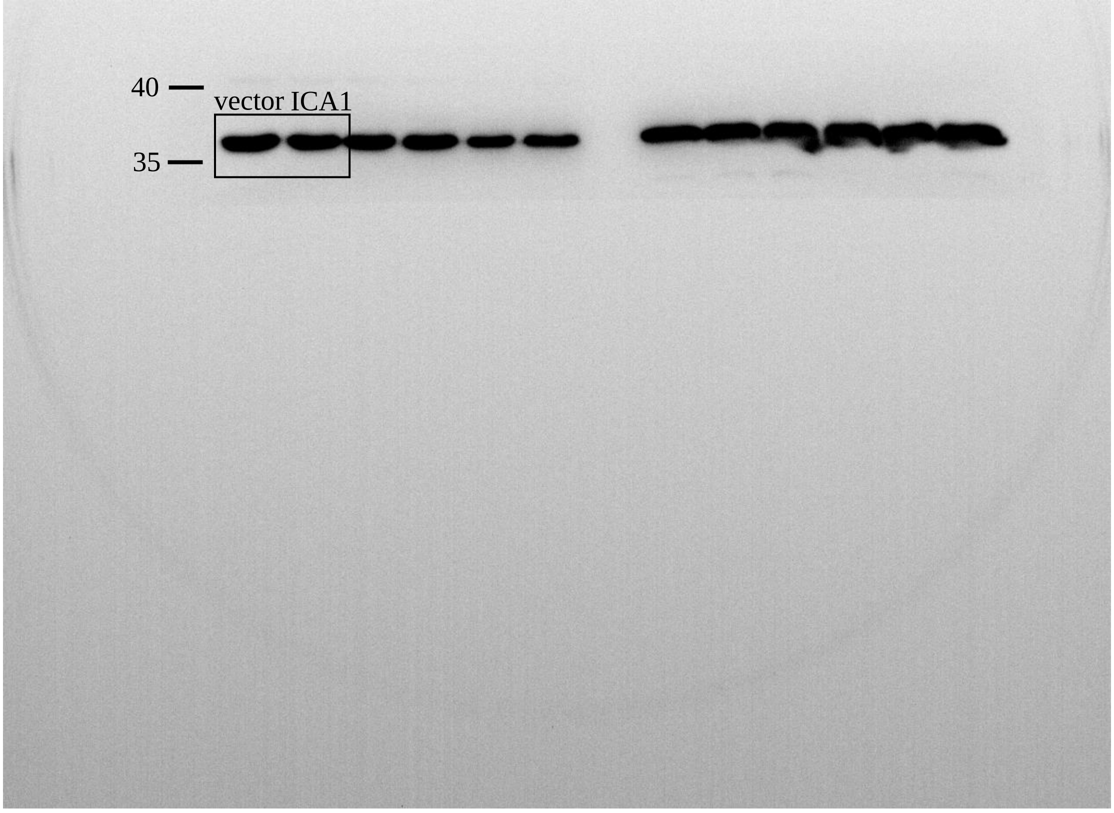

Full unedited blot for Figure 2b ICA1

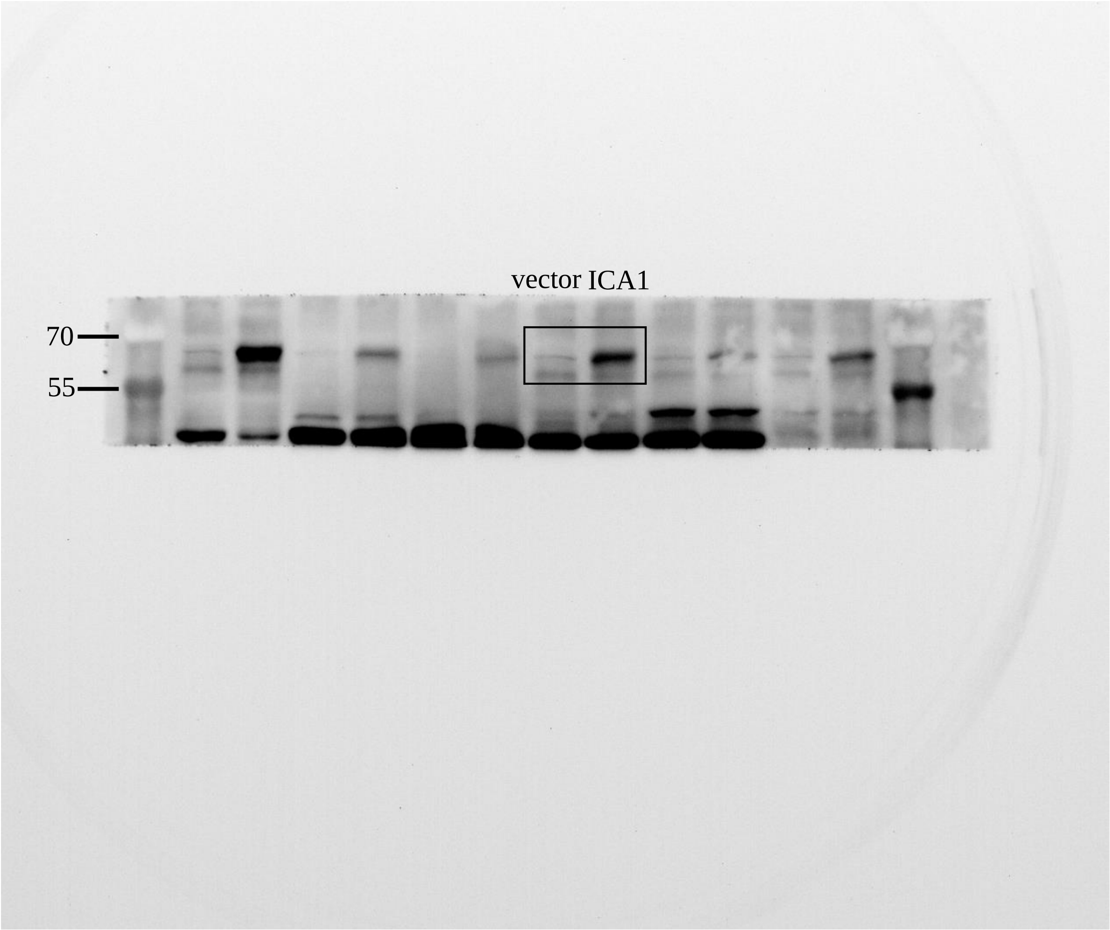

Full unedited blot for Figure 2b C83

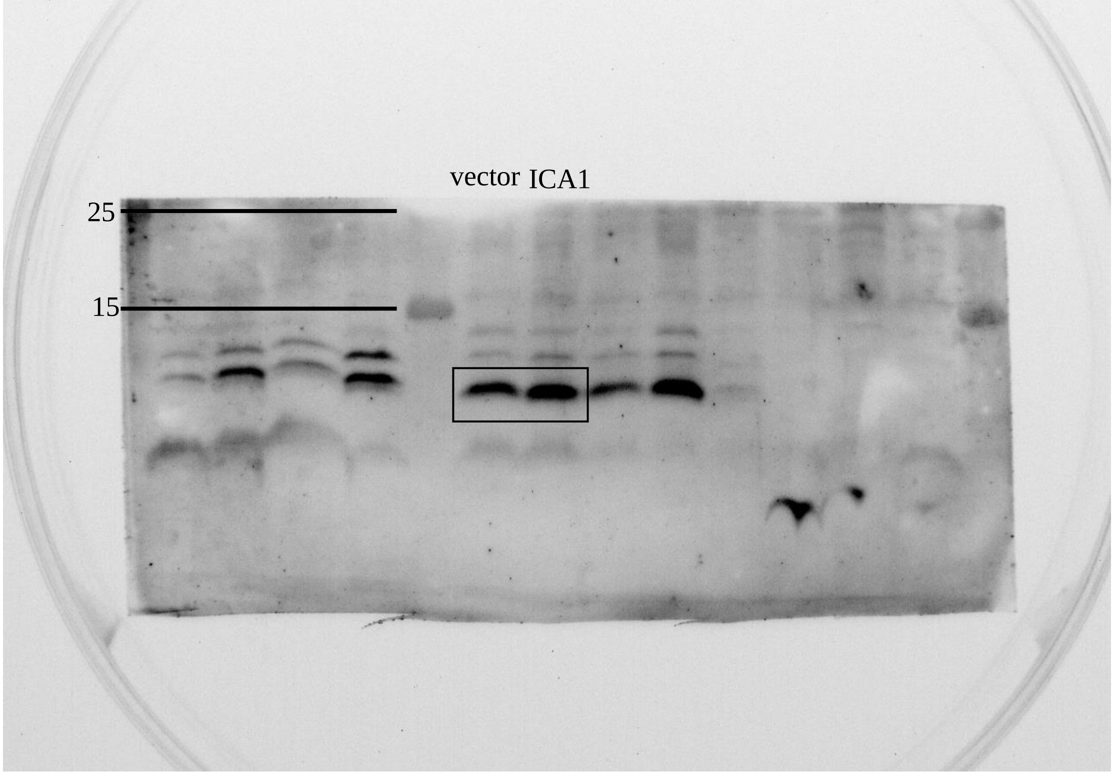

Full unedited blot for Figure 2b APP

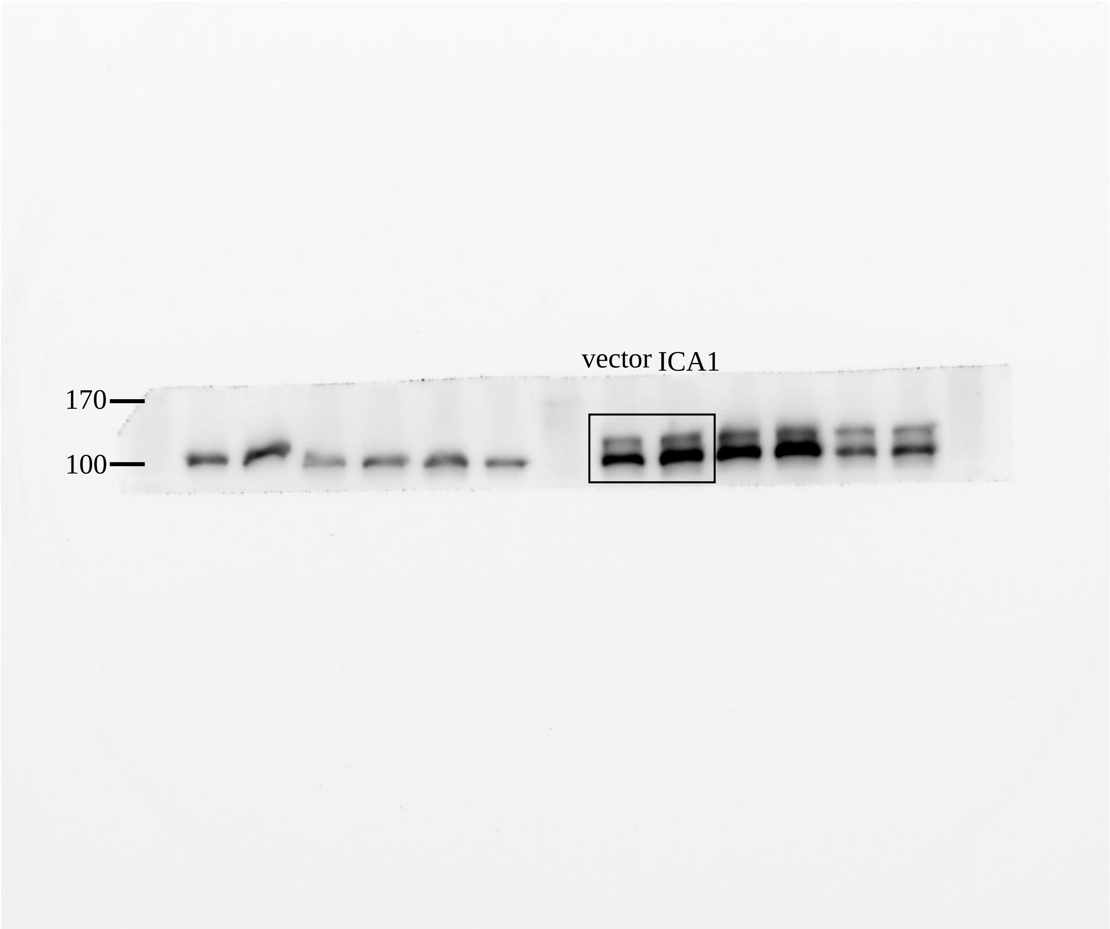

Full unedited blot for Figure 2b ADAM10

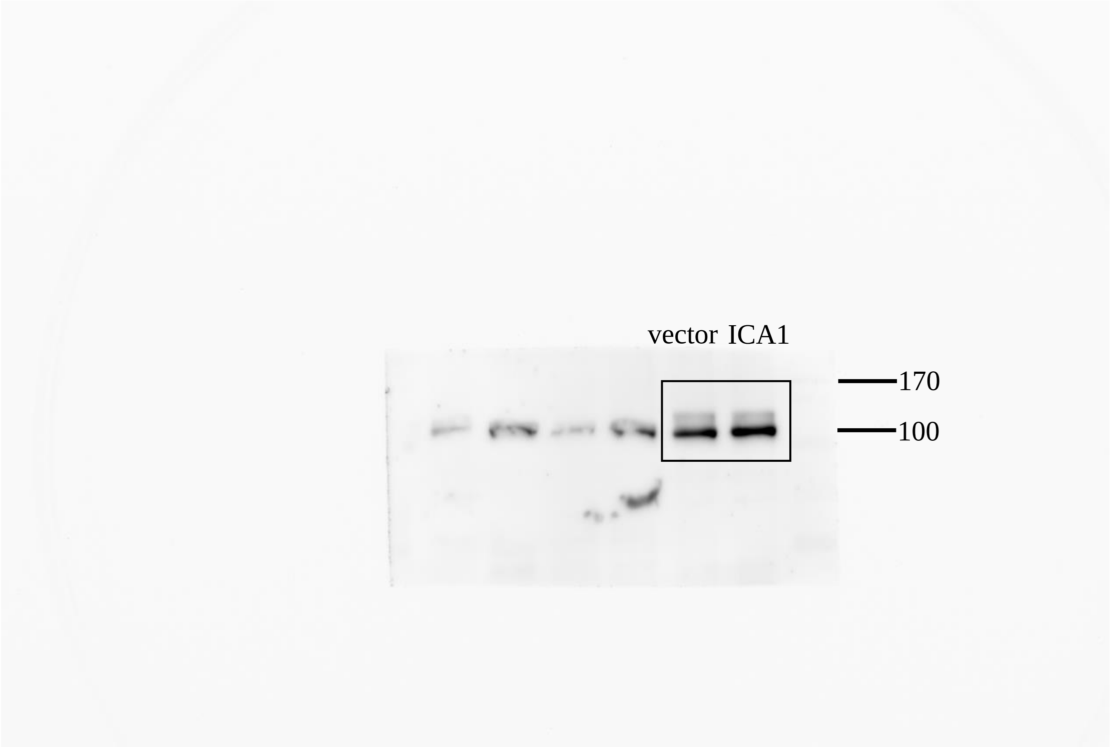

Full unedited blot for Figure 2b ADAM17

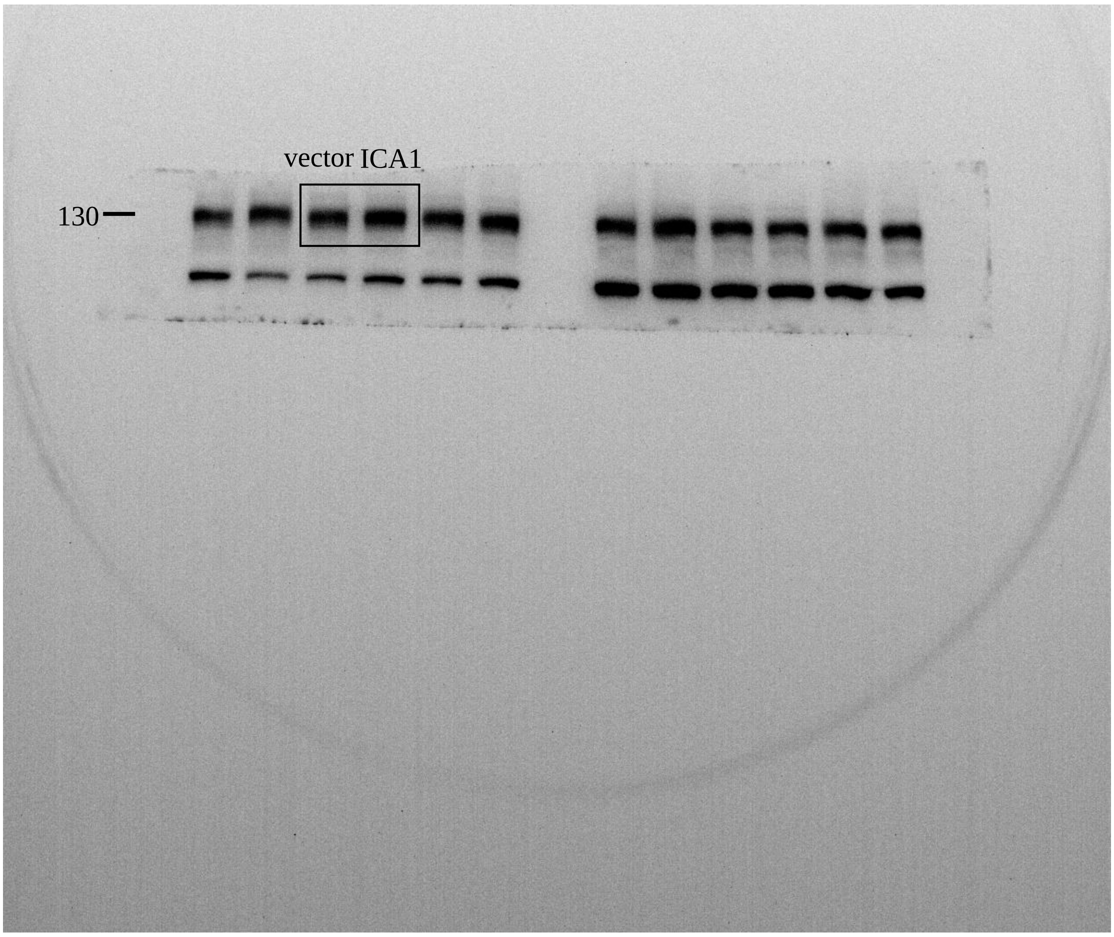

Full unedited blot for Figure 2b BACE1

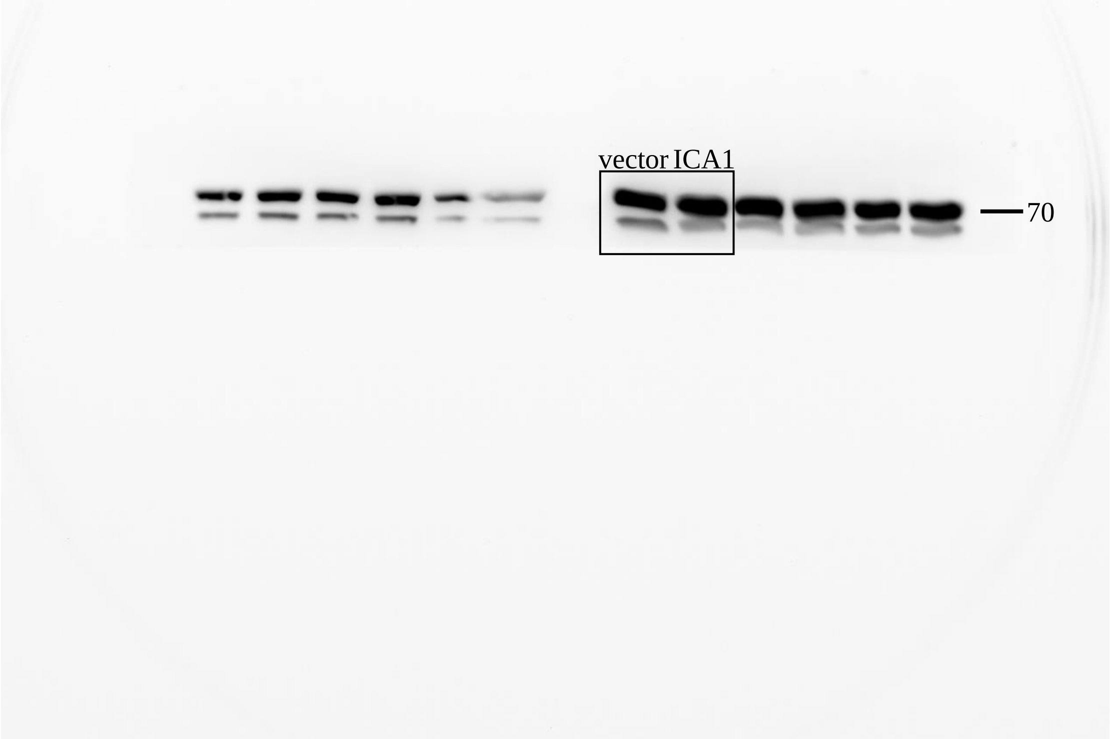

Full unedited blot for Figure 2b PS1

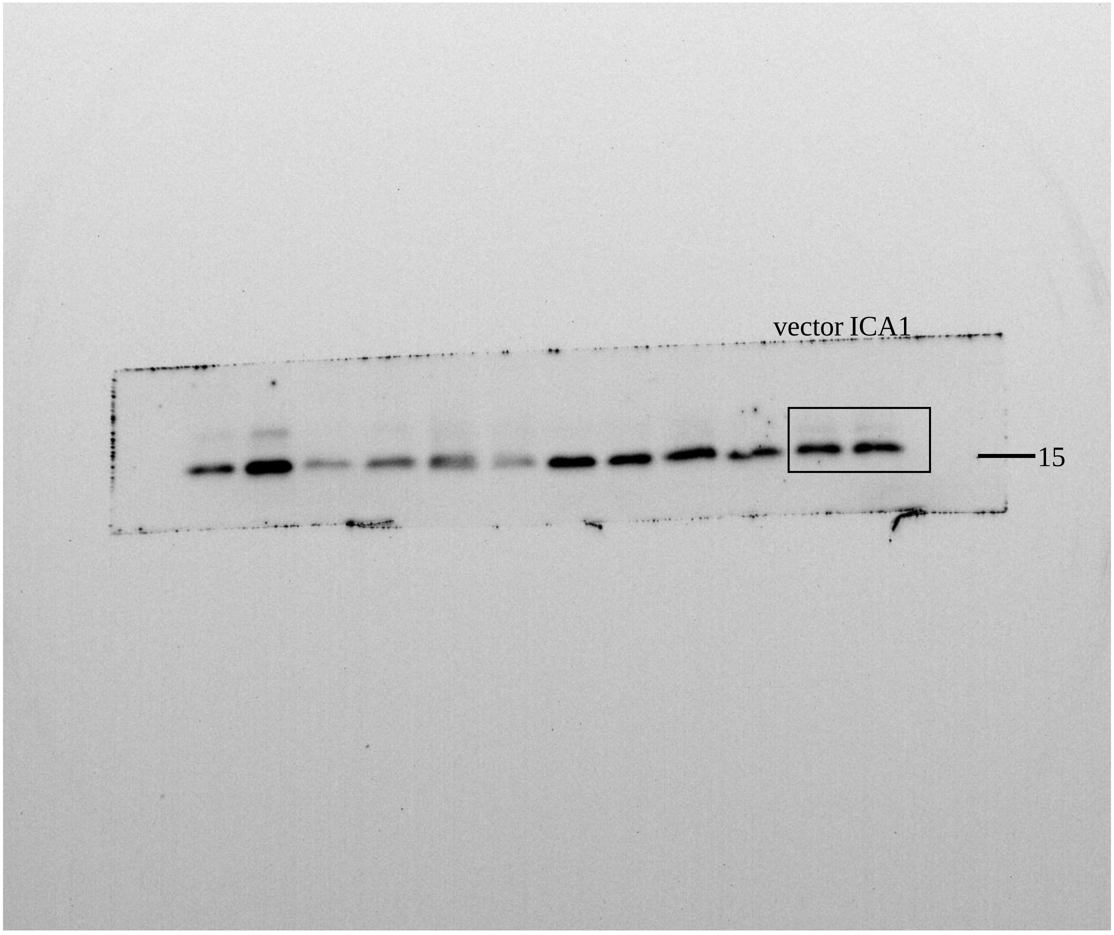

Full unedited blot for Figure 2b GAPDH

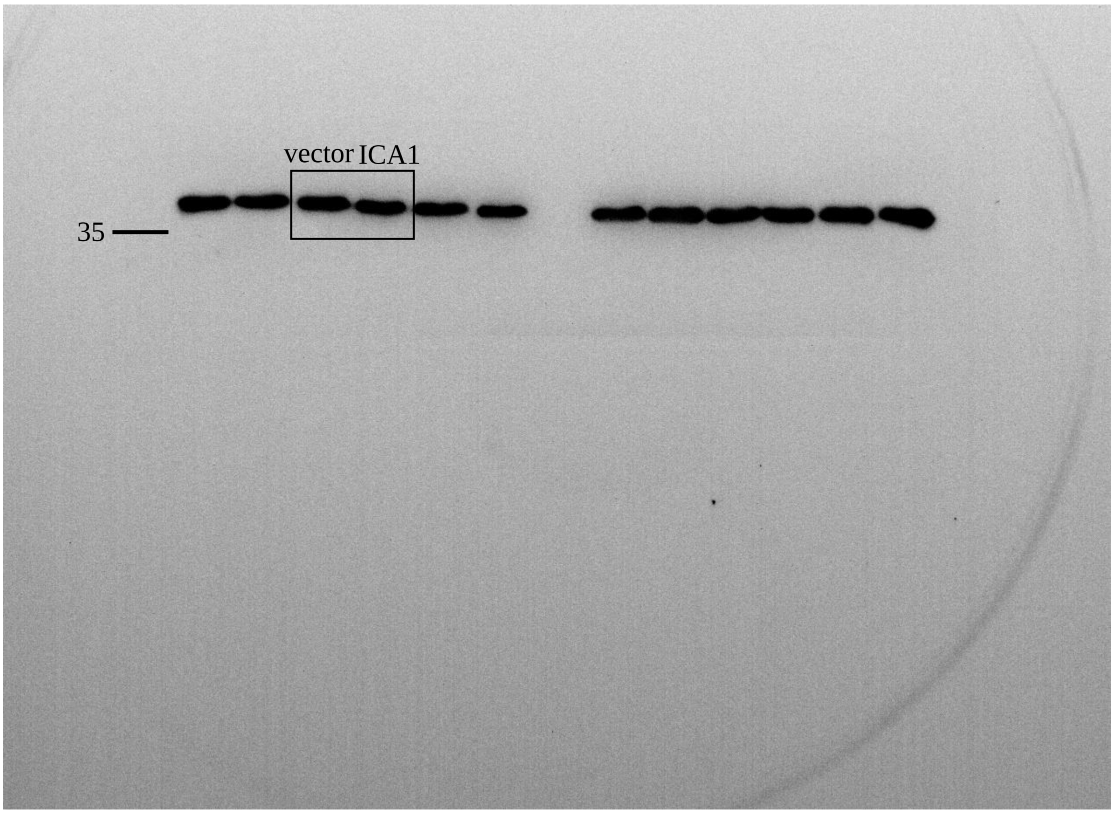

Full unedited blot for Figure 2c ICA1

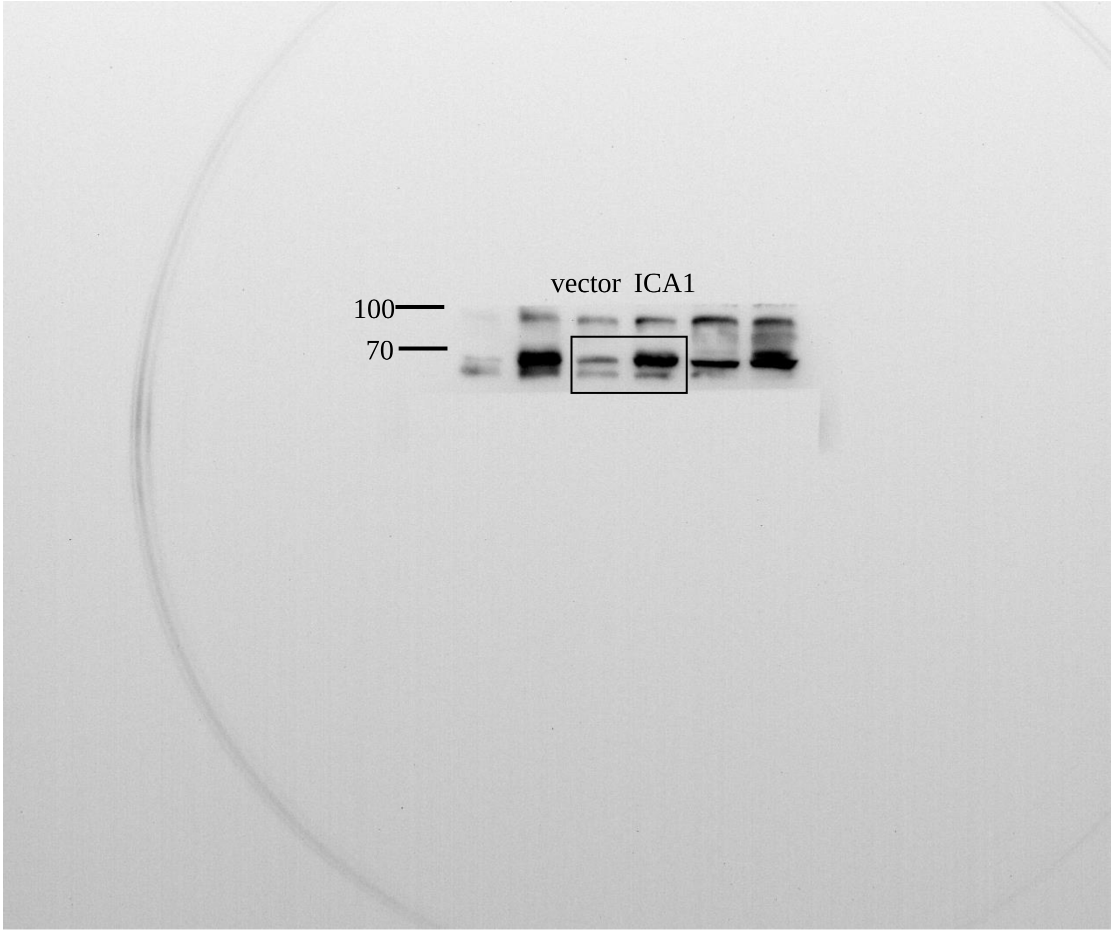

Full unedited blot for Figure 2c C83 and C99

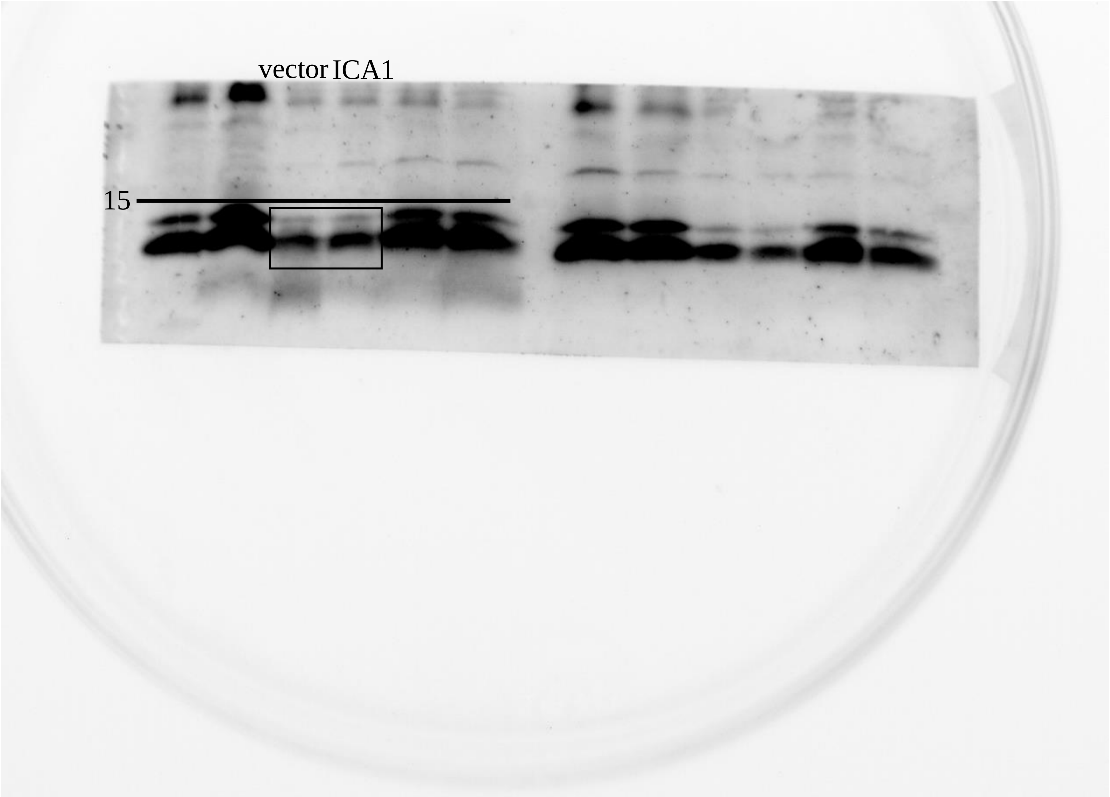

Full unedited blot for Figure 2c APP

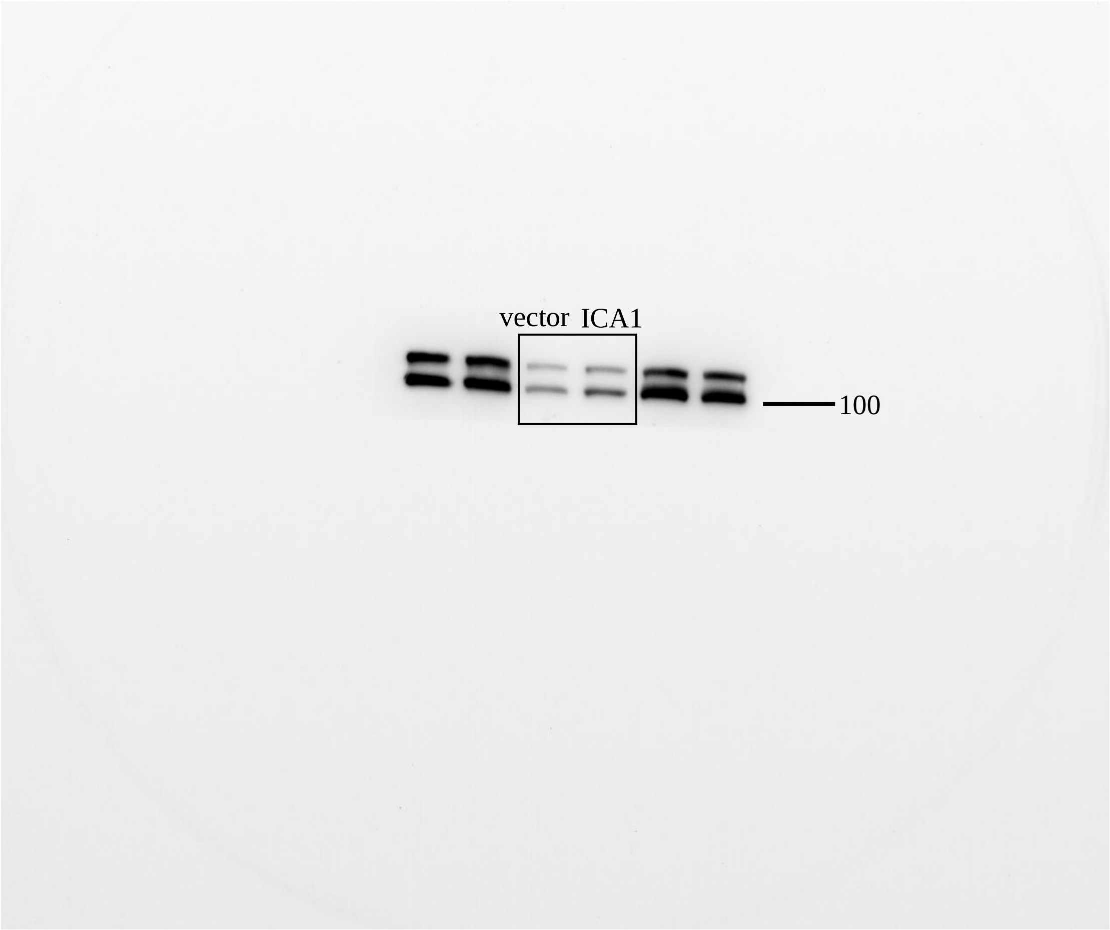

Full unedited blot for Figure 2c ADAM10

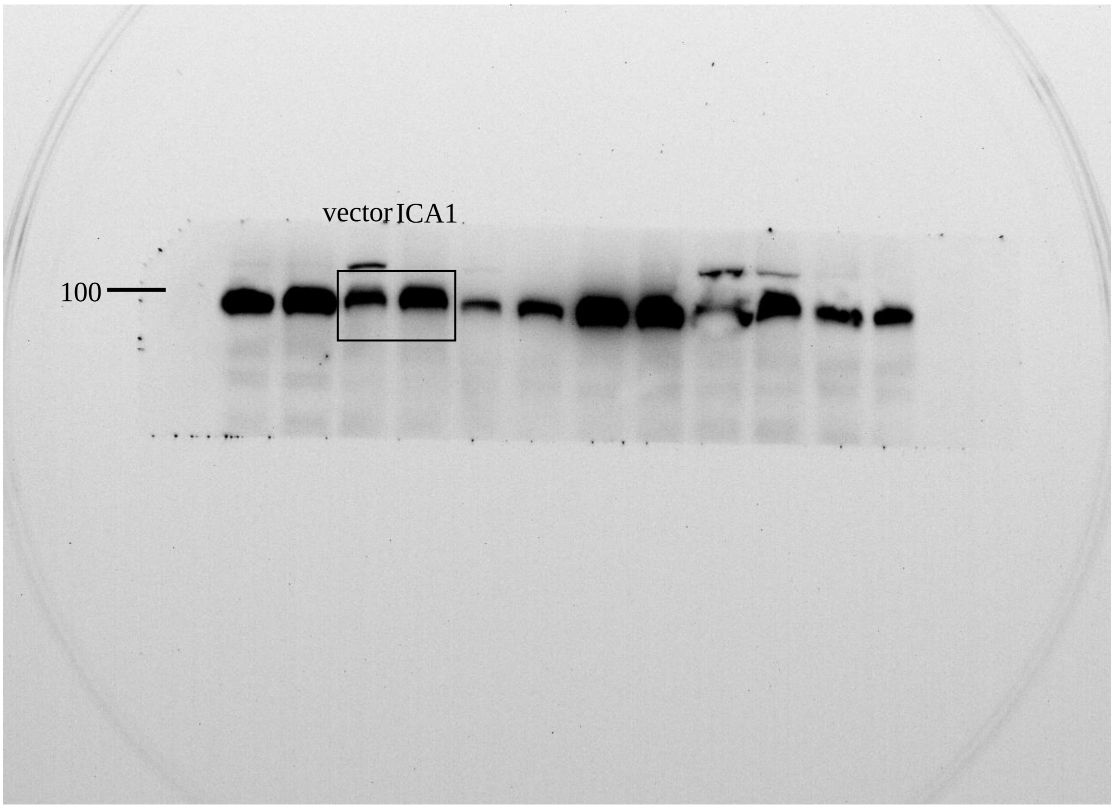

Full unedited blot for Figure 2c ADAM17

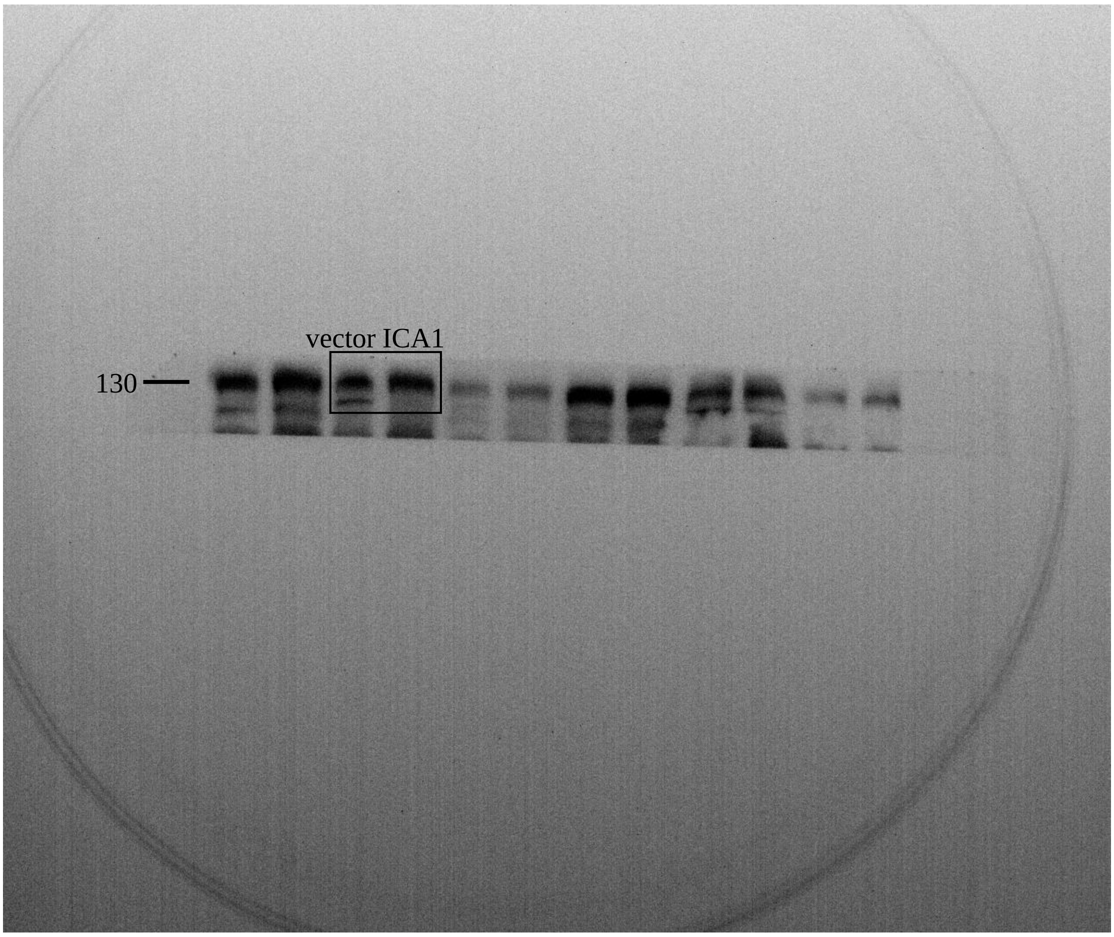

Full unedited blot for Figure 2c BACE1

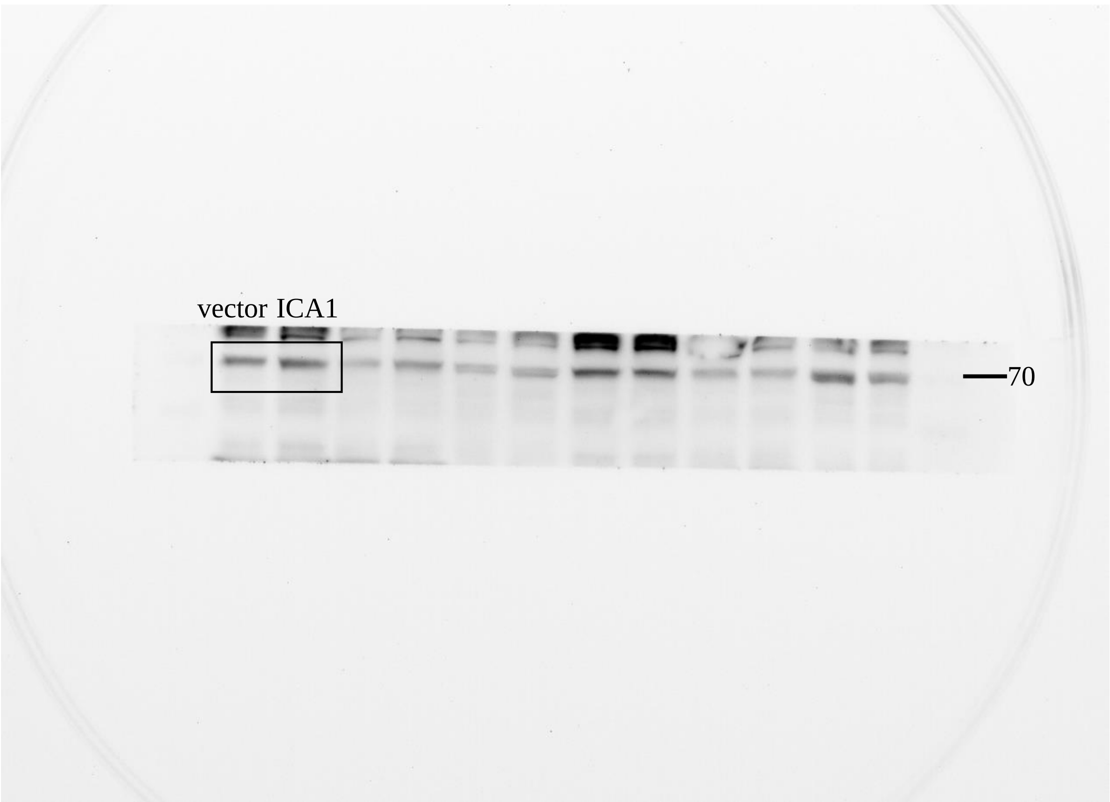

Full unedited blot for Figure 2c PS1

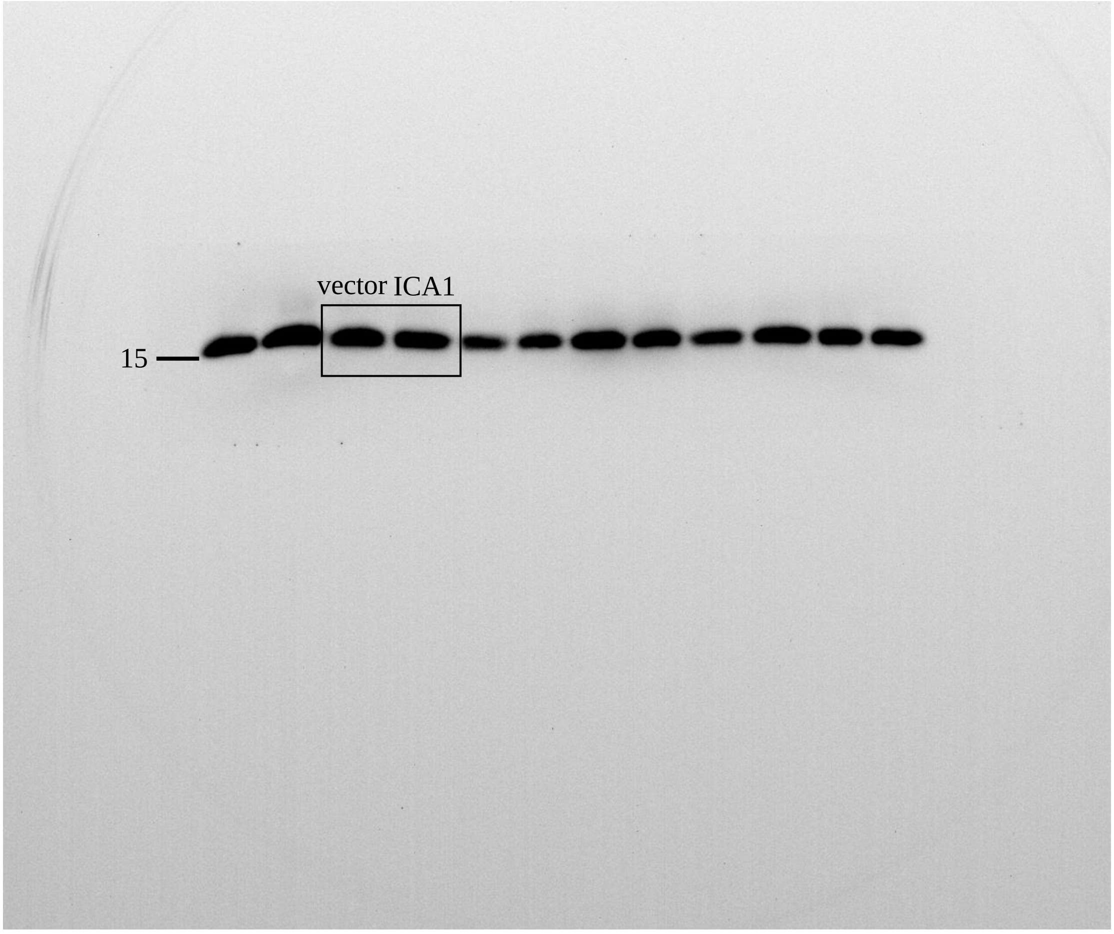

Full unedited blot for Figure 2c GAPDH

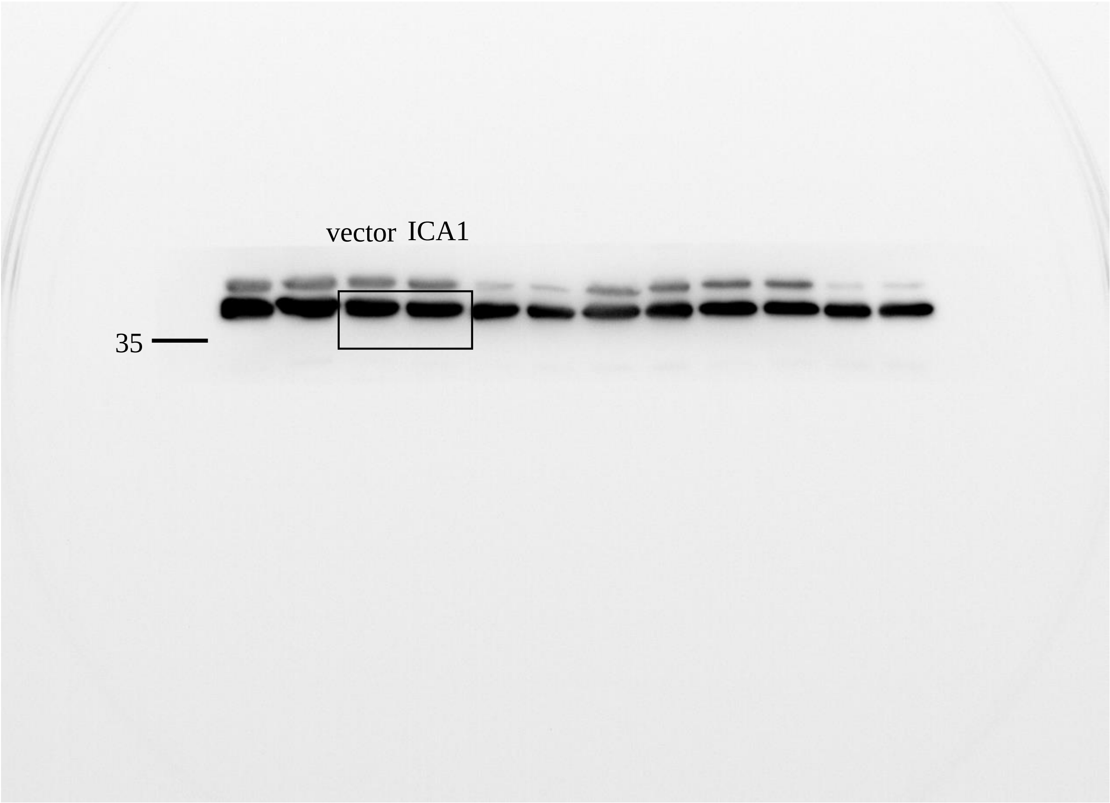

Full unedited blot for Figure 3a ICA1

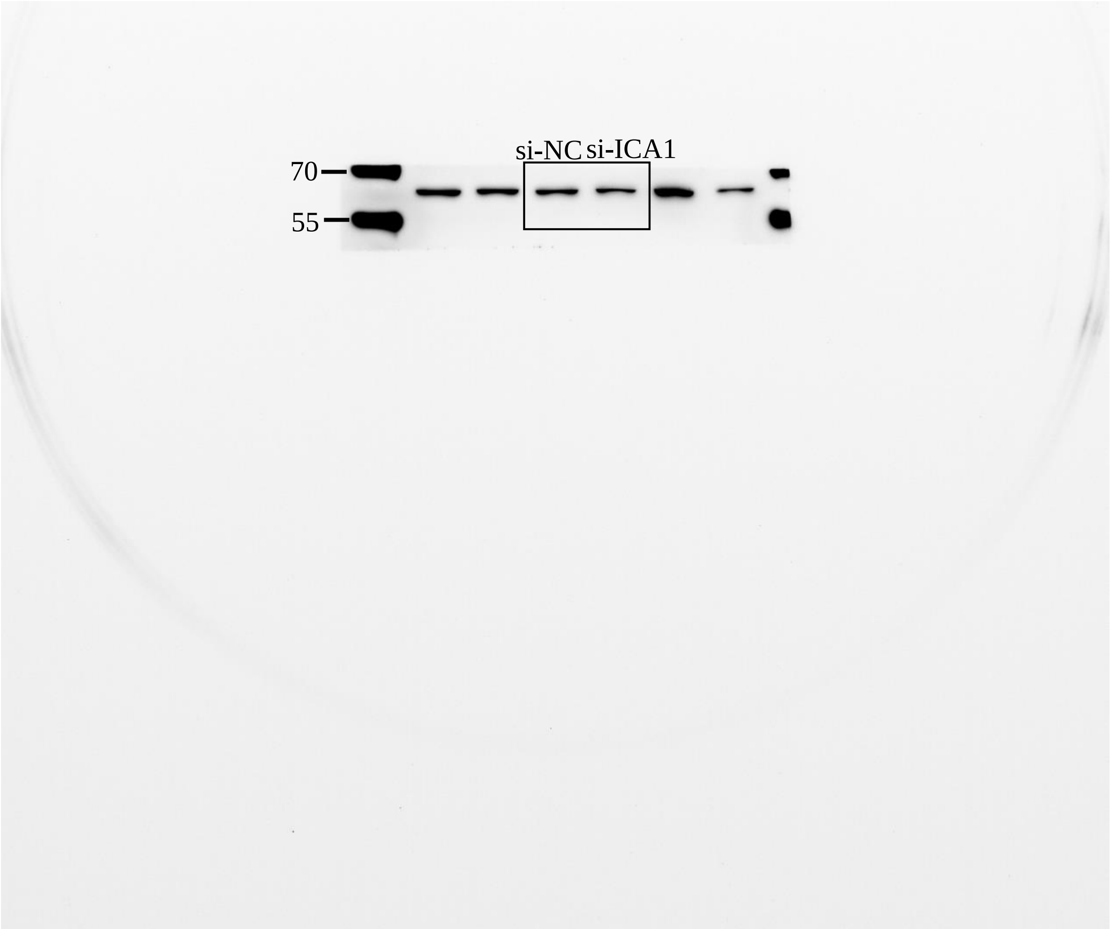

Full unedited blot for Figure 3a C89 and C99

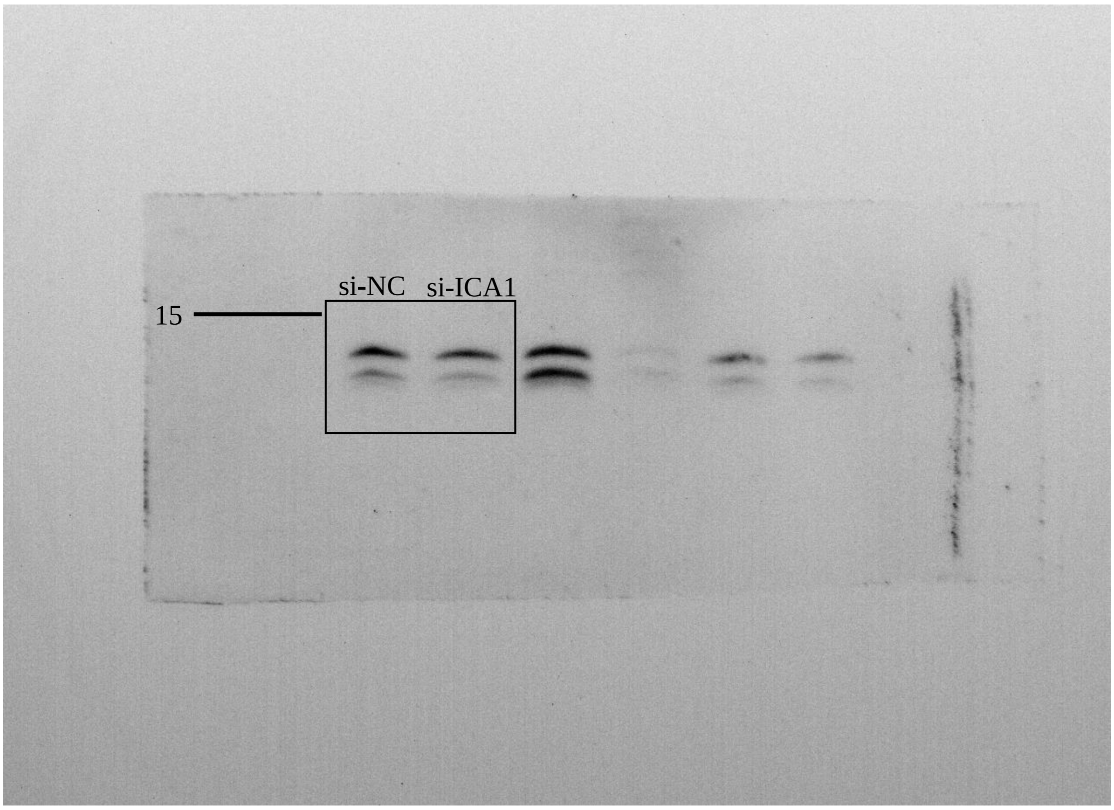

Full unedited blot for Figure 3a APP

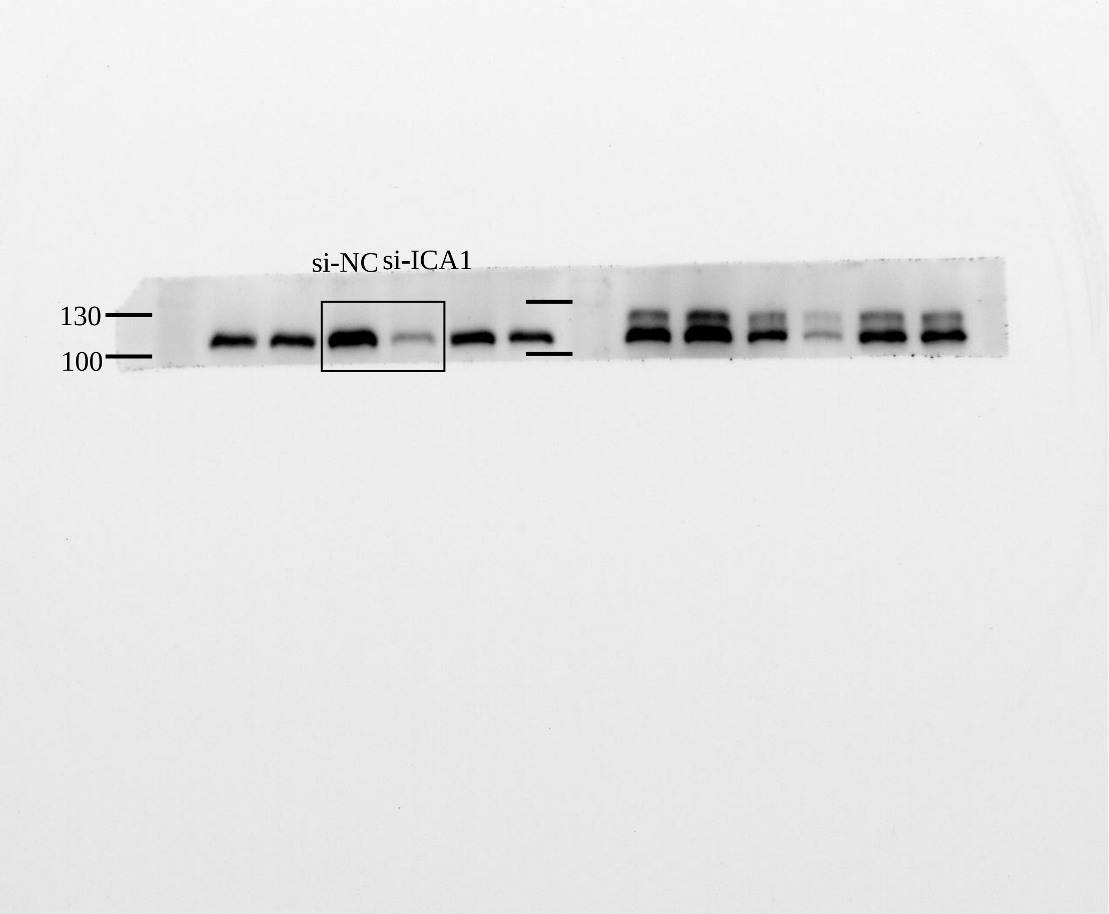

Full unedited blot for Figure 3a ADAM10

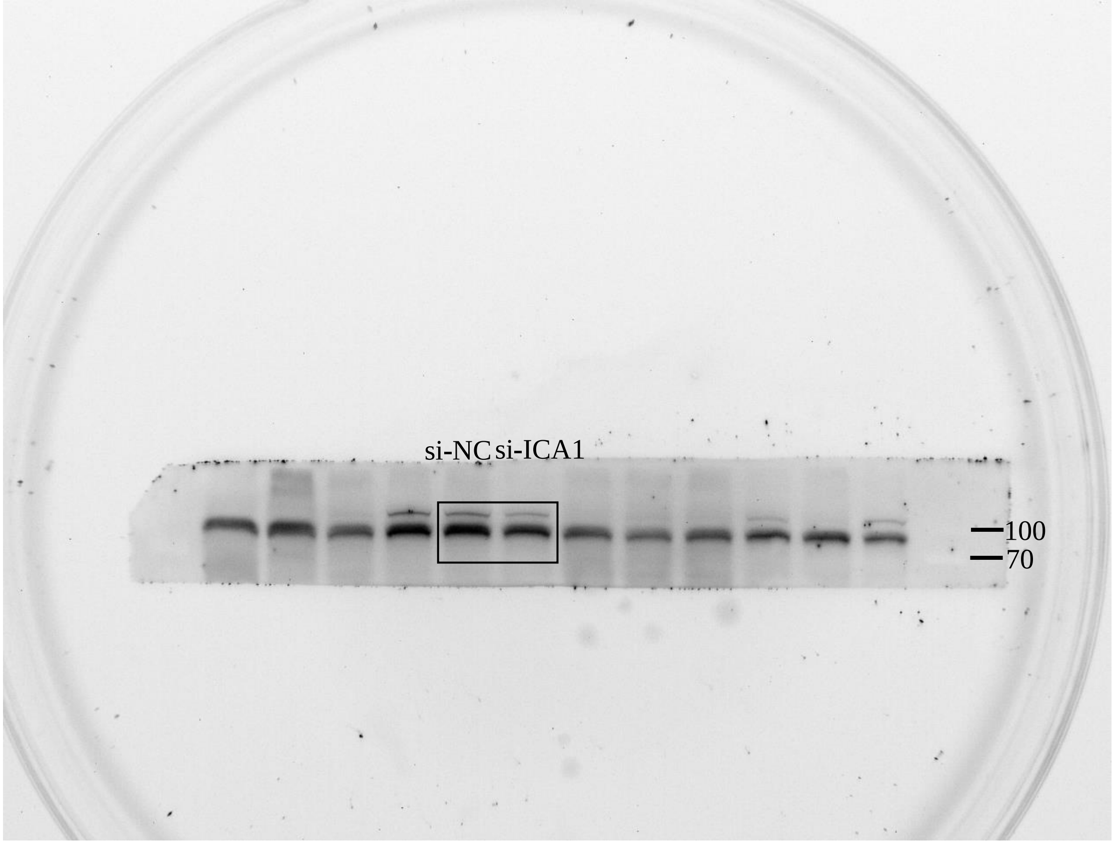

Full unedited blot for Figure 3a ADAM17

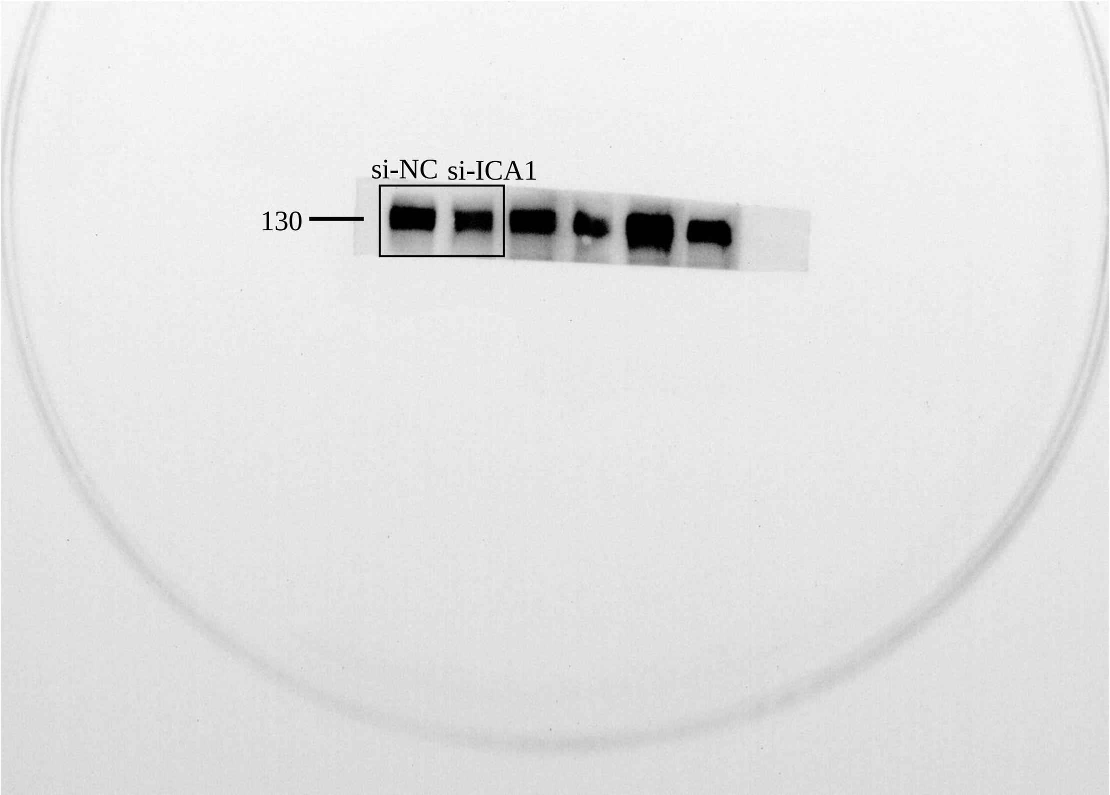

Full unedited blot for Figure 3a BACE1

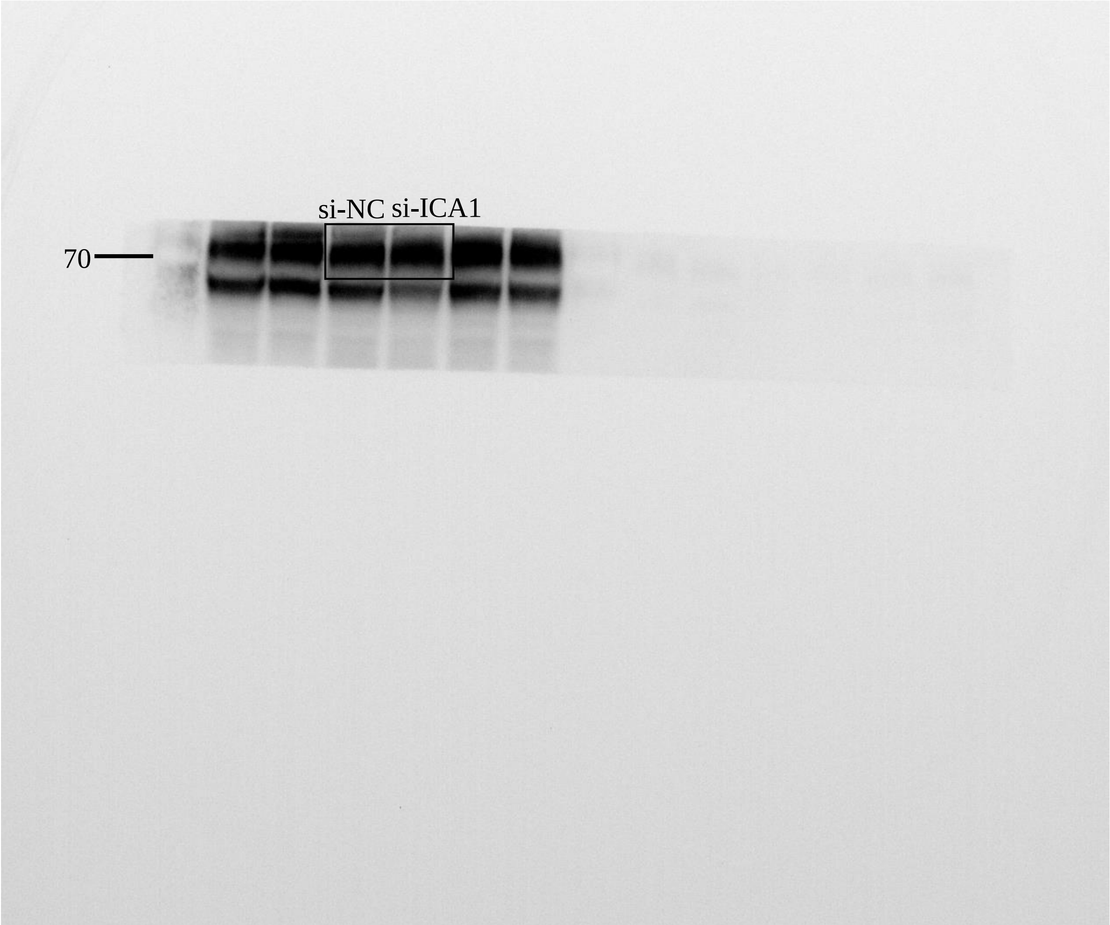

Full unedited blot for Figure 3a PS1

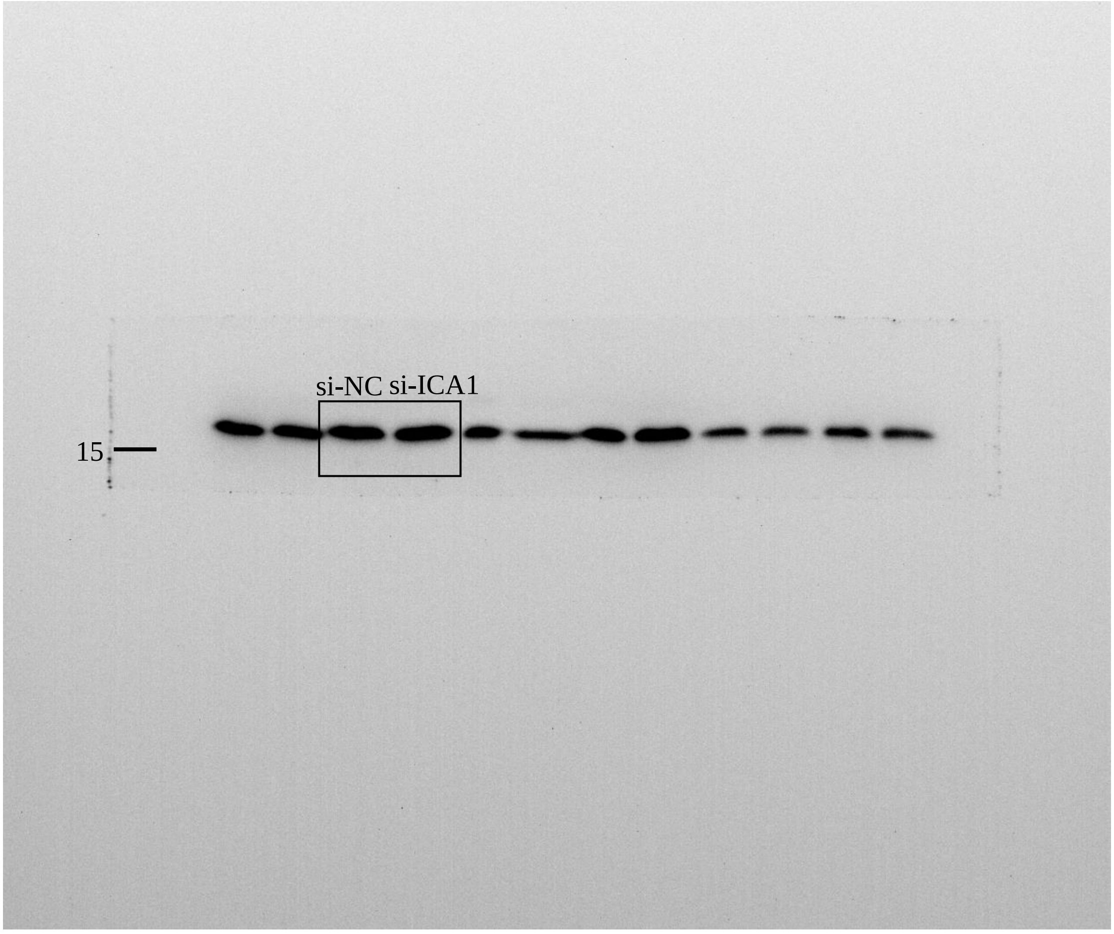

Full unedited blot for Figure 3a GAPDH

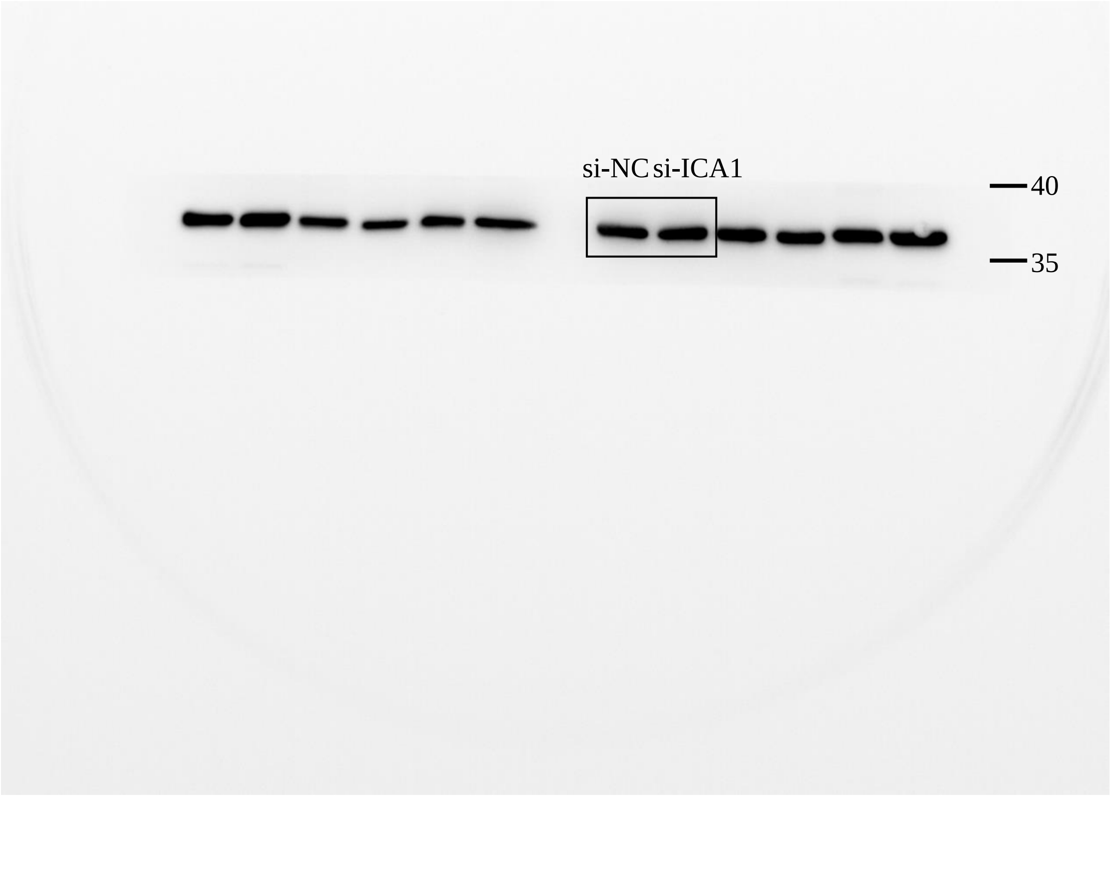

Full unedited blot for Figure 3b ICA1

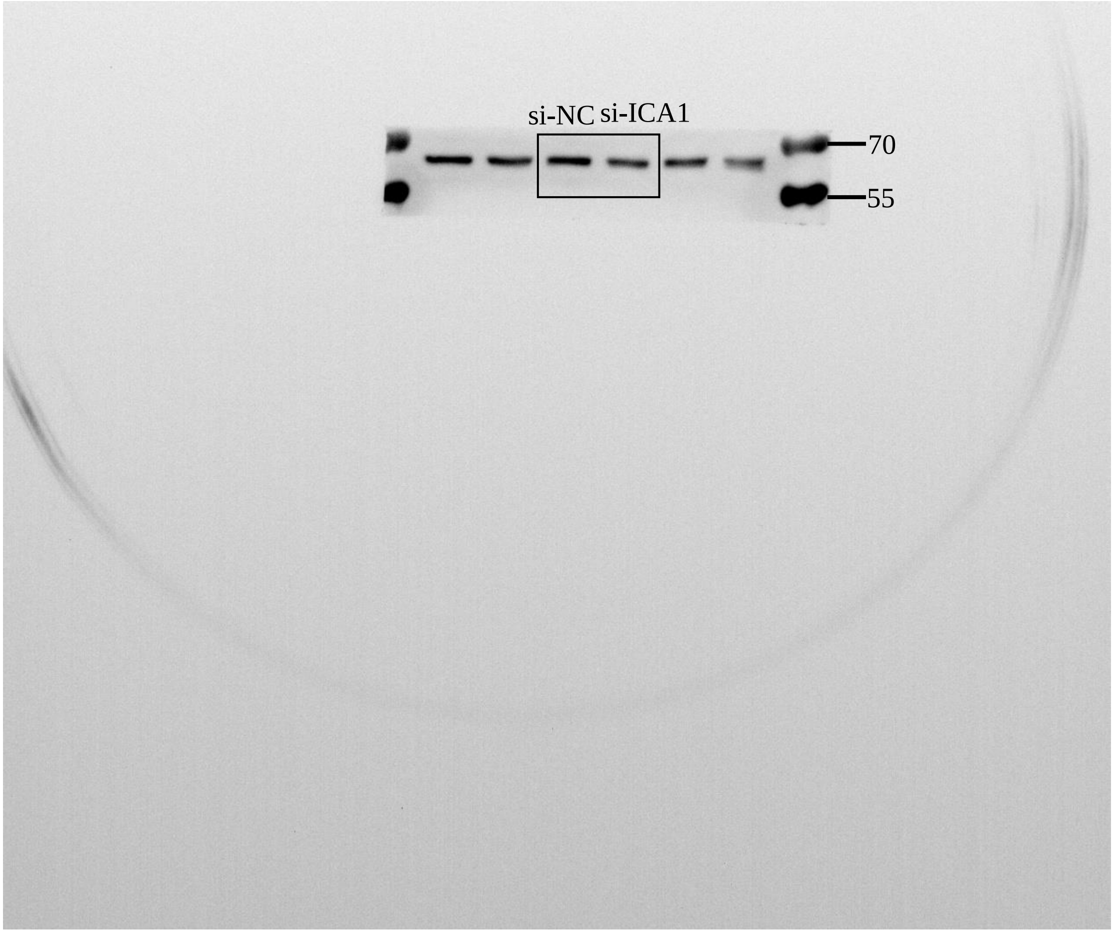

Full unedited blot for Figure 3b C83

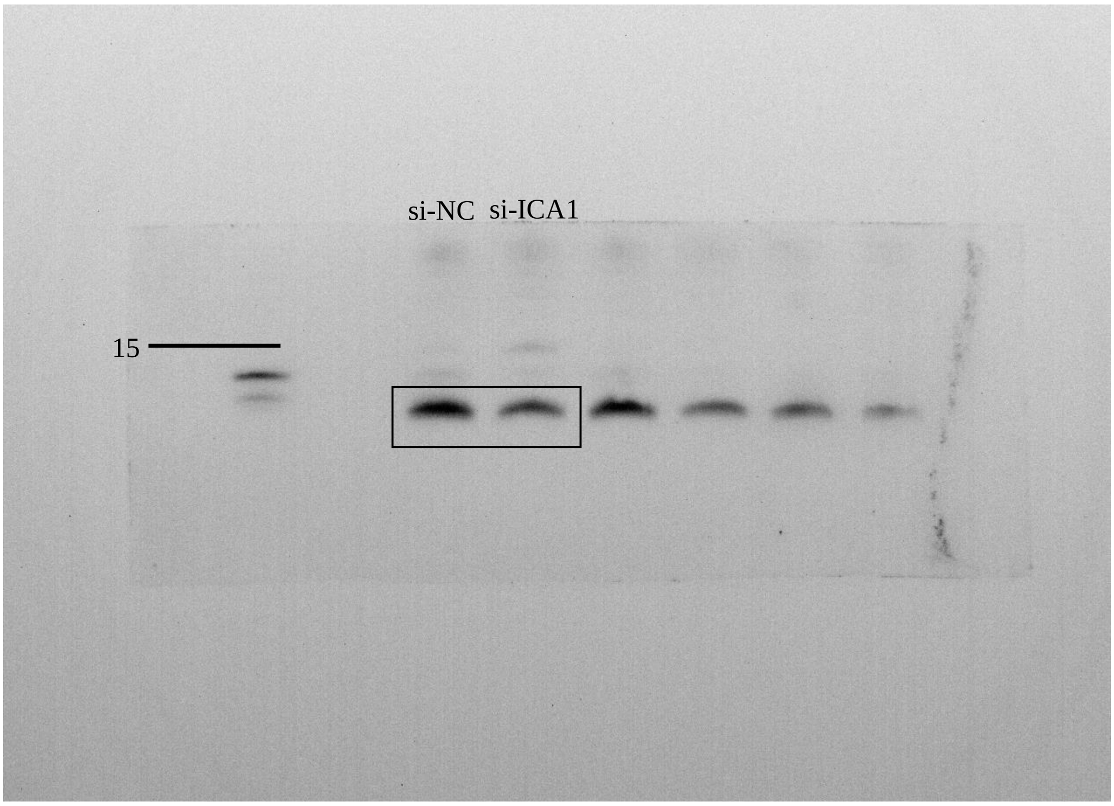

Full unedited blot for Figure 3b APP

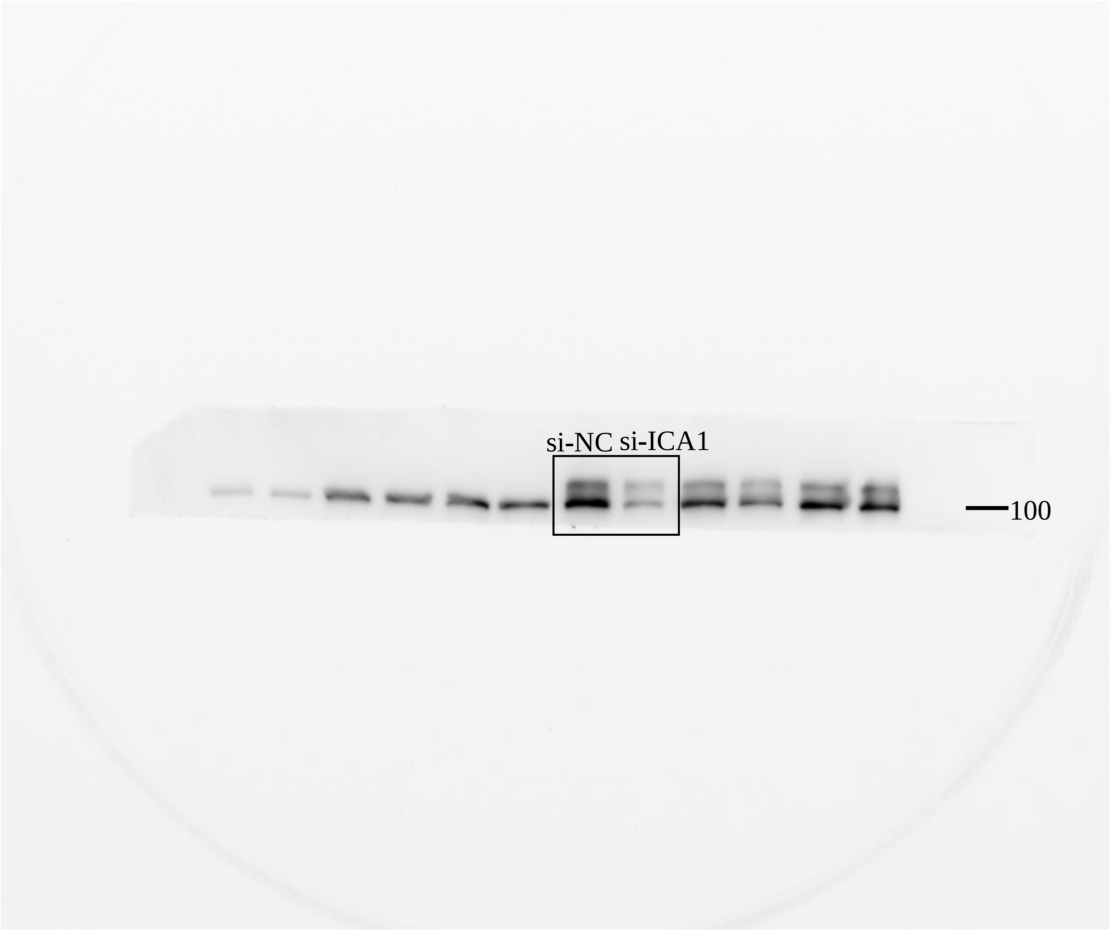

Full unedited blot for Figure 3b ADAM10

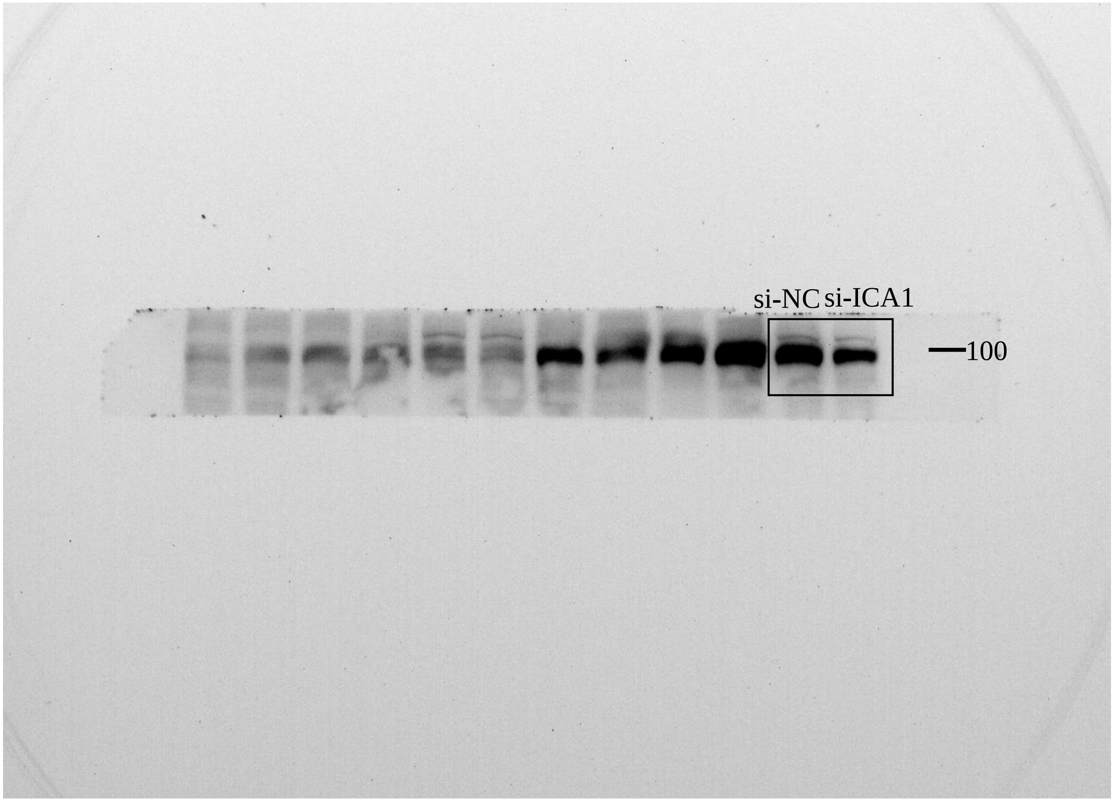

Full unedited blot for Figure 3b ADAM17

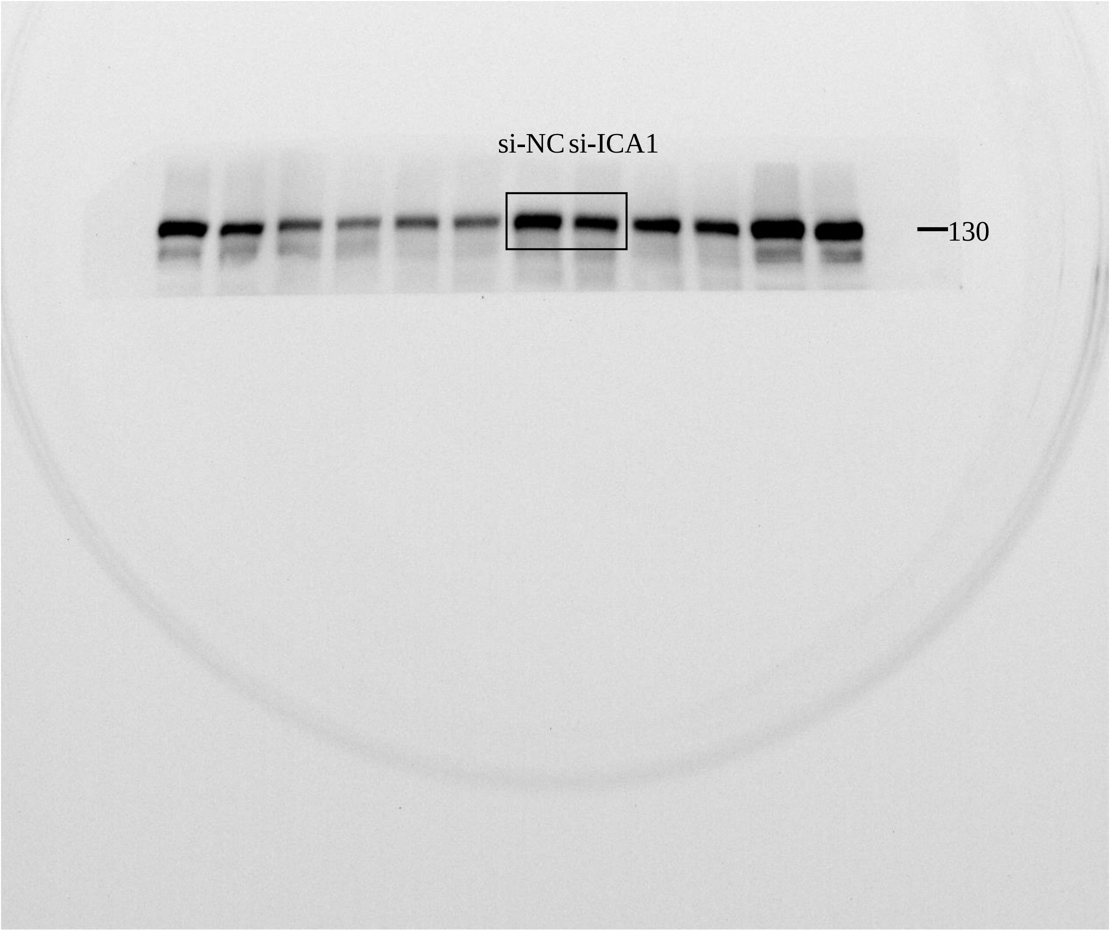

Full unedited blot for Figure 3b BACE1

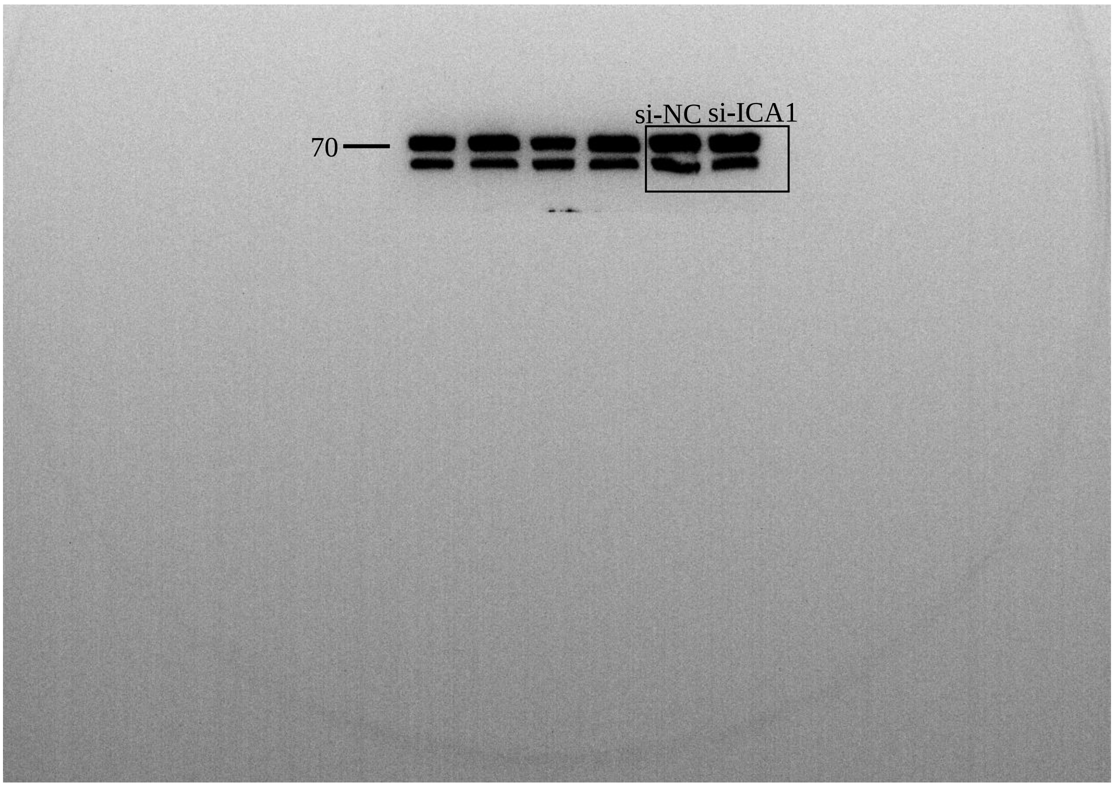

Full unedited blot for Figure 3b PS1

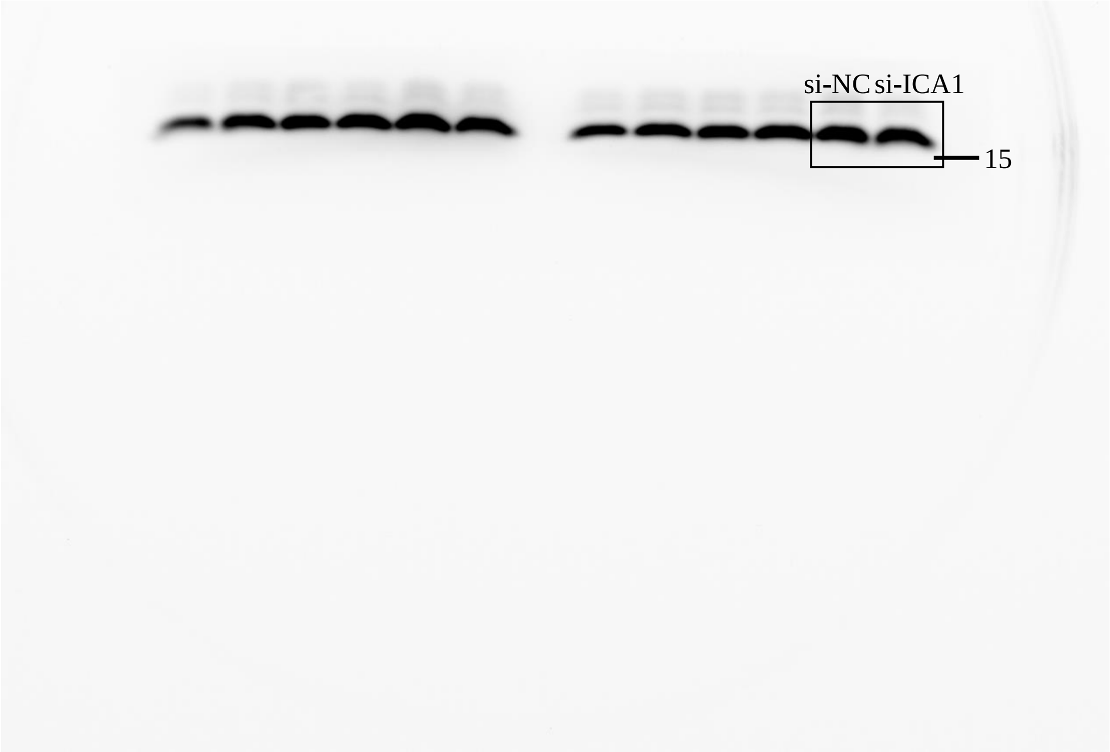

Full unedited blot for Figure 3b GAPDH

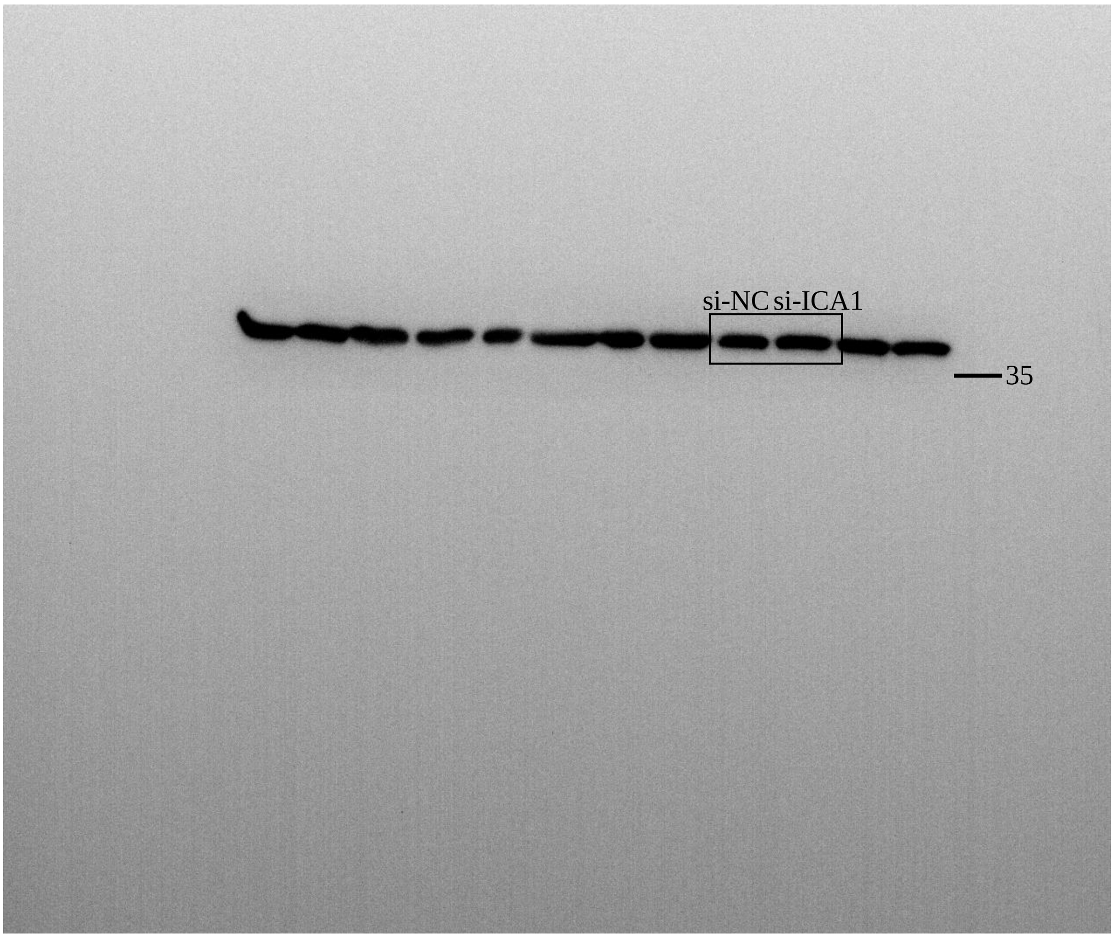

Full unedited blot for Figure 3c ICA1

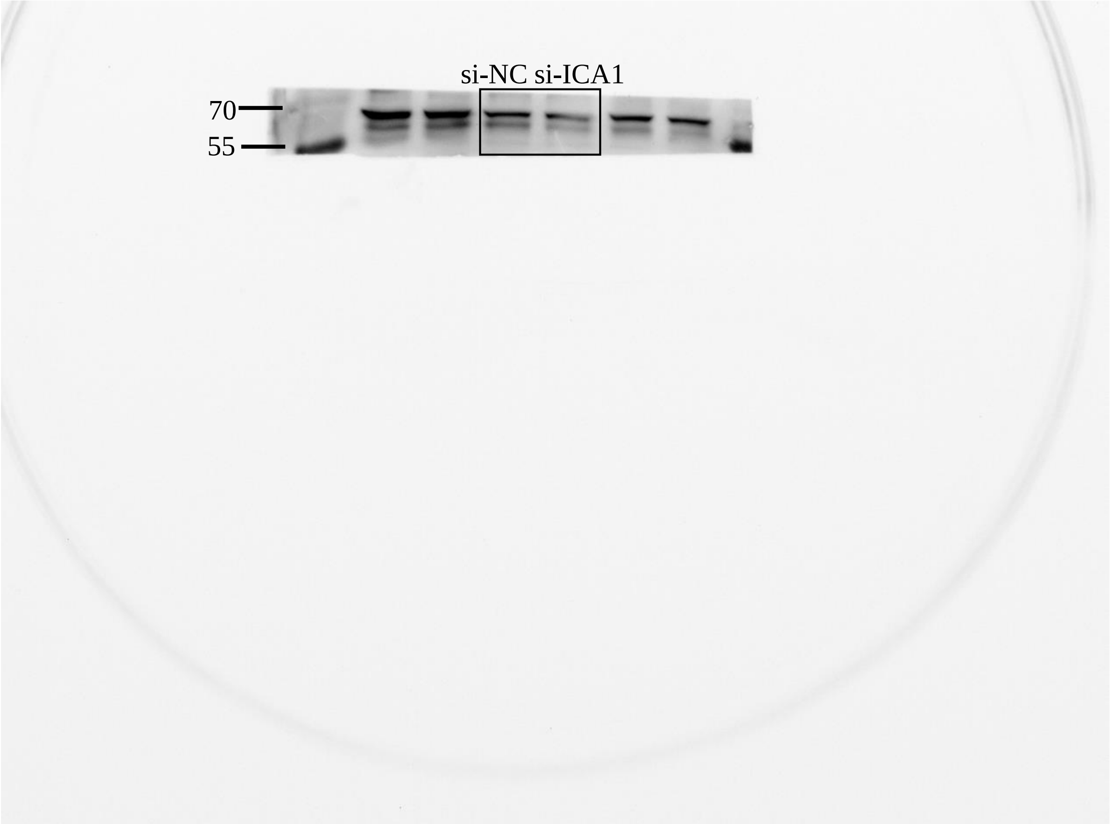

Full unedited blot for Figure 3c C83 and C99

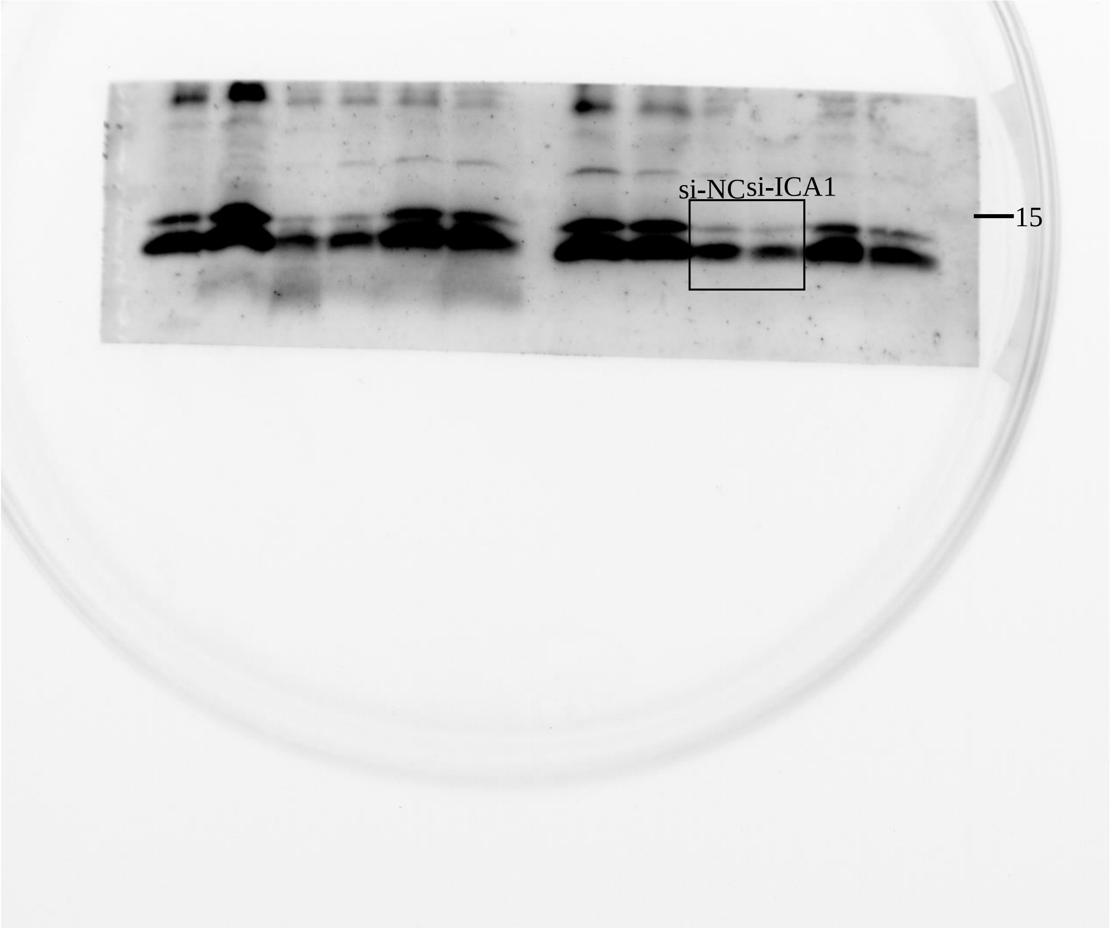

Full unedited blot for Figure 3c APP

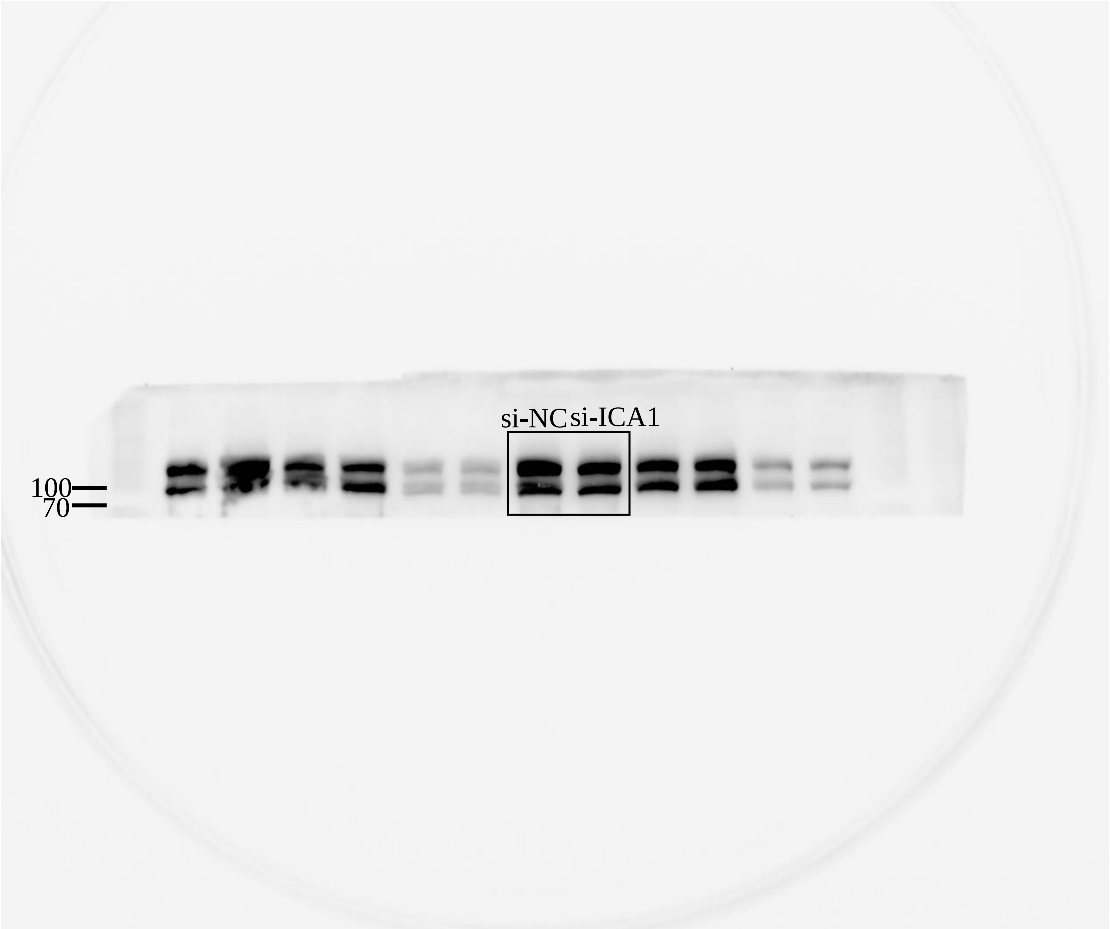

Full unedited blot forFigure 3c ADAM10

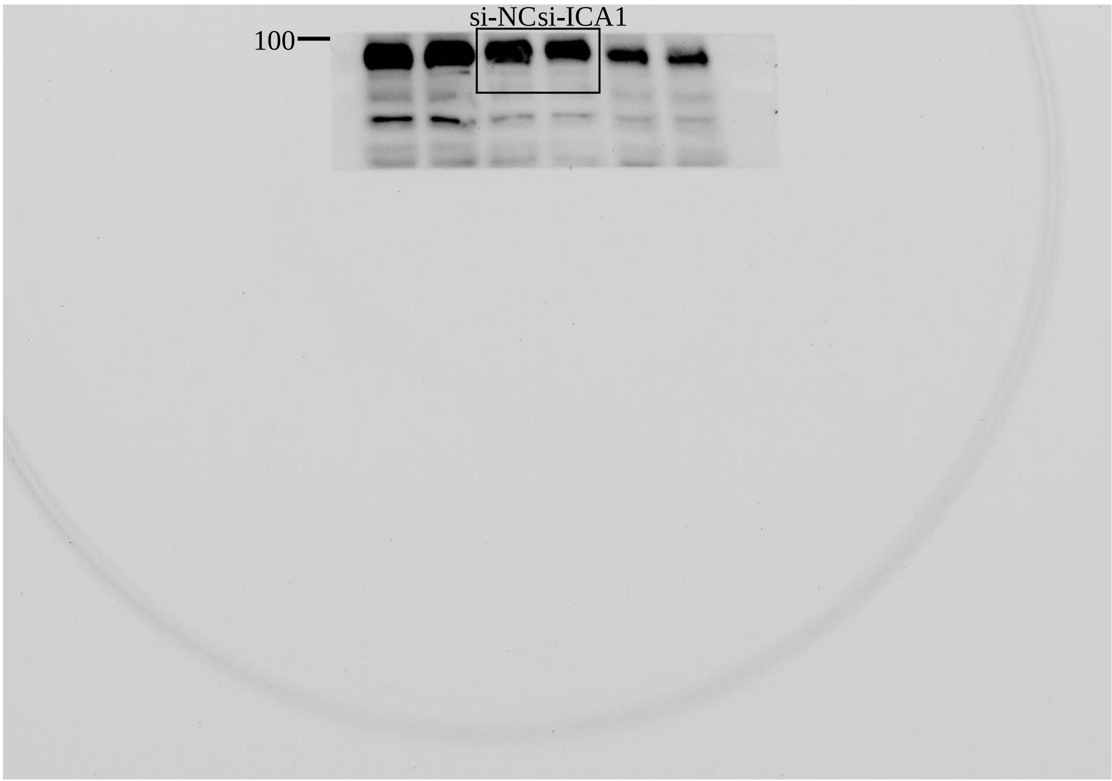

Full unedited blot for Figure 3c ADAM17

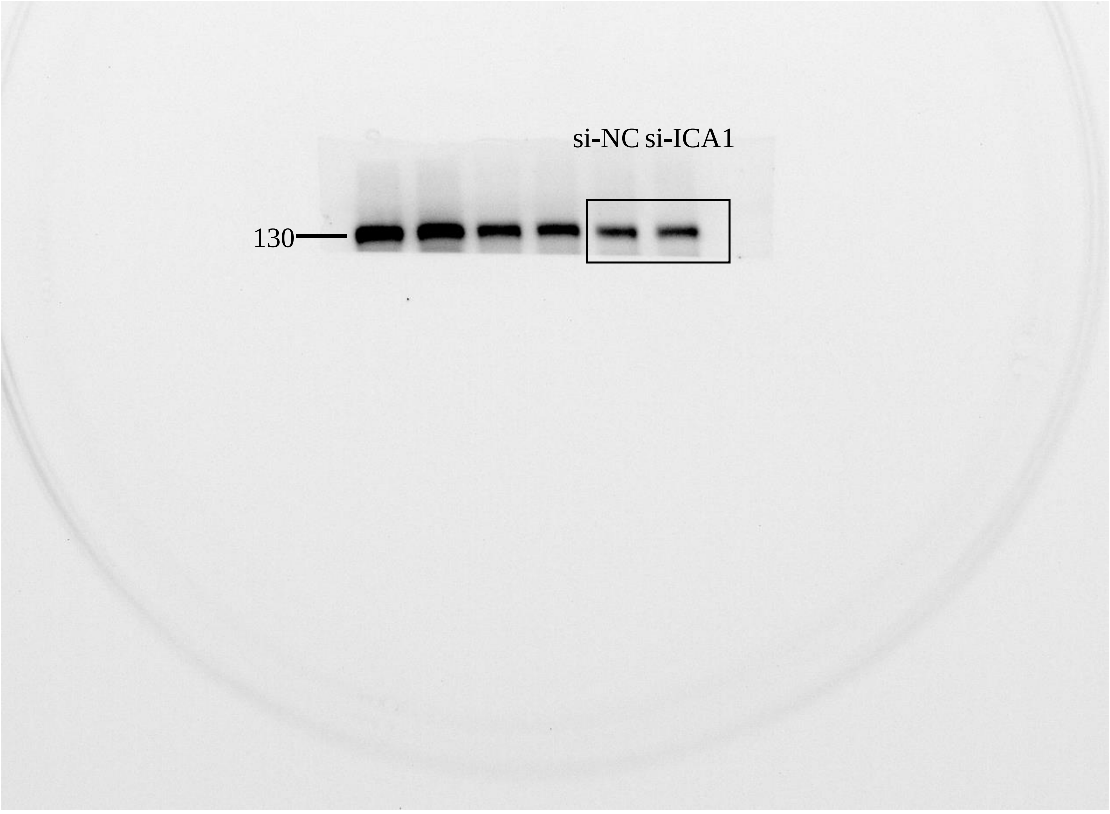

Full unedited blot for Figure 3c BACE1

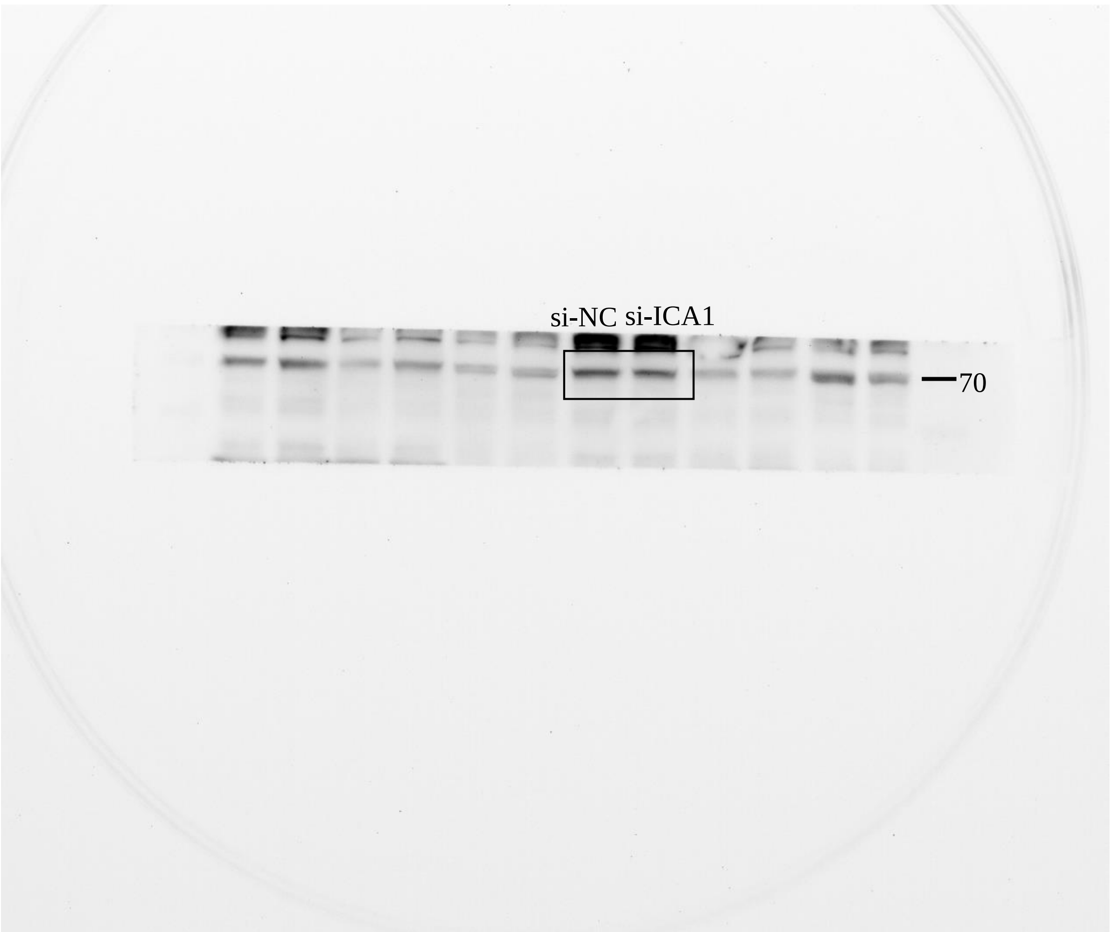

Full unedited blot for Figure 3c PS1

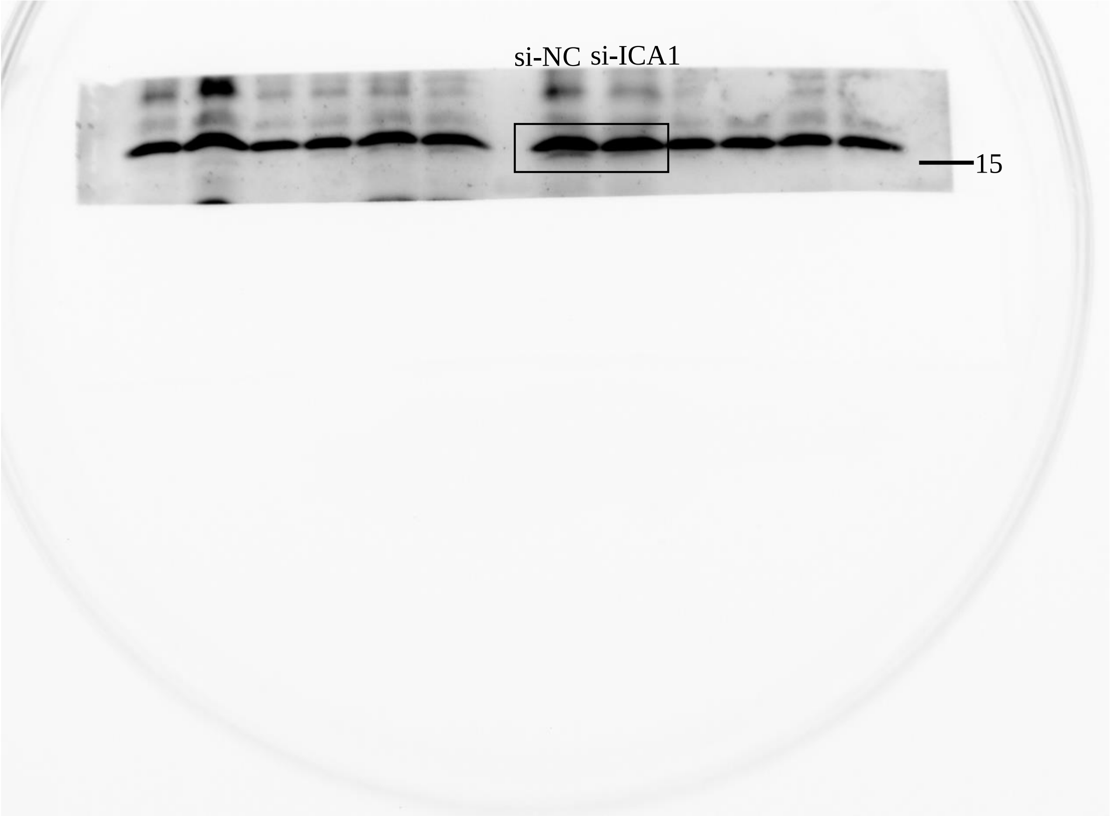

Full unedited blot for Figure 3c GAPDH

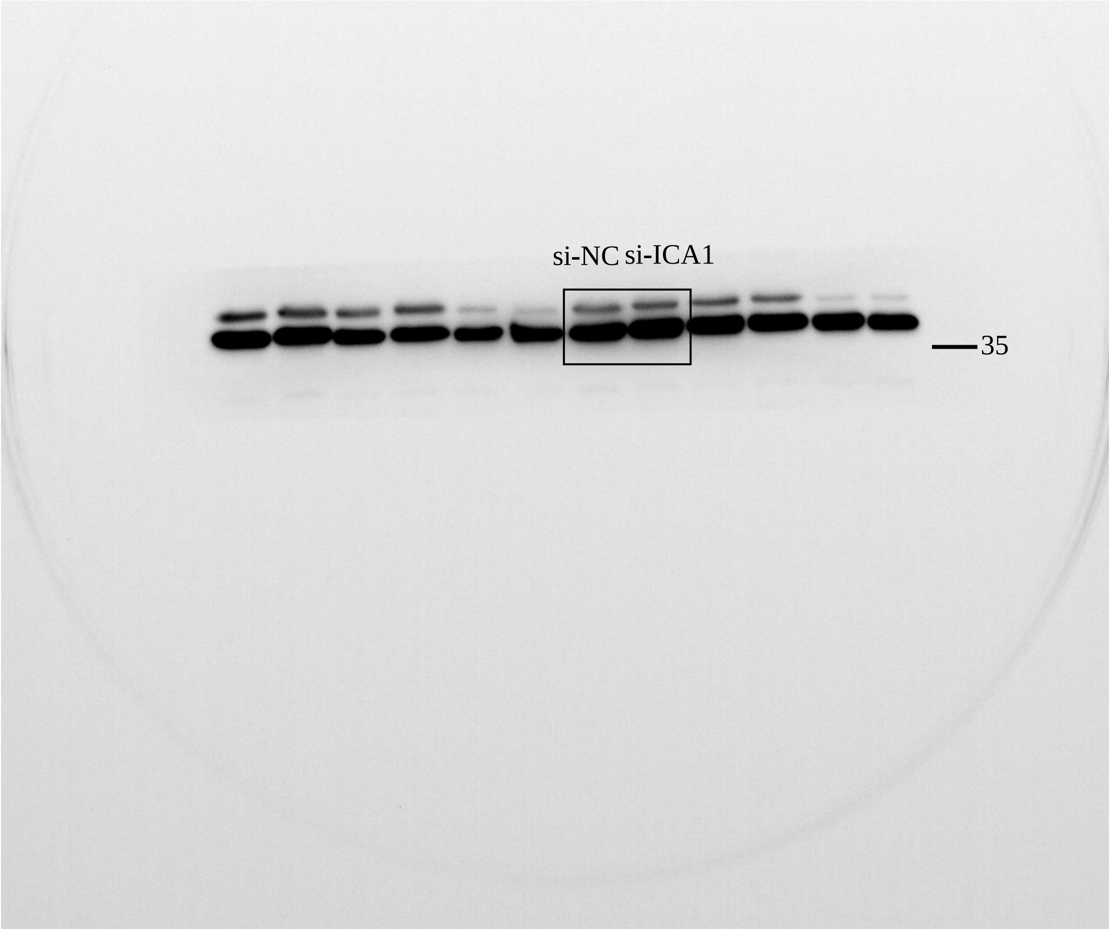

Full unedited blot for Figure 4a APP

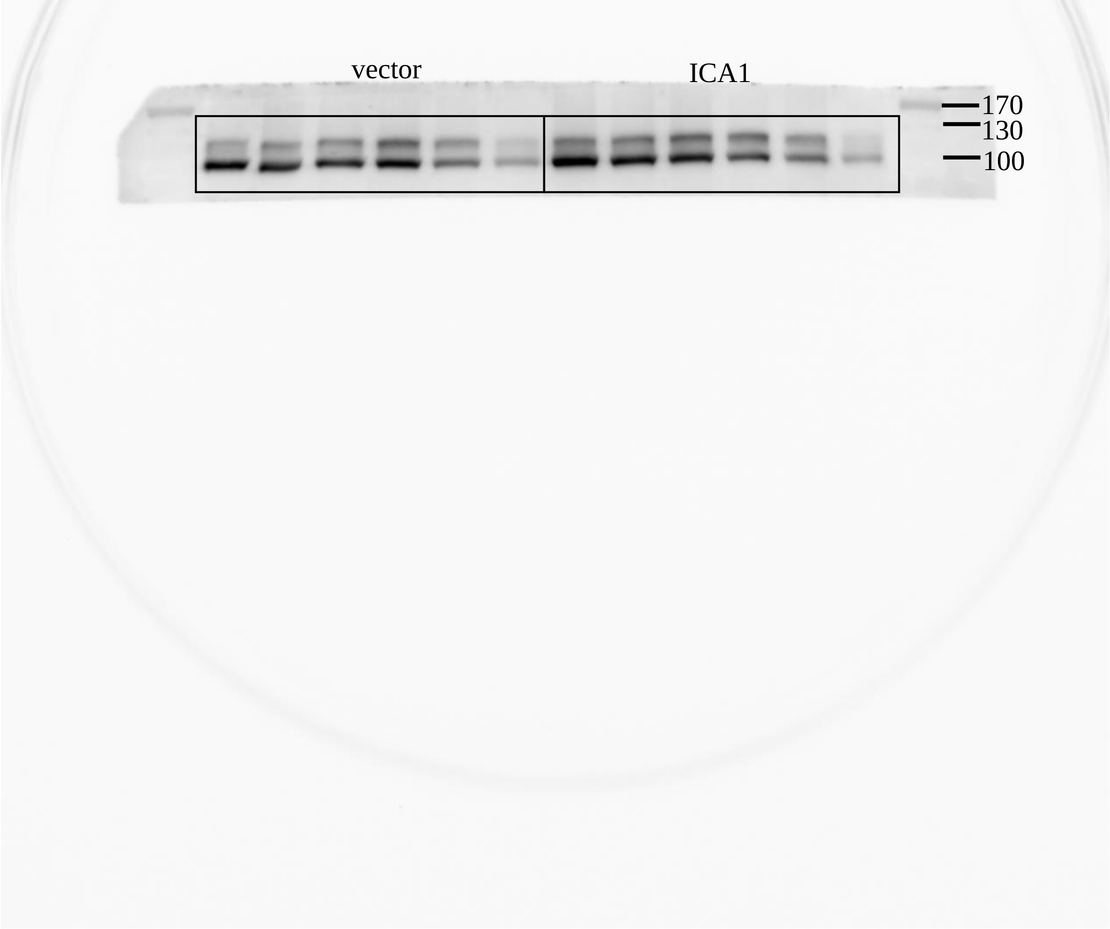

Full unedited blot forFigure 4a GAPDH

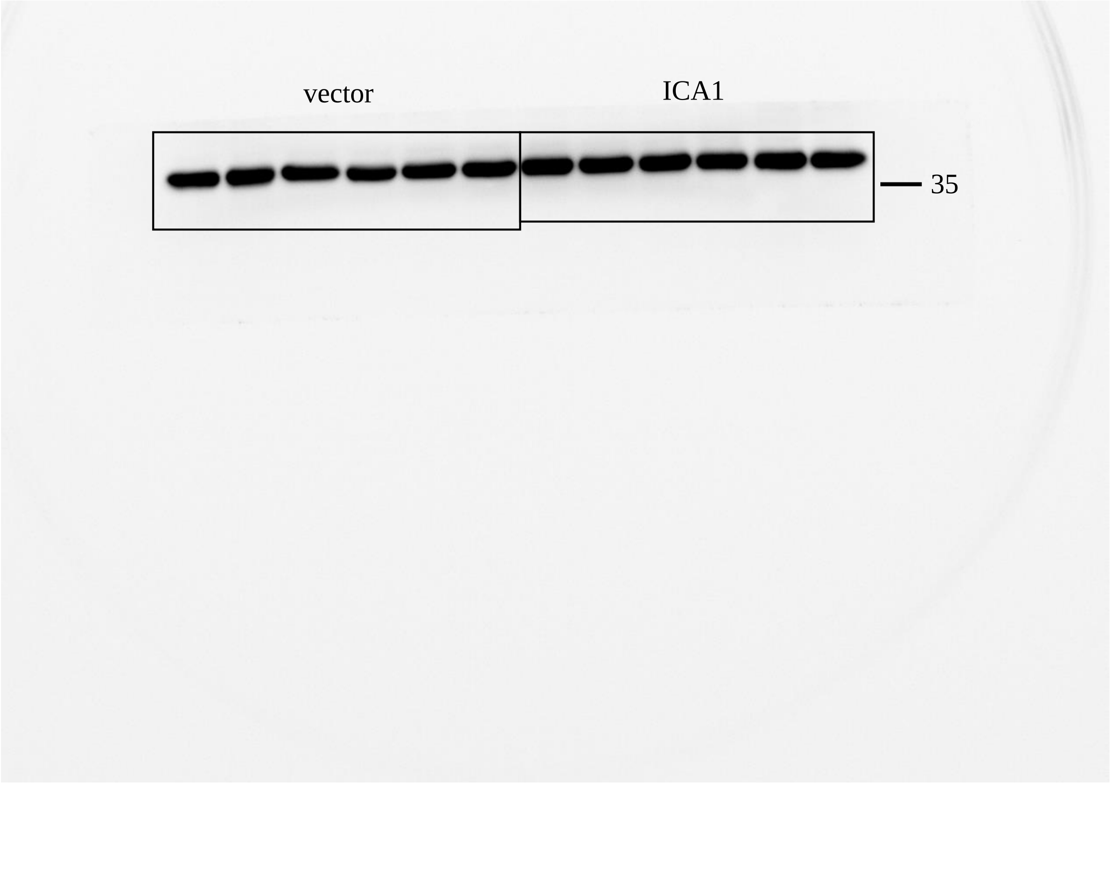

Full unedited blot for Figure 4b ADAM10

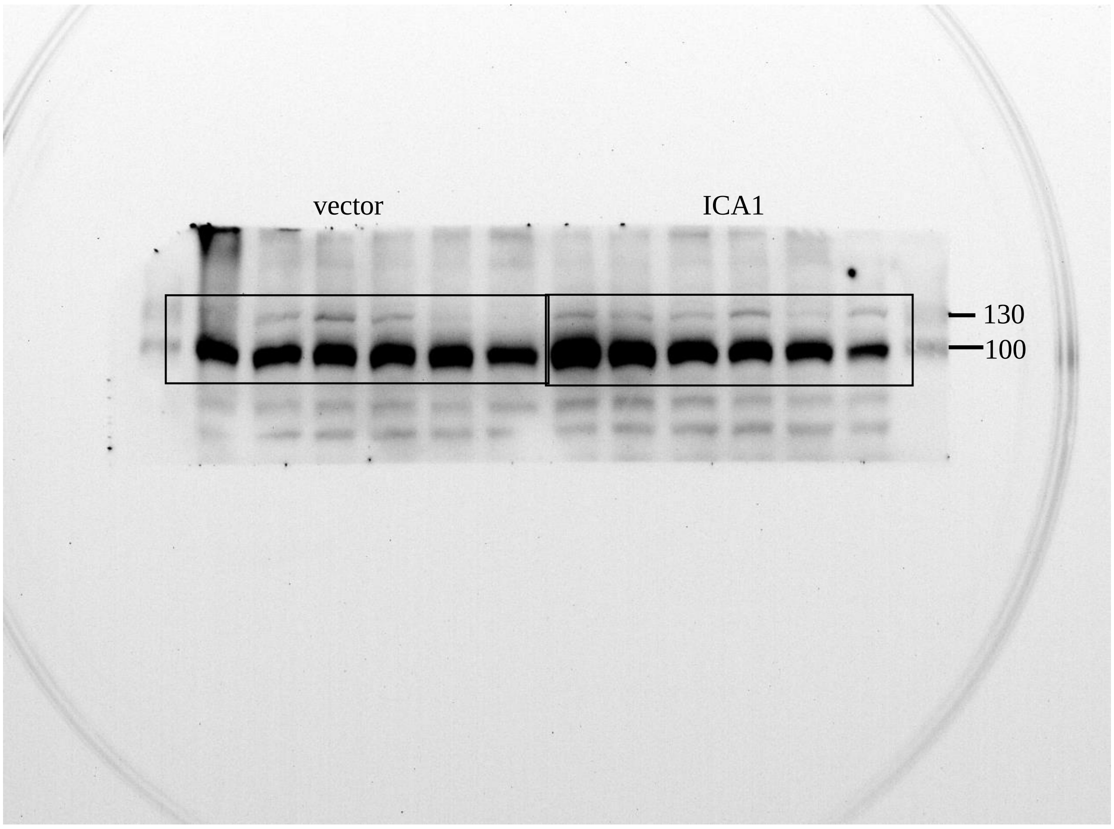

Full unedited blot for Figure 4b GAPDH

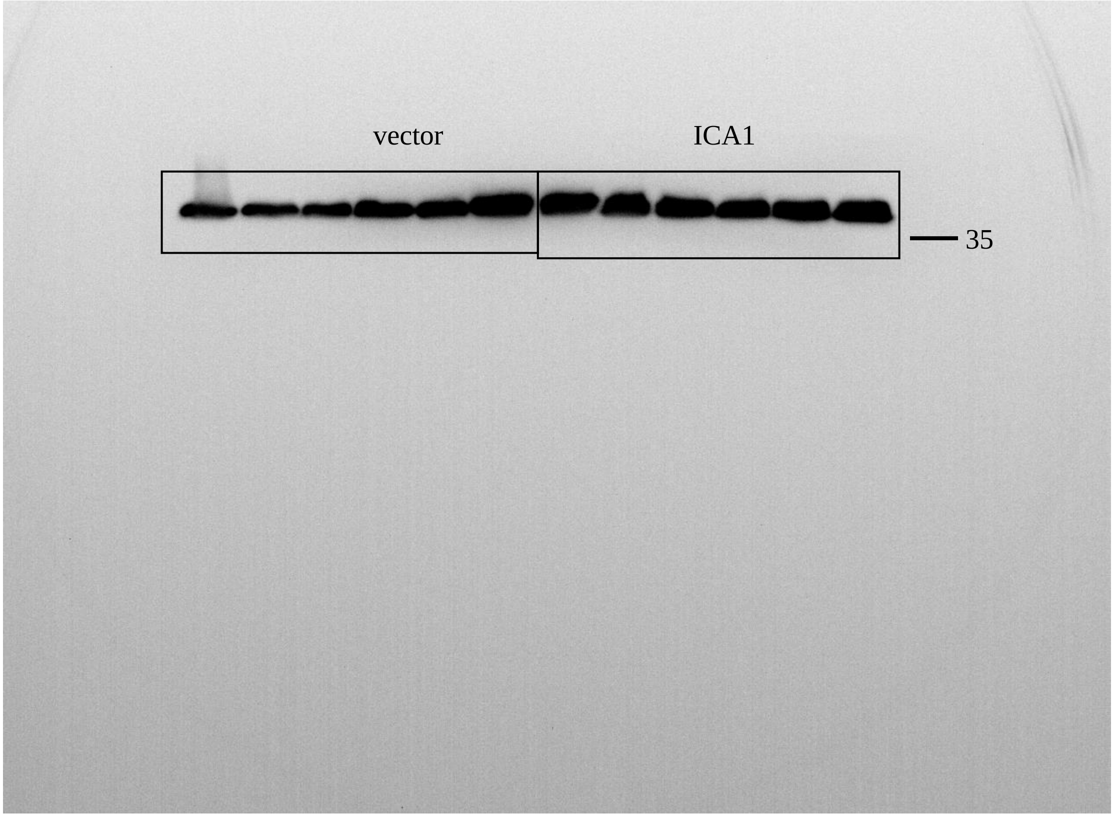

Full unedited blot for Figure 4c ADAM17

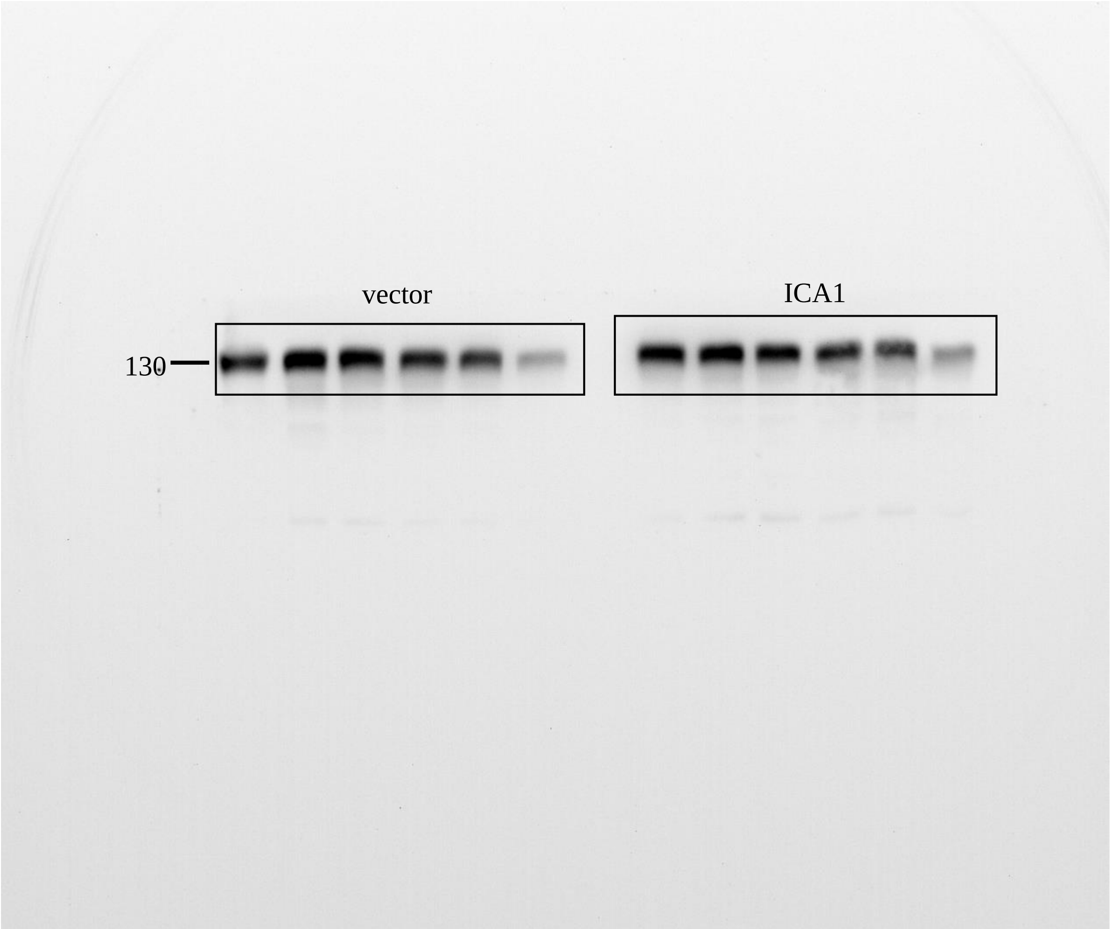

Full unedited blot for Figure 4c GAPDH

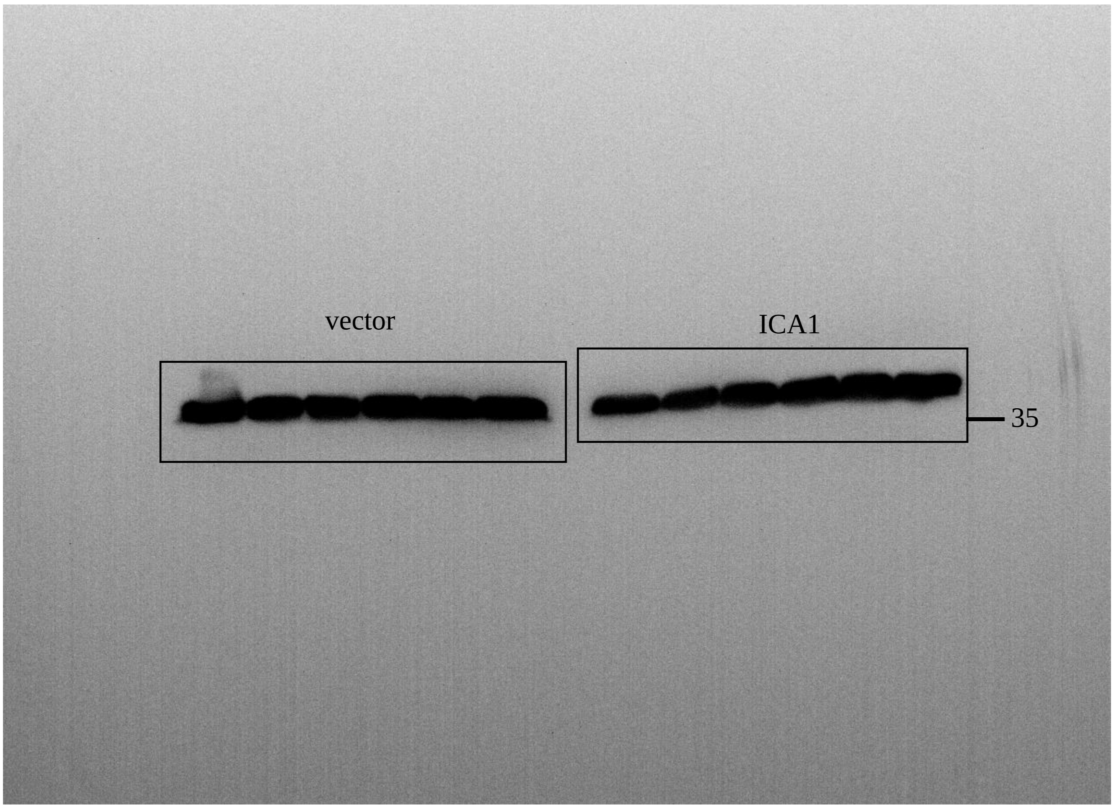

Full unedited blot for Figure 6a ICA1

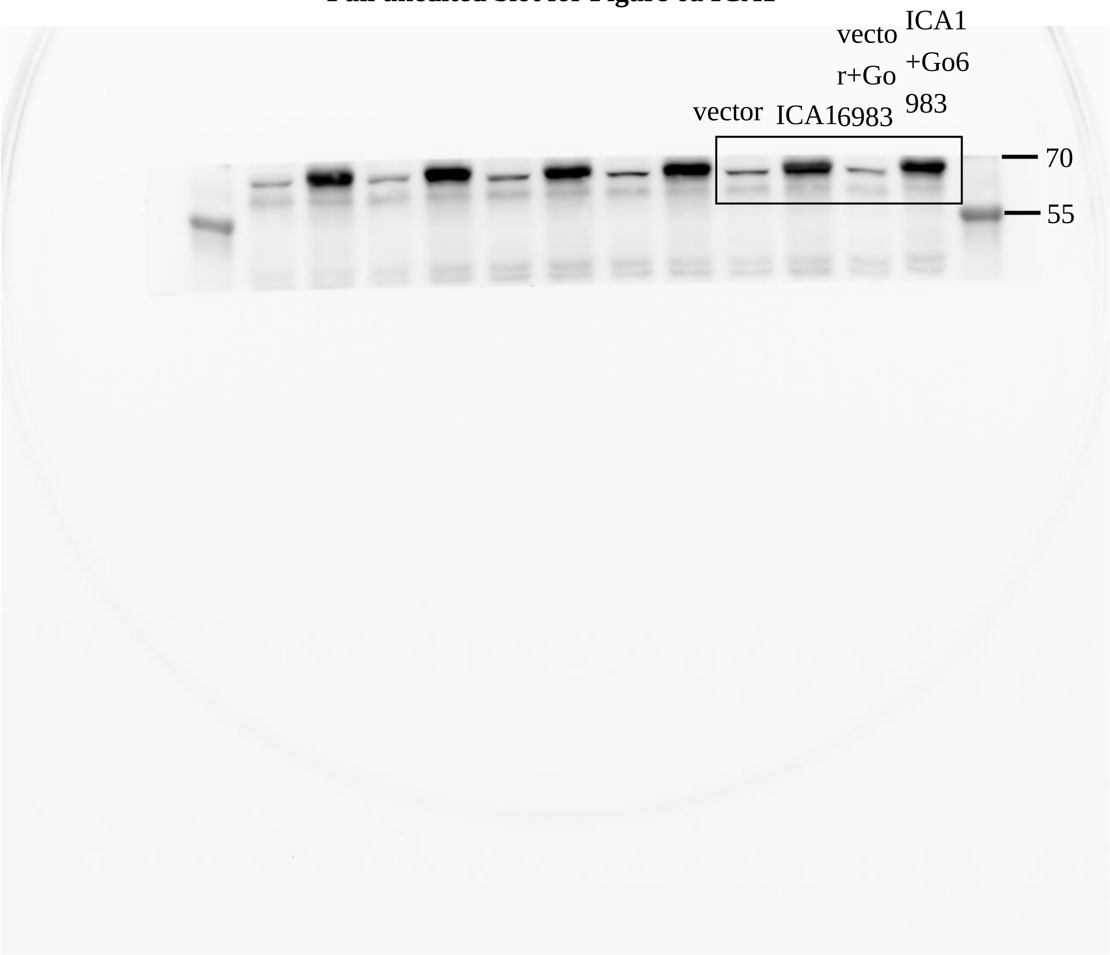

Full unedited blot for Figure 6a PICK1

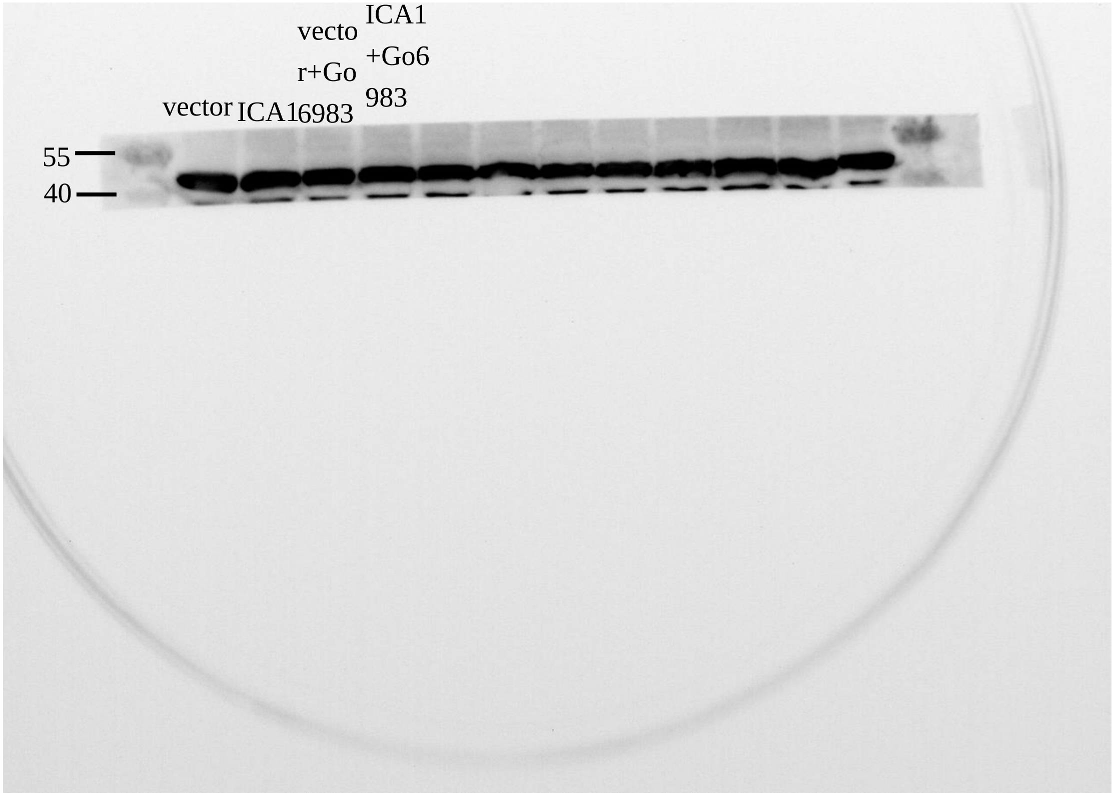

Full unedited blot for Figure 6a PKCα

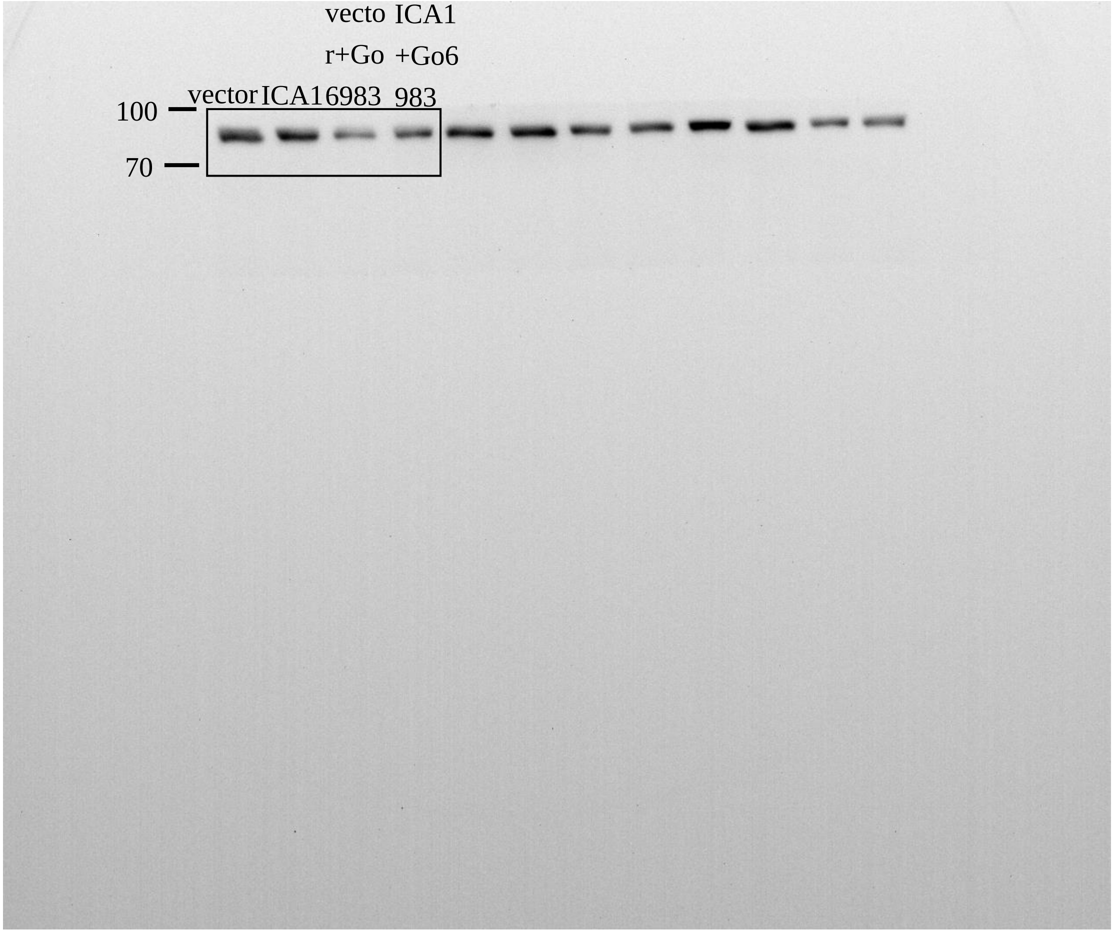

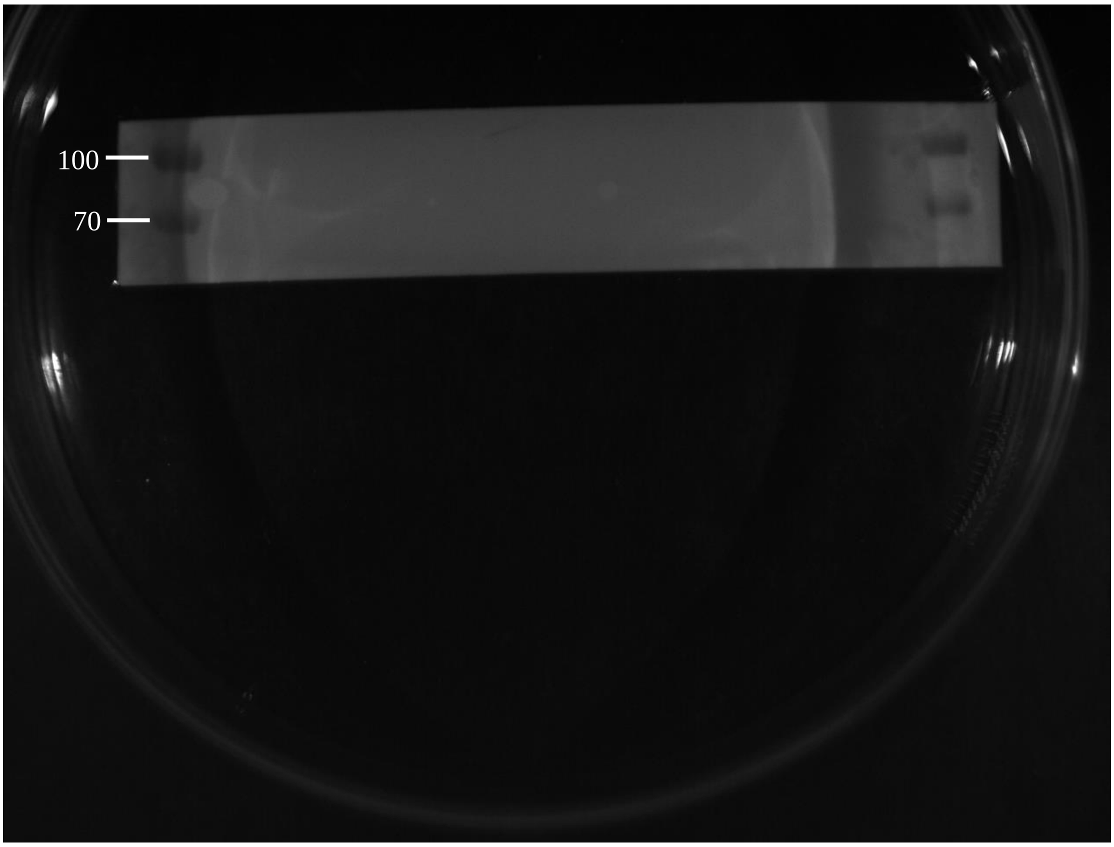

Full unedited blot for Figure 6a p-PKCα

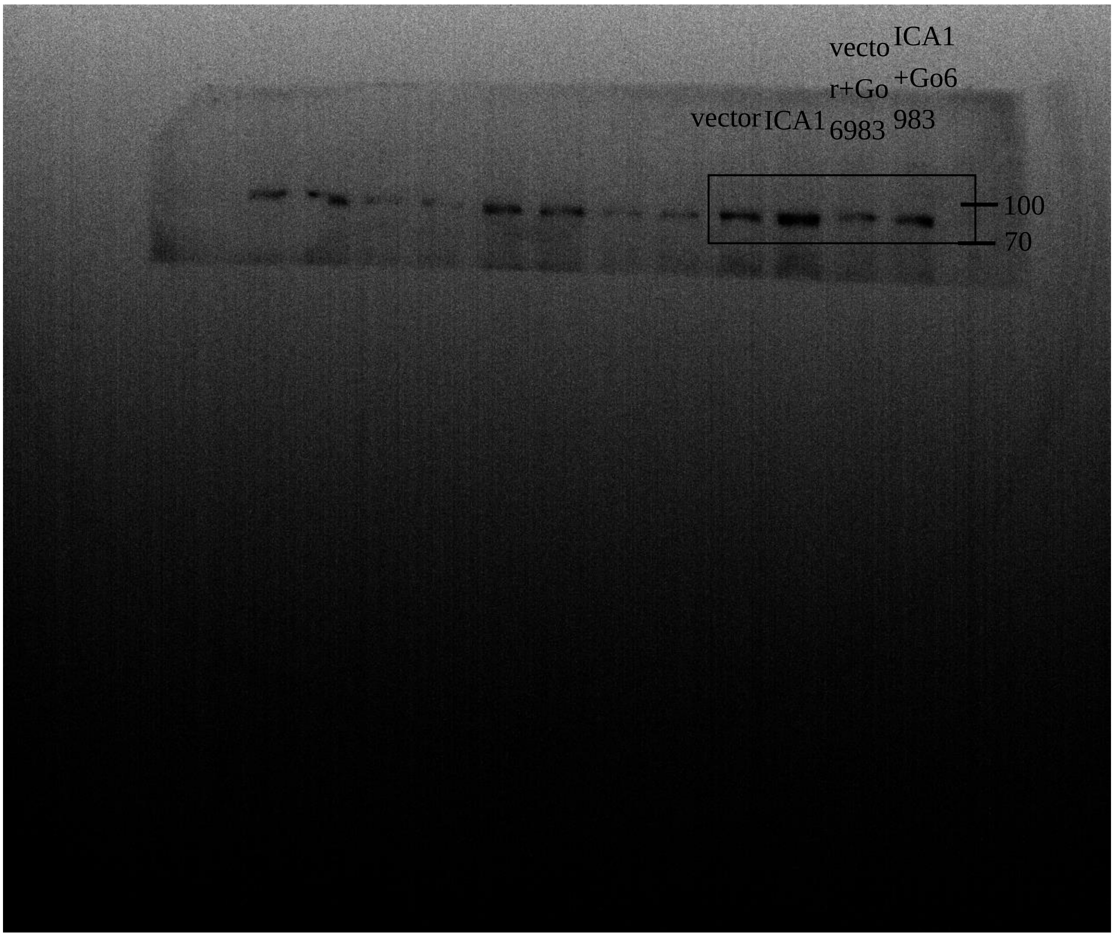

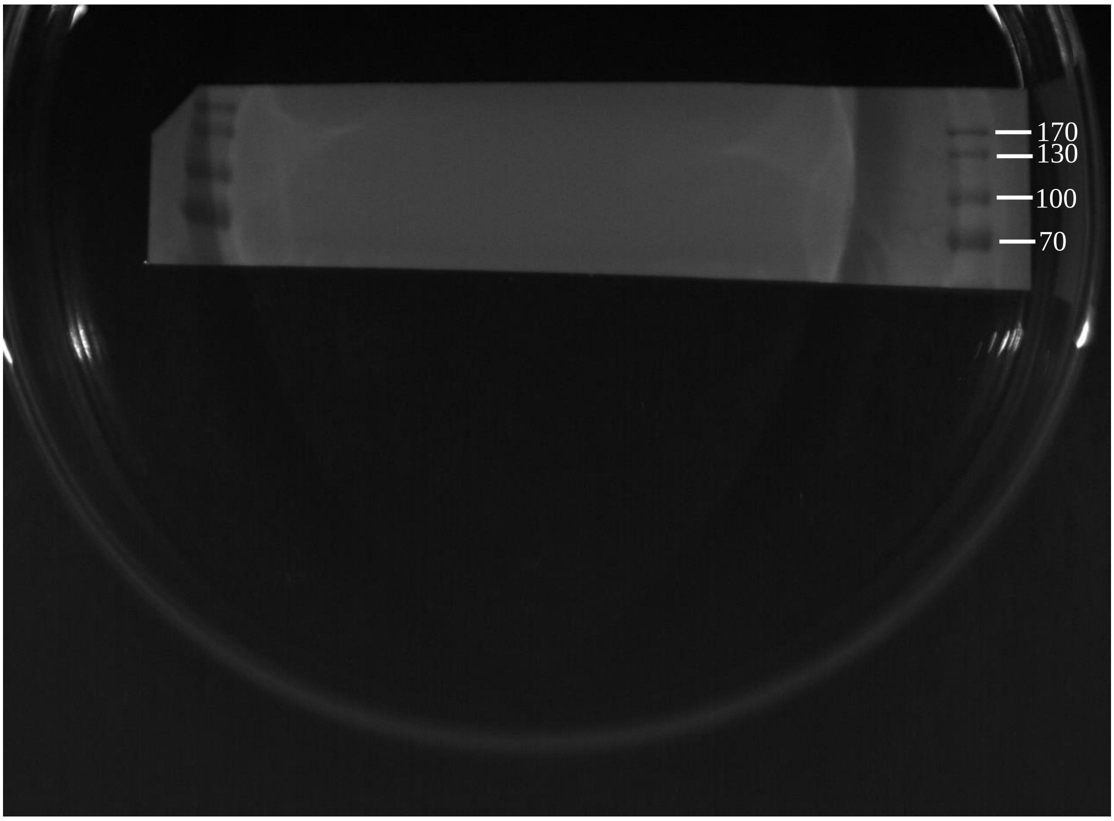

Full unedited blot for Figure 6a C83

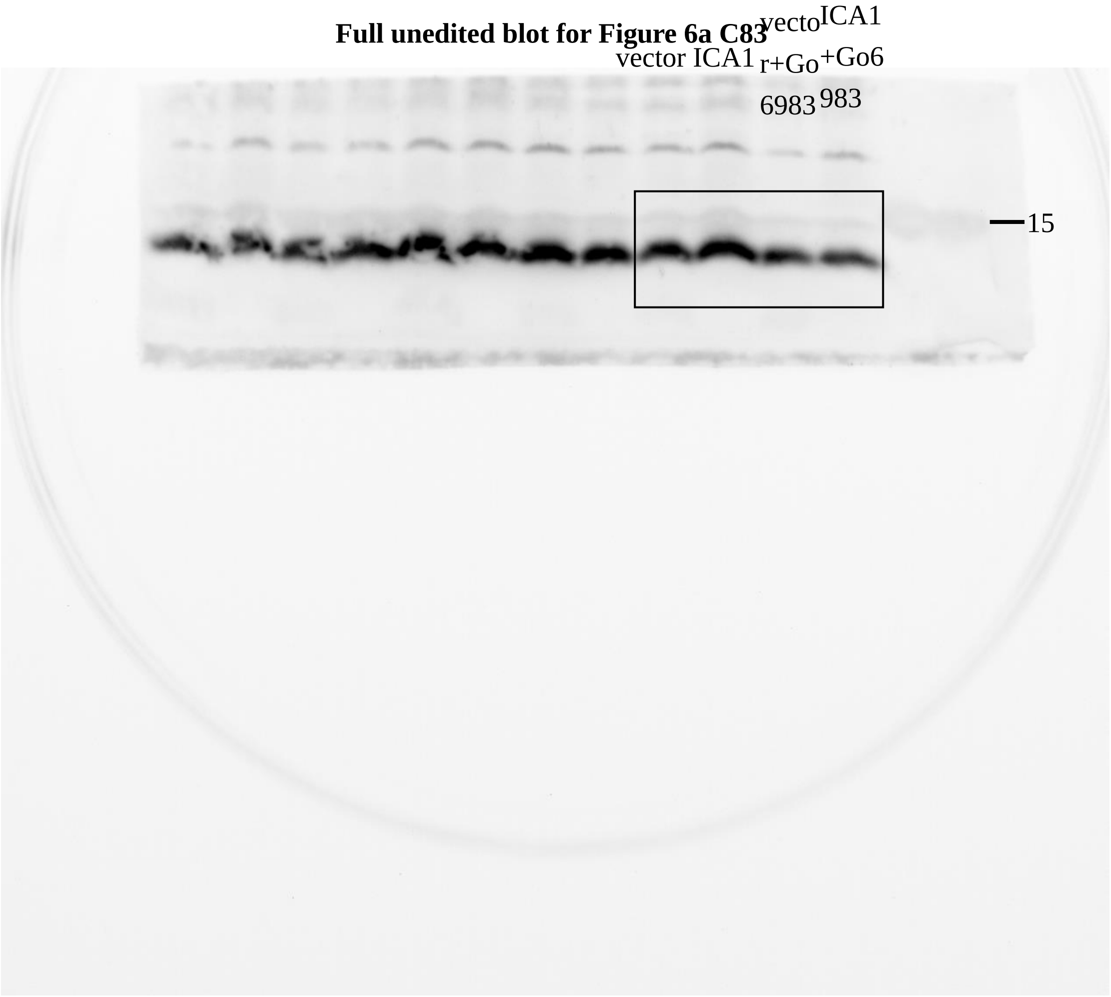

Full unedited blot for Figure 6a APP

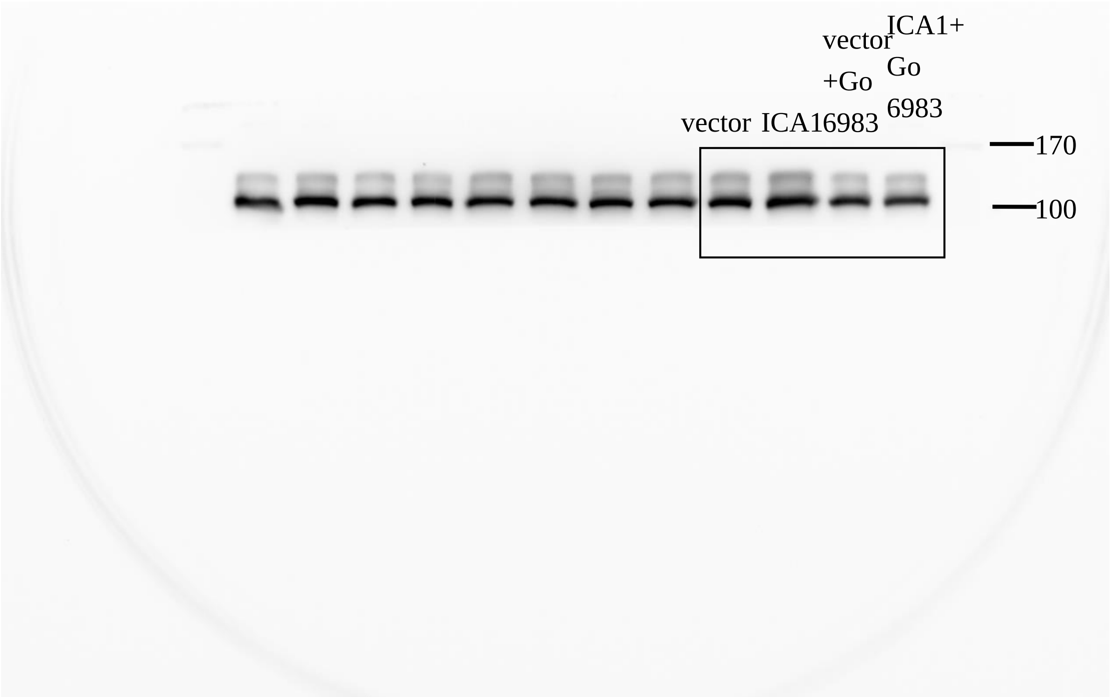

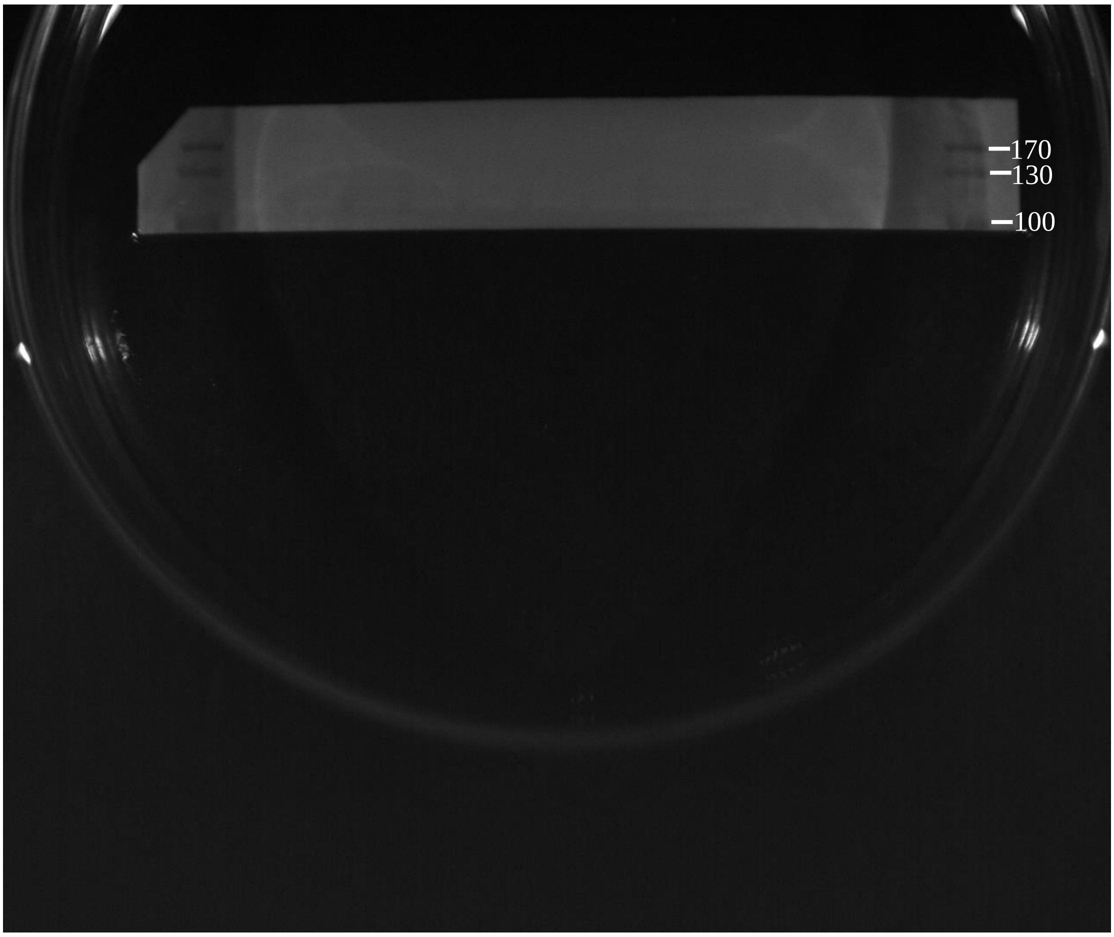

Full unedited blot for Figure 6a ADAM10

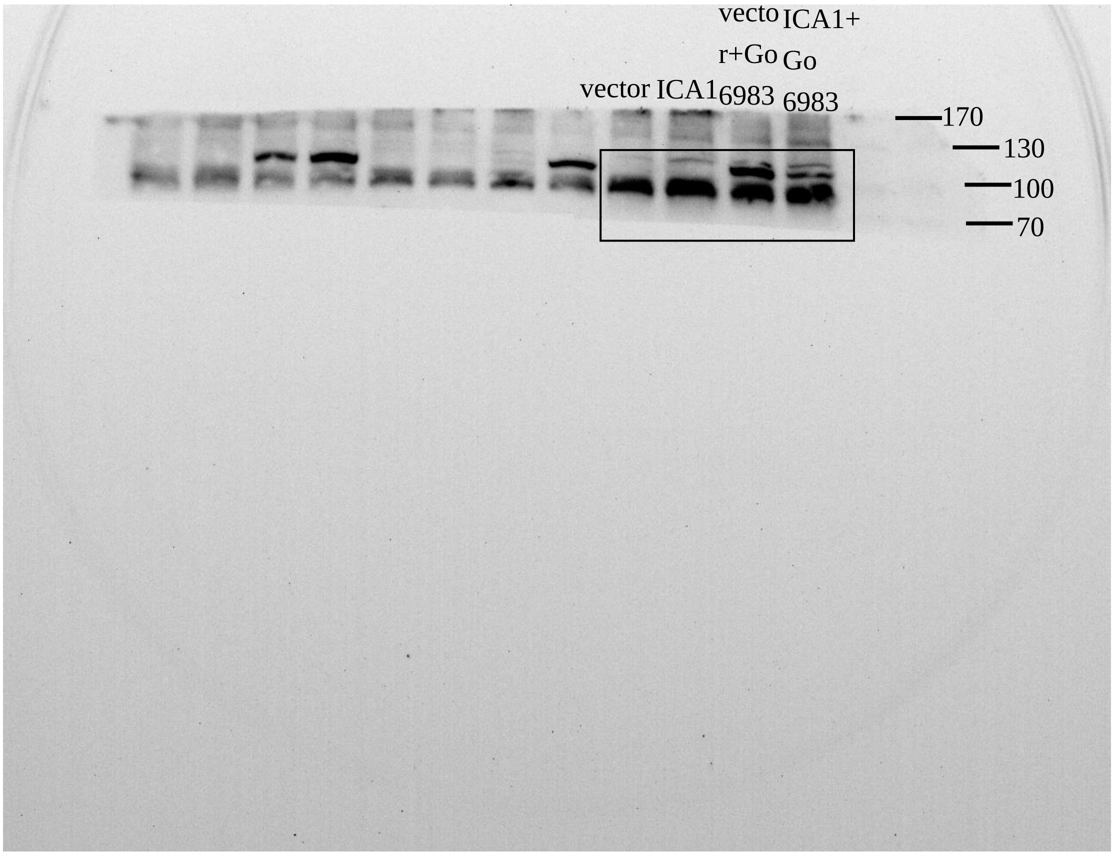

Full unedited blot for Figure 6a ADAM17

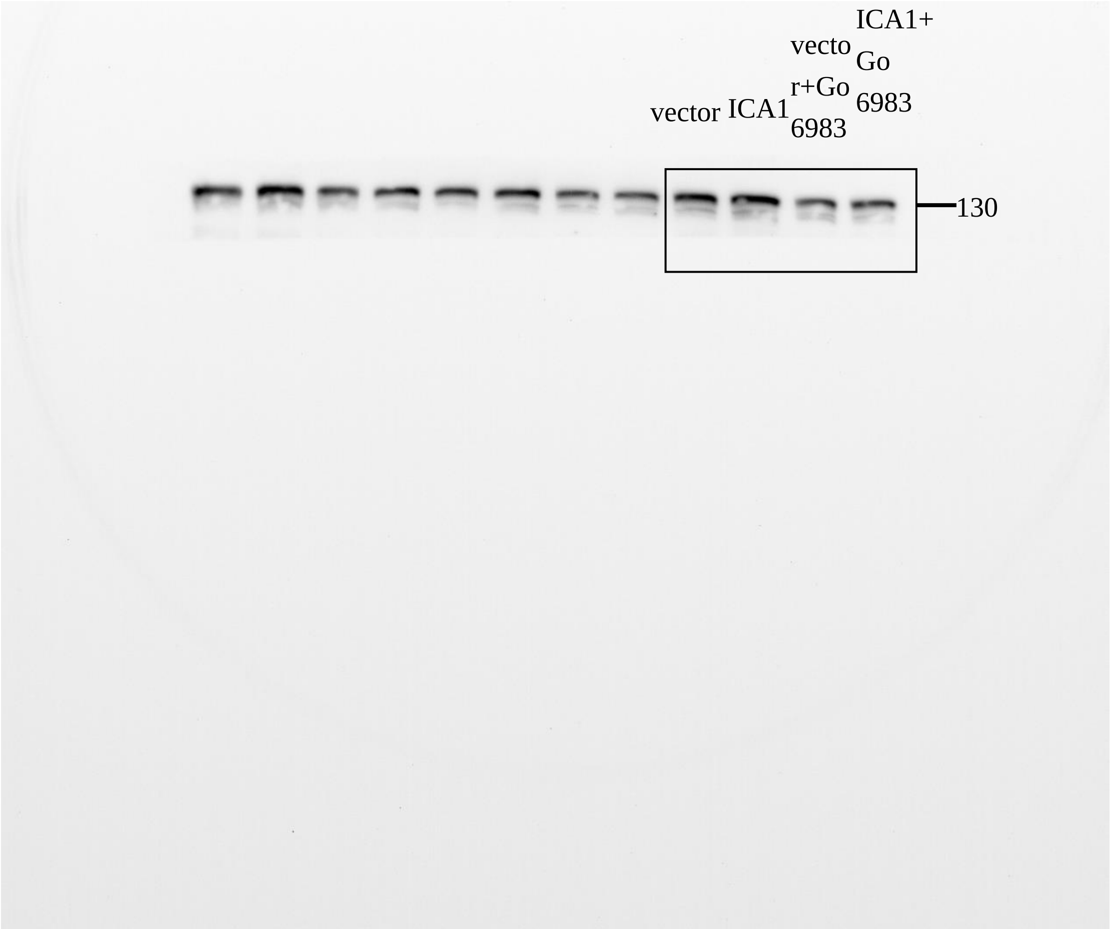

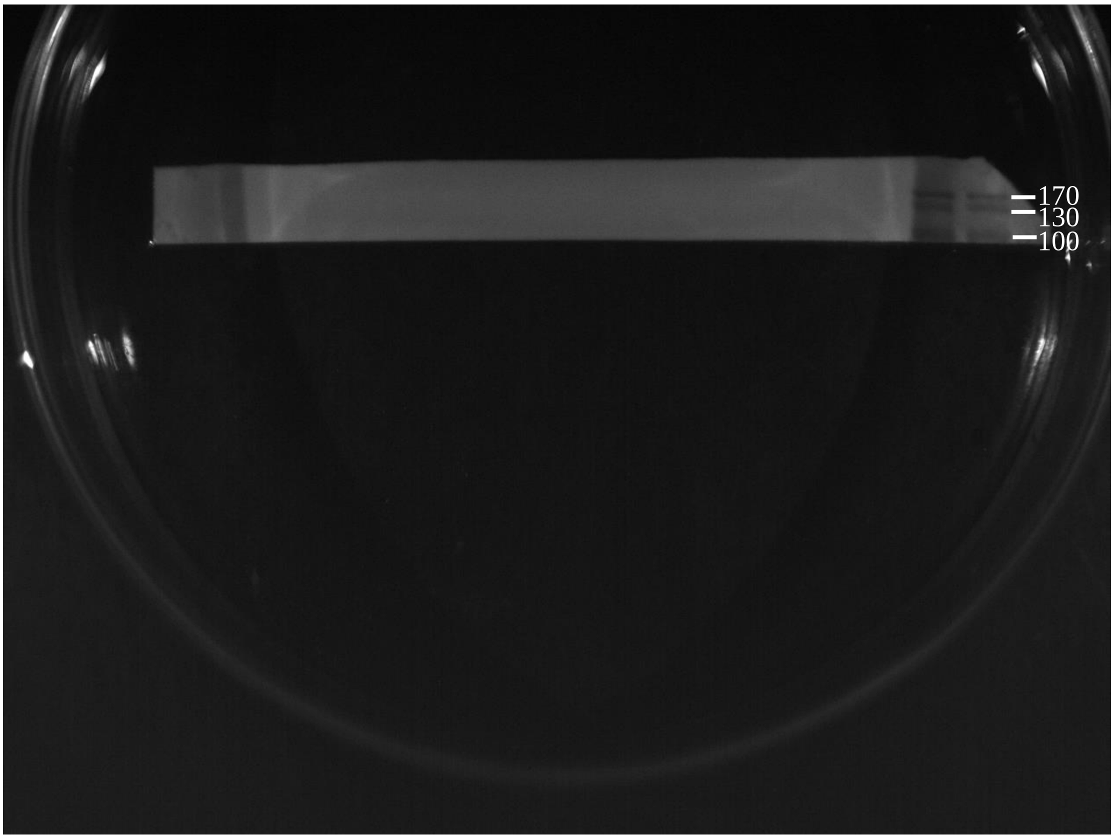

Full unedited blot for Figure 6a GAPDH

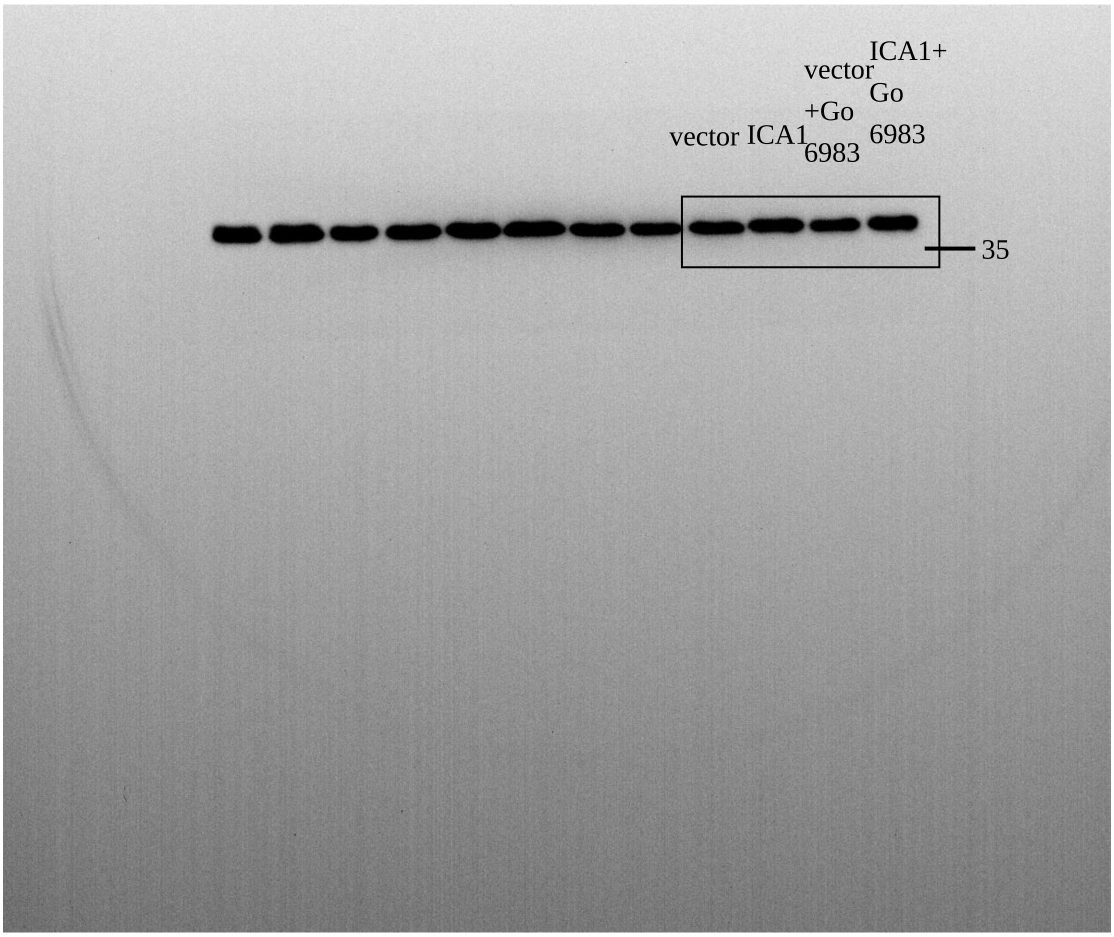

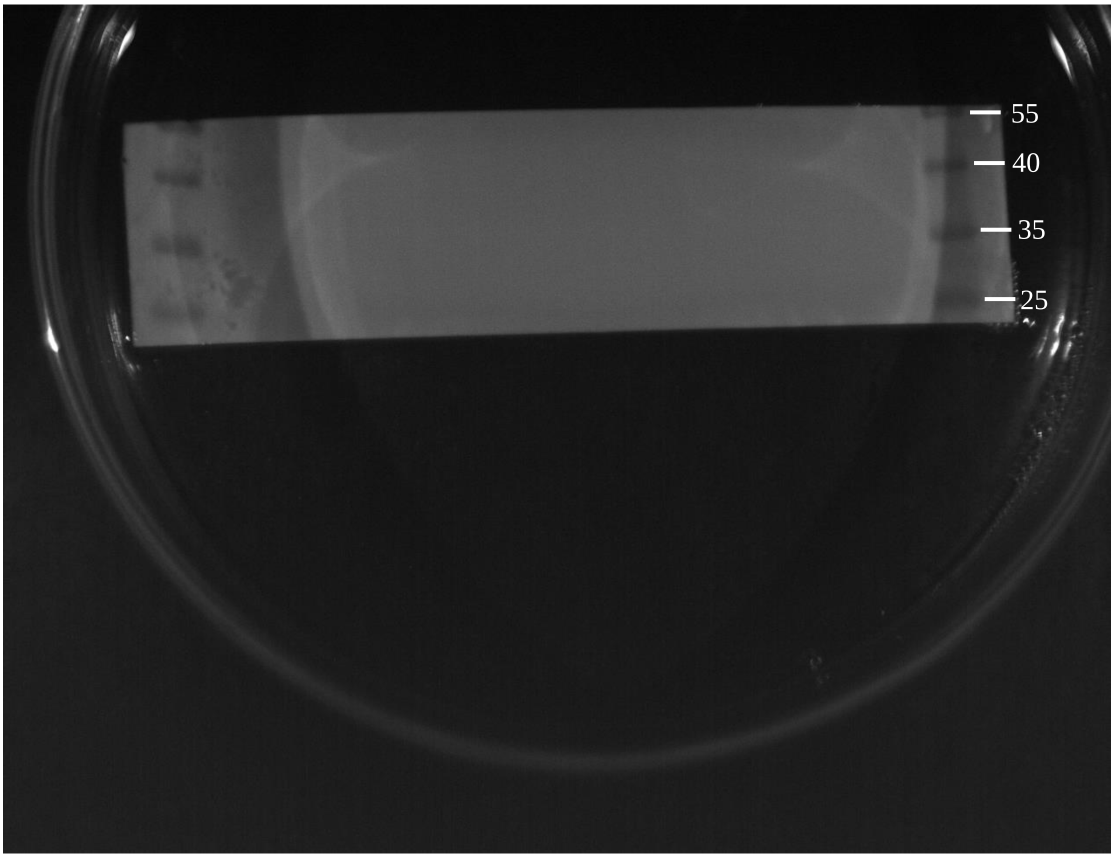

Full unedited blot for supplemental Figure 1a ICA1

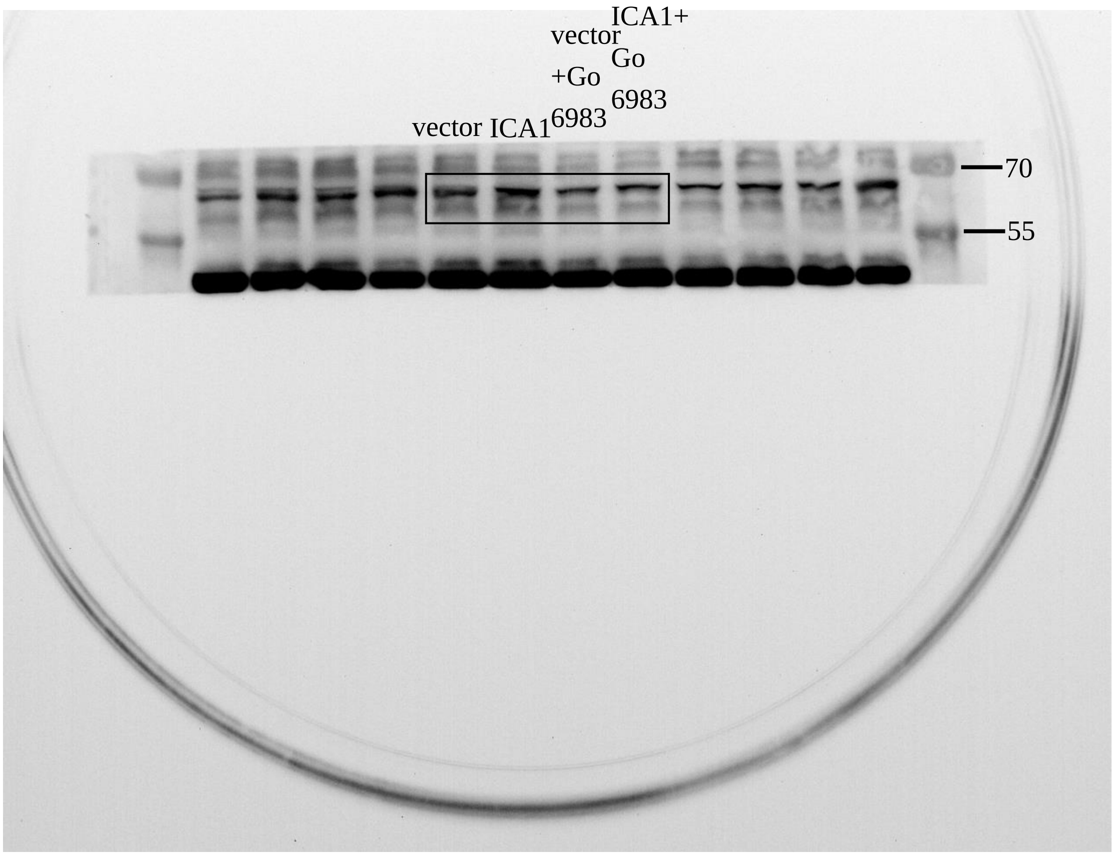

Full unedited blot for supplemental Figure 1a PICK1

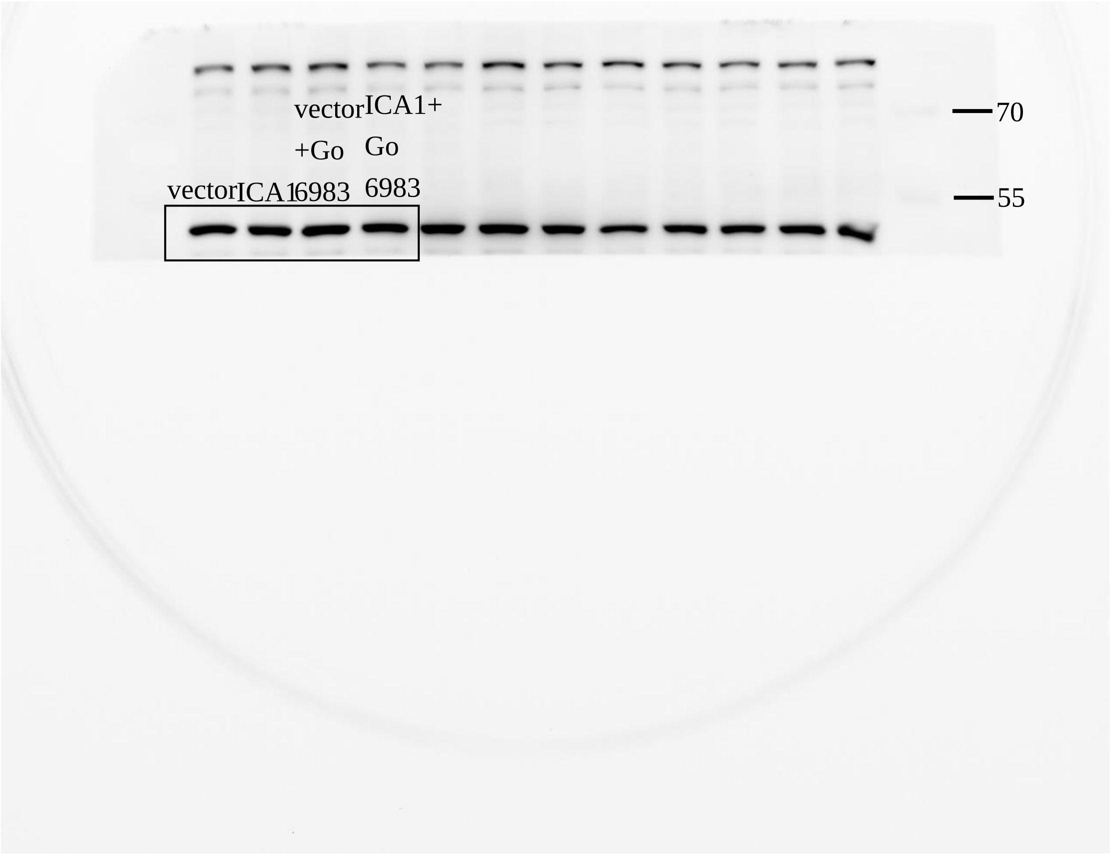

Full unedited blot for supplemental Figure 1a PKC alpha

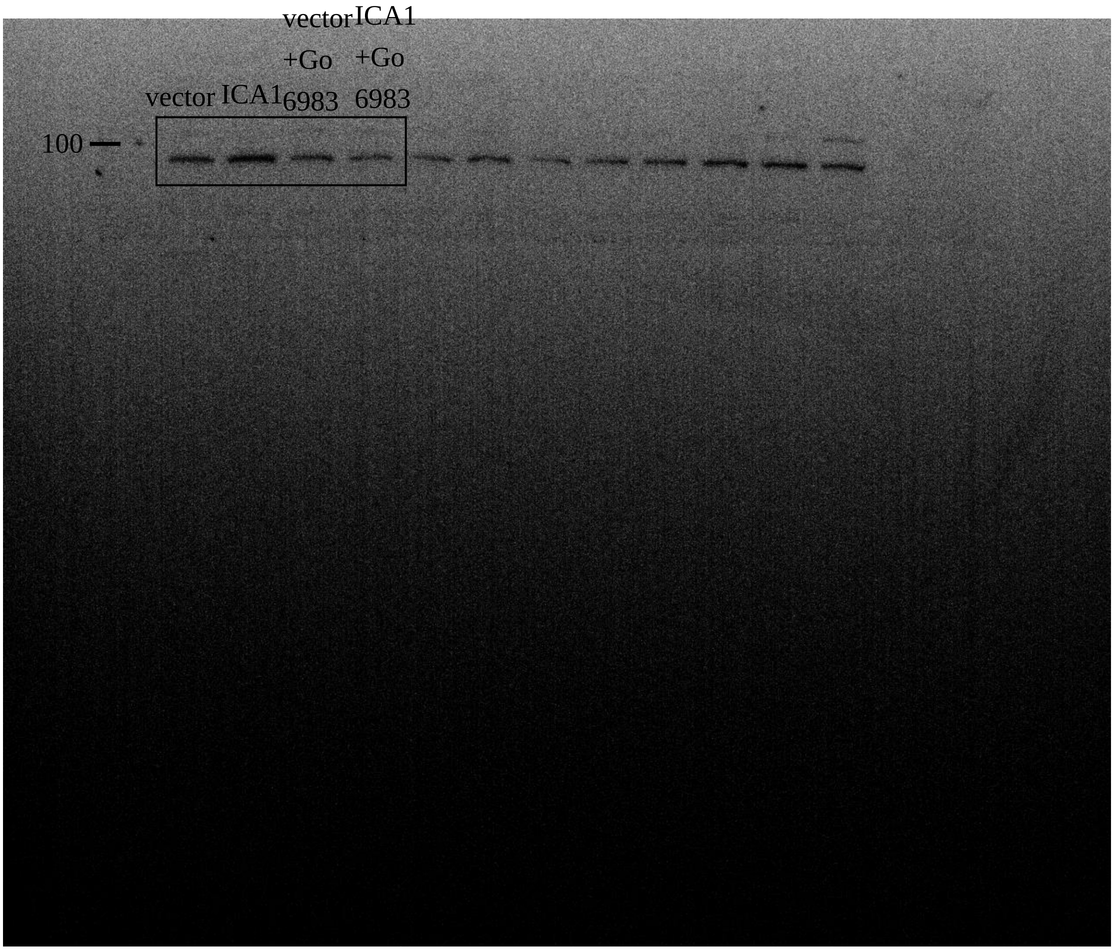

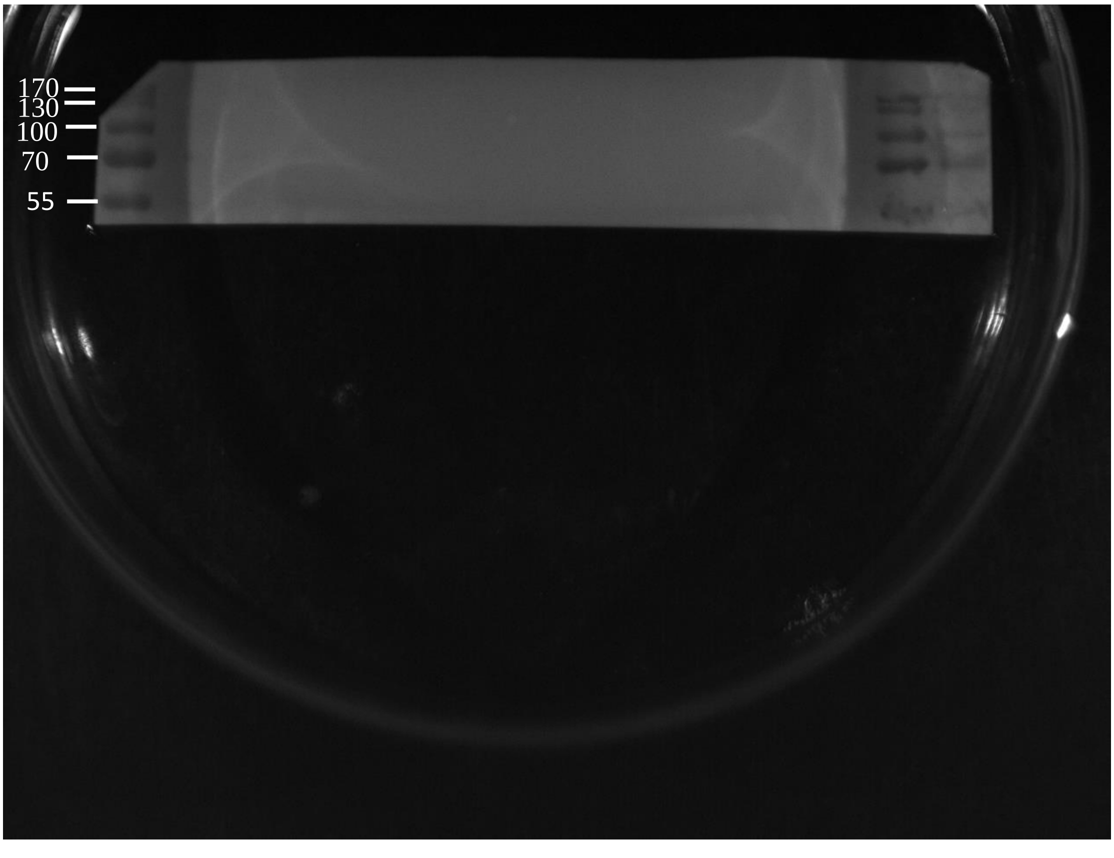

Full unedited blot for supplemental Figure 1a p-PKC alpha

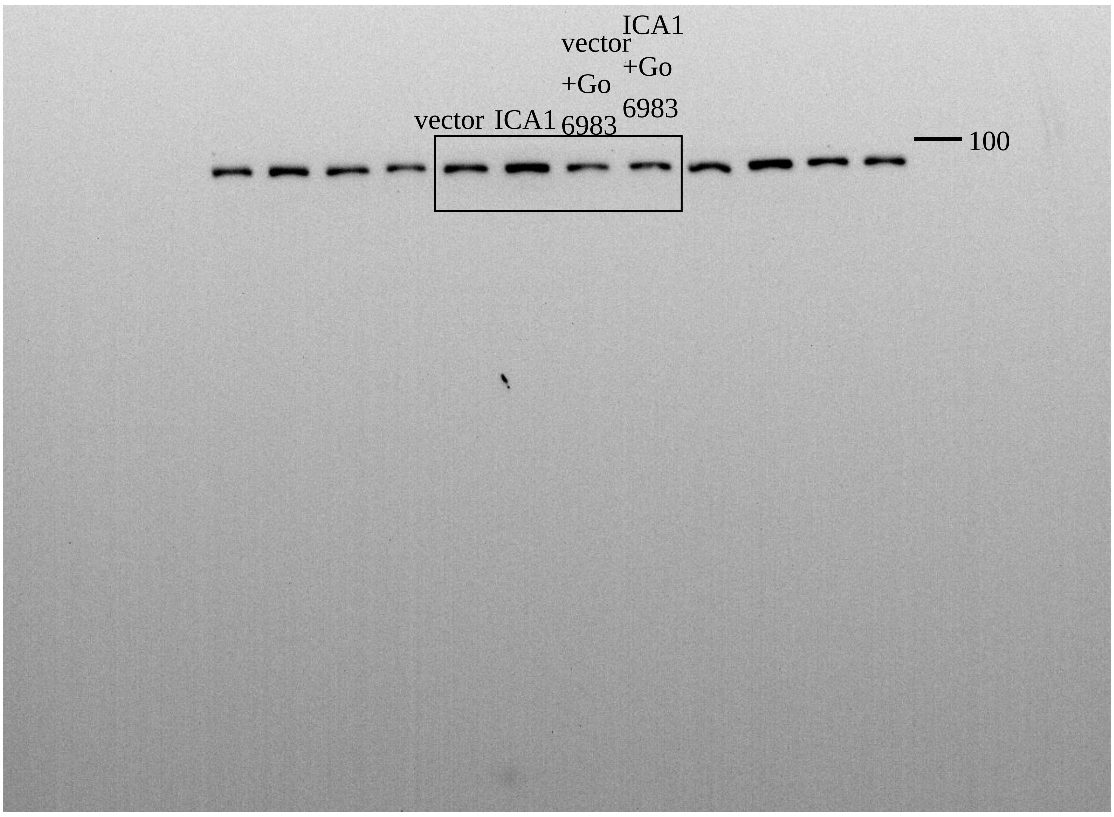

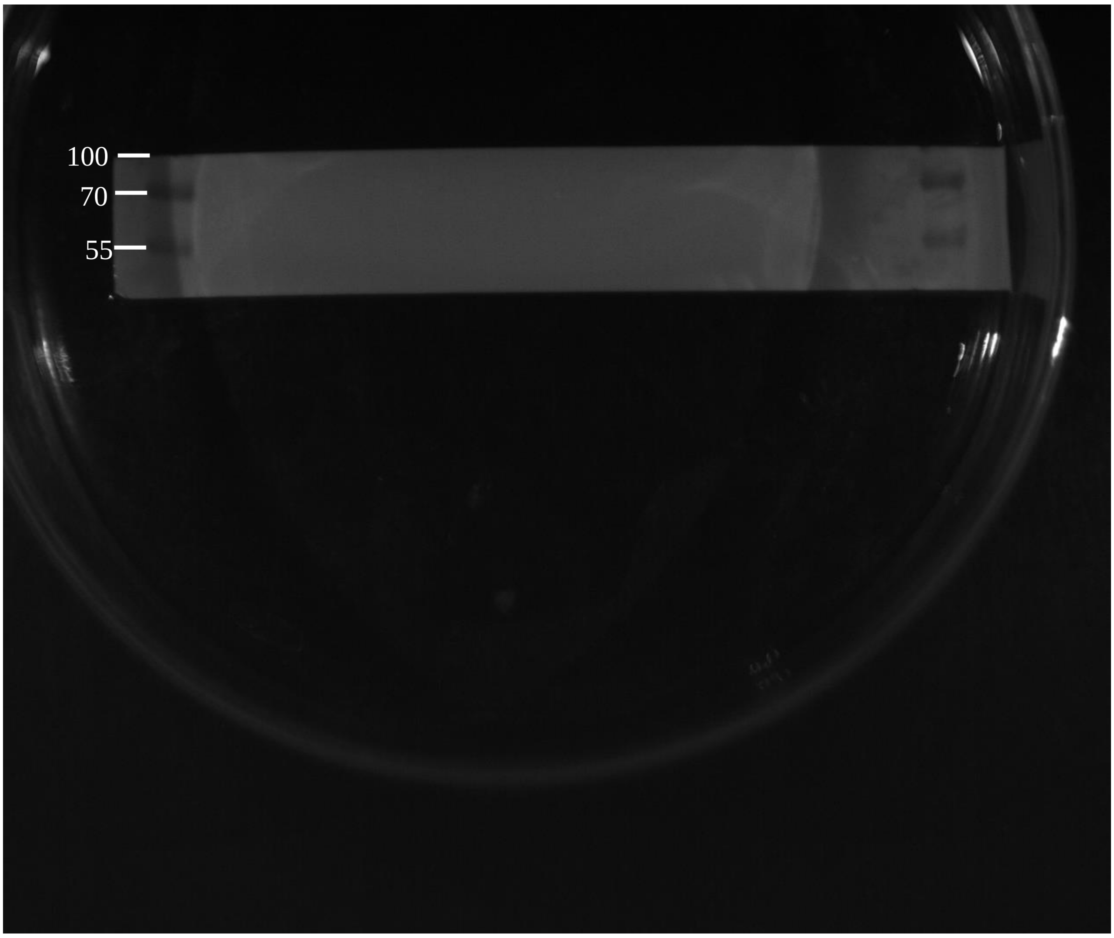

Full unedited blot for supplemental Figure 1a C89 and C99

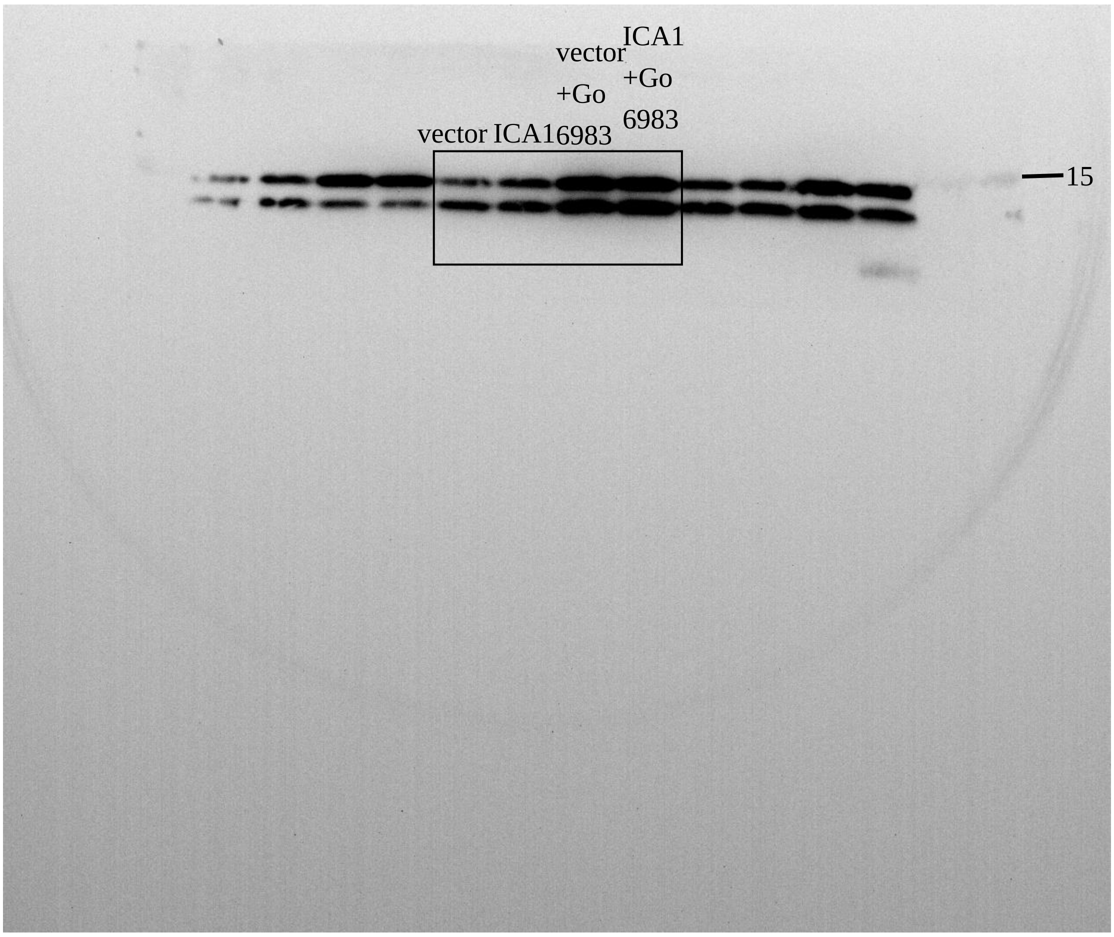

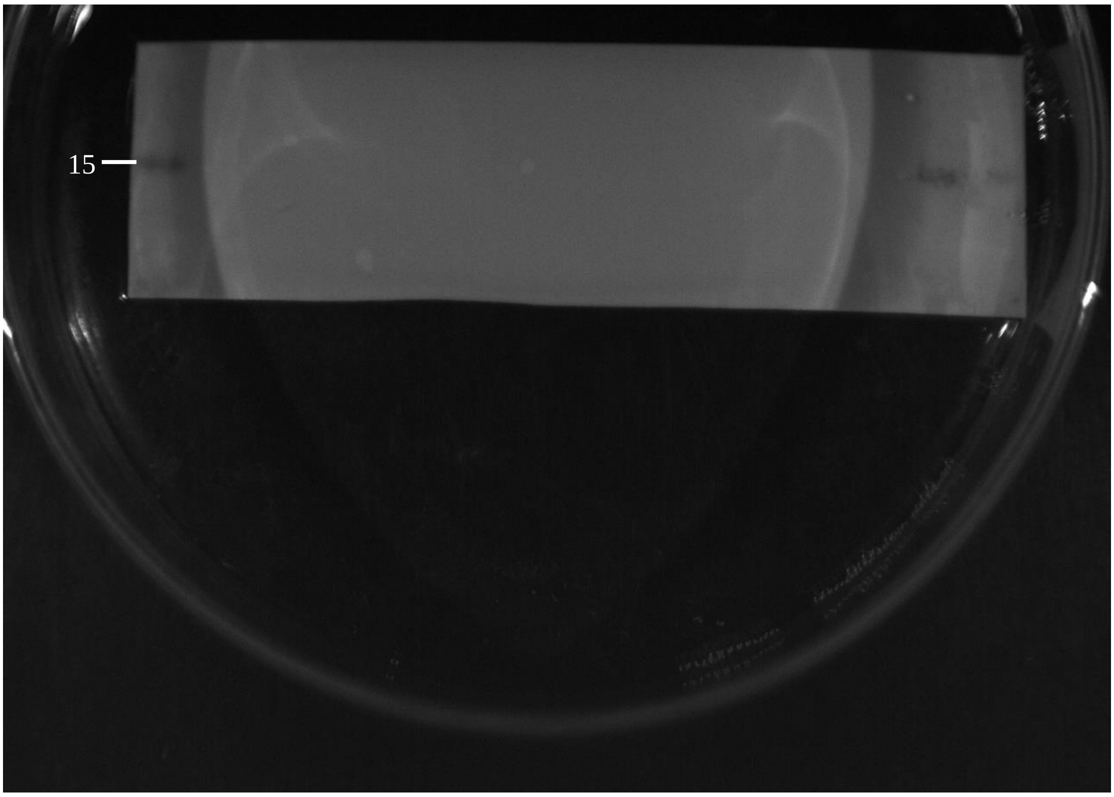

Full unedited blot for supplemental Figure 1a APP

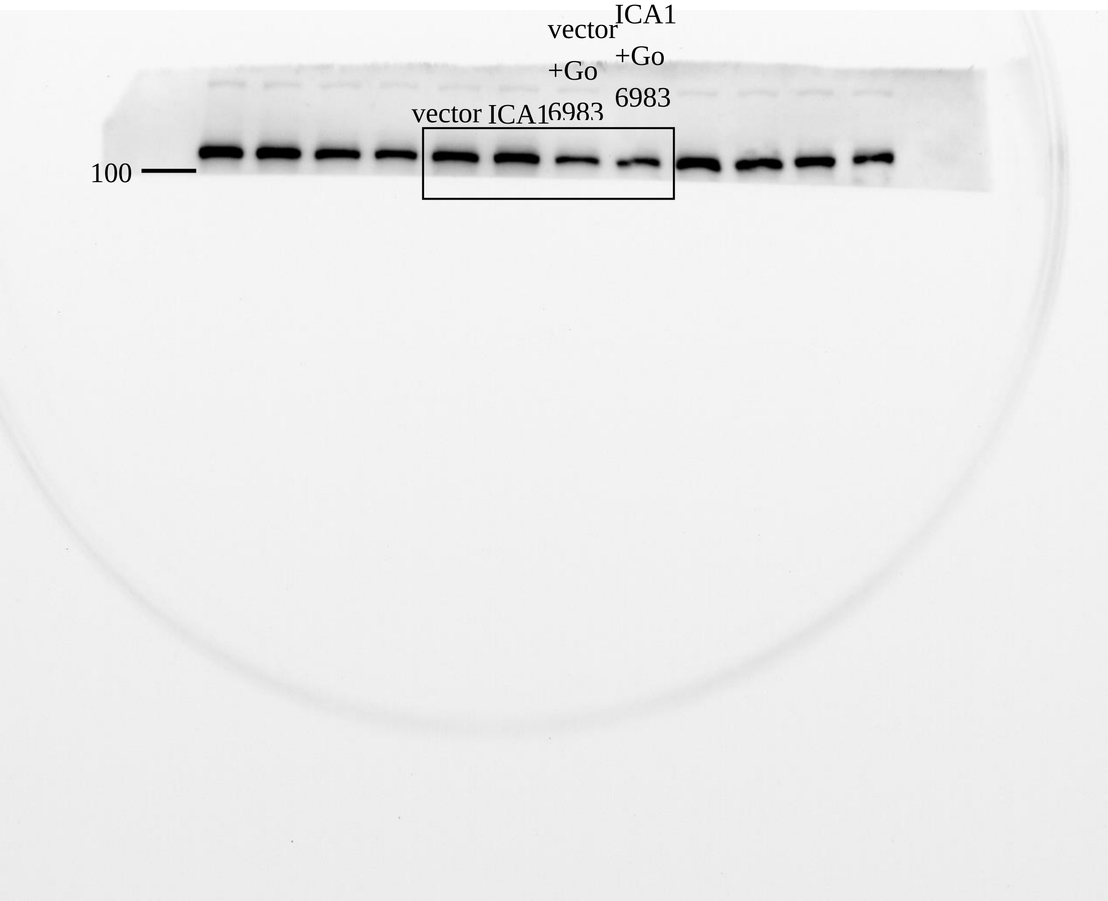

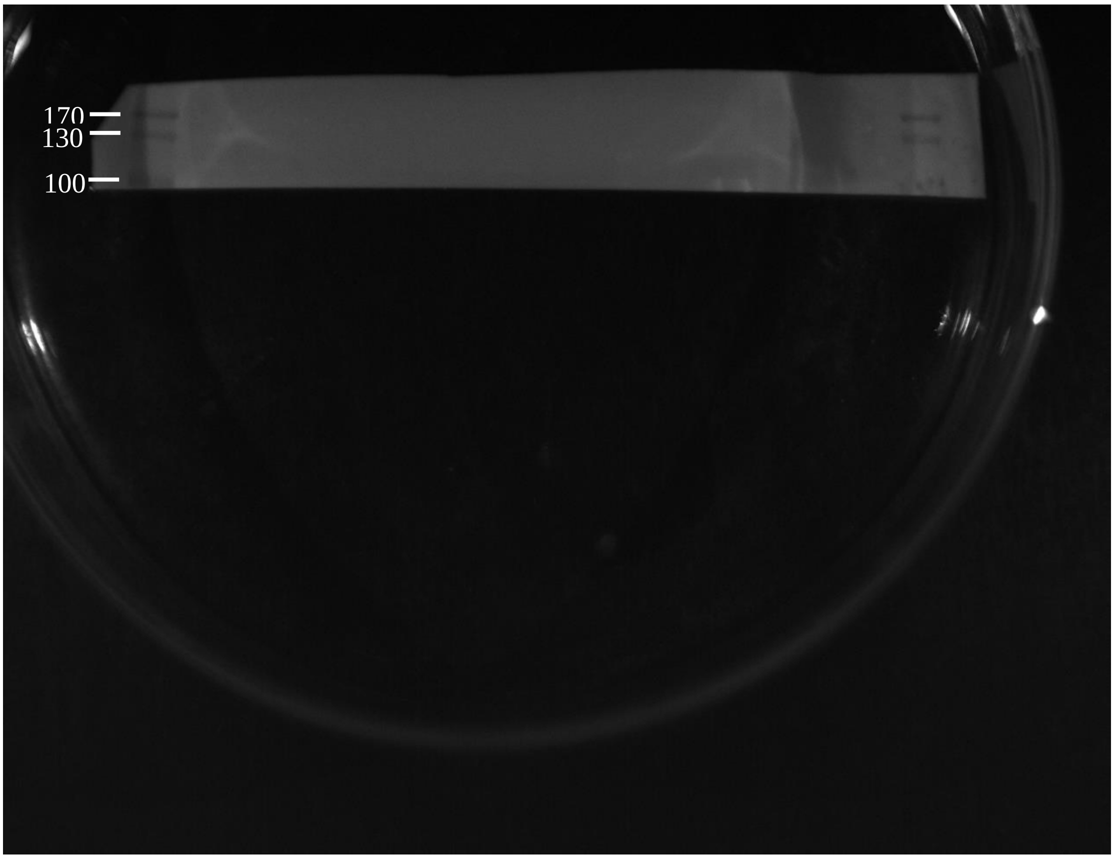

Full unedited blot for supplemental Figure 1a ADAM10

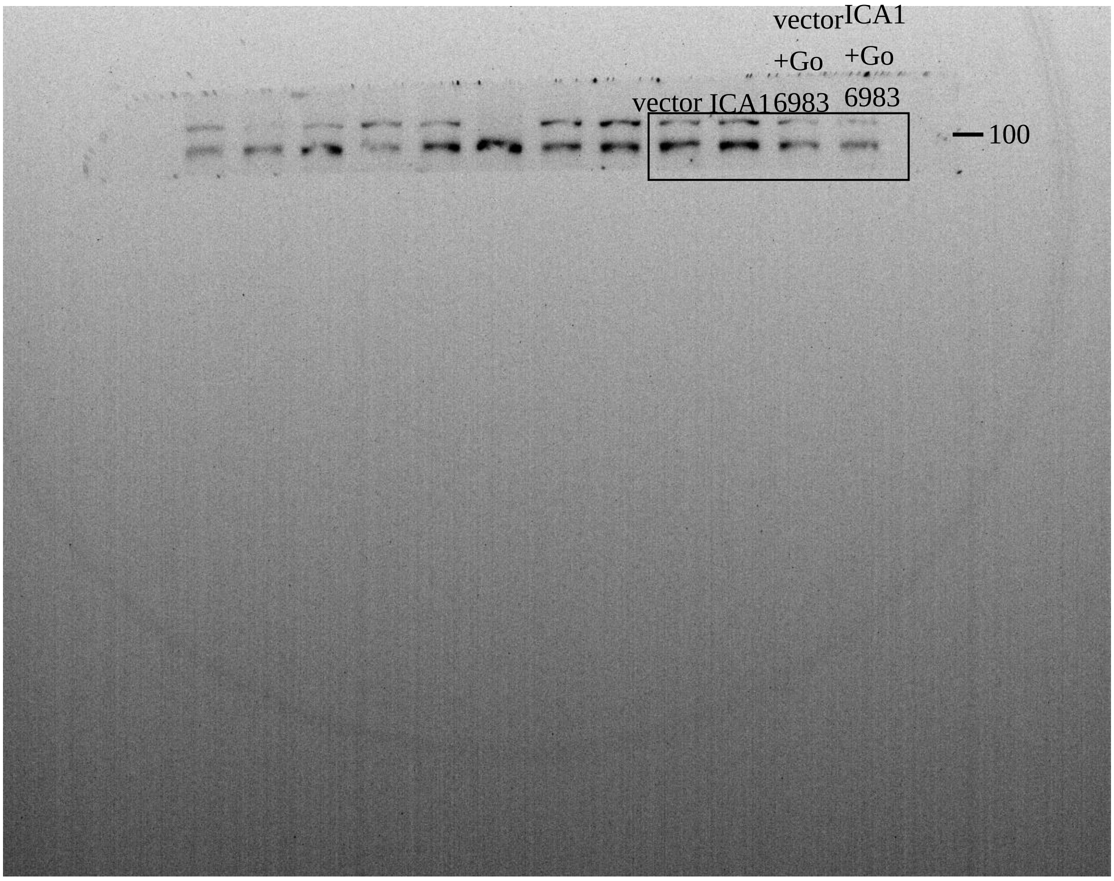

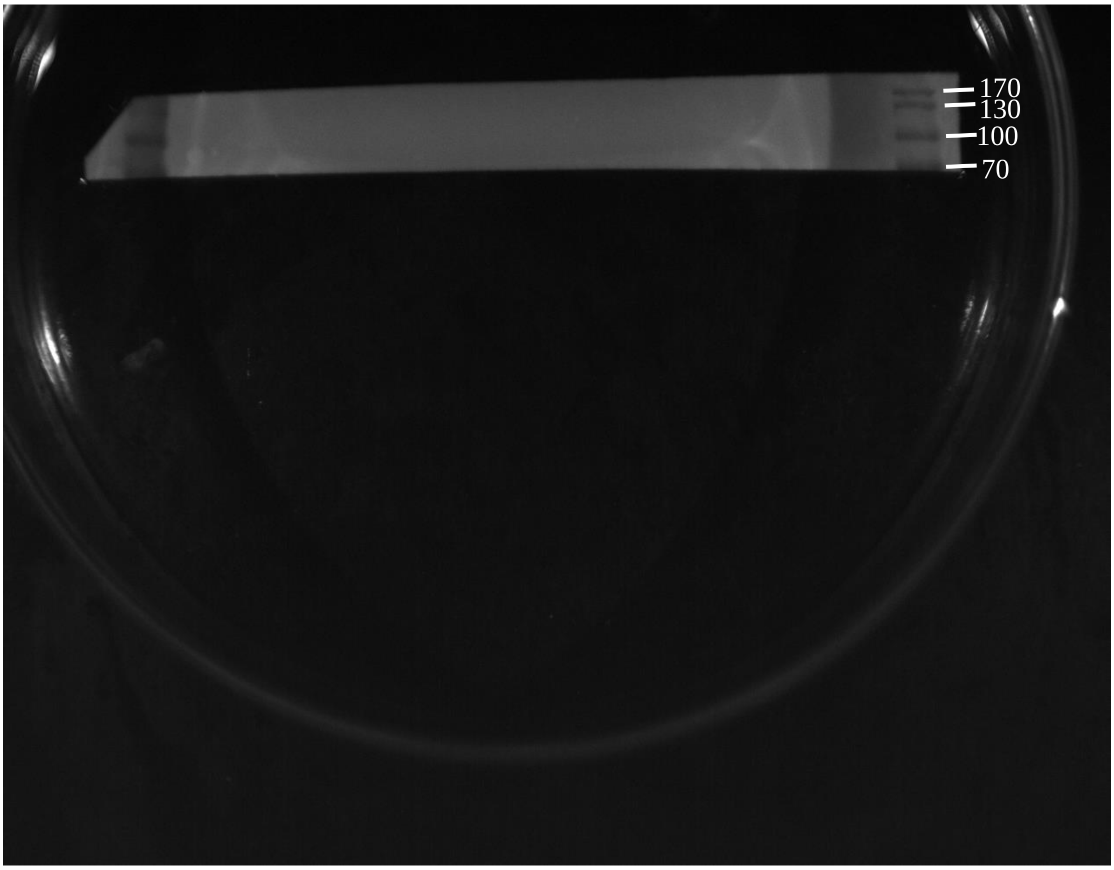

Full unedited blot for supplemental Figure 1a ADAM17

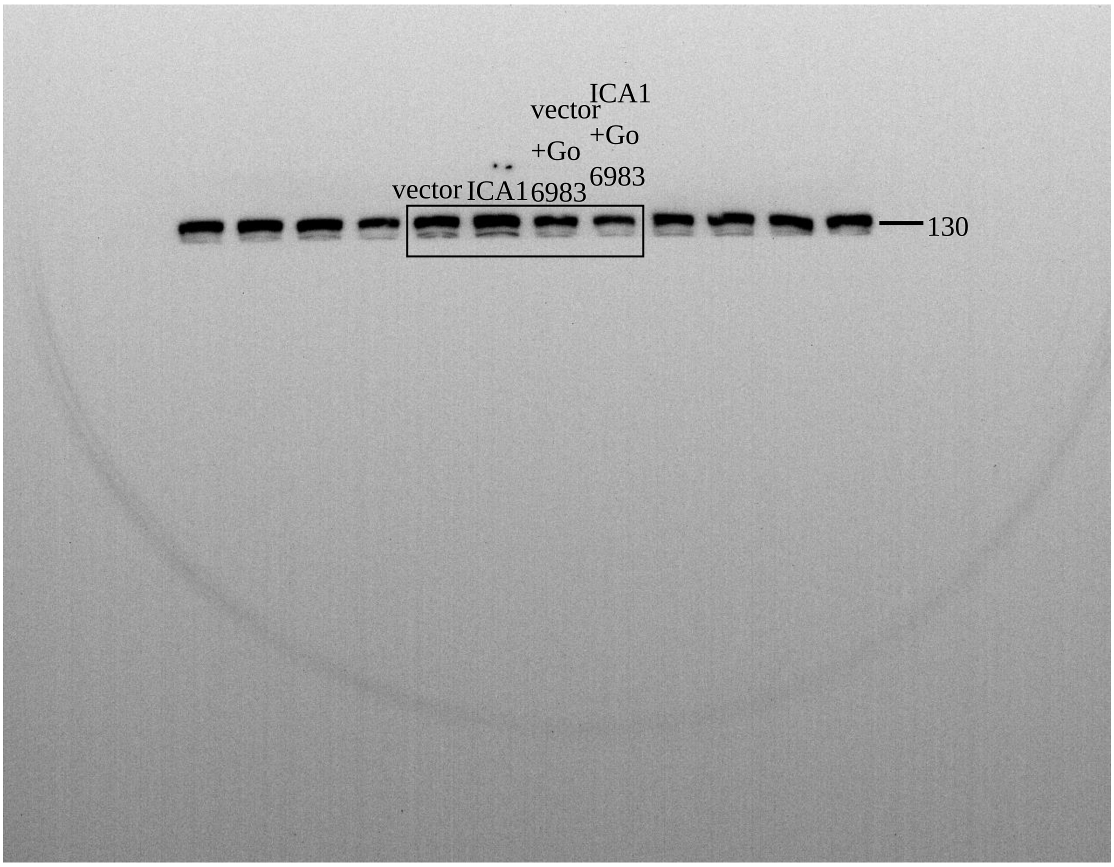

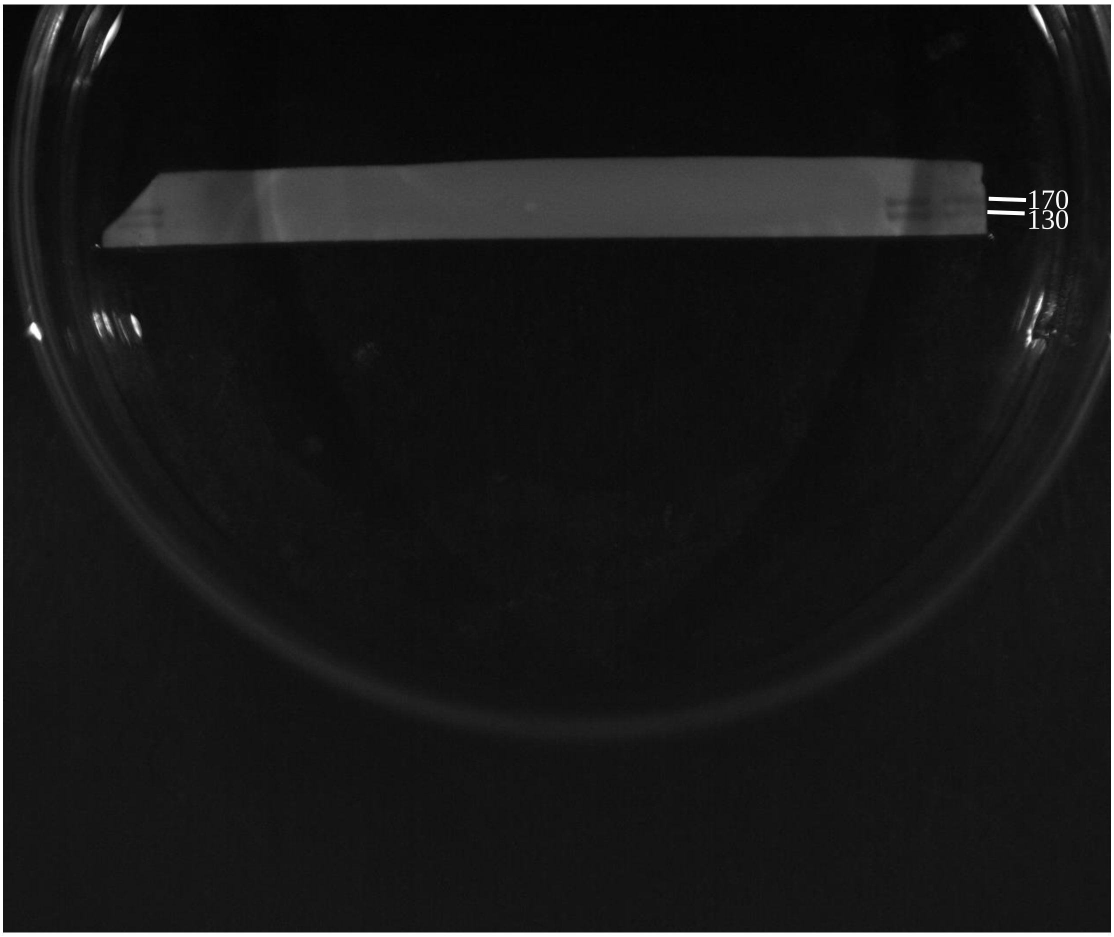

Full unedited blot for supplemental Figure 1a GAPDH

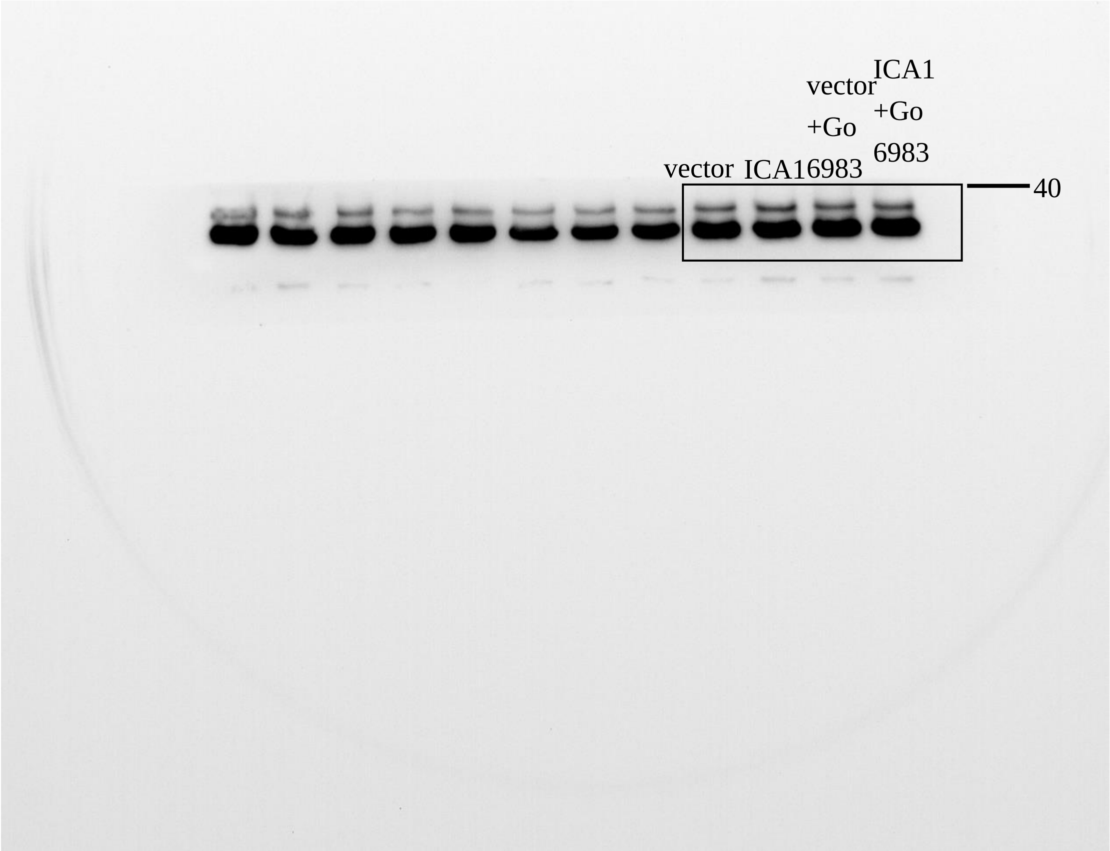

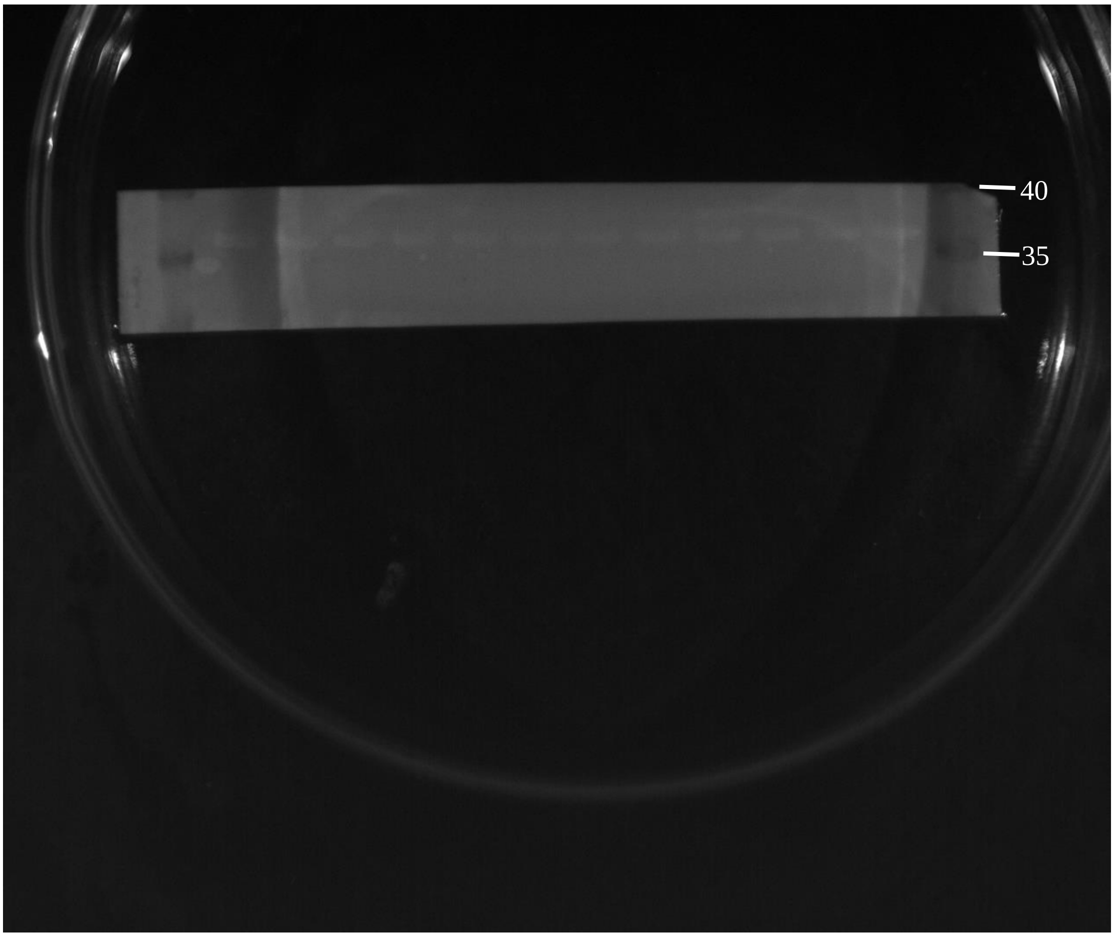

Full unedited blot for supplemental Figure 2a ICA1

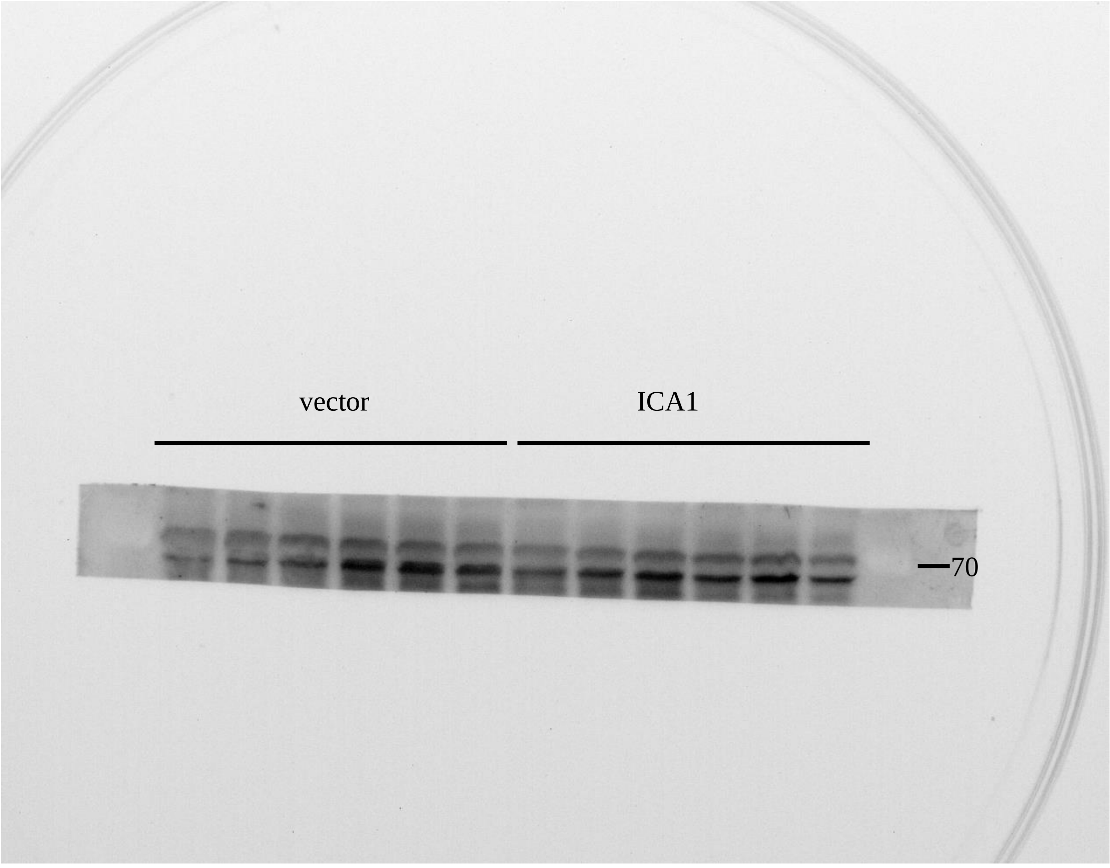

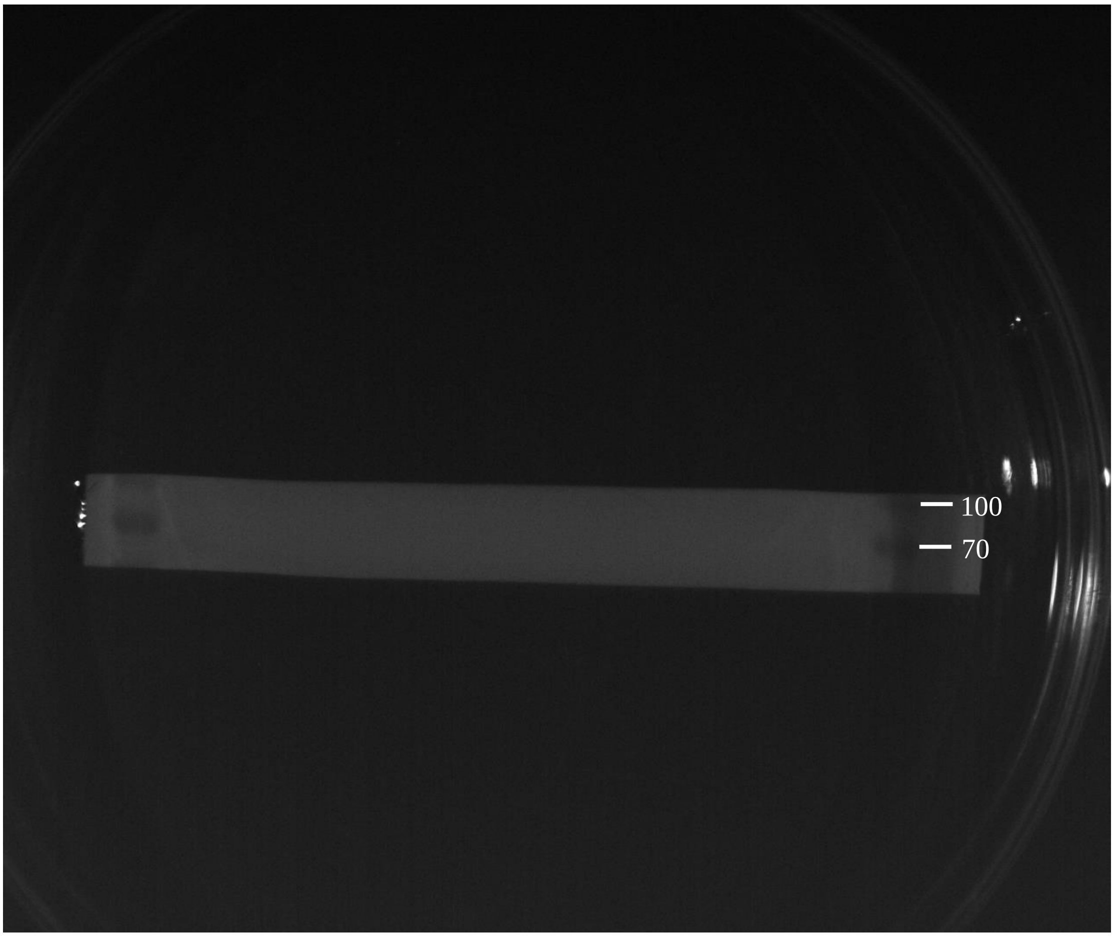

Full unedited blot for supplemental Figure 2a C89 and C99

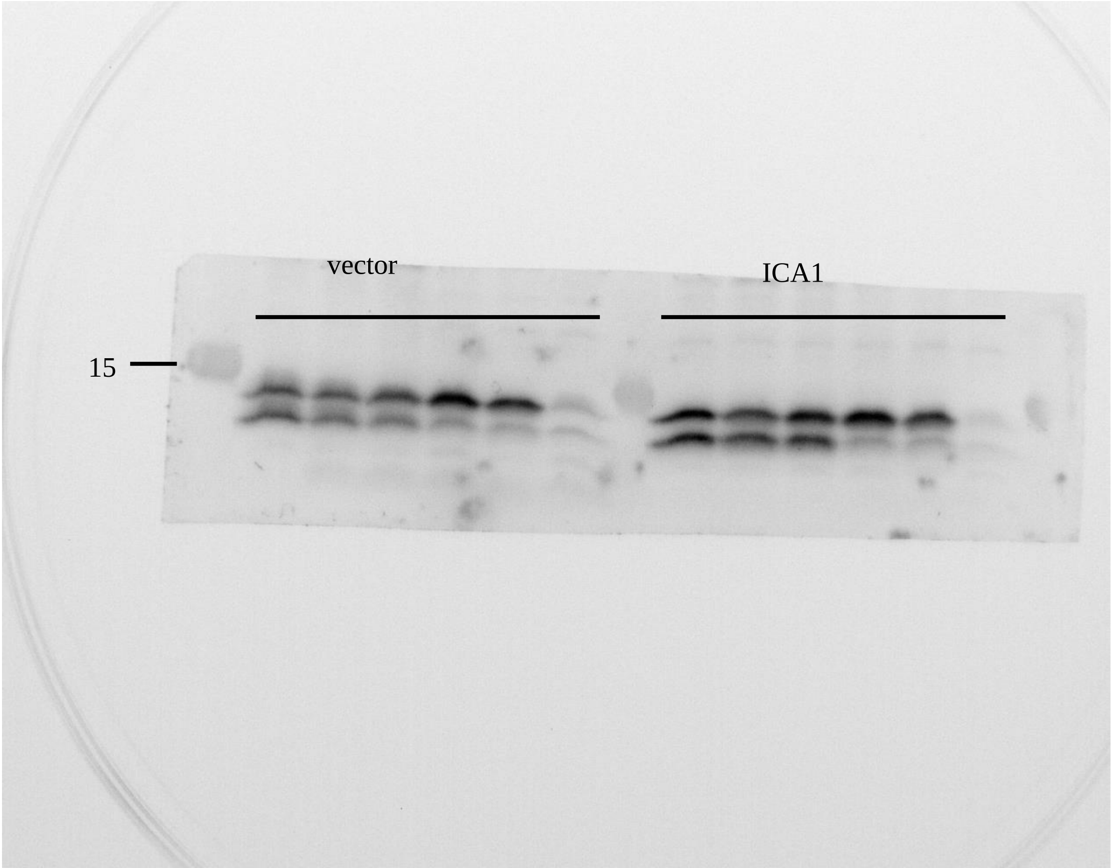

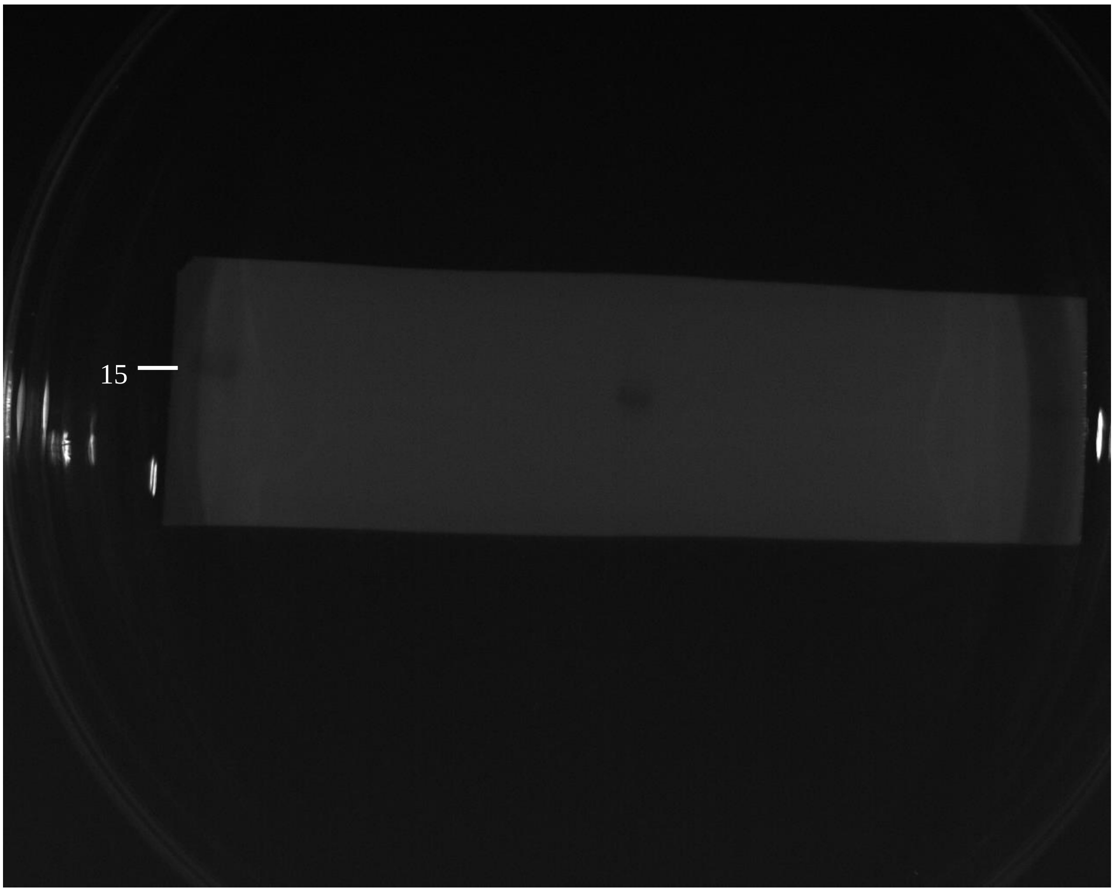

Full unedited blot for supplemental Figure 2a APP

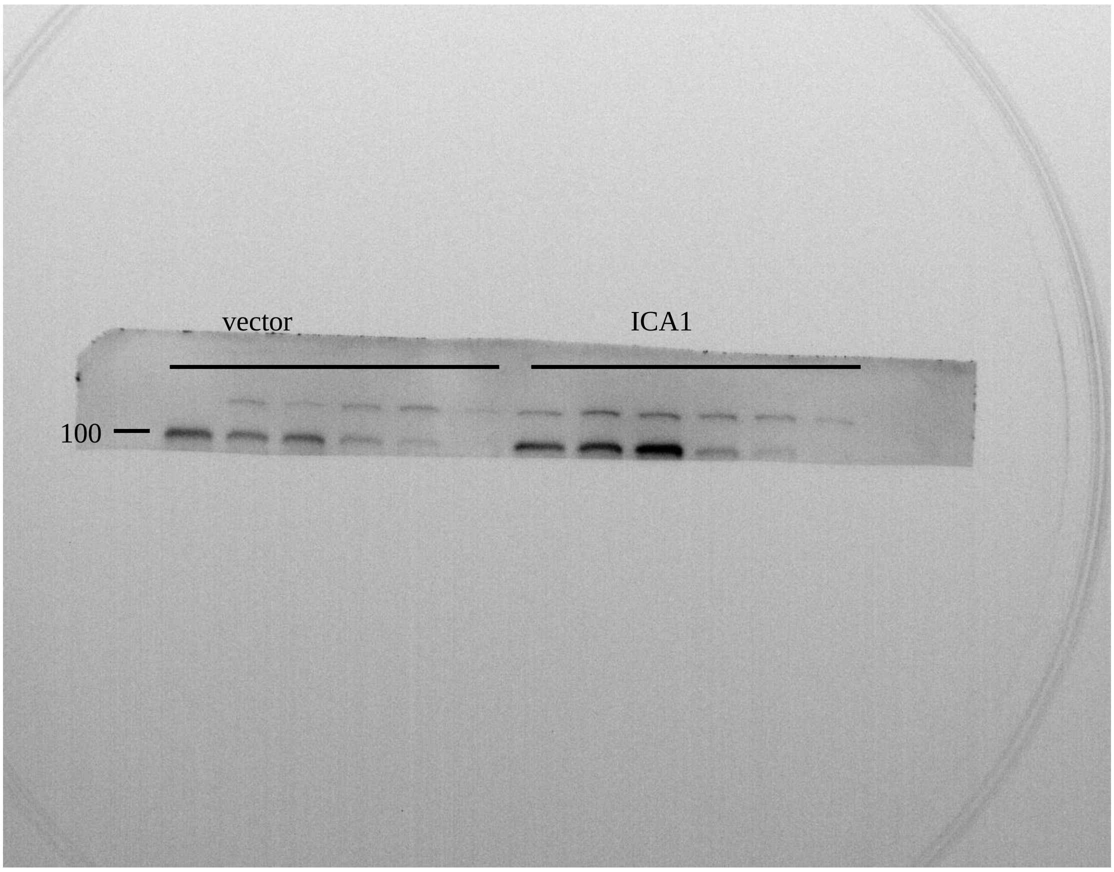

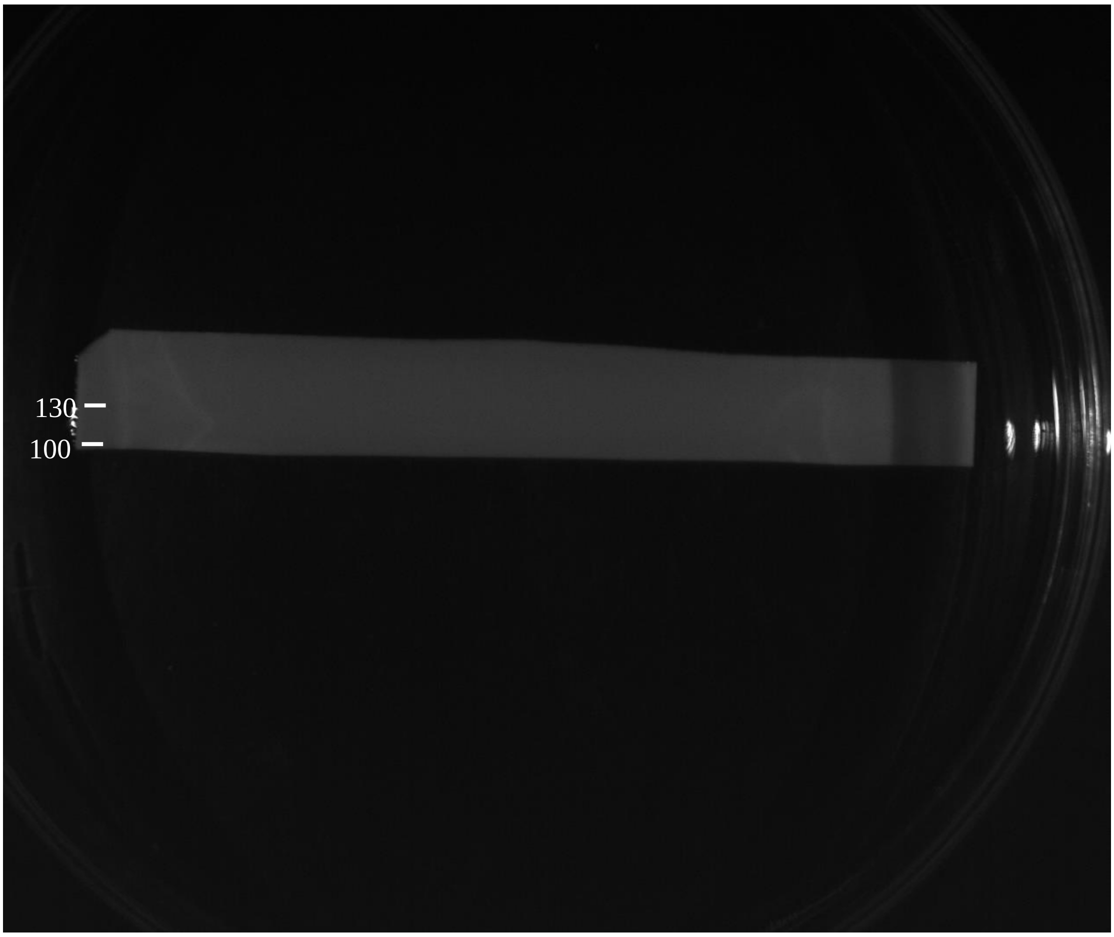

Full unedited blot for supplemental Figure 2a ADAM17

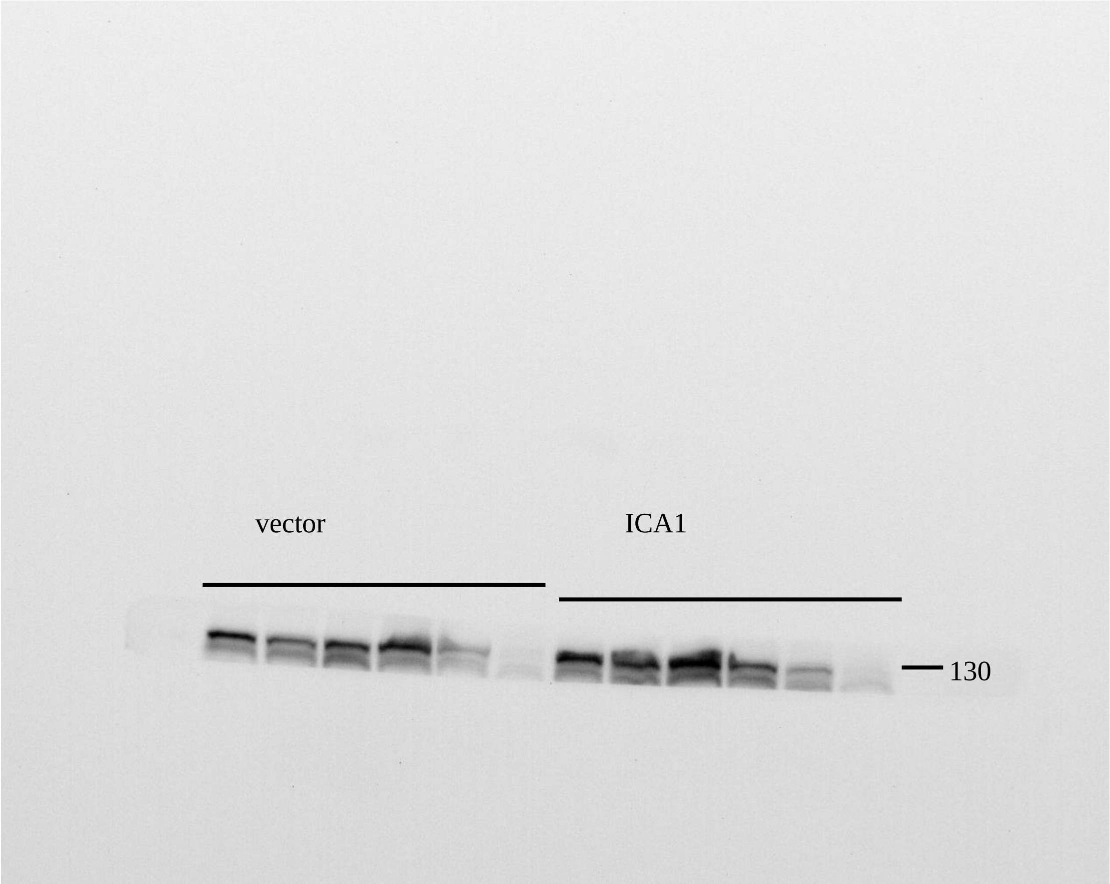

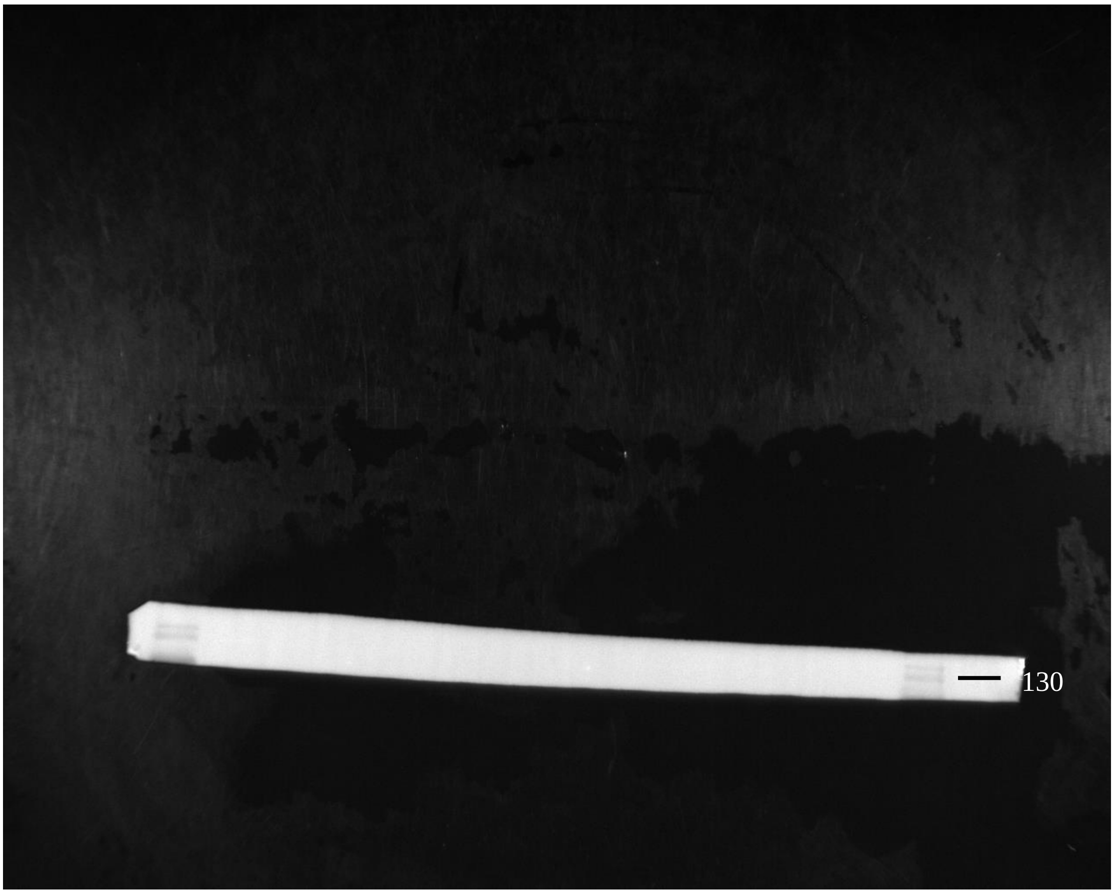

Full unedited blot for supplemental Figure 2a BACE1

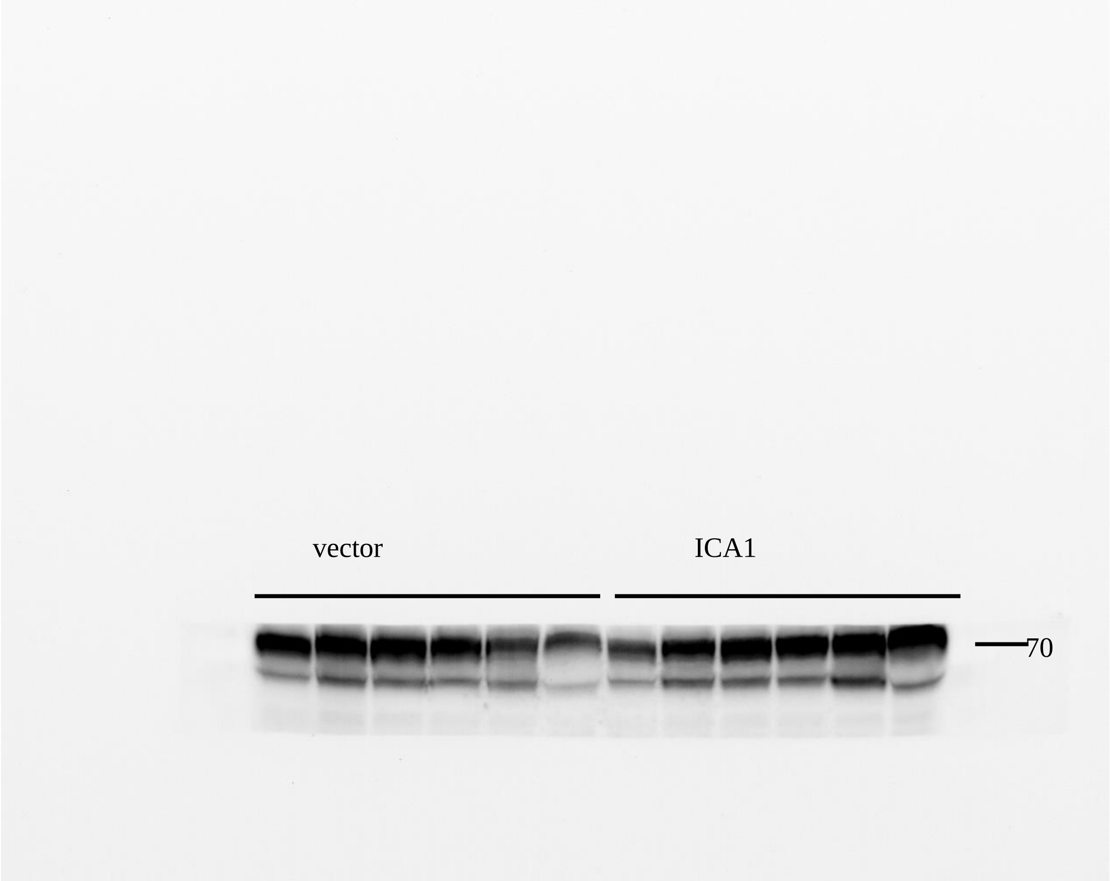

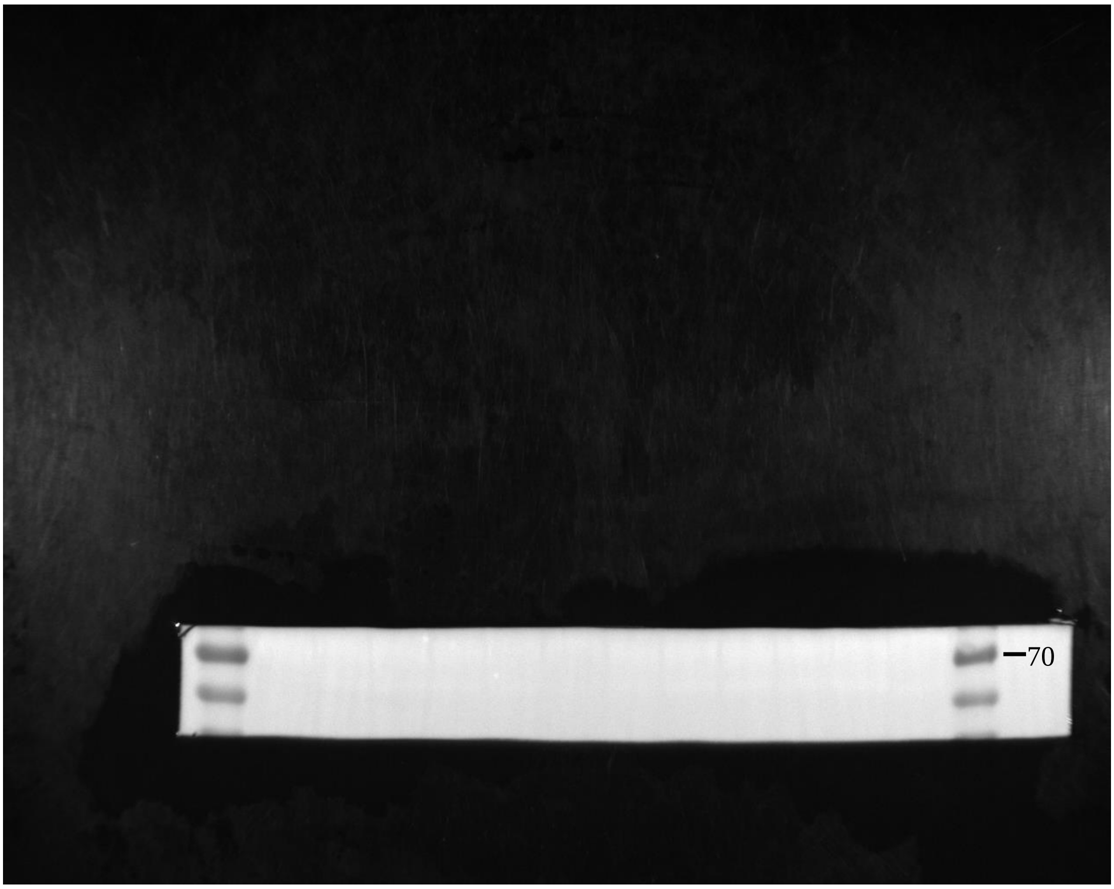

Full unedited blot for supplemental Figure 2a PS1

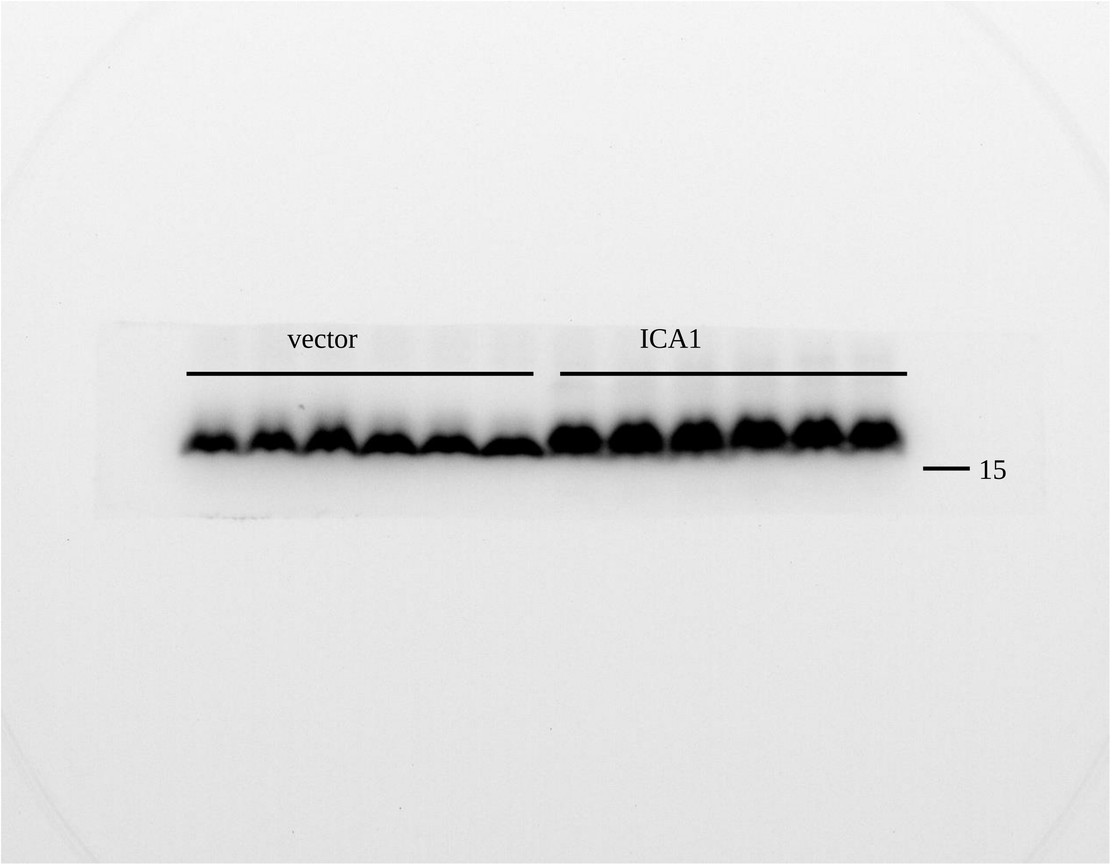

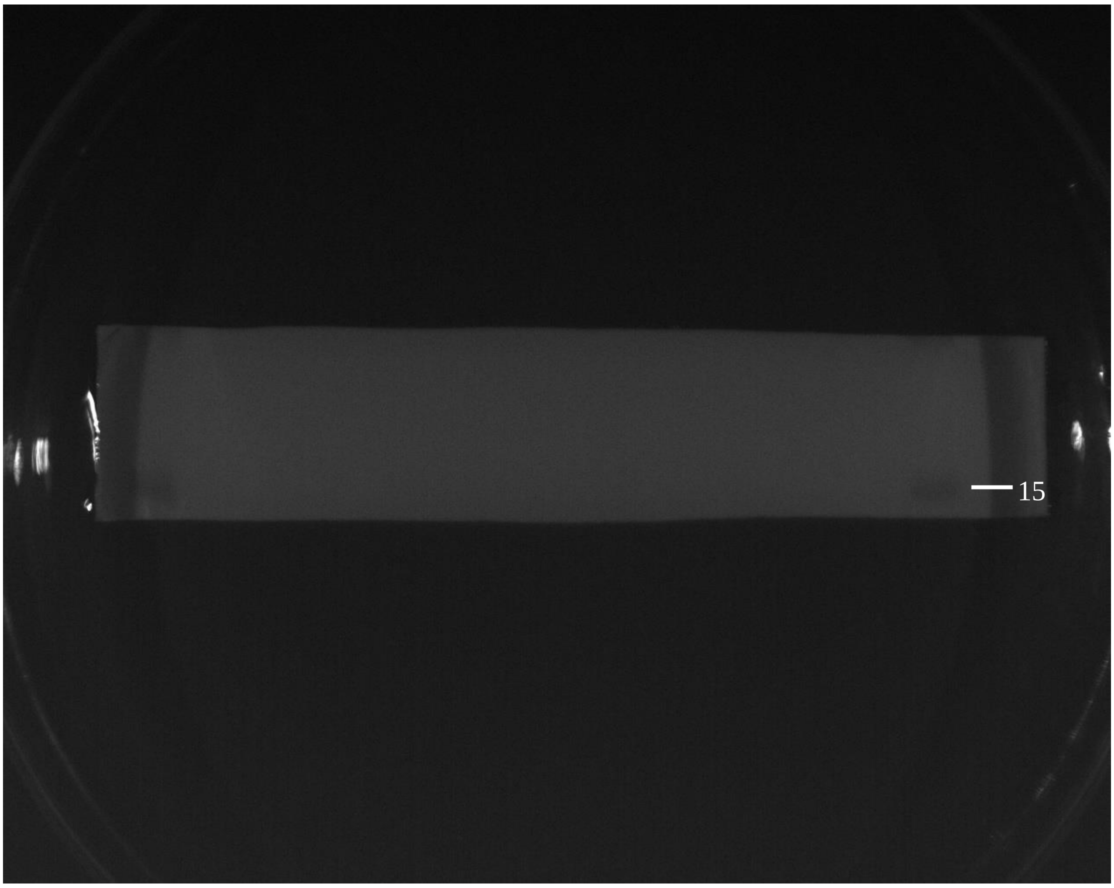

Full unedited blot for supplemental Figure 2a GAPDH

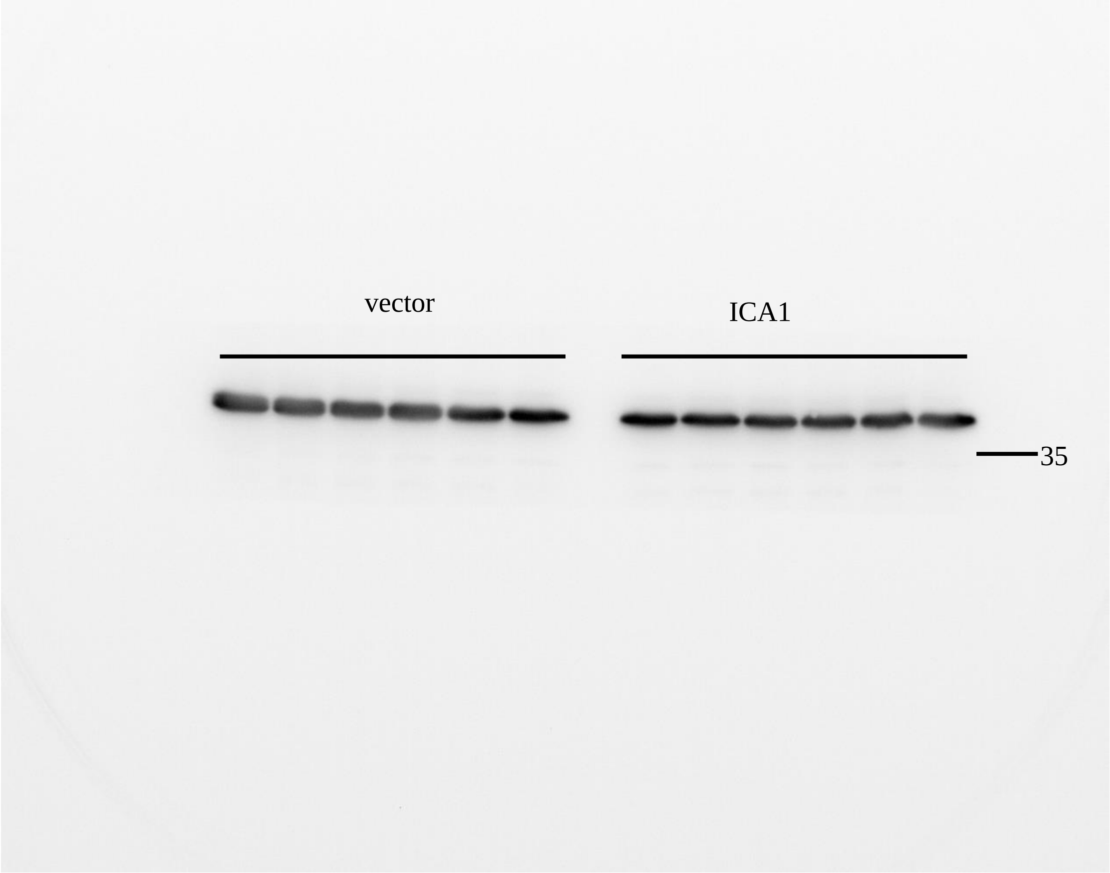

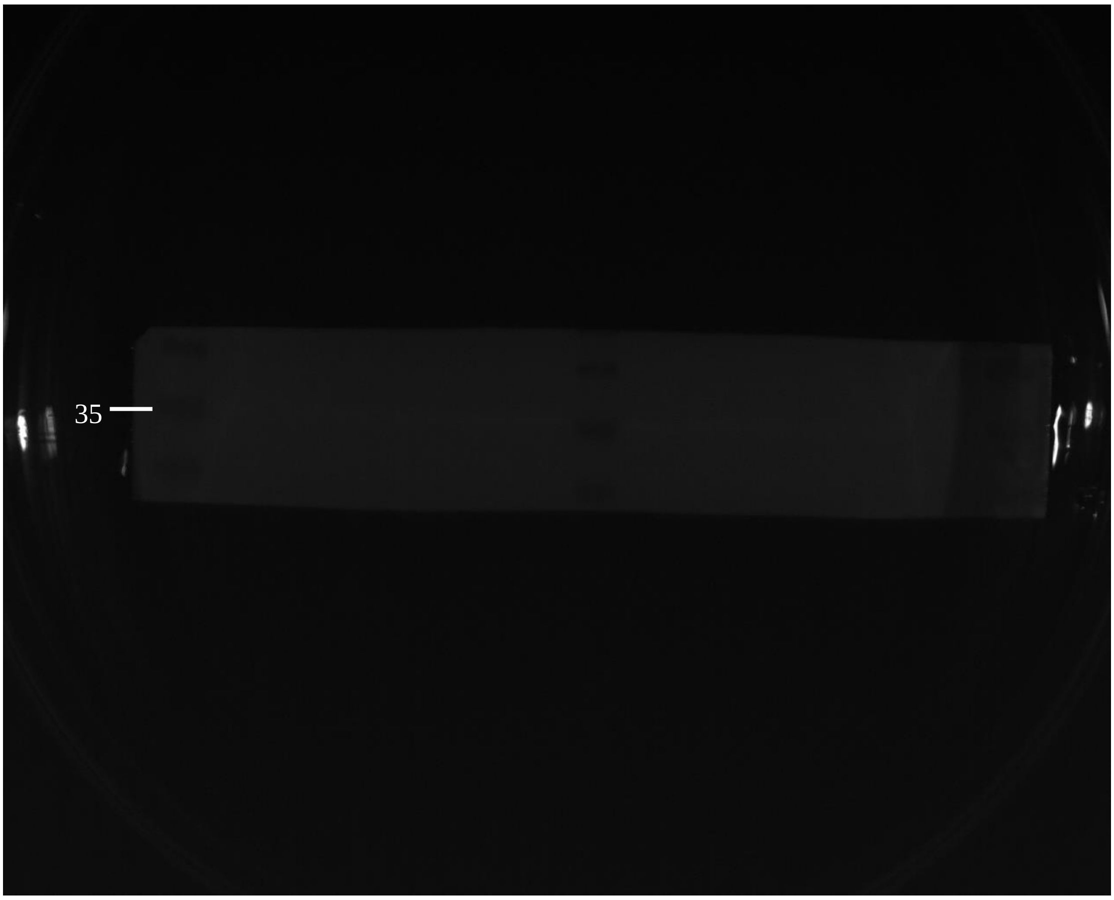

Full unedited blot for supplemental Figure 2b ICA1

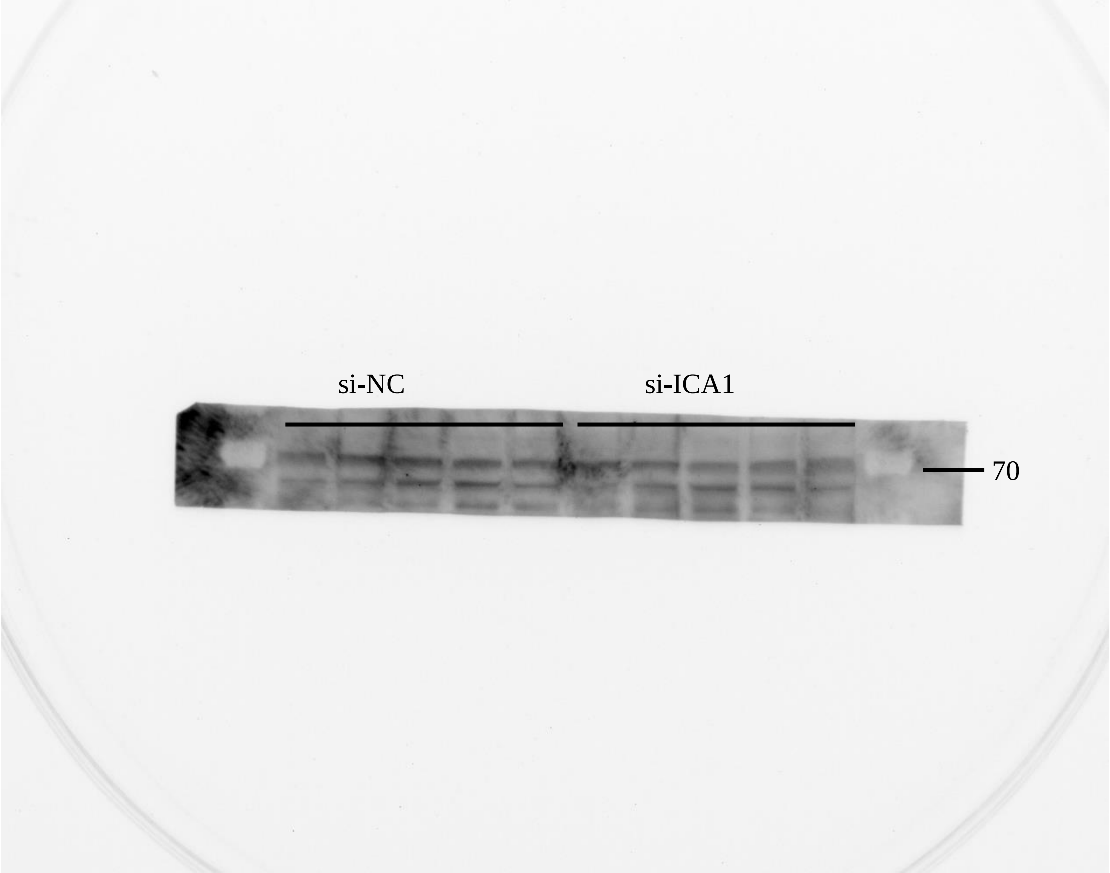

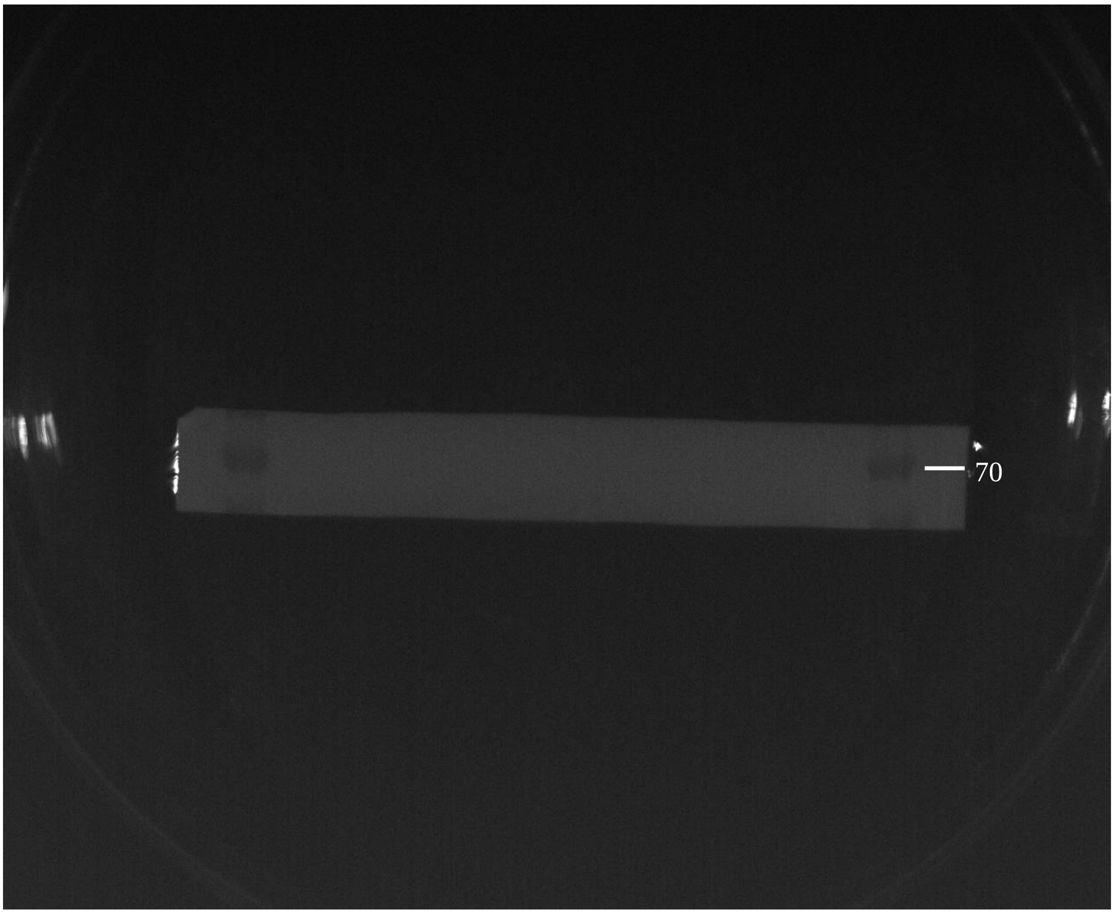

Full unedited blot for supplemental Figure 2b C89 and C99

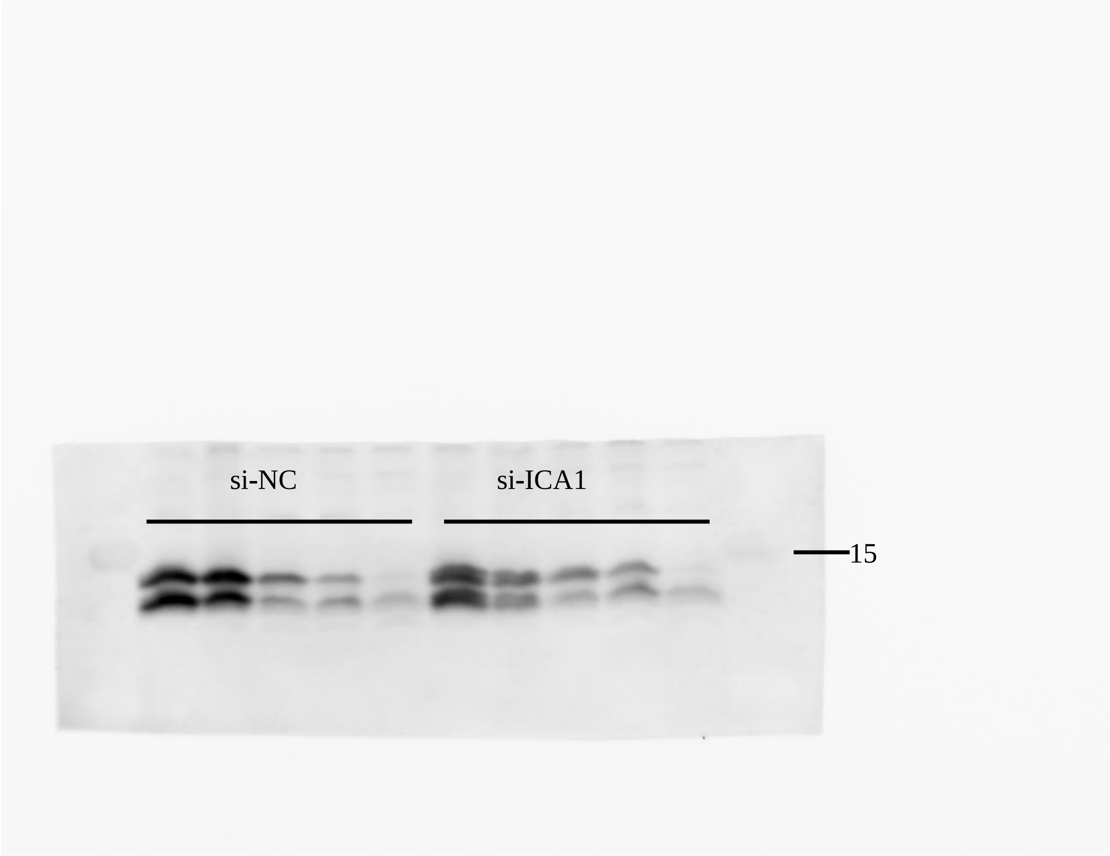

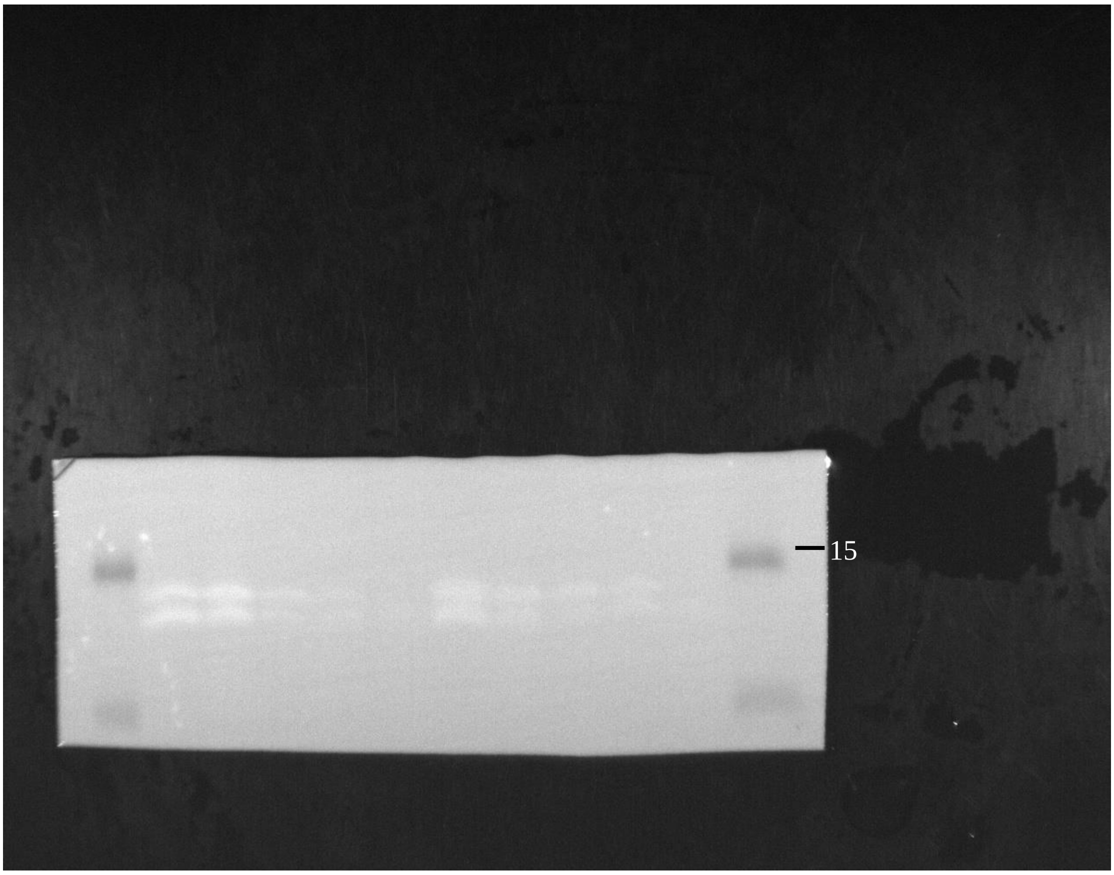

Full unedited blot for supplemental Figure 2b APP

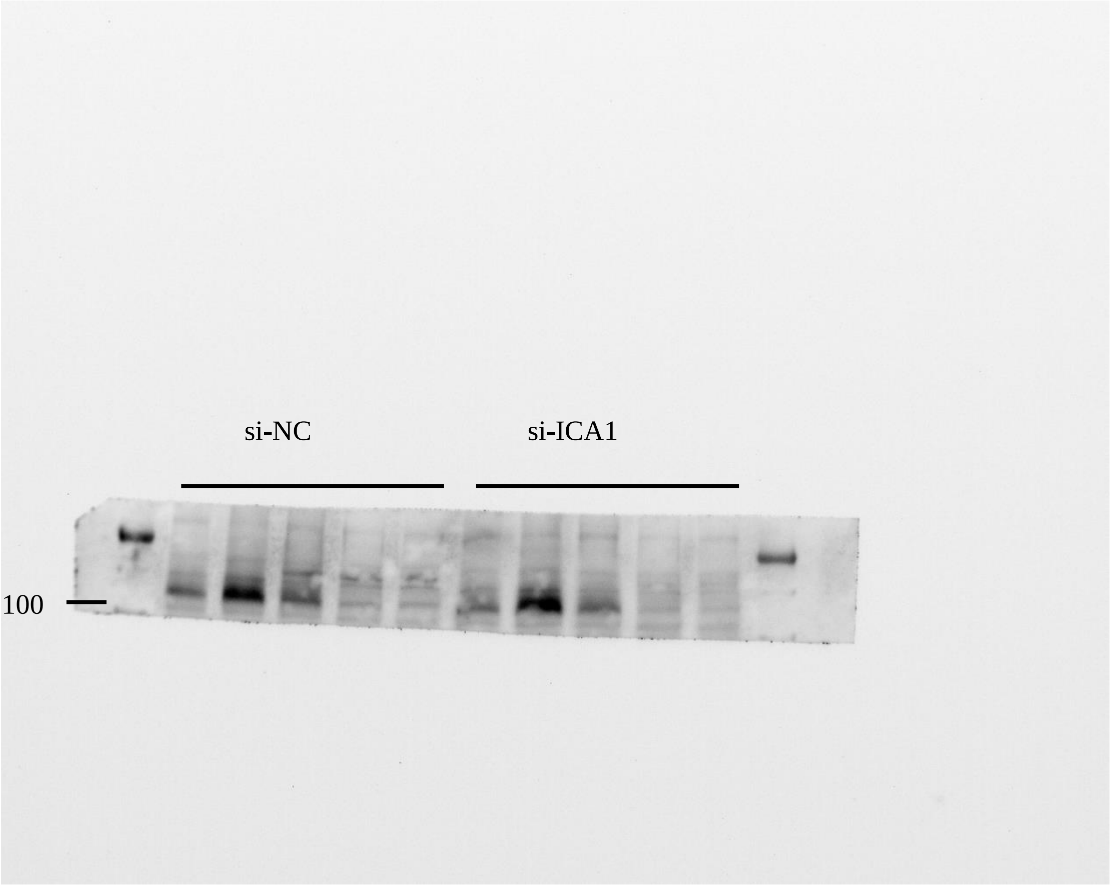

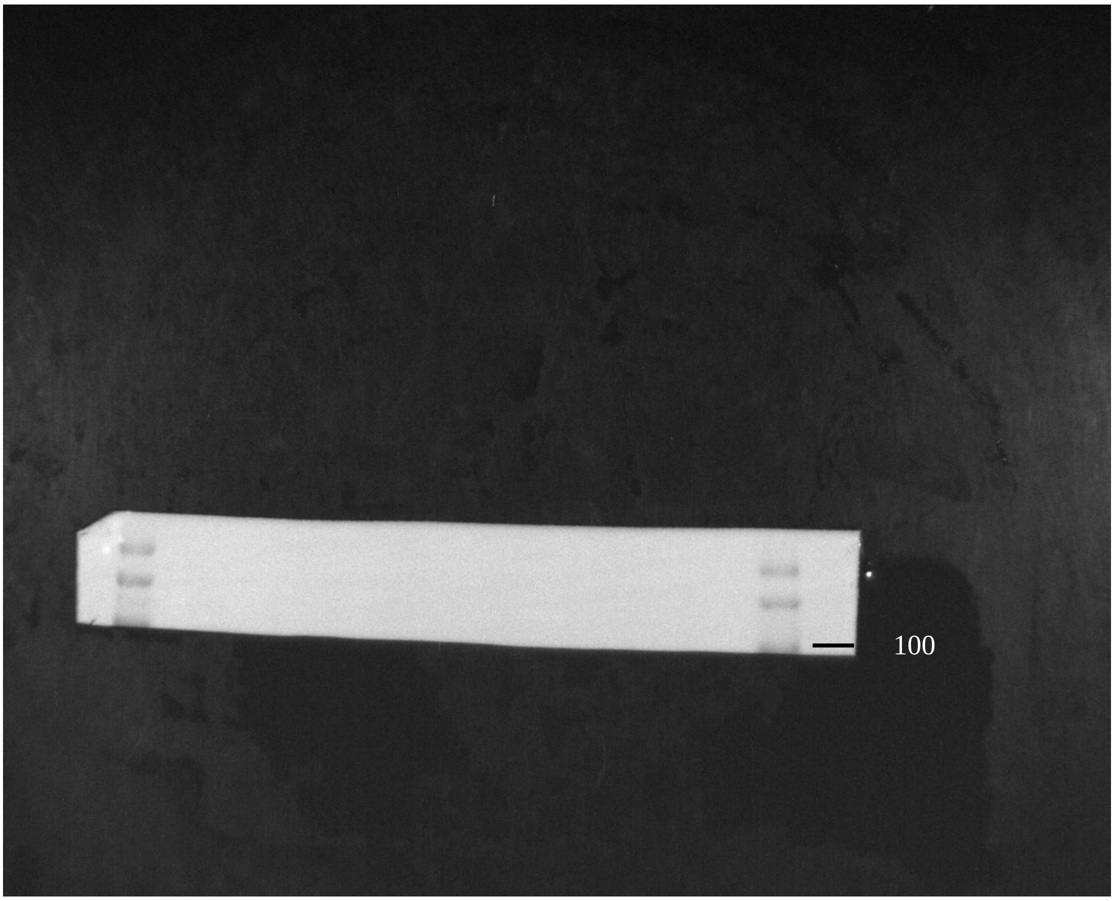

Full unedited blot for supplemental Figure 2b ADAM17

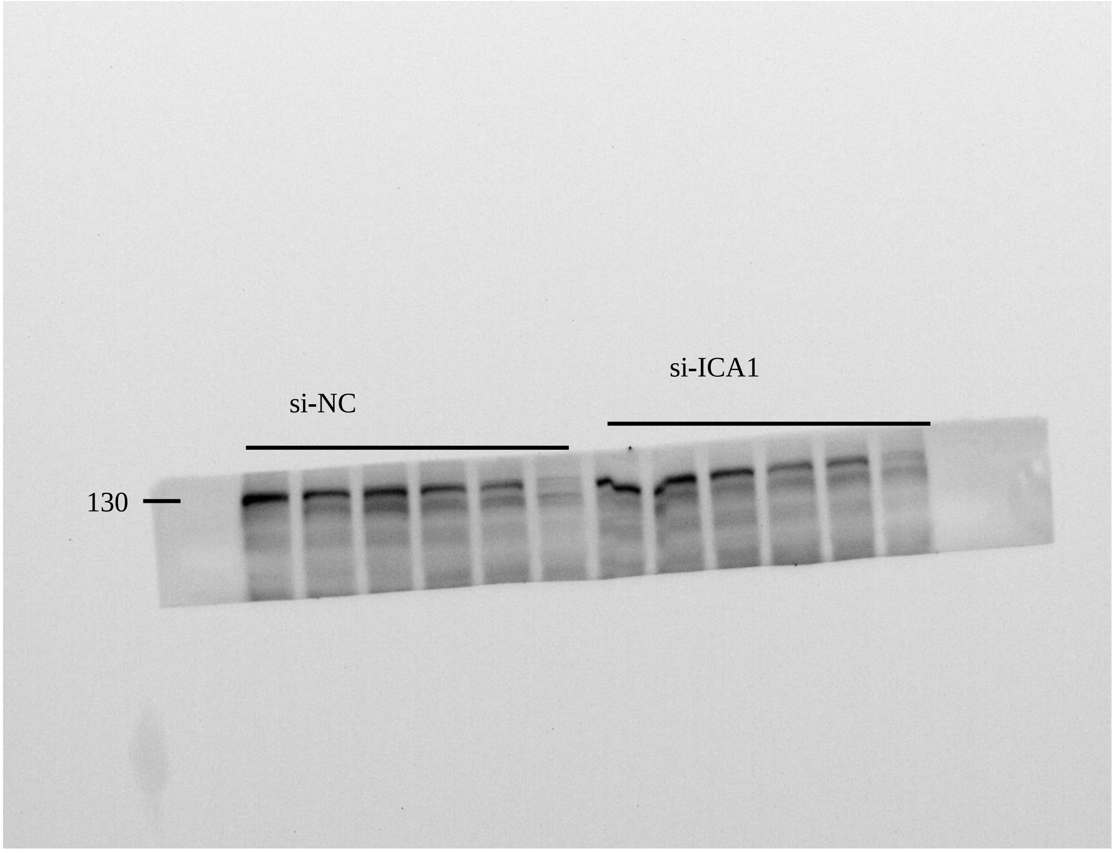

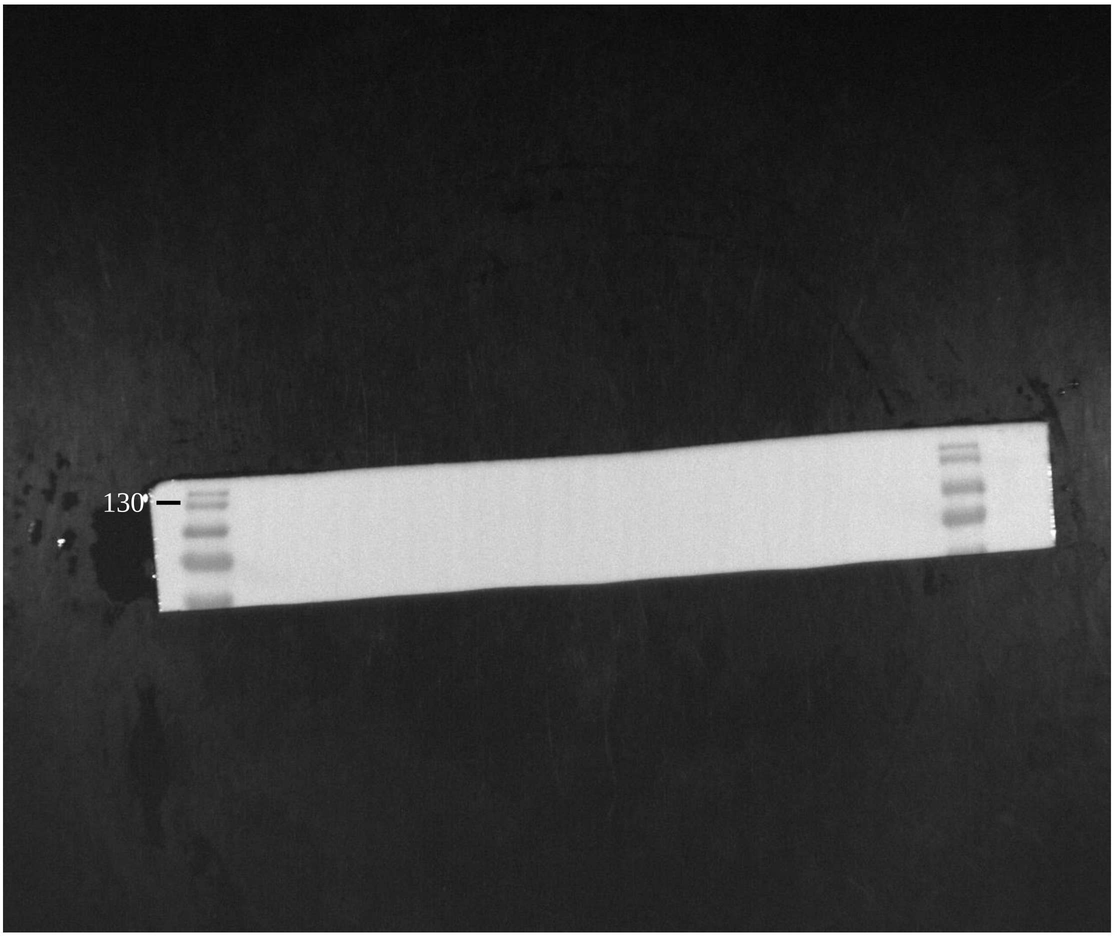

Full unedited blot for supplemental Figure 2b BACE1

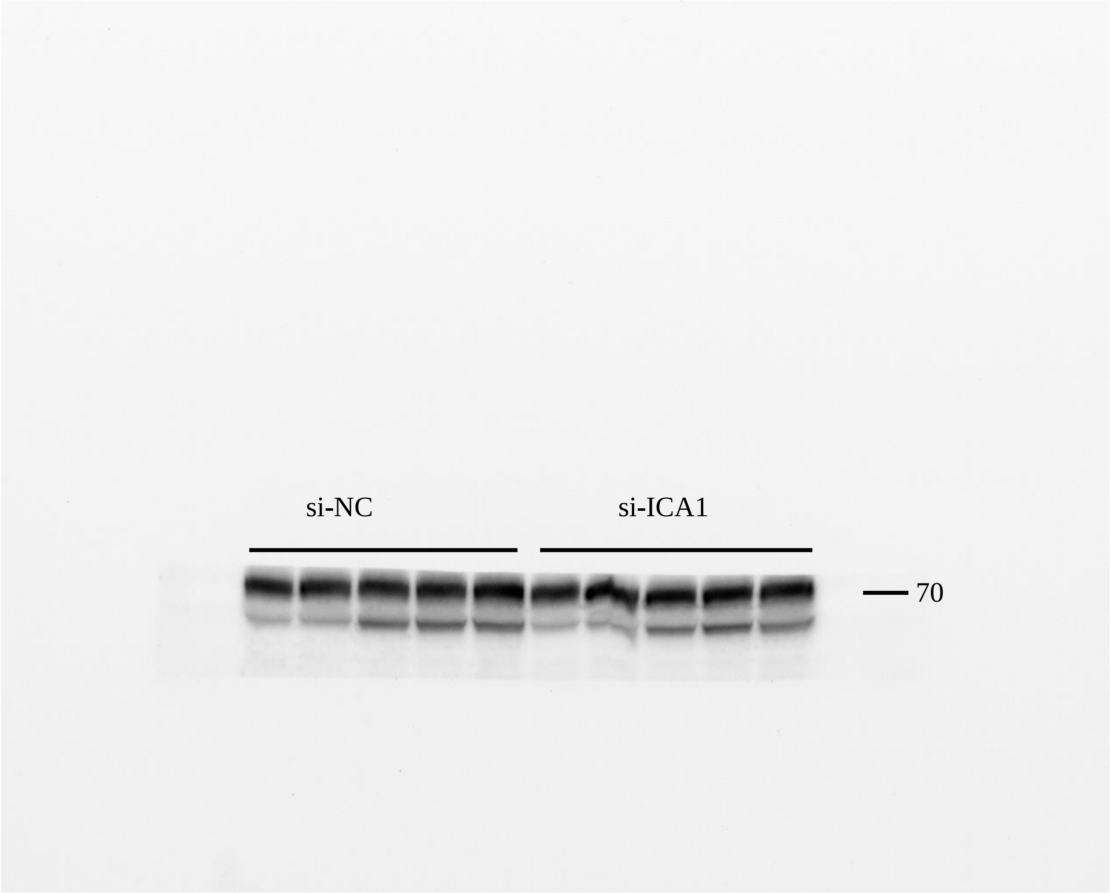

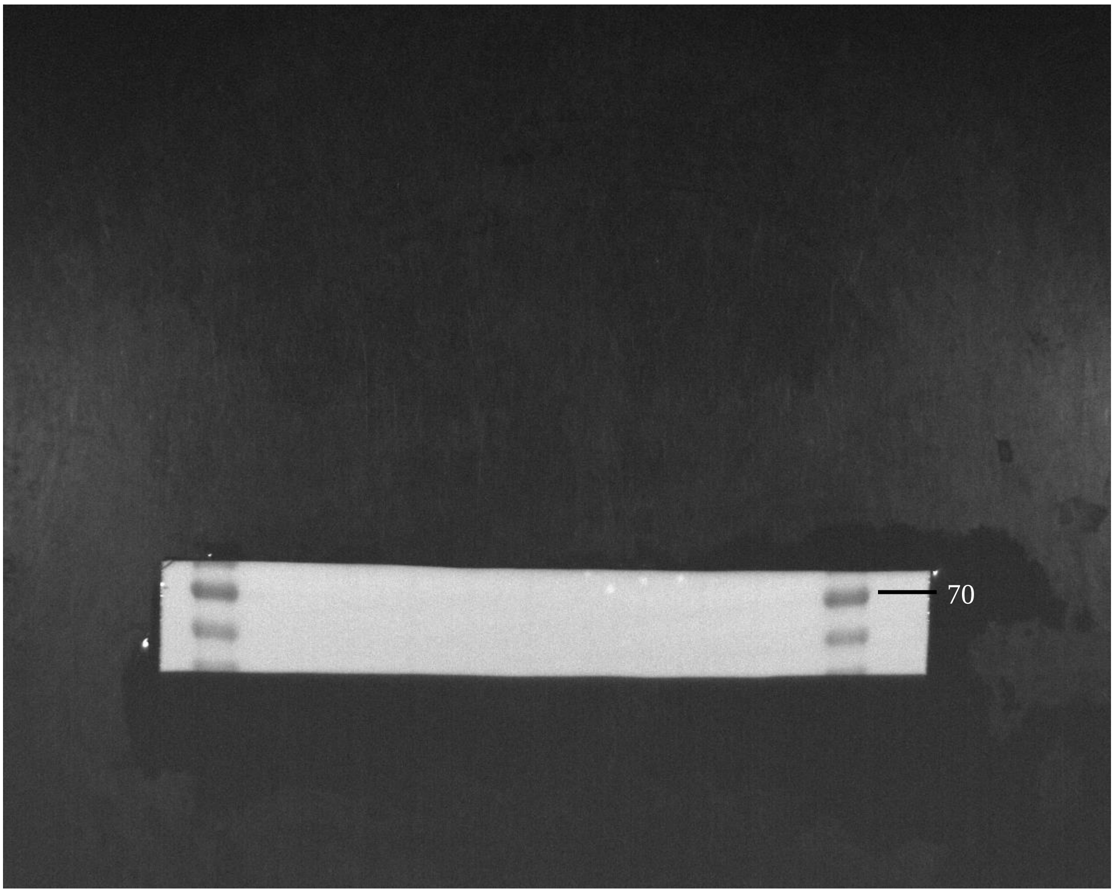

Full unedited blot for supplemental Figure 2b PS1

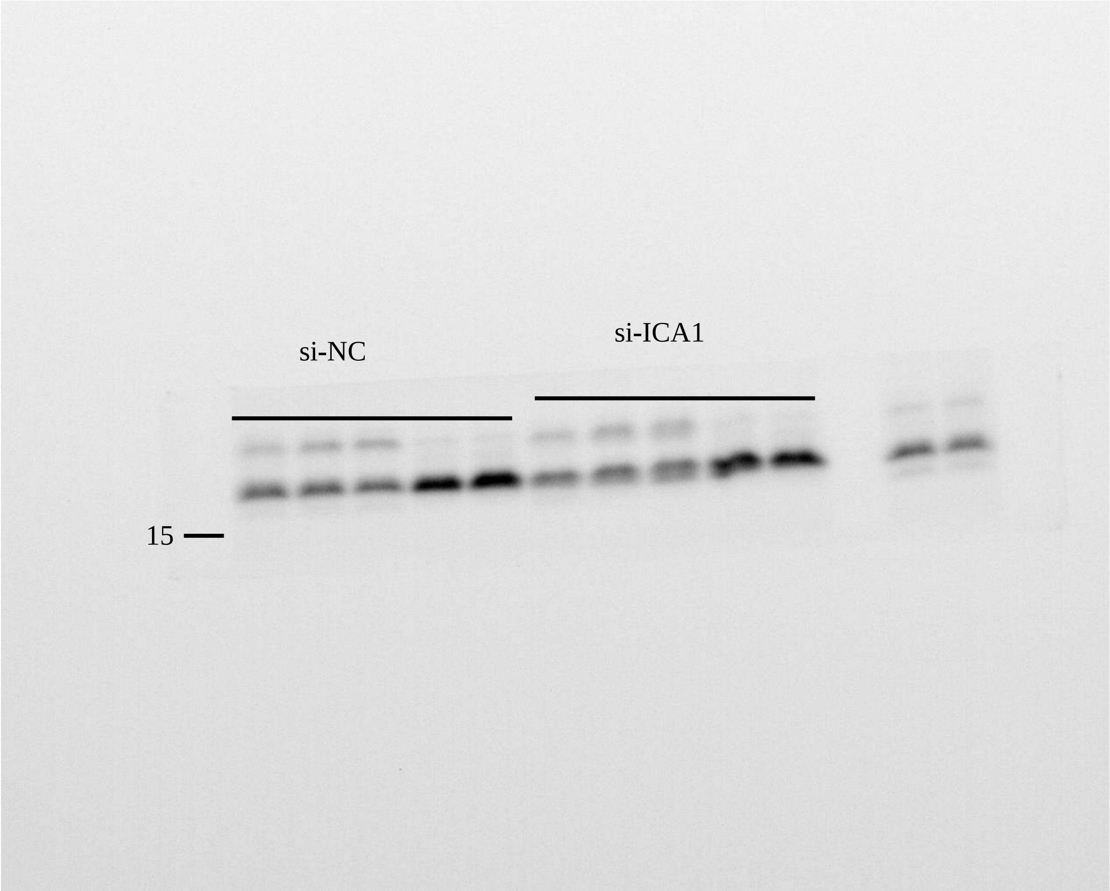

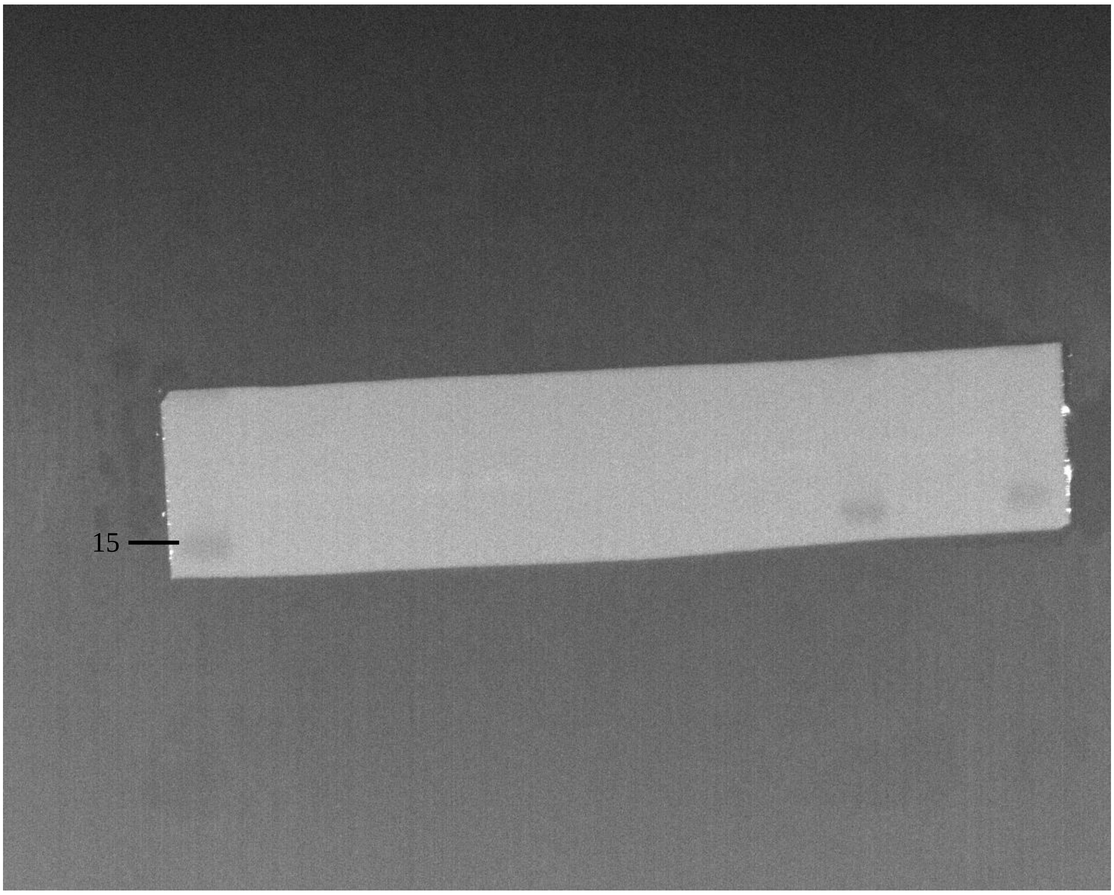

Full unedited blot for supplemental Figure 2b GAPDH

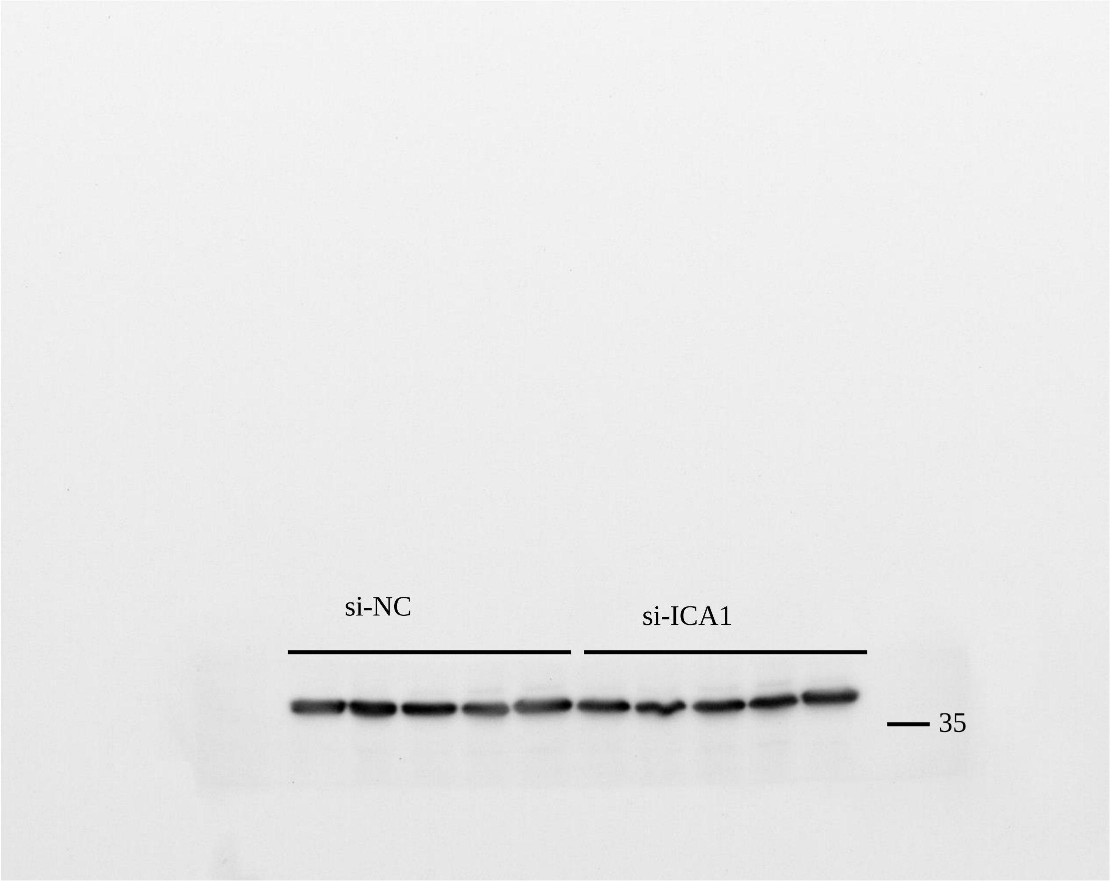

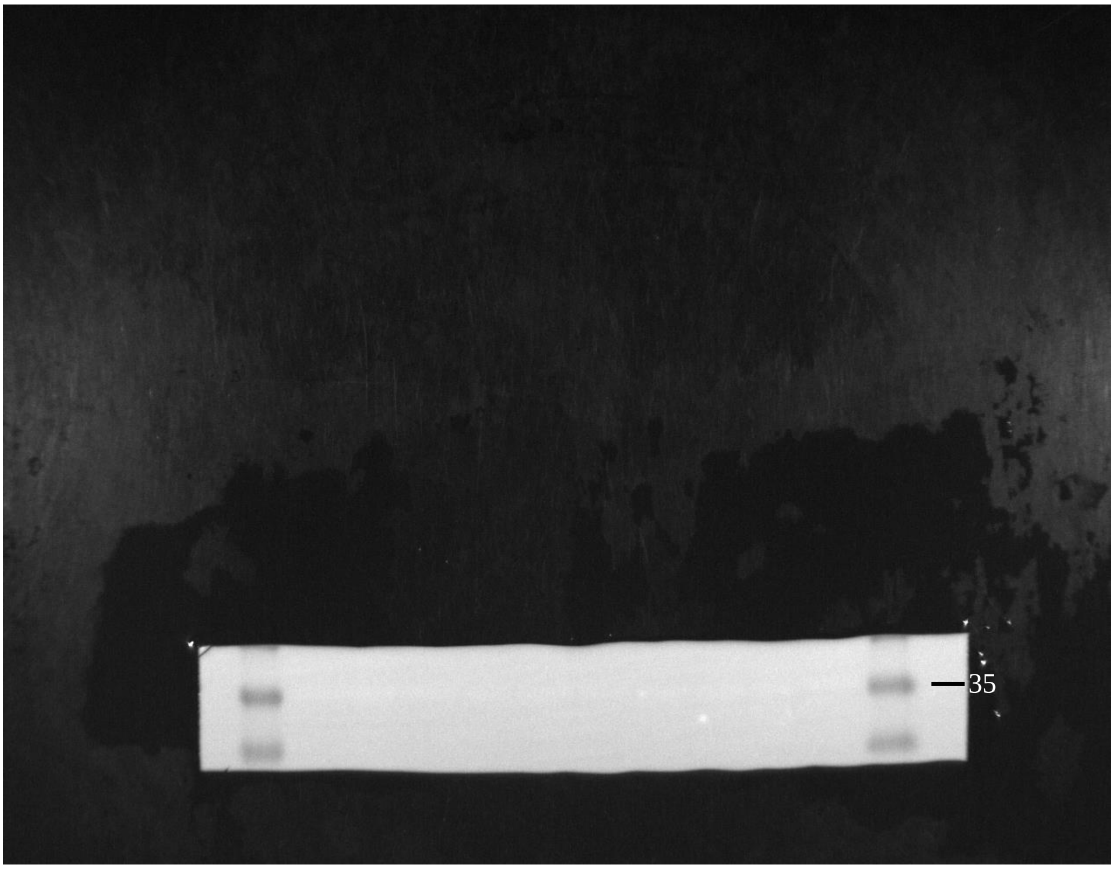

Full unedited blot for supplemental Figure 3a ICA1

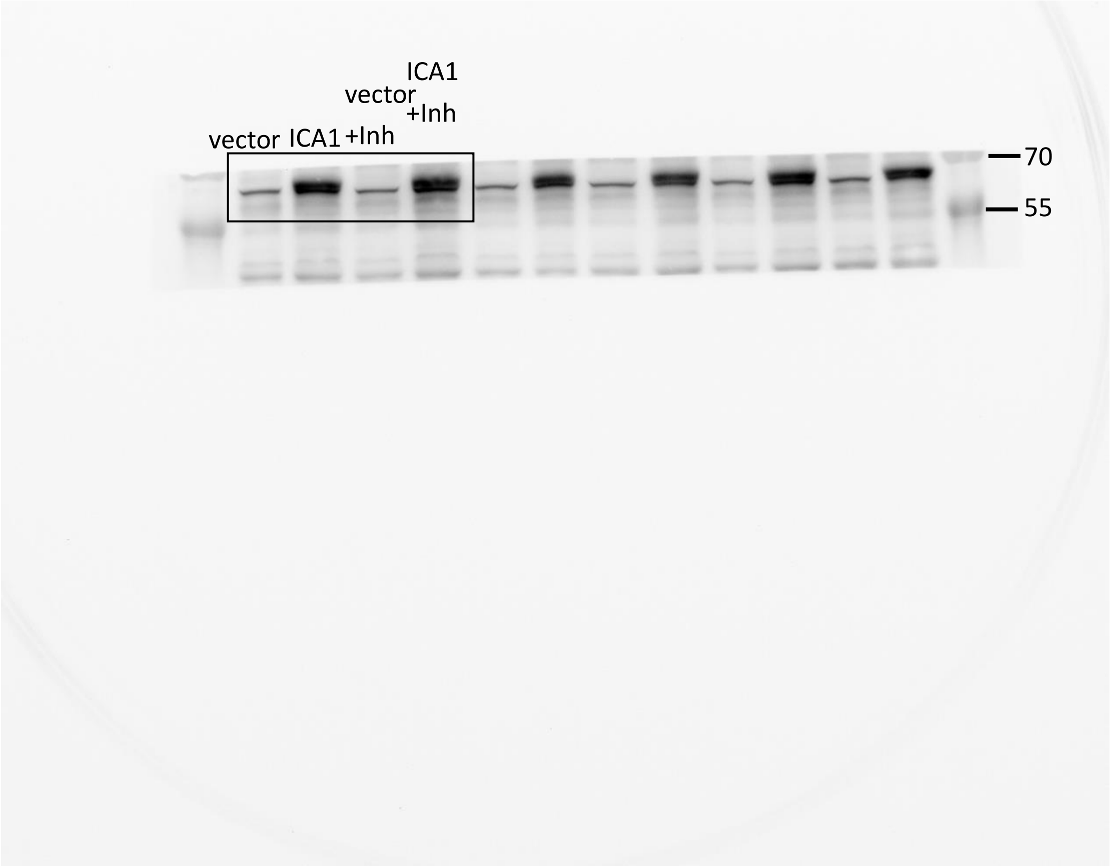

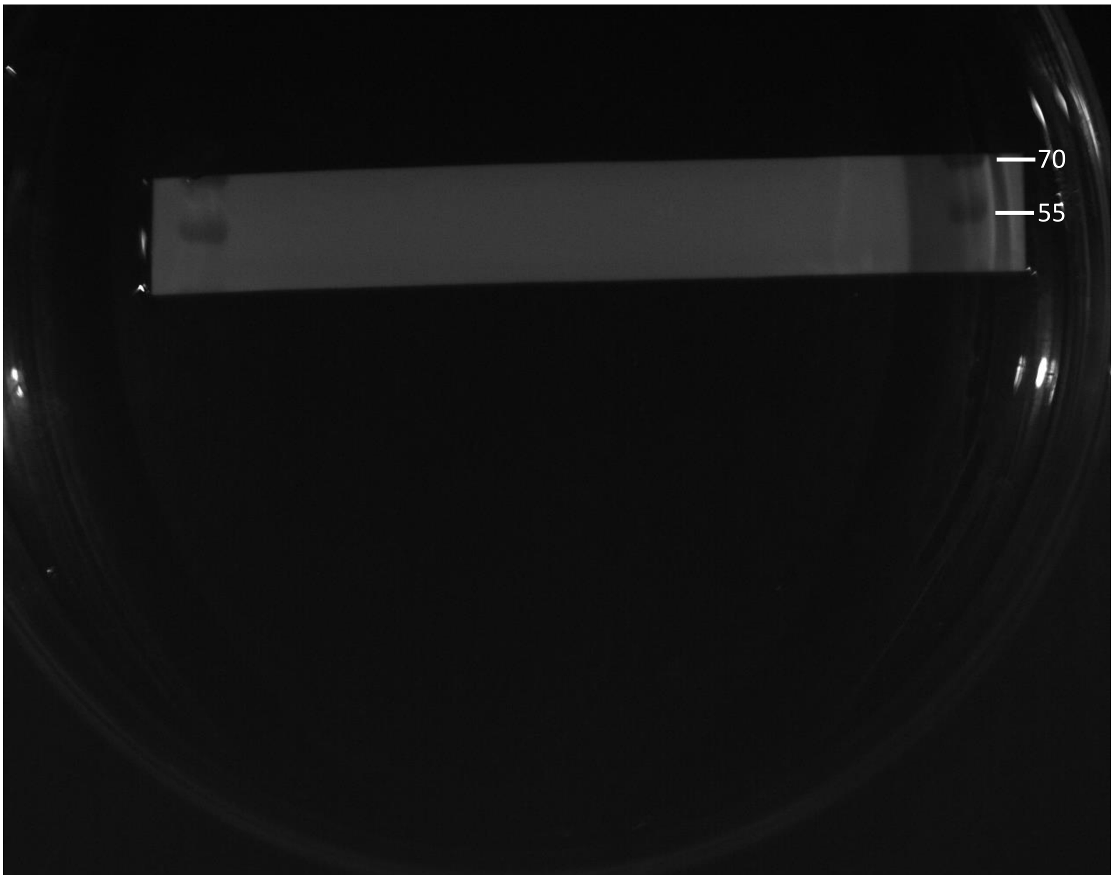

Full unedited blot for supplemental Figure 3a PICK1

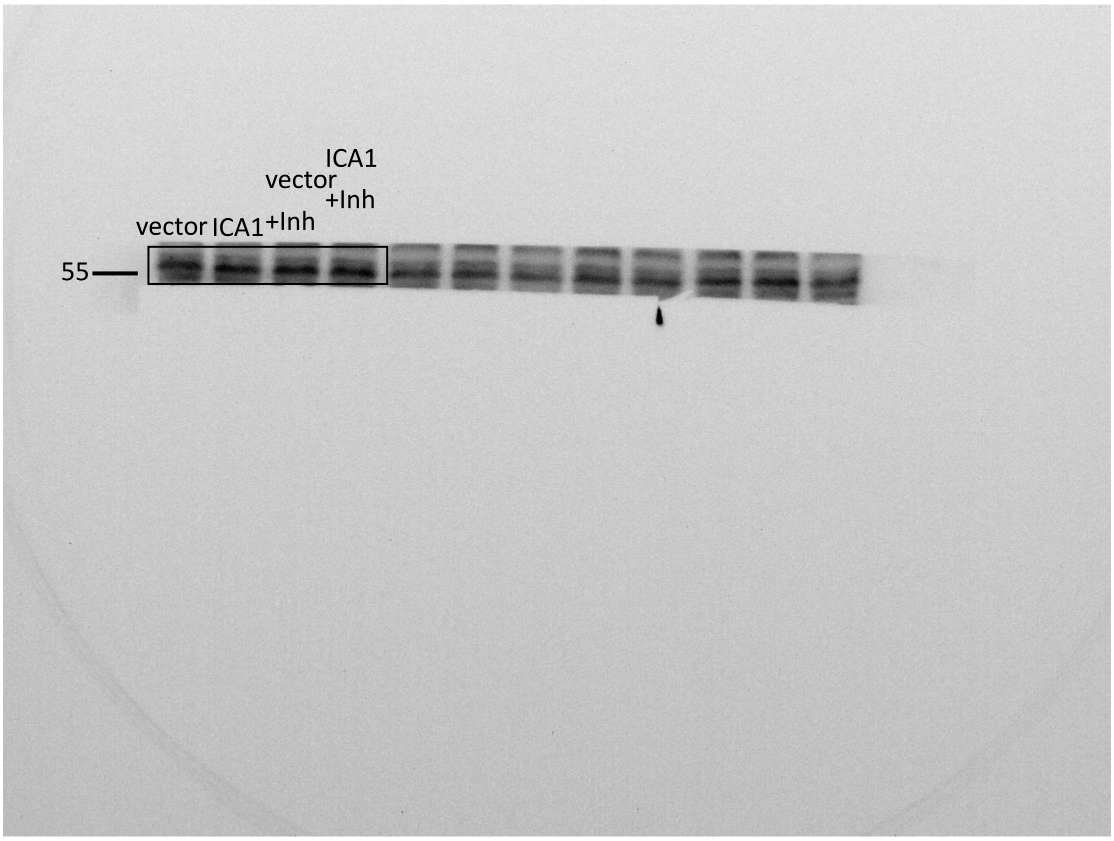

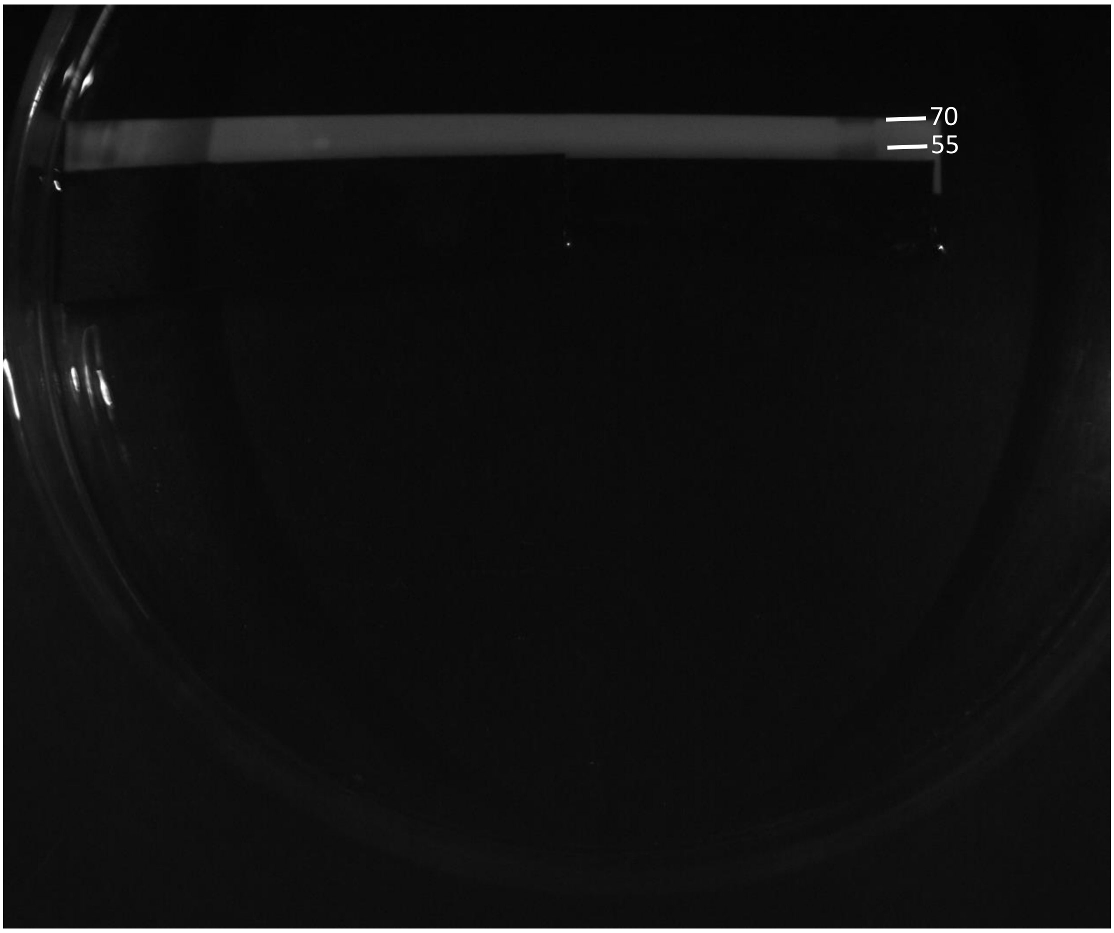

Full unedited blot for supplemental Figure 3a PKCα

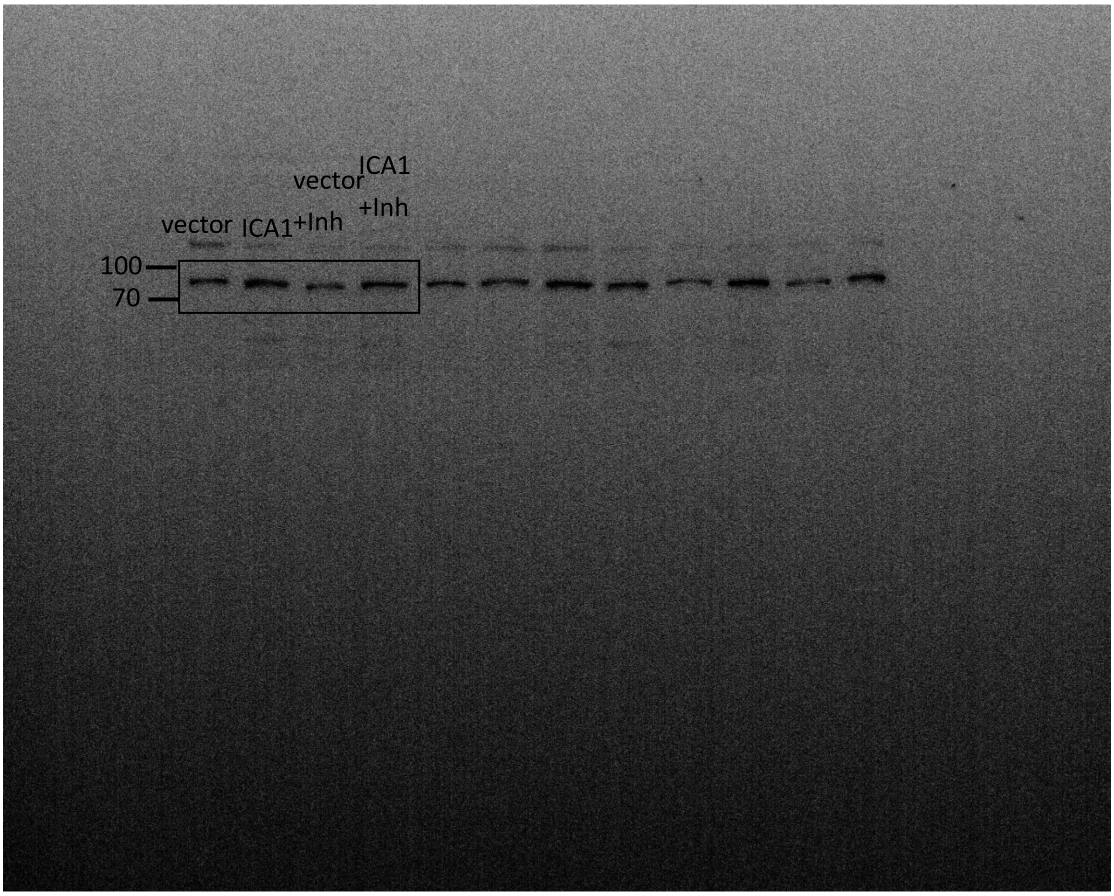

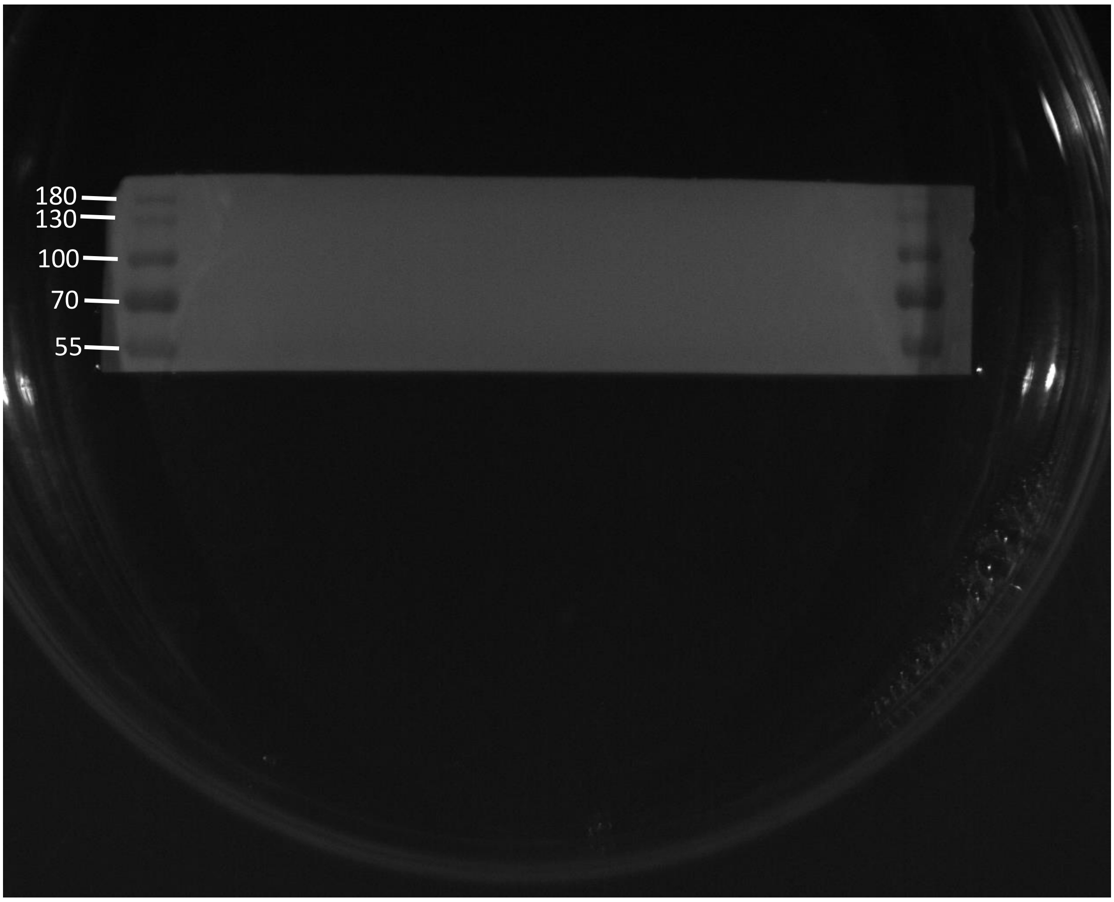

Full unedited blot for supplemental Figure 3a p-PKCα

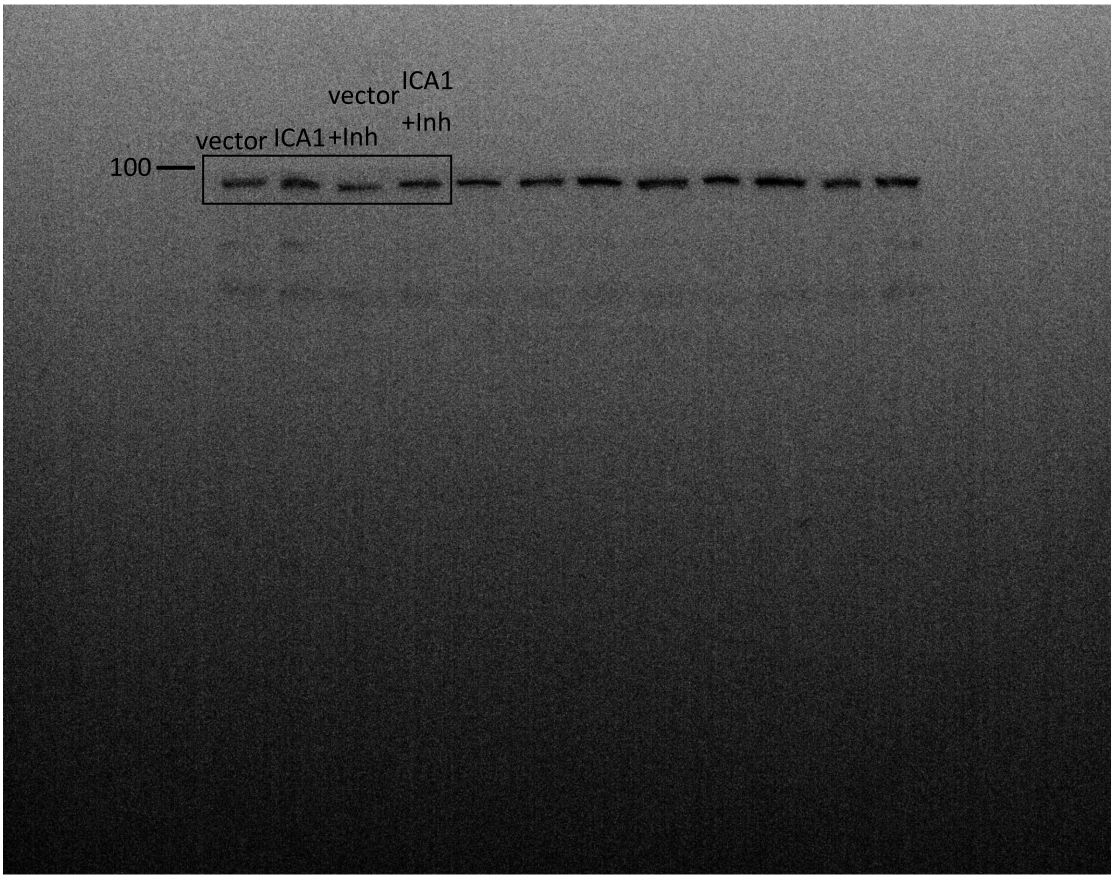

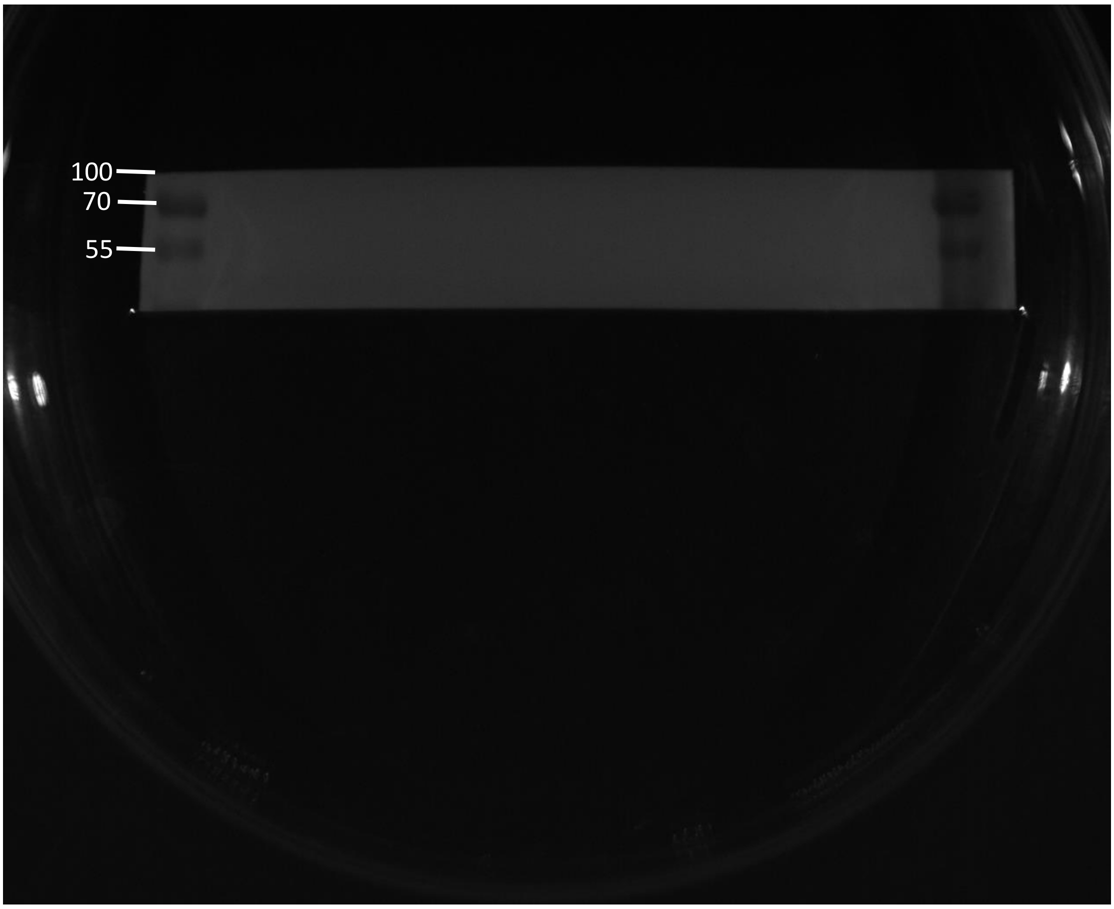

Full unedited blot for supplemental Figure 3a C83

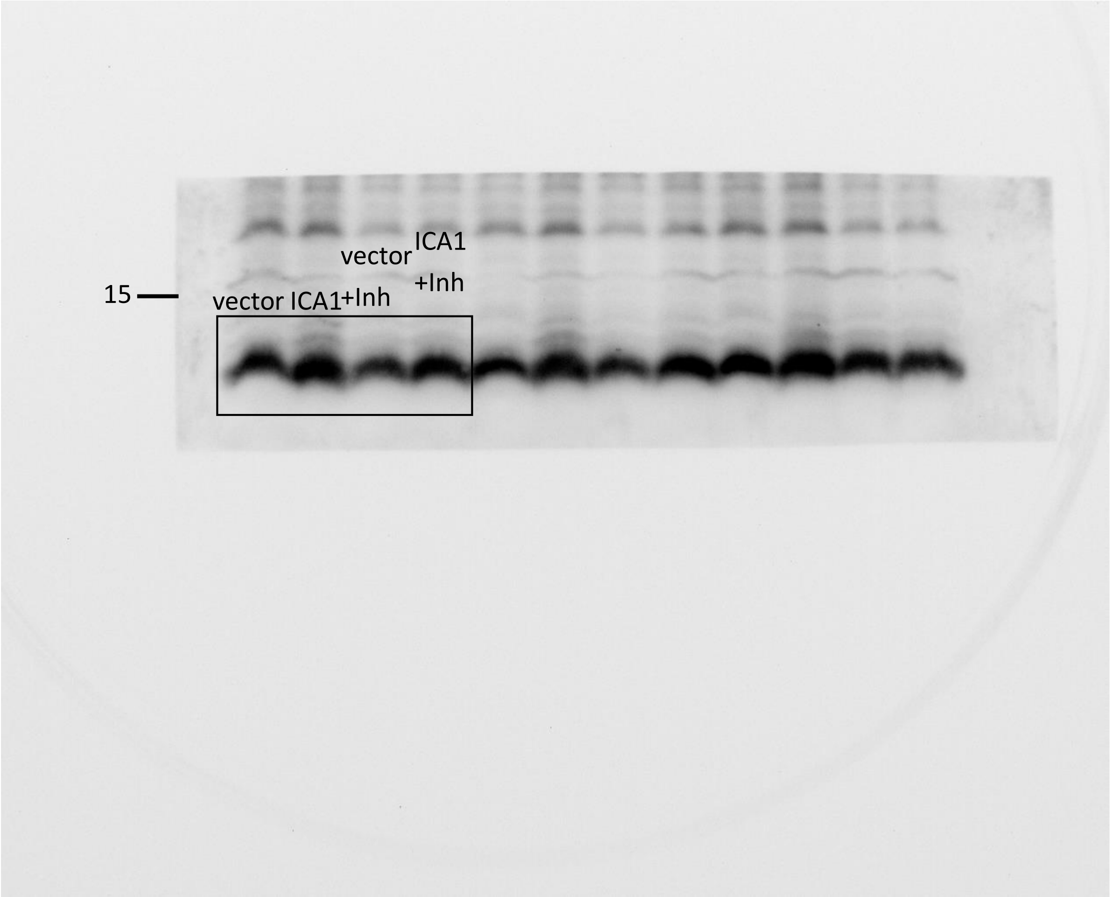

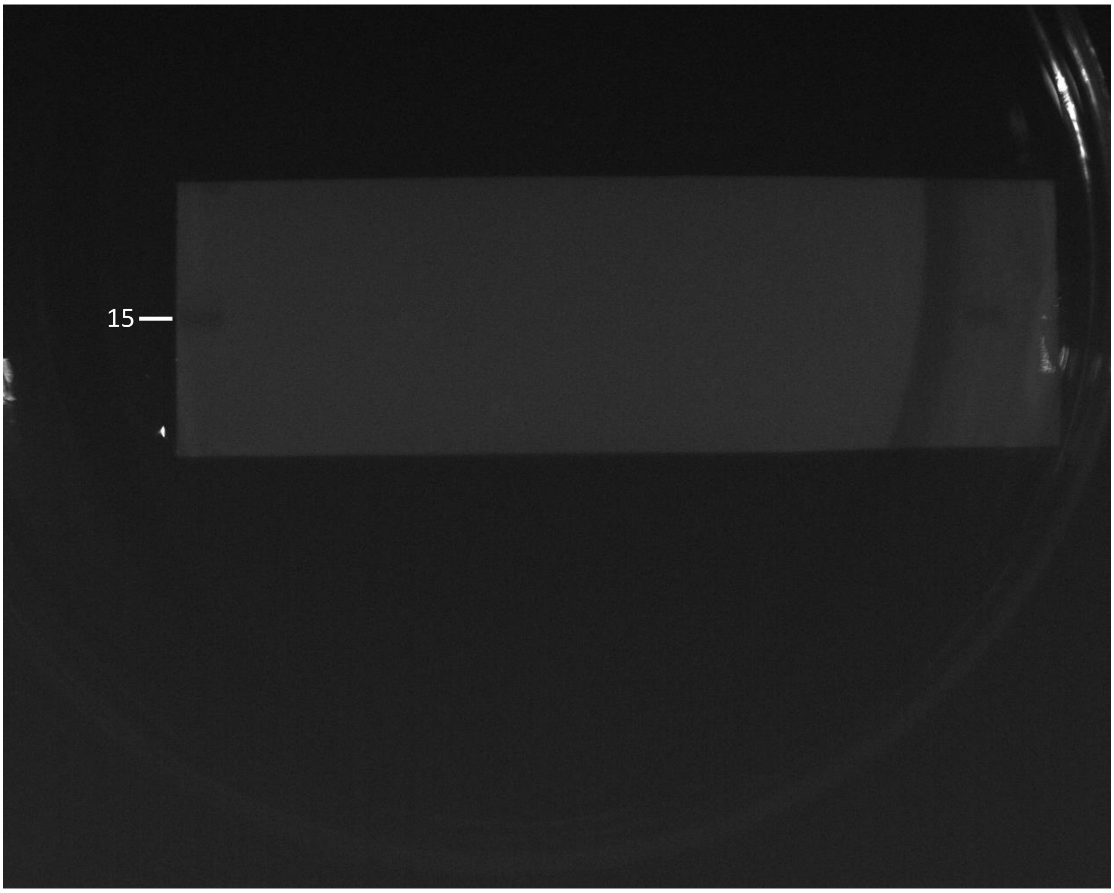

Full unedited blot for supplemental Figure 3a APP

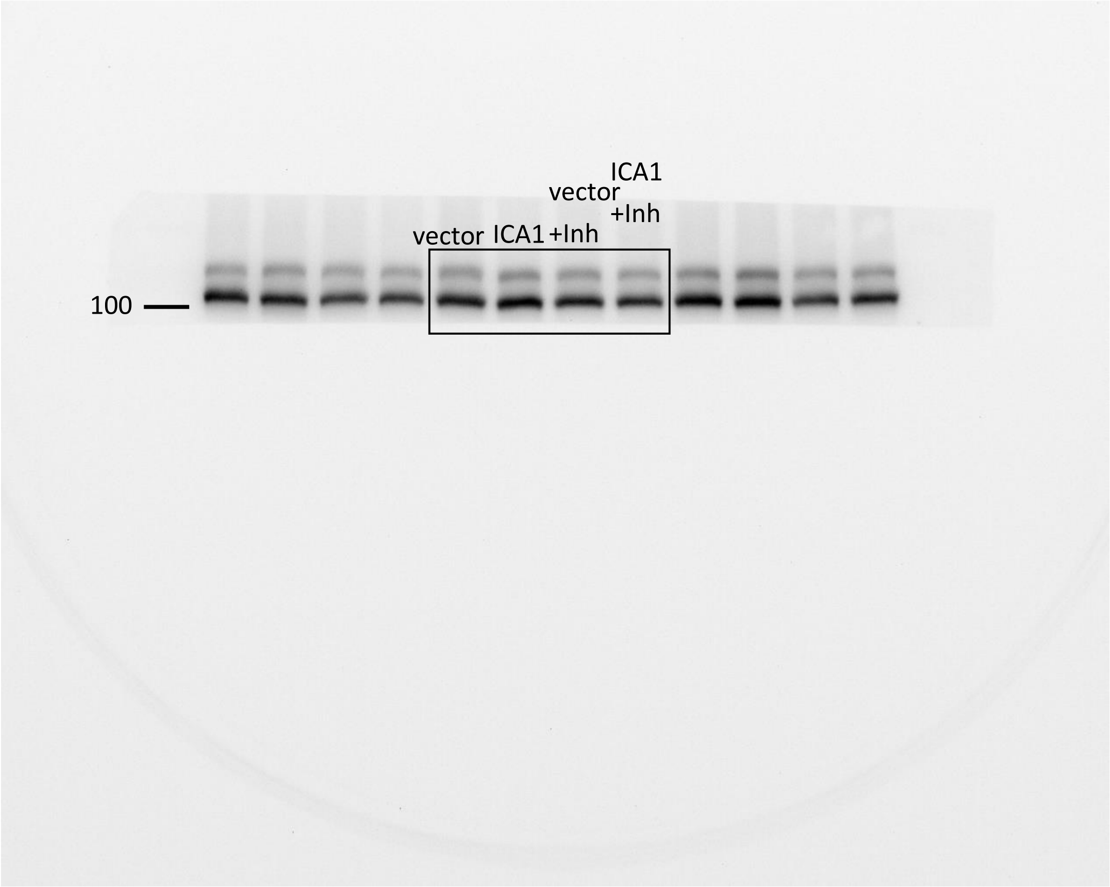

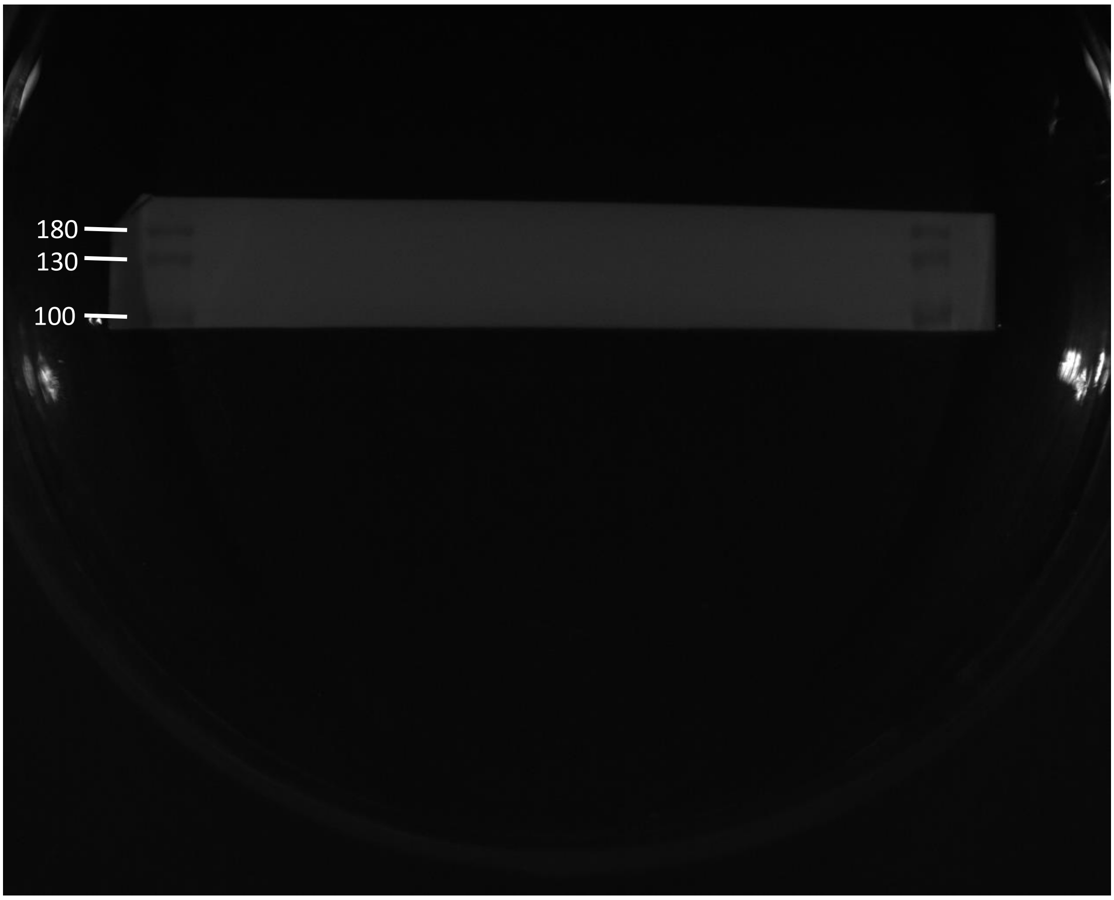

Full unedited blot for supplemental Figure 3a ADAM10

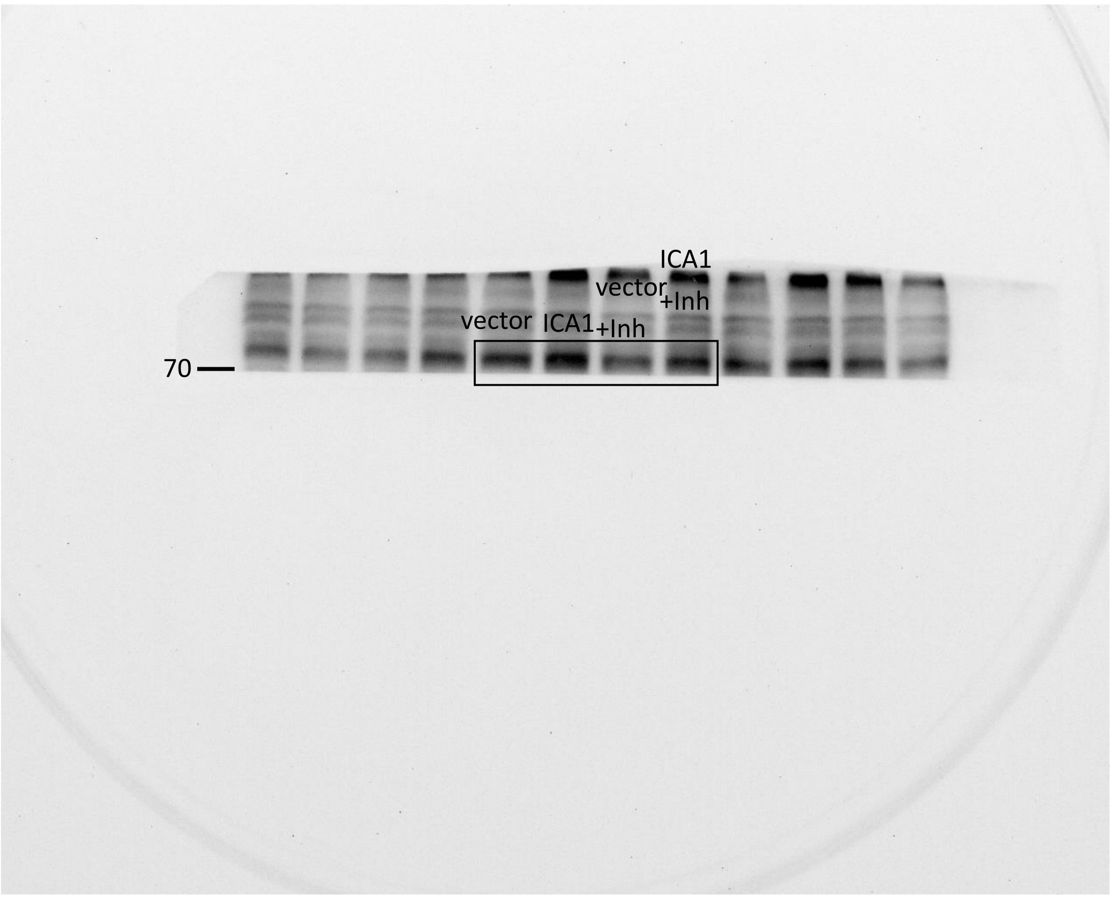

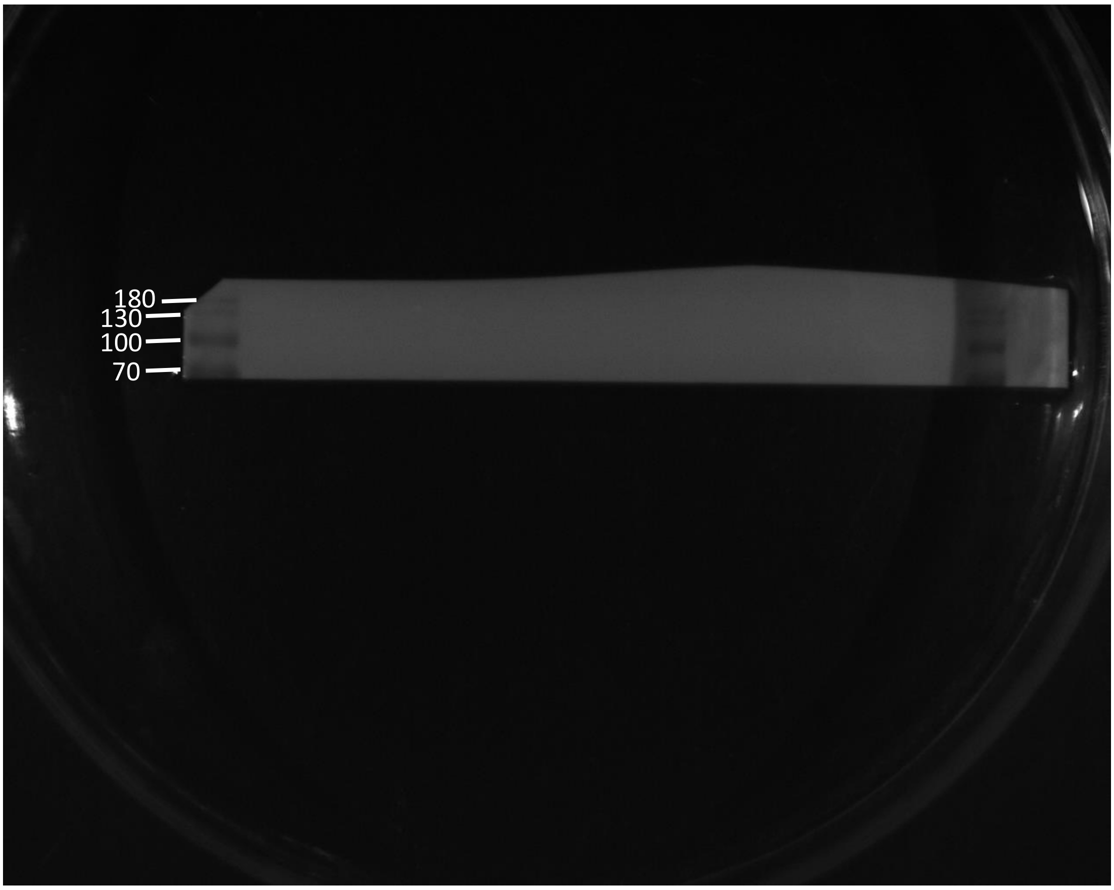

Full unedited blot for supplemental Figure 3a ADAM17

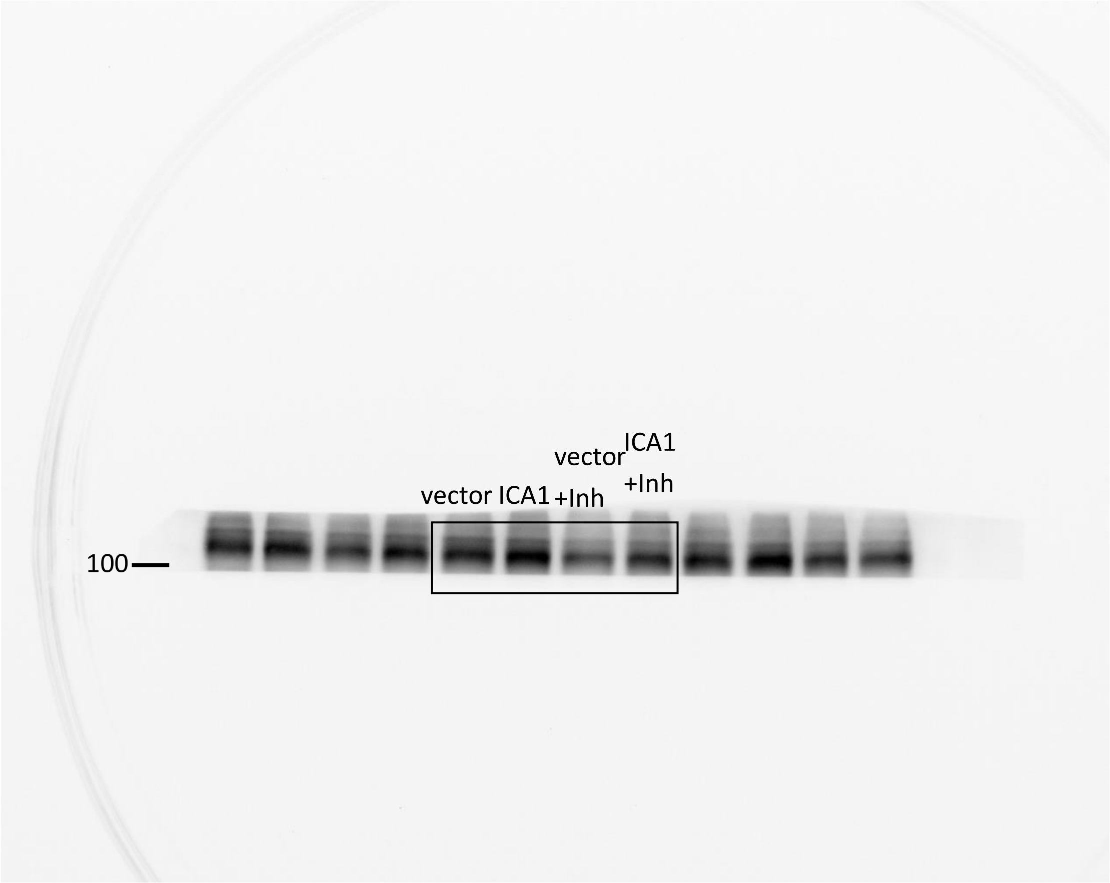

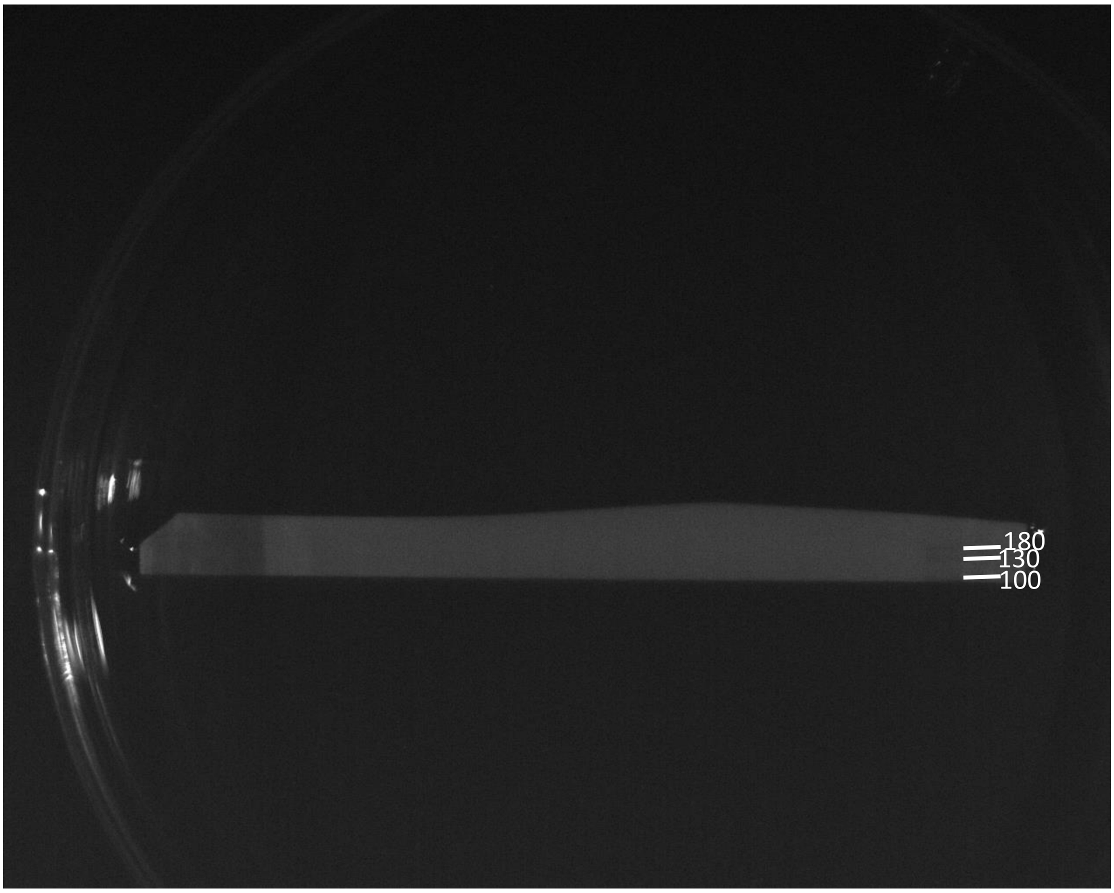

Full unedited blot for supplemental Figure 3a GAPDH

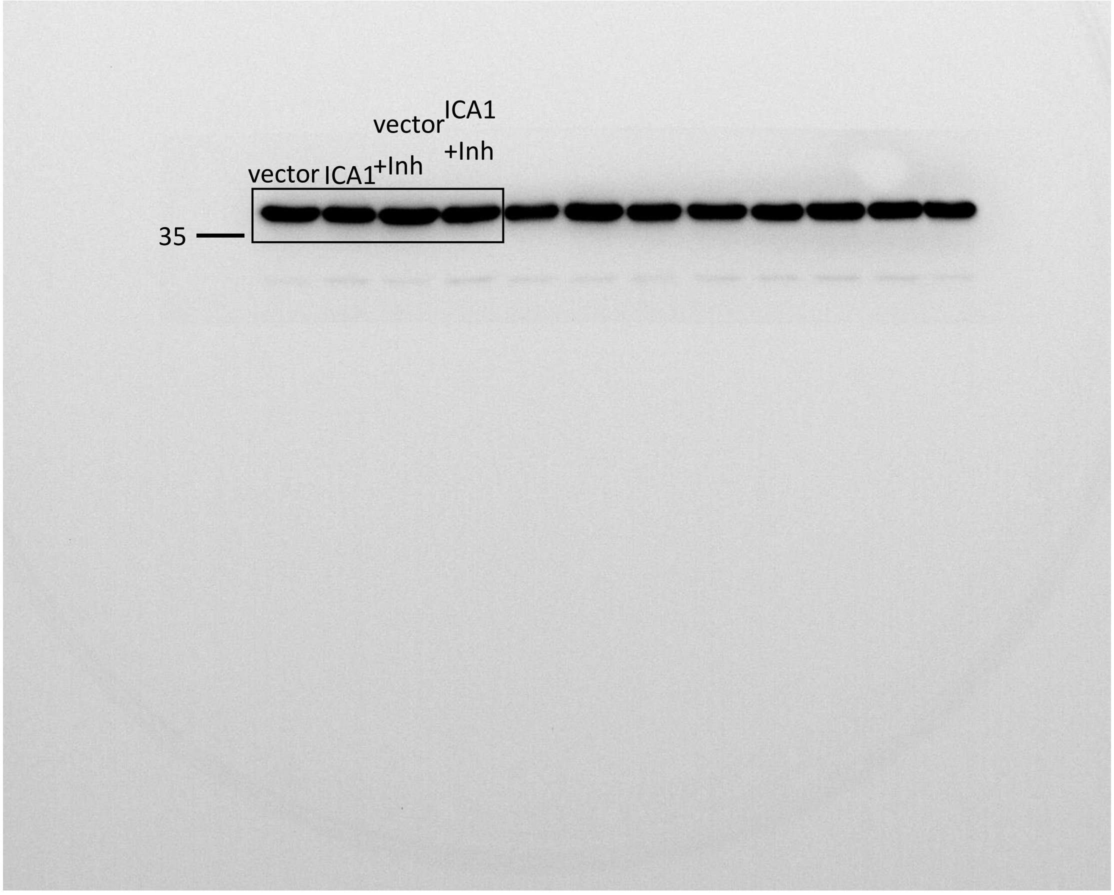

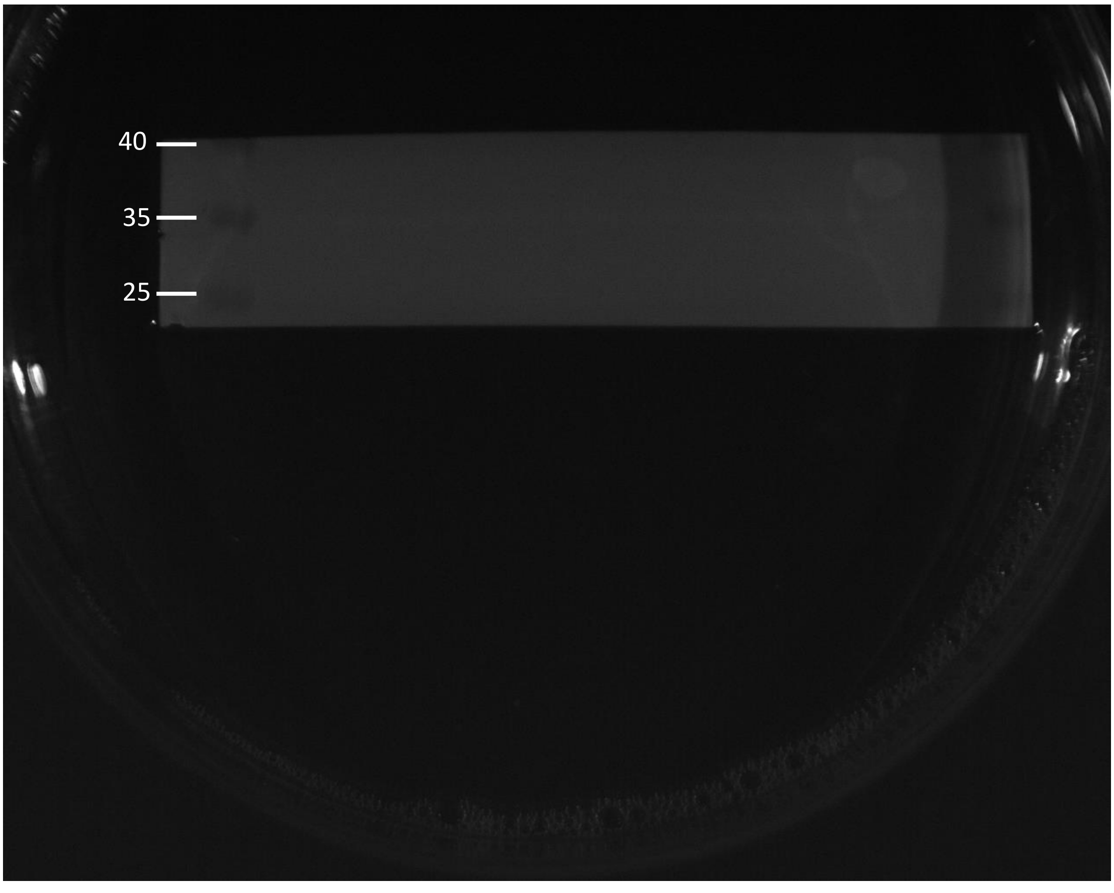

Supplement: Supplementary file 2 — File S1. [file CNS-30-e14754-s004.pdf]
