# Supplementary material for: ICA1 affects APP processing through the PICK1‐PKCα signaling pathway
Source: CNS Neurosci Ther. 2024 Jun 17;30(6):e14754. doi: 10.1111/cns.14754 (PMC11181291; doi:10.1111/cns.14754)
Supplement: Supplementary file 4 — Table S1. [file CNS-30-e14754-s003.zip › supplementary table legend.docx]

**Table Legends**

**Supplemental Table The differential genes in ICA1 knockdown 20E2 cells.**

Differential expression analysis identified 581 genes, of which 283 genes were significantly up-regulated and 298 genes were significantly down-regulated in ICA1 knockdown 20E2 cells. GO enrichment analysis and KEGG enrichment analysis of differential genes were conducted.
